# Supplementary material for: Radical aryl migration enables diversity-oriented synthesis of structurally diverse medium/macro- or bridged-rings
Source: Nat Commun. 2016 Dec 22;7:13852. doi: 10.1038/ncomms13852 (PMC5192181; doi:10.1038/ncomms13852)
Supplement: Supplementary Information — Supplementary figures, supplementary tables, supplementary notes, supplementary methods and supplementary references. [file ncomms13852-s1.pdf]

Supplementary Figures

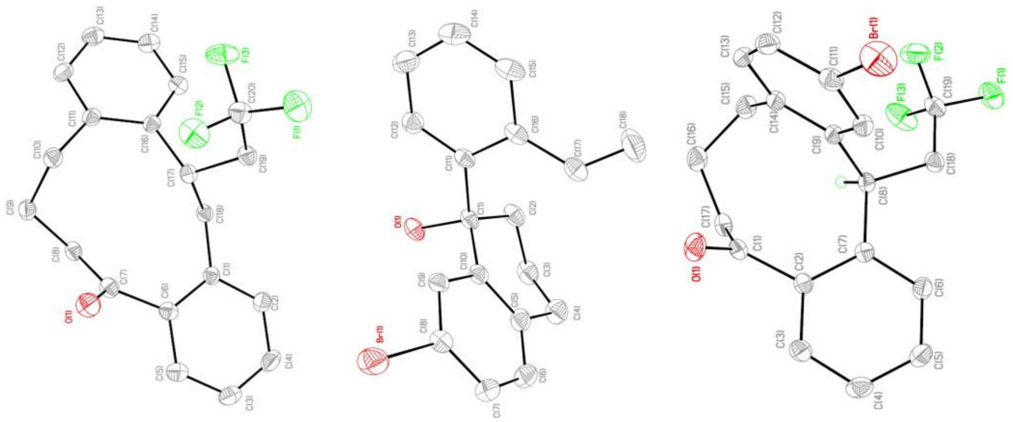

4O | (*R*)-**1A** | (*R*)-**4A**

Supplementary Figure 1. X-ray structure for **4O**, (*R*)-**1A** and (*R*)-**4A**.

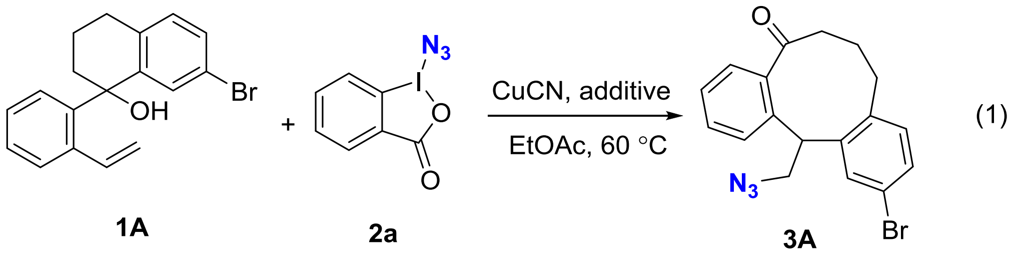

additive: BHT, 0% yield of **3A**
TEMPO, 28% yield of **3A**

Equation 1: azidation reaction in presence of radical scavengers

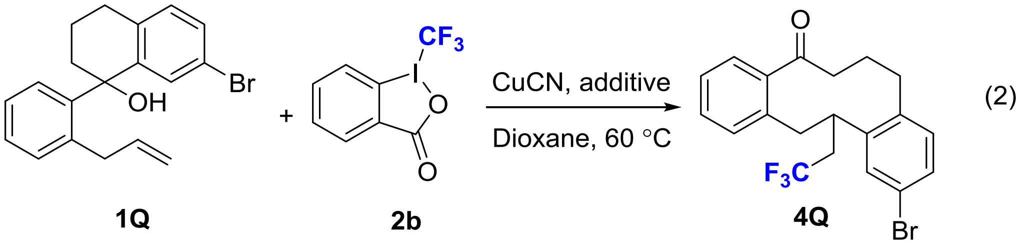

additive: TEMPO, 0% yield of **4Q**
1,4-dinitrobenzene, 12% yield of **4Q**

Equation 2: trifluoromethylation reaction in presence of radical scavengers

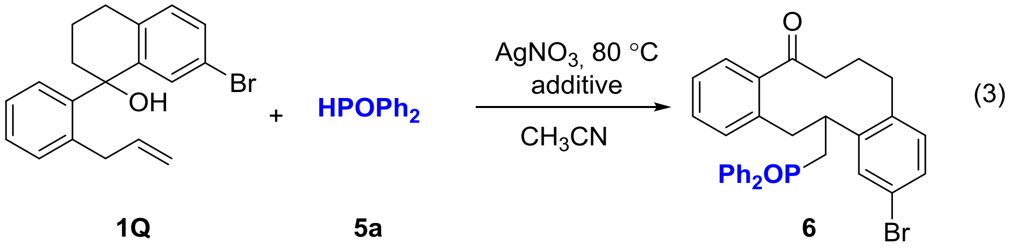

additive: TEMPO, 0% yield of **6**
1,4-dinitrobenzene, 17% yield of **6**

Equation 3: phosphonylation reaction in presence of radical scavengers

Supplementary Figure 2. Control experiment in the presence of radical scavengers.

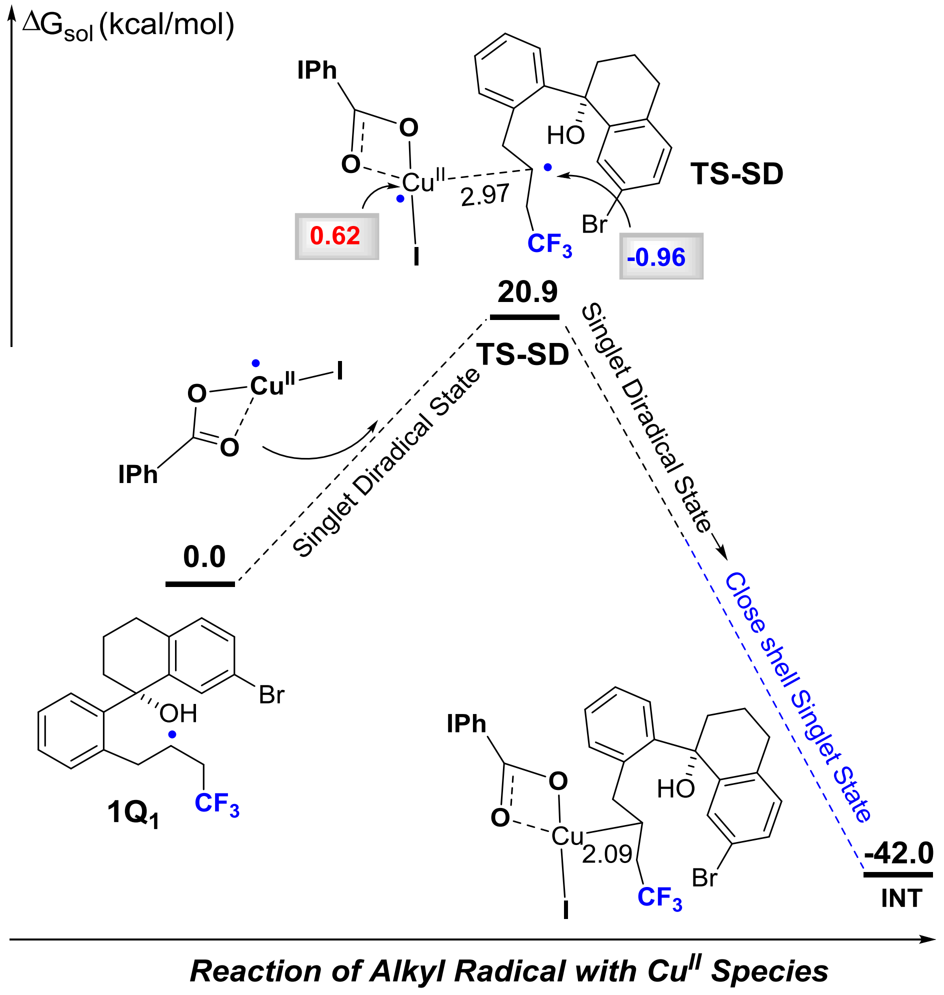

Supplementary Figure 3. The calculated relative free energies (ΔG_{sol}) in 1,4-dioxane with SMD model at the M11/6-31+G**/SDD/Aug-cc-PVTZ level are given in kcal/mol. The selected bond lengths are in Å.

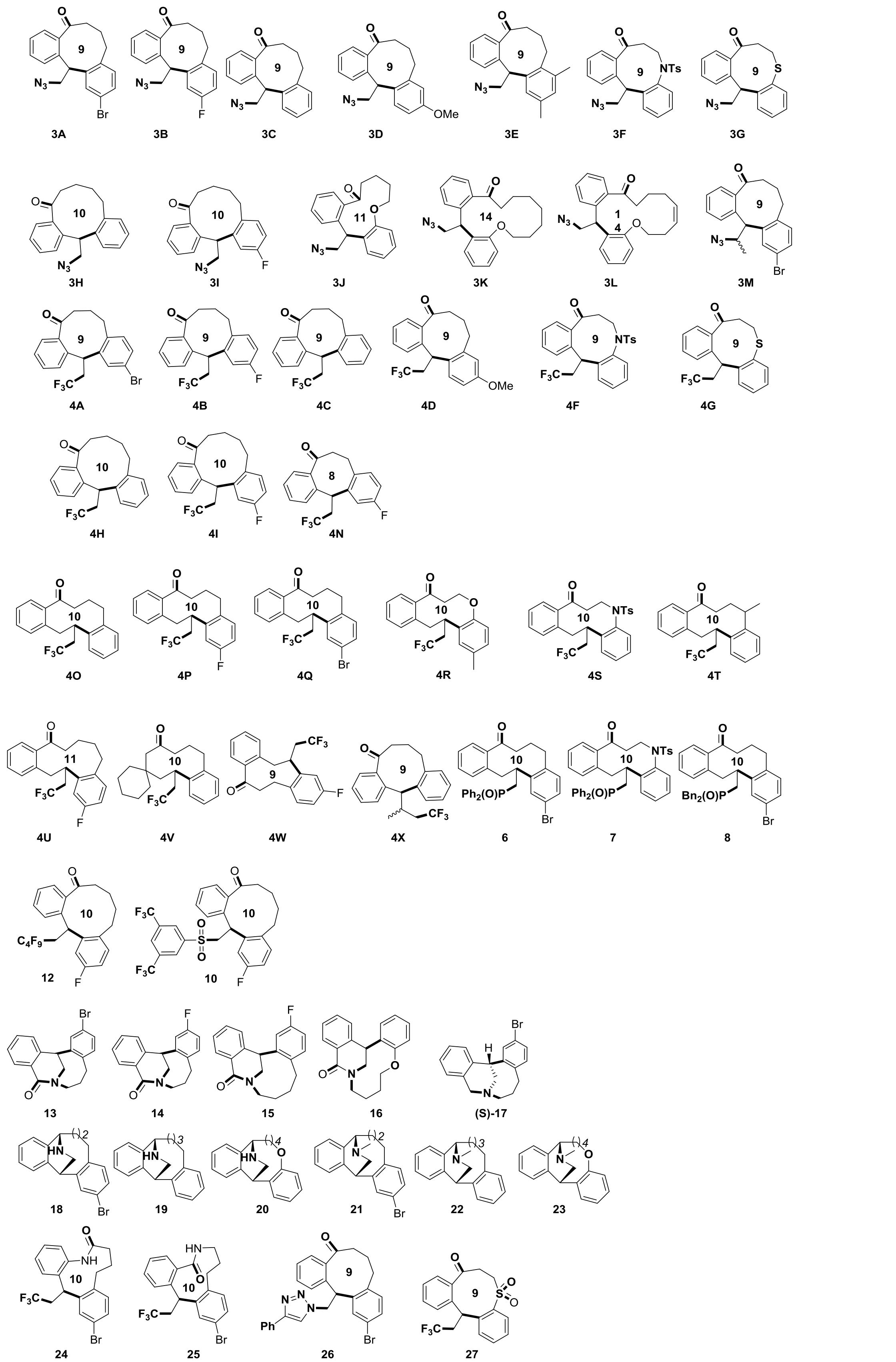

Supplementary Figure 4. Structures of 52 our prepared molecules employed in the principal component analysis.

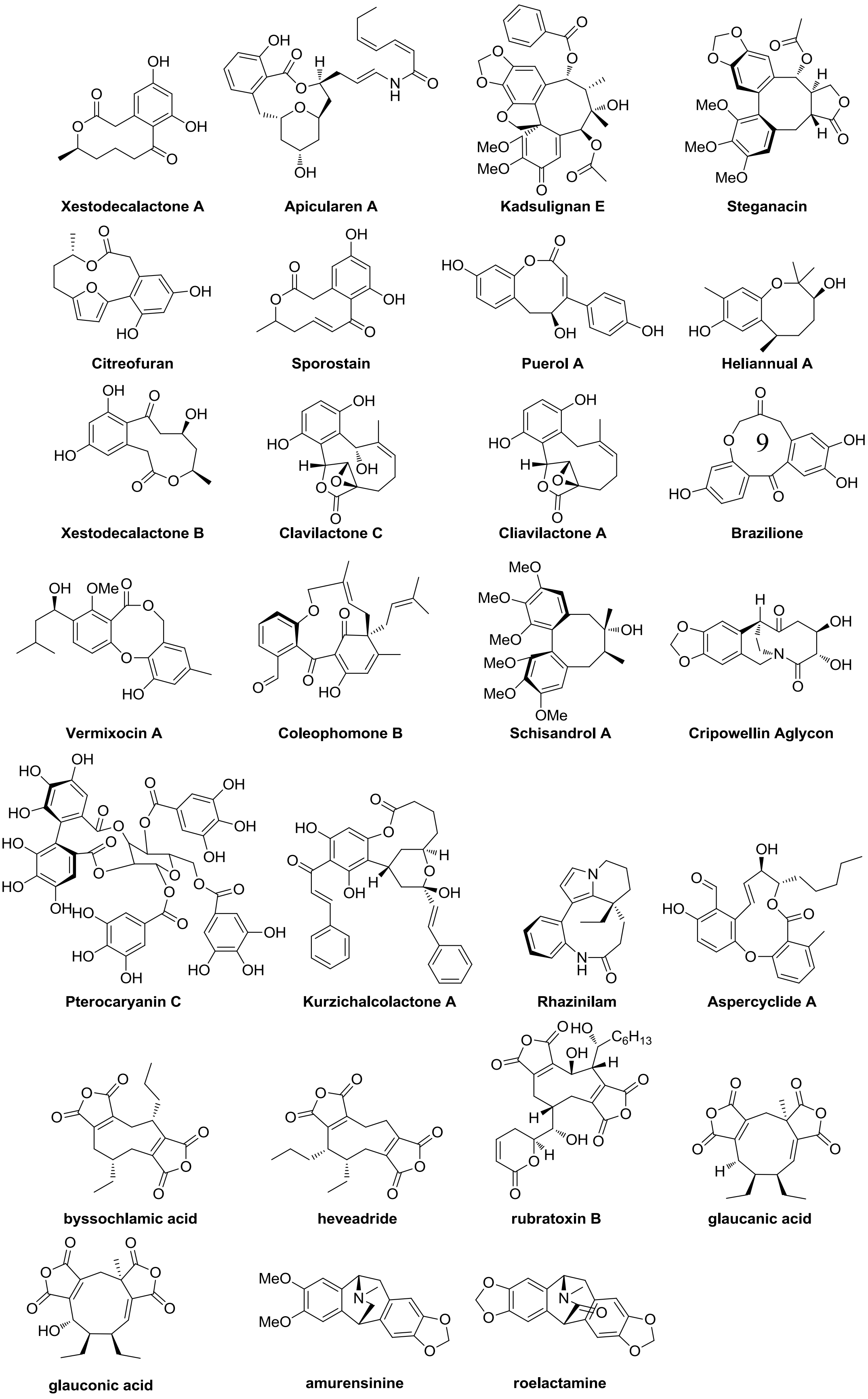

Supplementary Figure 5. Structures of 27 benzannulated medium-ring natural products employed in the principal component analysis.

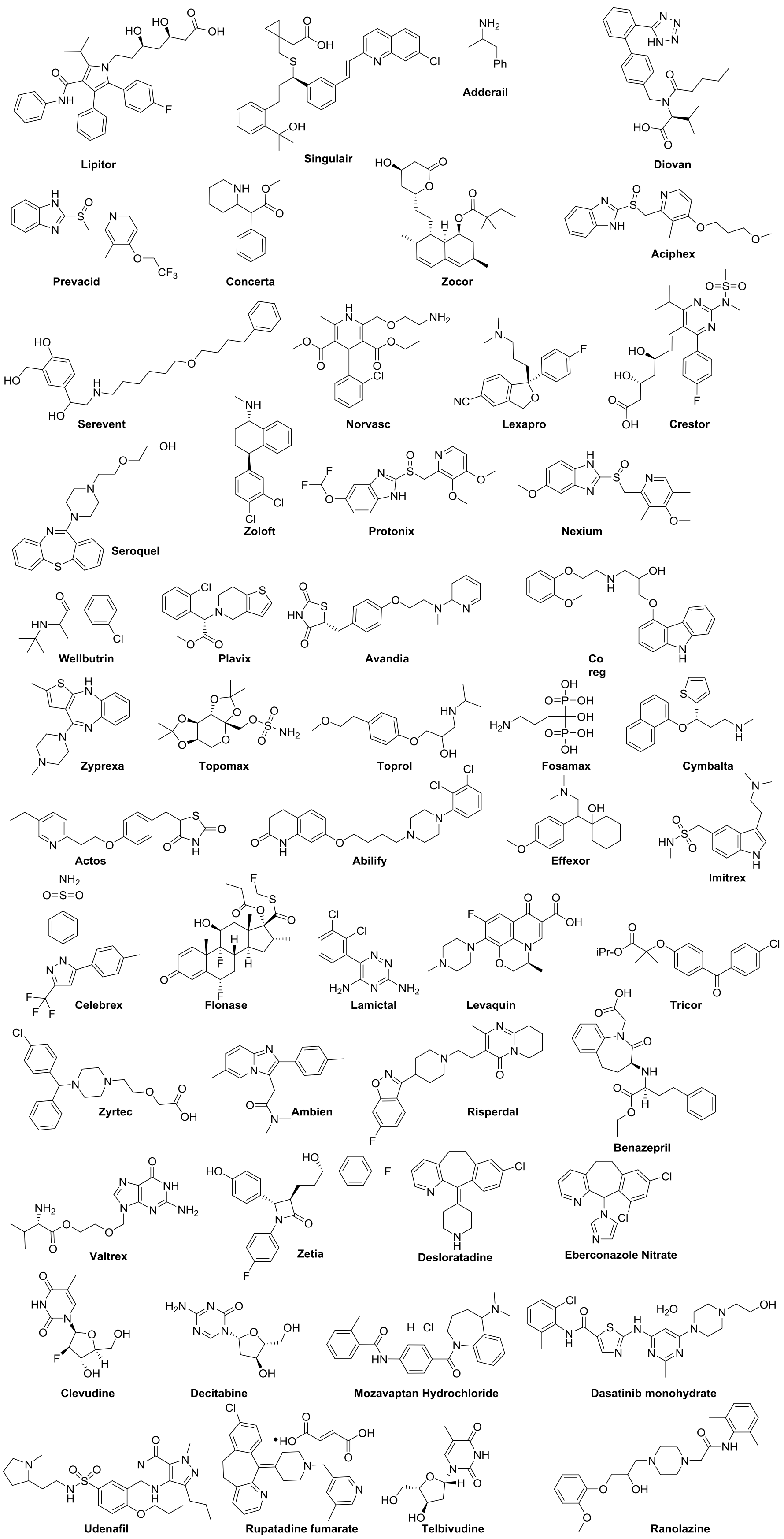

Supplementary Figure 6. Structures of 47 brand-name small molecule drugs of 2006 employed in the principal component analysis.

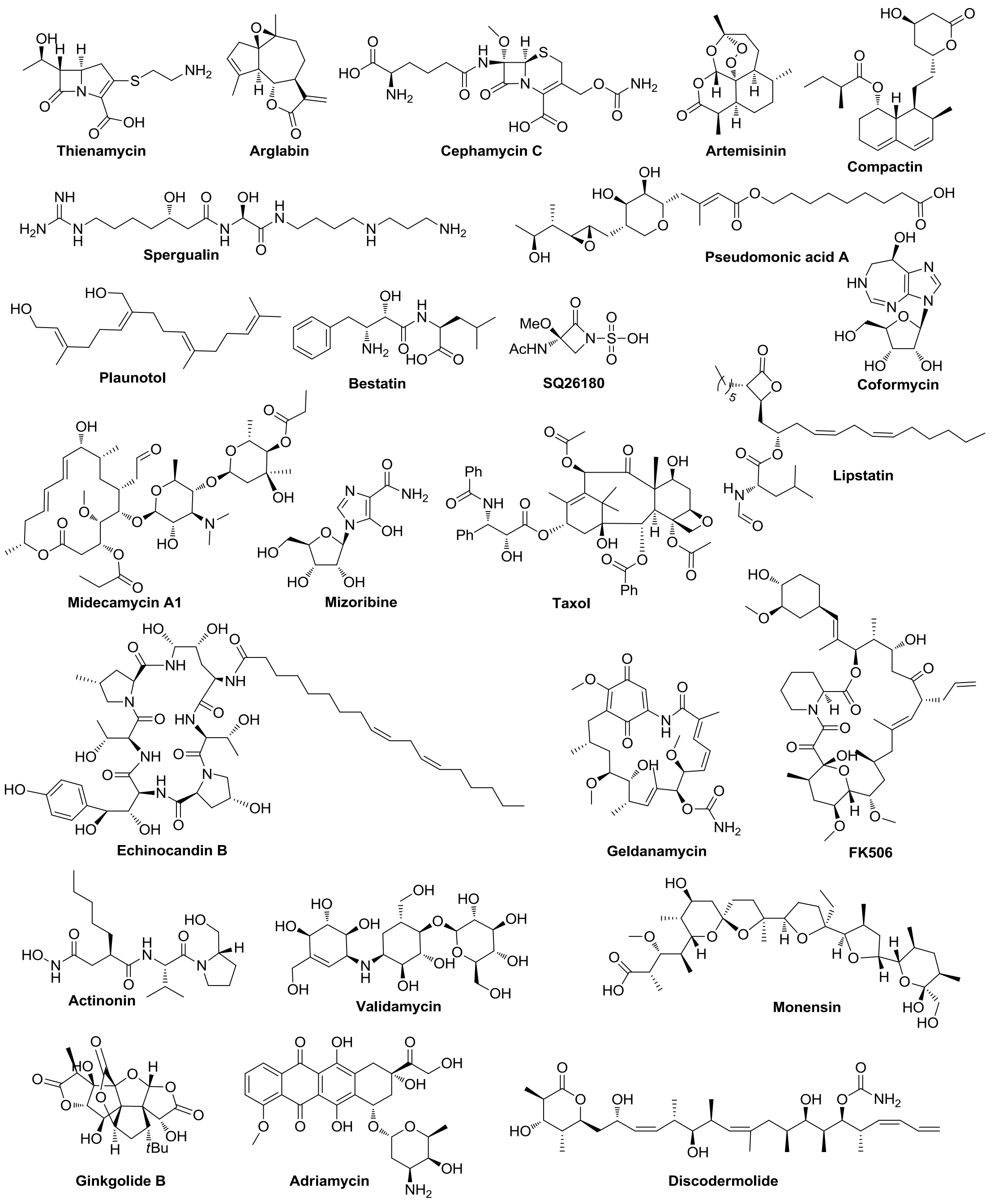

Supplementary Figure 7. Structures of 60 diverse natural products employed in the principal component analysis (*continued on next two pages*).

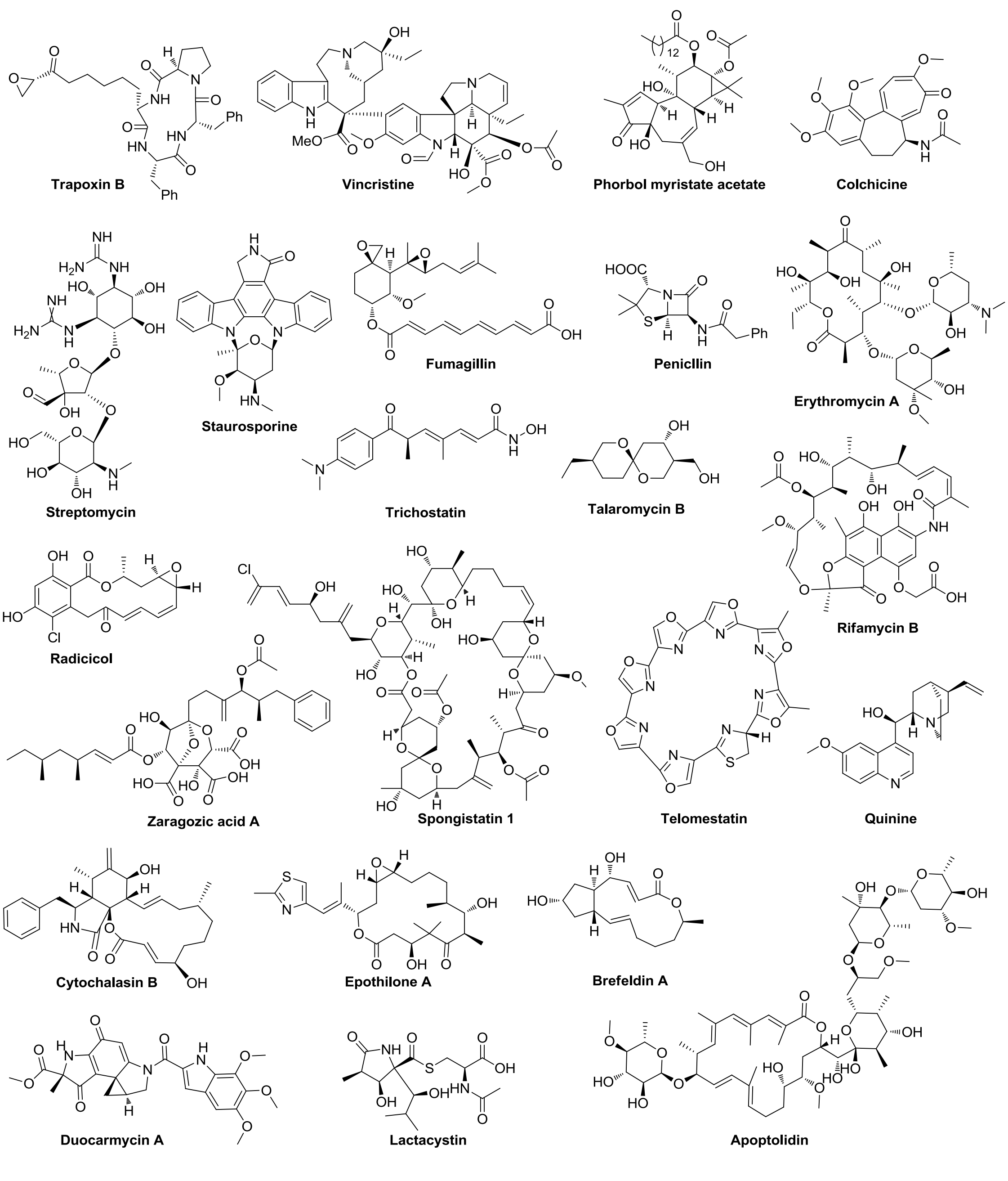

Supplementary Figure 7. Structures of 60 diverse natural products employed in the principal component analysis (*continued on next page*).

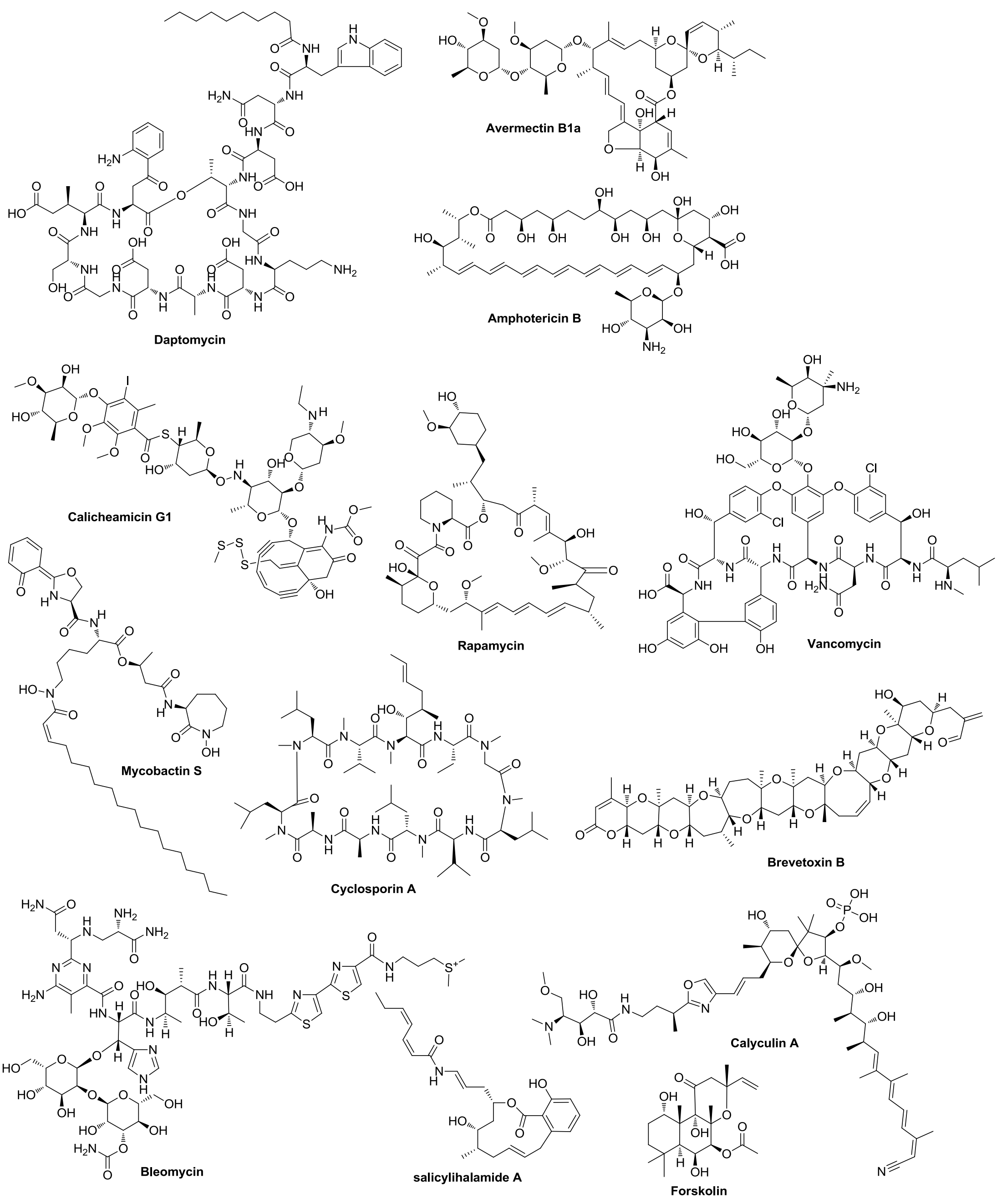

Supplementary Figure 7. Structures of 60 diverse natural products employed in the principal component analysis.

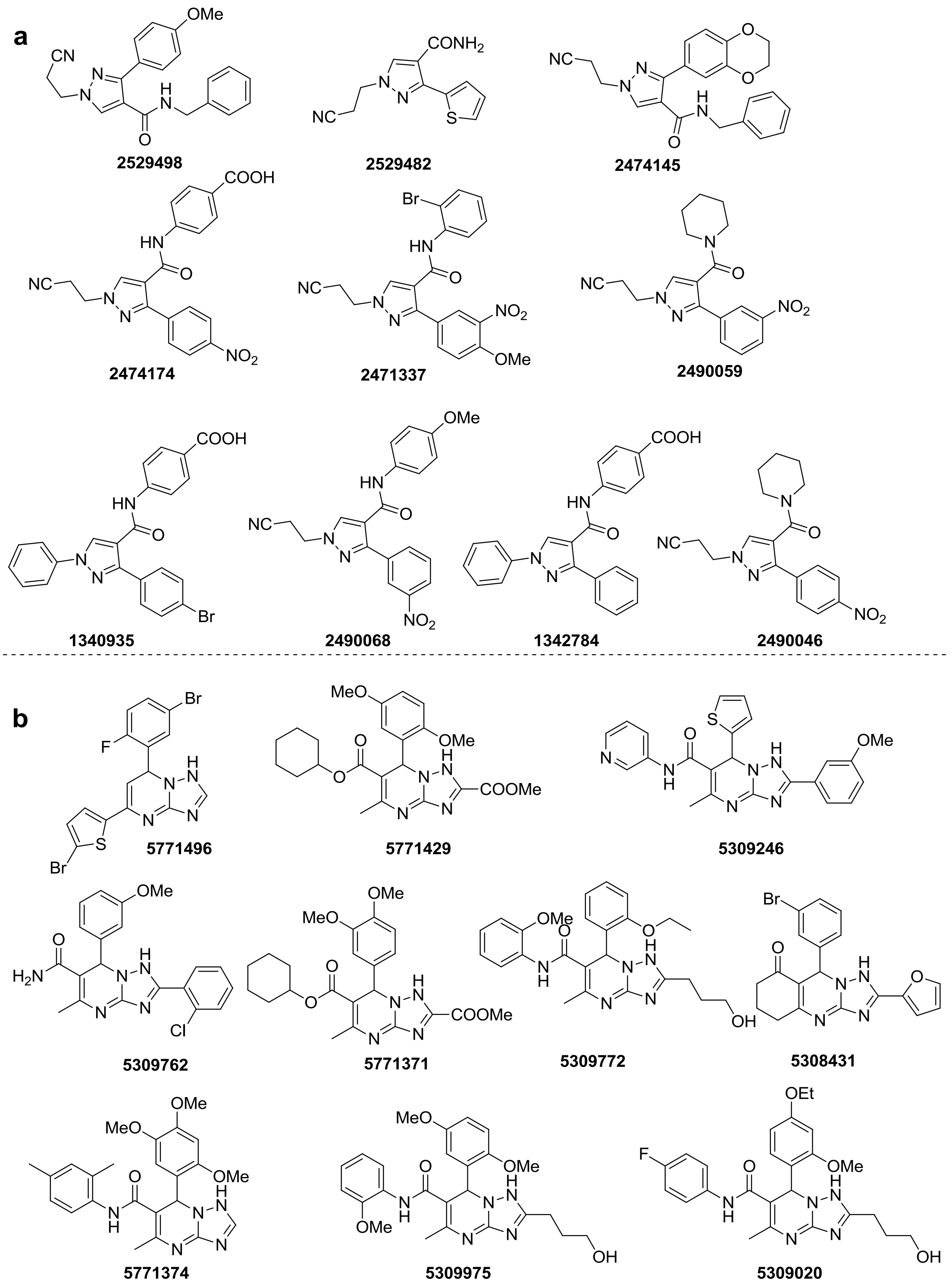

Supplementary Figure 8. Structures of 20 commercial drug-like library compounds in the Molecular Libraries Small Molecule Repository employed in the principal component analysis. (a) Pyrrazolecarboxamides from ChemBridge. (b) Dihydrotriazolopyrimidines from ChemDiv. All compounds were treated as racemates as applicable.

a

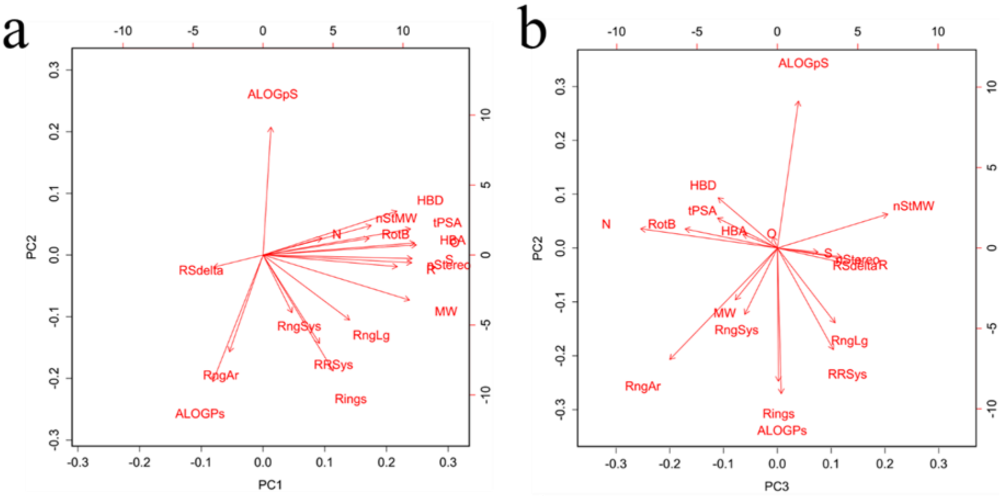

b

c

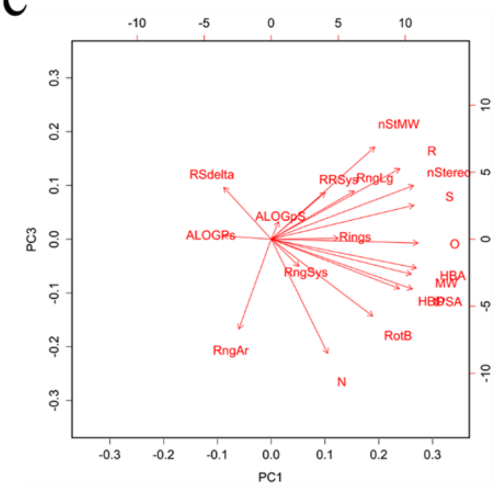

d

	PC1	PC2	PC3
**MW**	0.312786	-0.1622	-0.14342
**N**	0.126107	0.060342	-0.46675
**O**	0.32763	0.036089	-0.01573
**HBD**	0.285901	0.157575	-0.20223
**HBA**	0.324127	0.043304	-0.11811
**RotB**	0.226638	0.059908	-0.31501
**tPSA**	0.315274	0.094014	-0.20413
**ALOGPs**	-0.10749	-0.45574	0.013319
**ALOGpS**	0.016805	0.459938	0.071945
**nStereo**	0.317713	-0.02785	0.219458
**R**	0.286933	-0.04196	0.288428
**S**	0.318495	-0.0128	0.138788
**nStMW**	0.23096	0.106205	0.376986
**RSdelta**	-0.10571	-0.04381	0.212006
**Rings**	0.149984	-0.41717	0.003718
**RngAr**	-0.072	-0.34872	-0.36718
**RngSys**	0.06191	-0.2076	-0.11122
**RngLg**	0.185116	-0.23395	0.196919
**RRSys**	0.120489	-0.31782	0.191227

Supplementary Figure 9. Biplots and component loadings for principal component analysis (PCA). The biplots for (a) PC1 vs. PC2, (b) PC3 vs. PC2, and (c) PC1 vs. PC3, and (d) component loadings of the 19 original structural and physicochemical descriptors on the first three principal components indicate the influence of each structural and physicochemical descriptor upon the positioning of compounds in the PCA plots (Figure S9). The four most influential parameters on each principal component are highlighted (yellow).

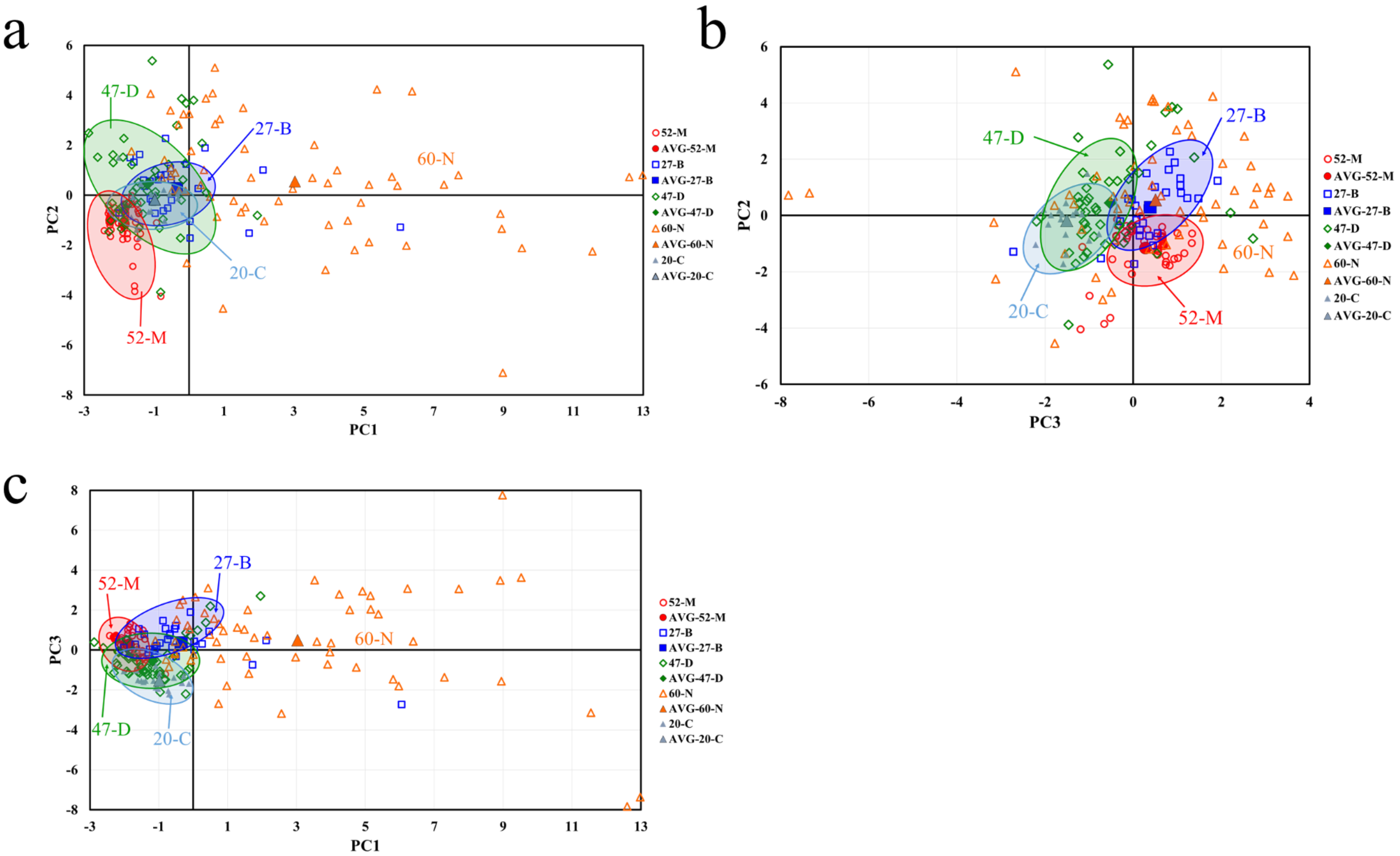
a
PC1
PC2
47-D
27-B
60-N
20-C
52-M
52-M
AVG-52-M
27-B
AVG-27-B
47-D
AVG-47-D
60-N
AVG-60-N
20-C
AVG-20-C
b
PC3
PC2
47-D
27-B
60-N
20-C
52-M
c
PC1
PC3
52-M
27-B
60-N
47-D
20-C

Supplementary Figure 10. Cheminformatic analysis. Principal component analysis (PCA) of 52 our prepared compounds (labeled as 52-M), 27 benzannulated medium-ring natural products (labeled as 27-B), 47 brand-name small molecule drugs of 2006 (labeled as 47-D), 60 diverse natural products (labeled as 60-N), and 20 commercial available drug-like library compounds in the Molecular Libraries Small Molecule Repository (labeled as 20-C) based on 19 structural and physicochemical parameters. The hypothetical average structure for each series (-AVG) is also shown. (a) PCA plot of PC1 vs. PC2. (b) PCA plot of PC3 vs. PC2. (c) PCA plot of PC1 vs. PC3.

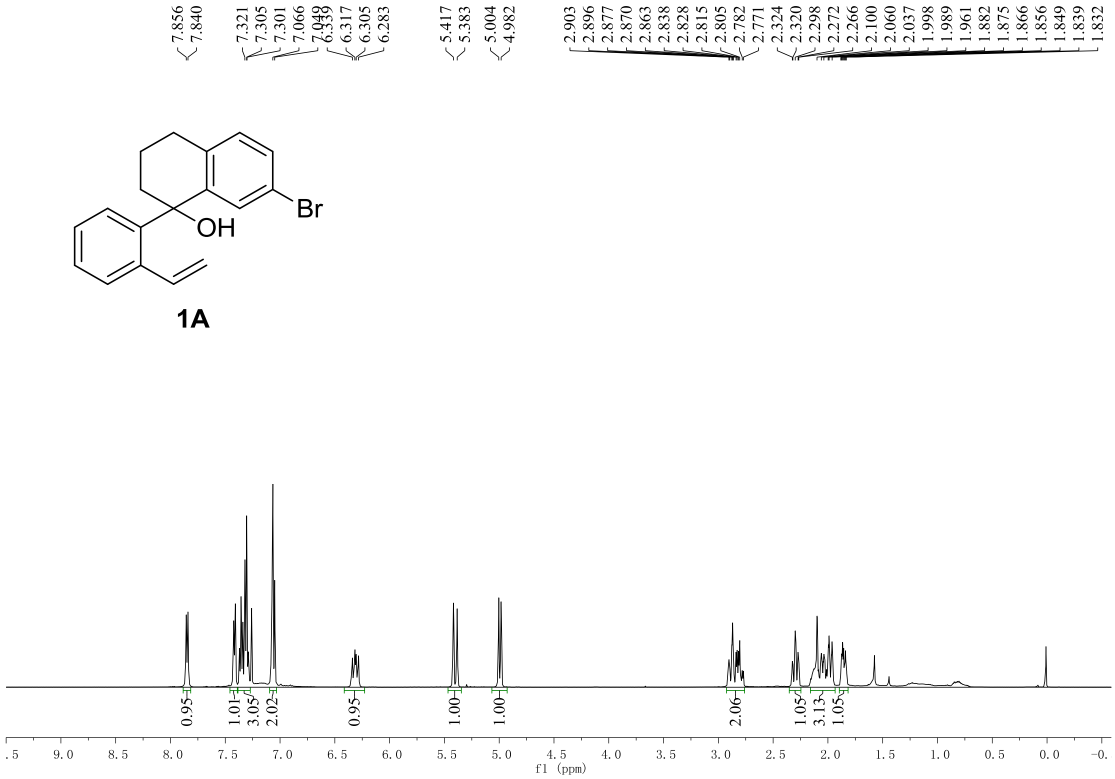

Supplementary Figure 11. ^{1}H NMR of **1A**

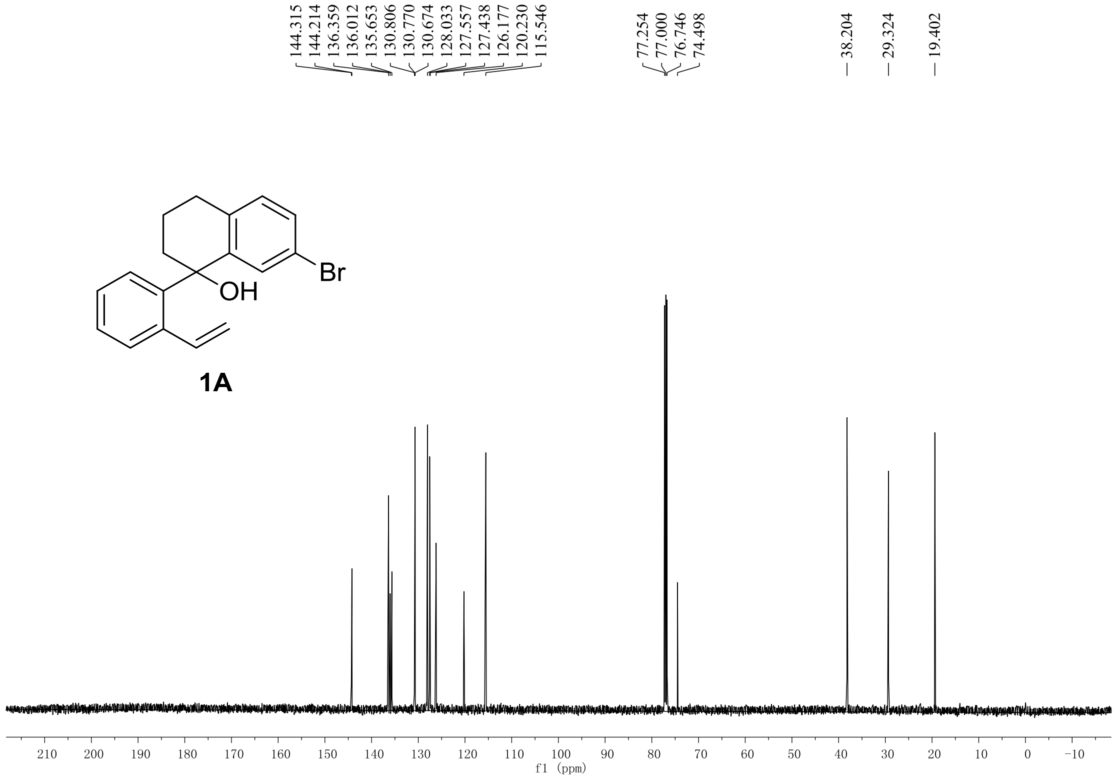

Supplementary Figure 12. ^{13}C NMR of **1A**

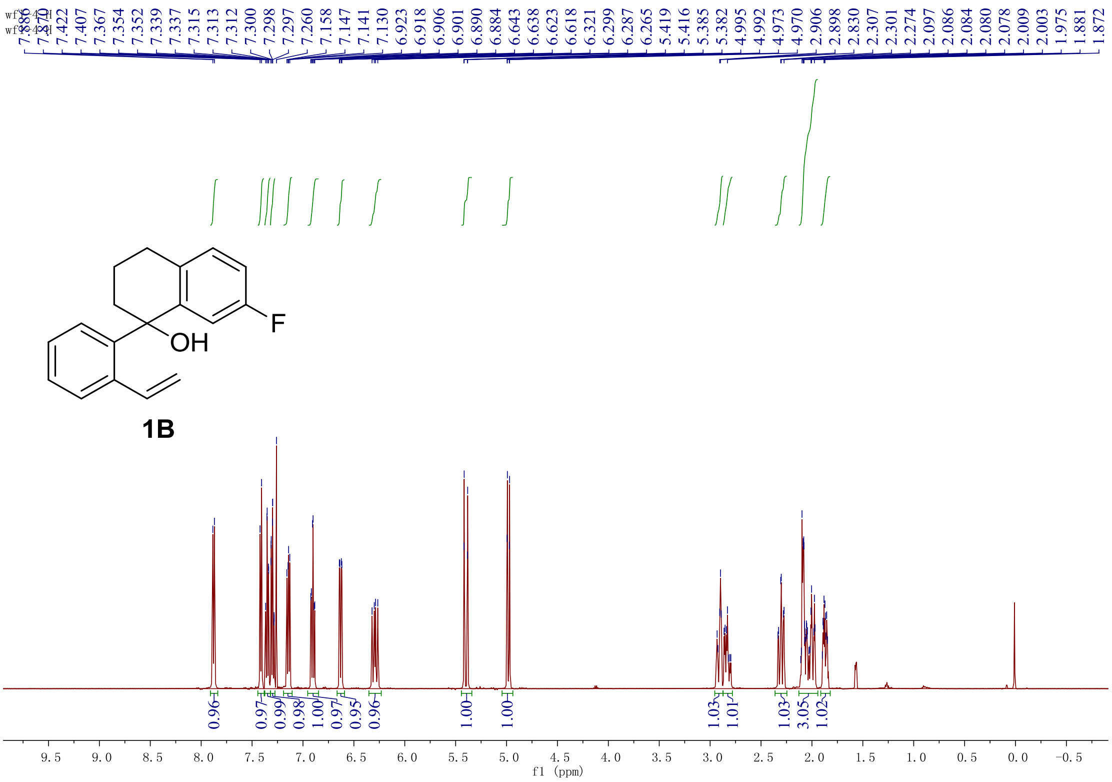

Supplementary Figure 13. ^{1}H NMR of **1B**

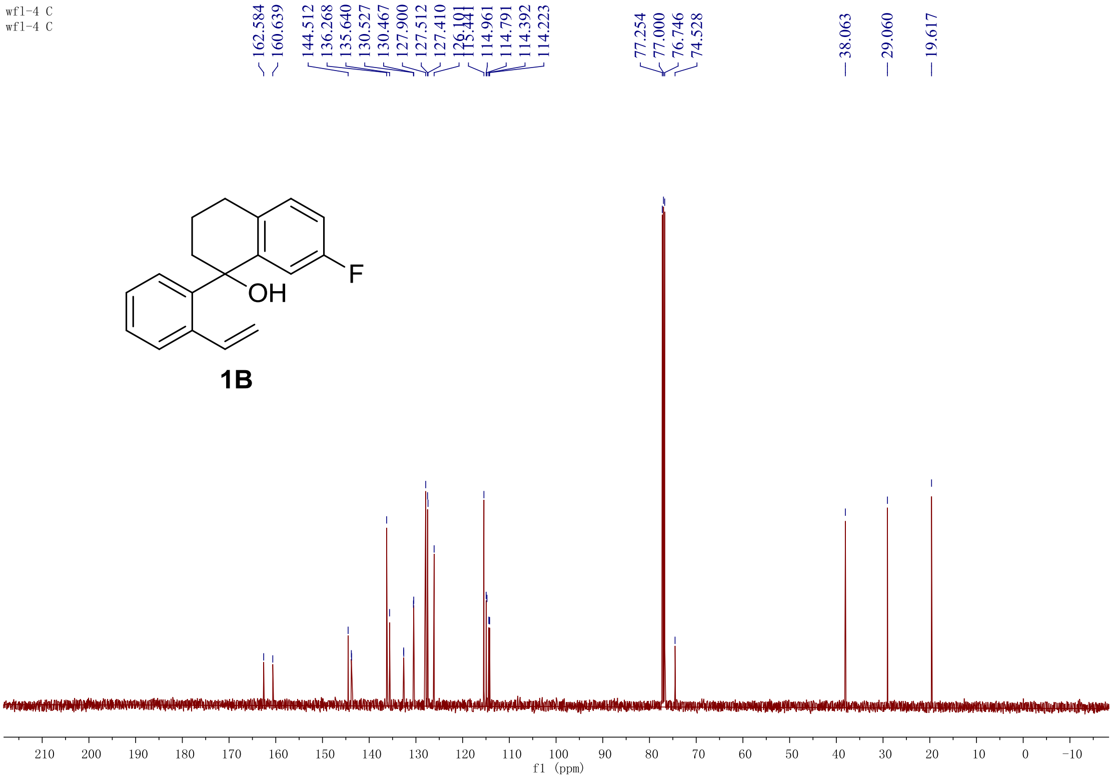

Supplementary Figure 14. ^{13}C NMR of **1B**

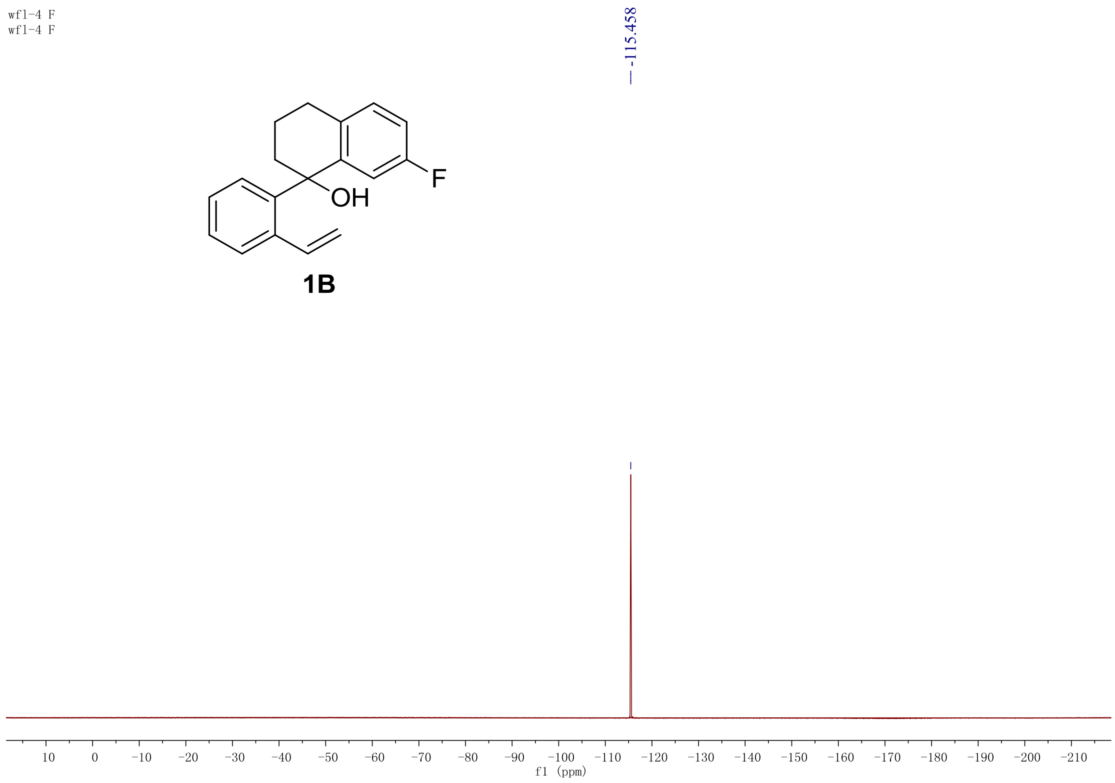

Supplementary Figure 15. ^{19}F NMR of **1B**

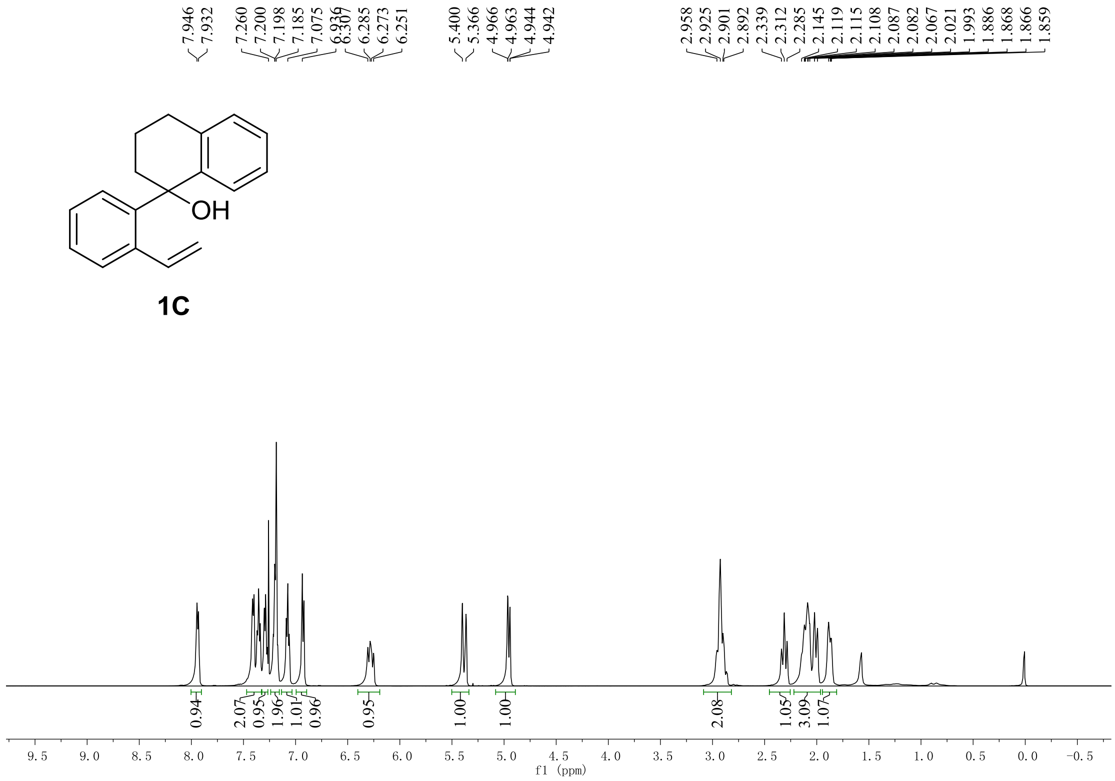

Supplementary Figure 16. ^{1}H NMR of **1C**

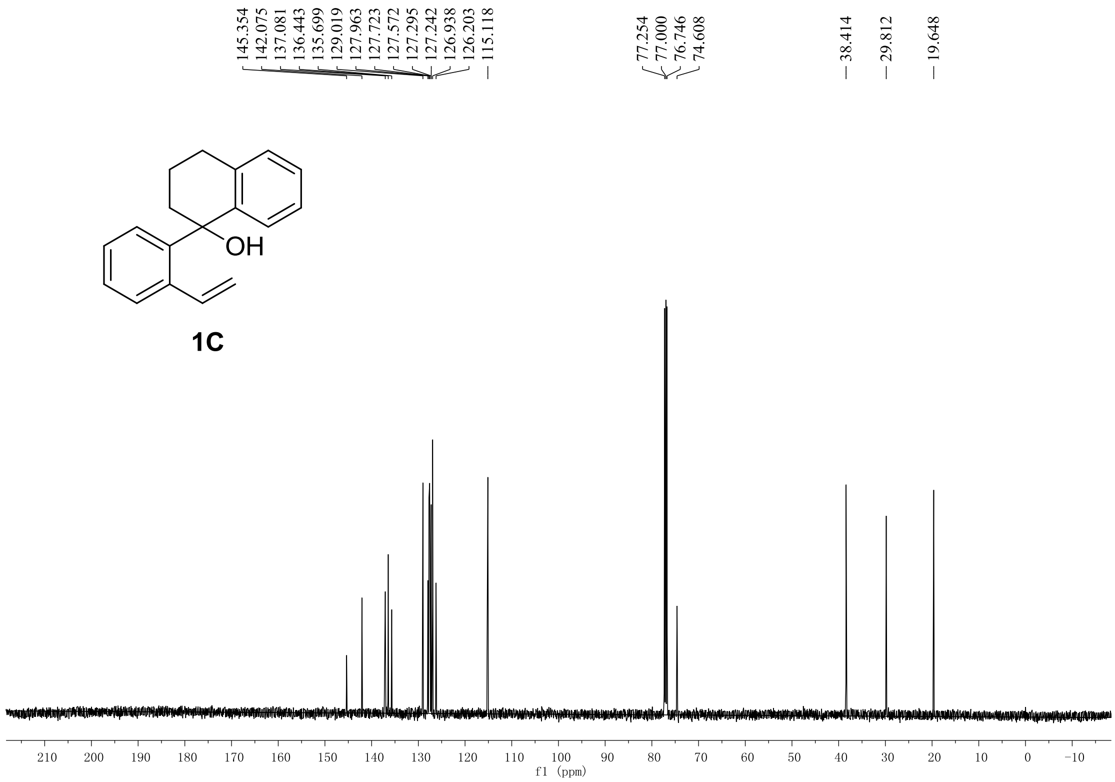

Supplementary Figure 17. ^{13}C NMR of **1C**

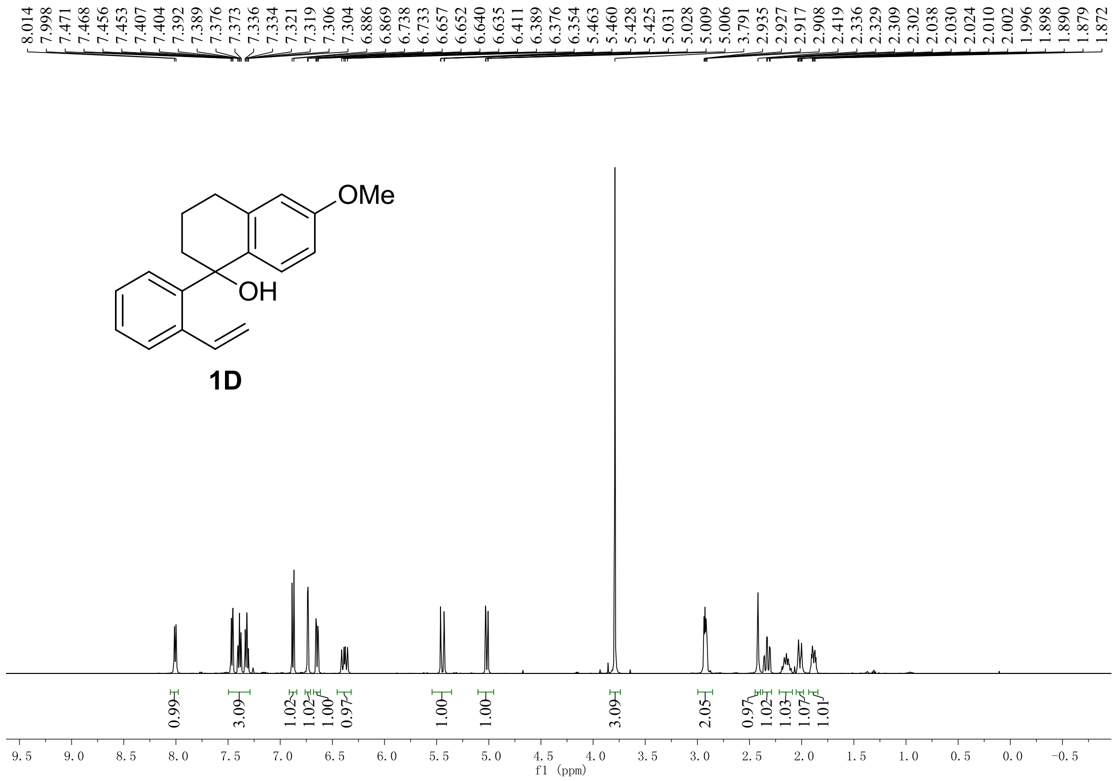

Supplementary Figure 18. ^{1}H NMR of **1D**

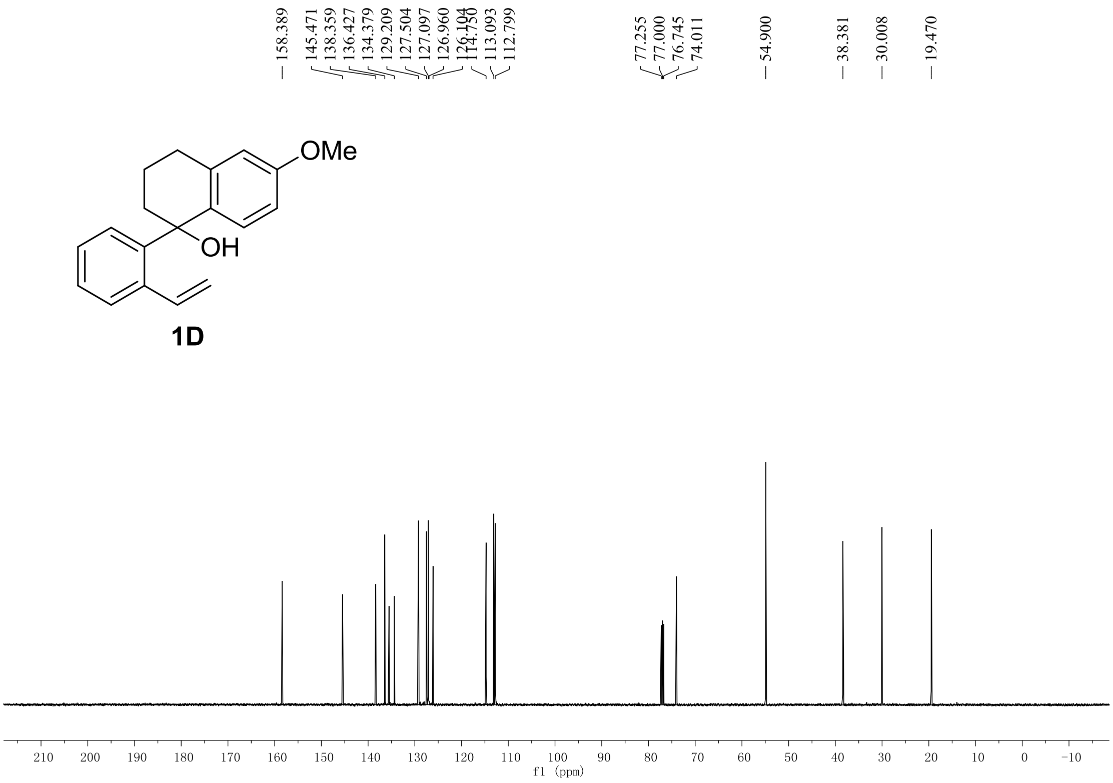

Supplementary Figure 19. ^{13}C NMR of **1D**

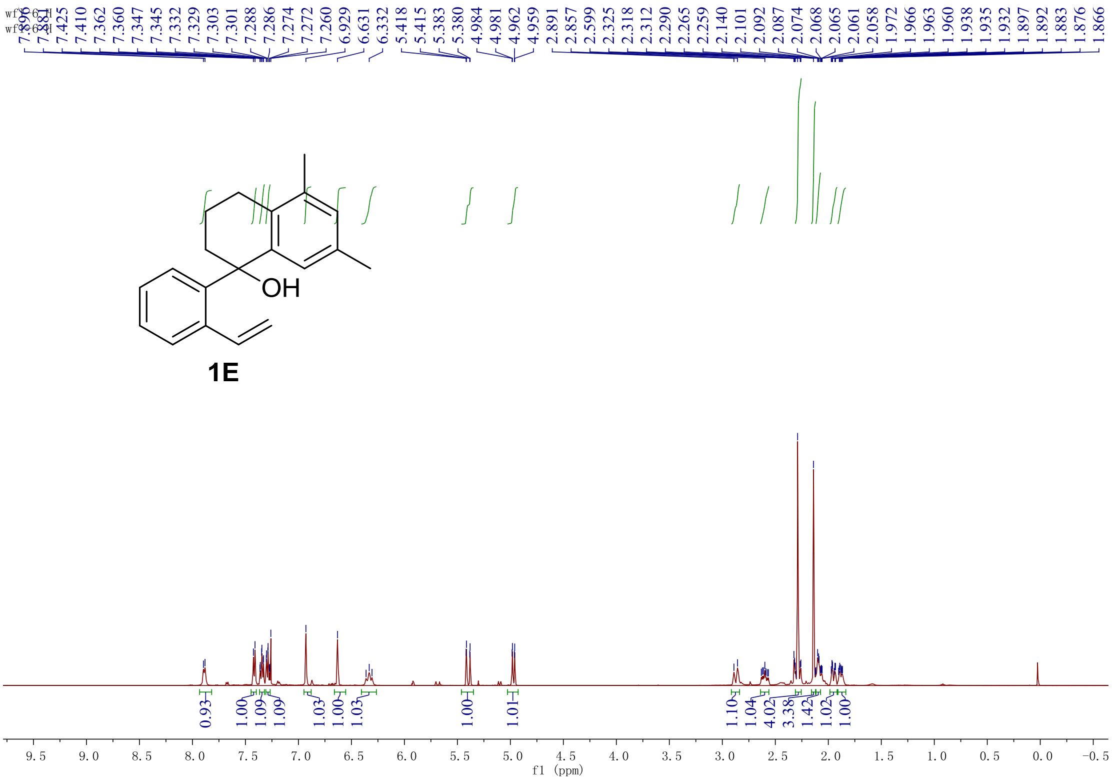

Supplementary Figure 20. ^{1}H NMR of **1E**

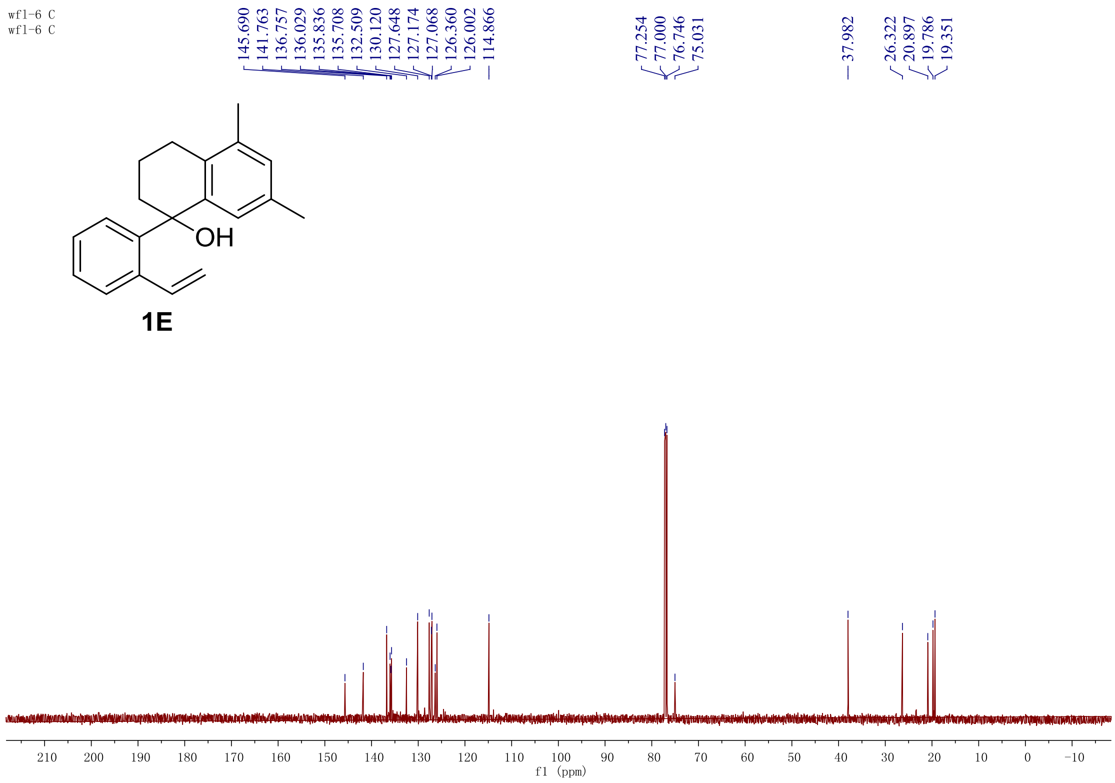

Supplementary Figure 21. ^{13}C NMR of **1E**

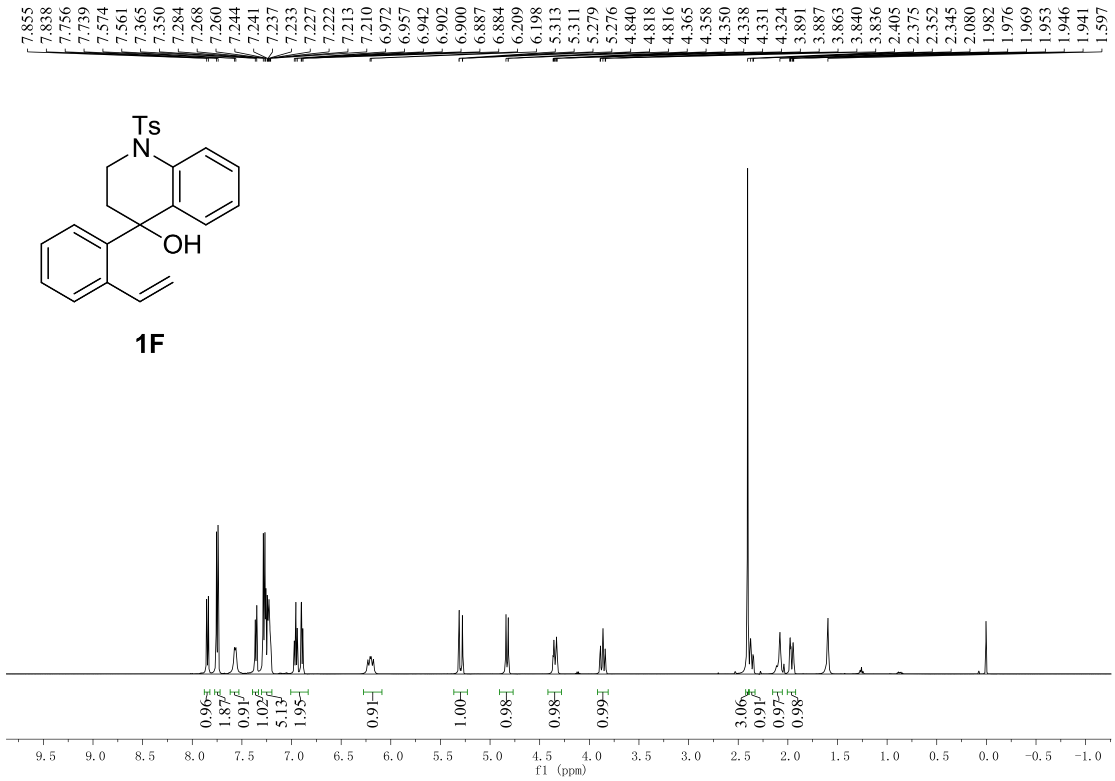

Supplementary Figure 22. ^{1}H NMR of **1F**

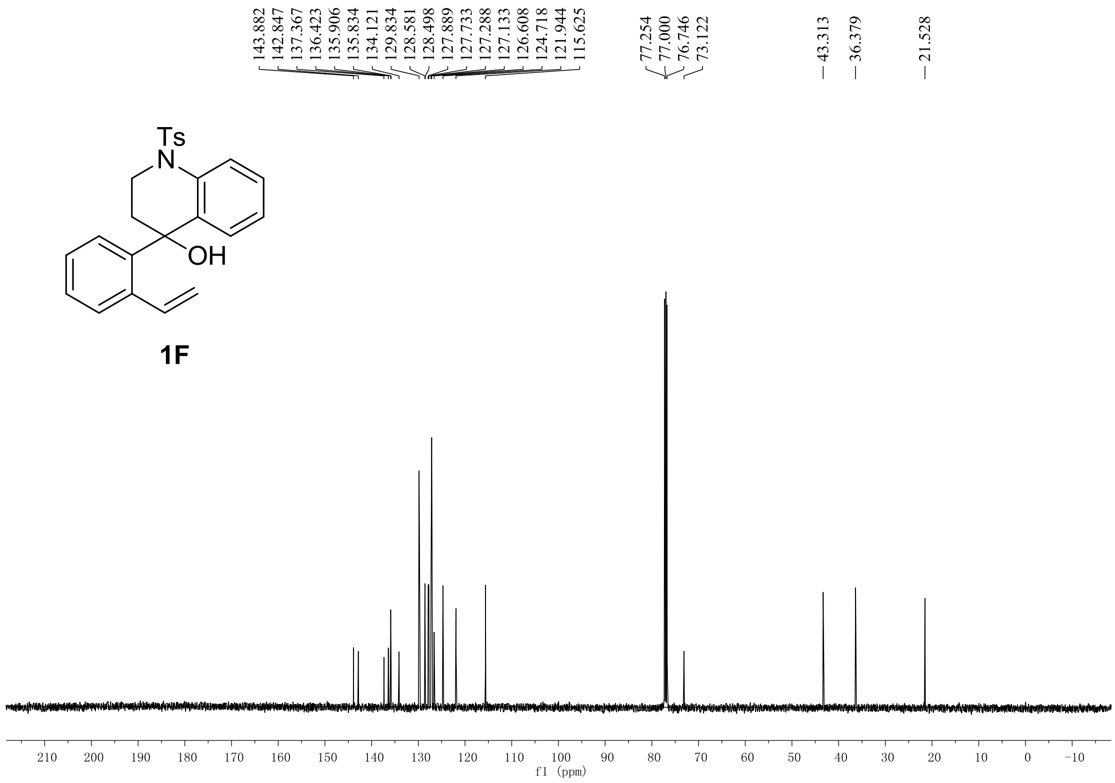

Supplementary Figure 23. ^{13}C NMR of **1F**

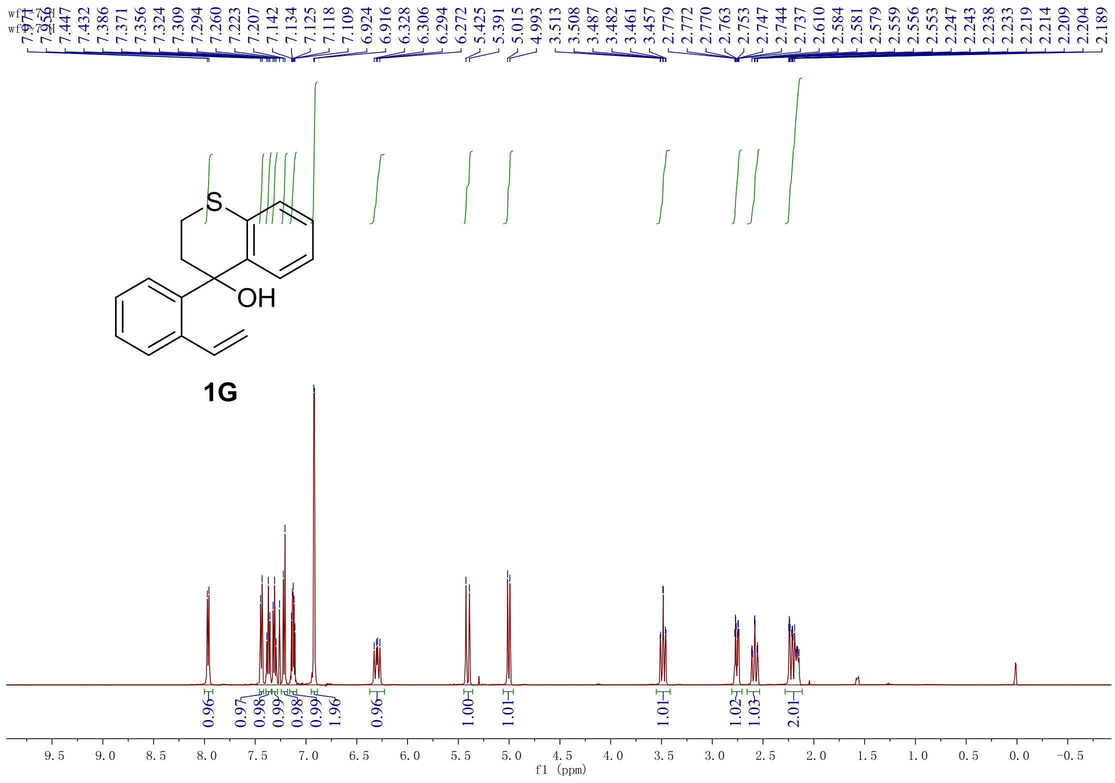

Supplementary Figure 24. ^{1}H NMR of **1G**

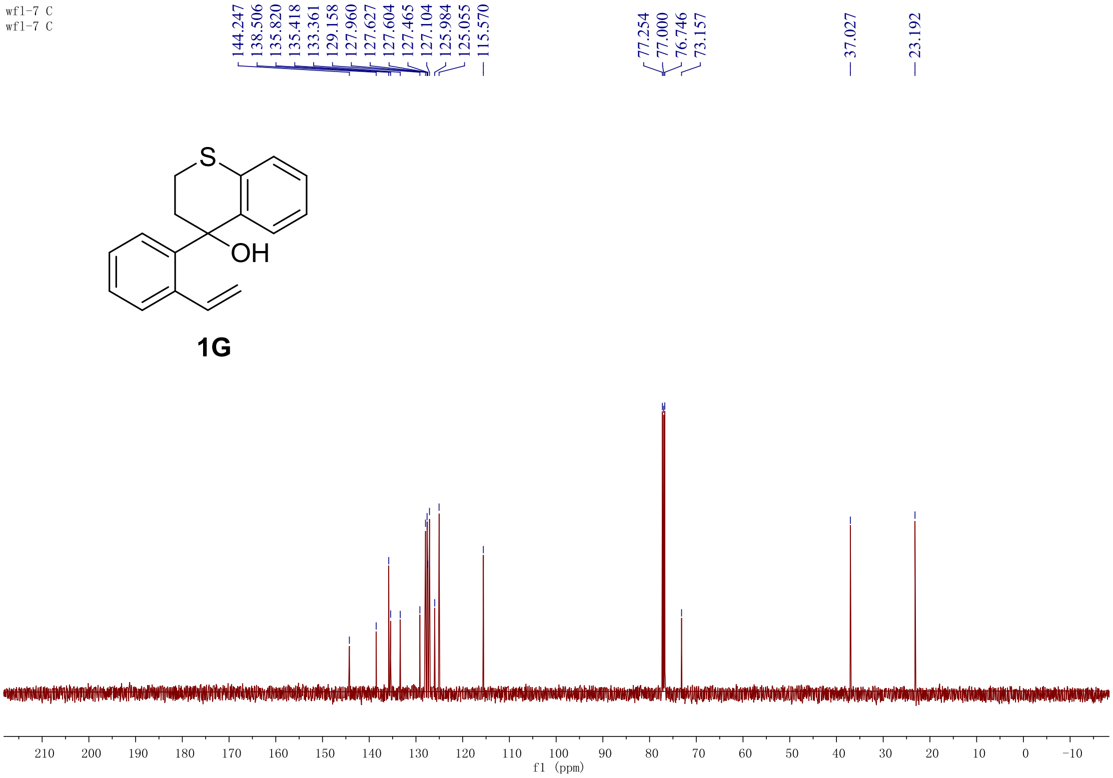

Supplementary Figure 25. ^{13}C NMR of **1G**

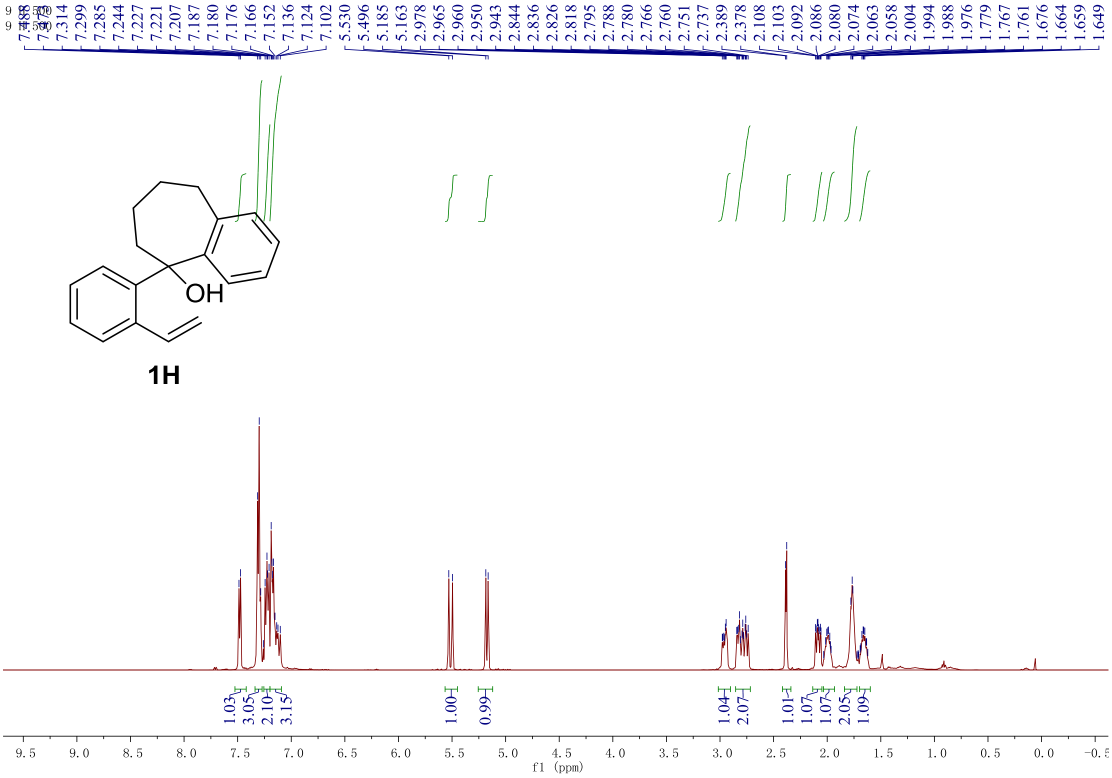

Supplementary Figure 26. ^{1}H NMR of **1H**

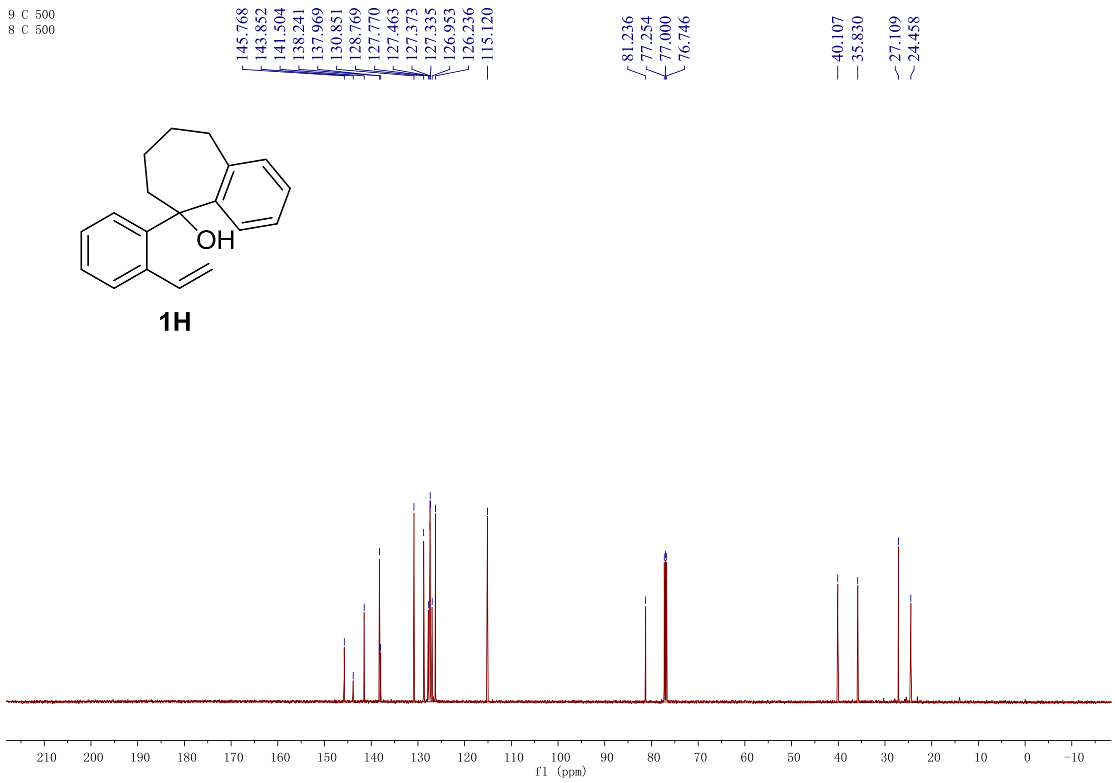

Supplementary Figure 27. ^{13}C NMR of **1H**

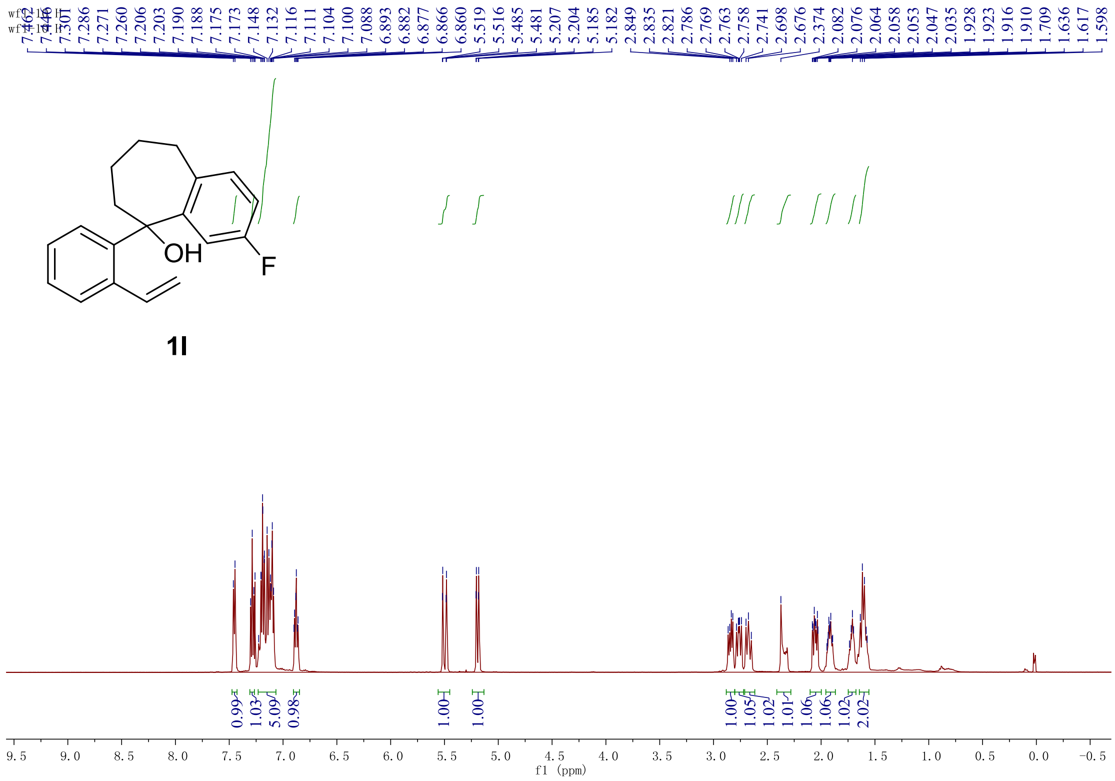

Supplementary Figure 28. ^{1}H NMR of **1I**

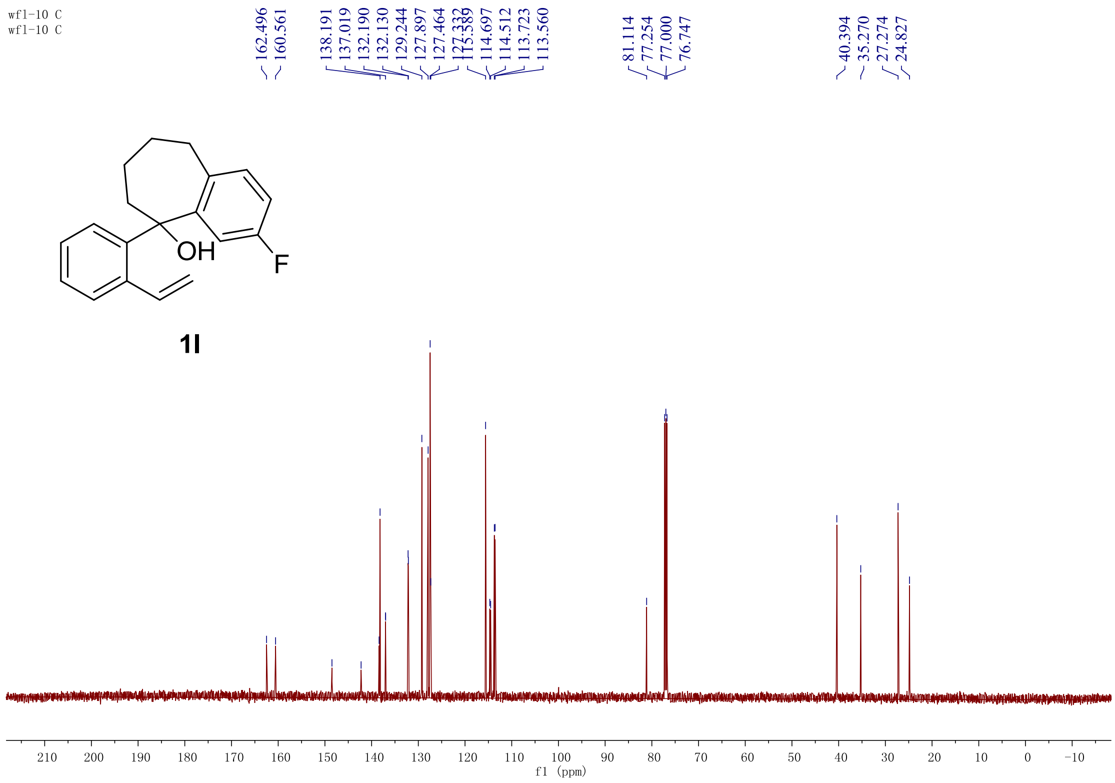

Supplementary Figure 29. ^{13}C NMR of **1l**

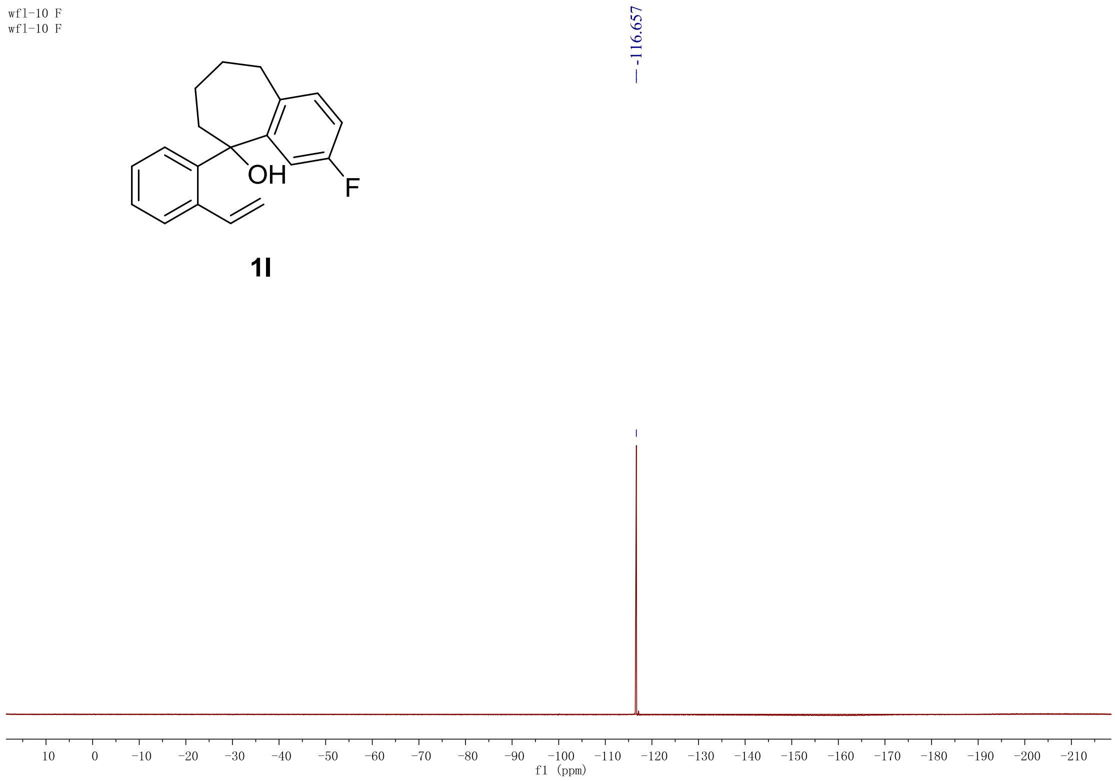

Supplementary Figure 30. ^{19}F NMR of **1l**

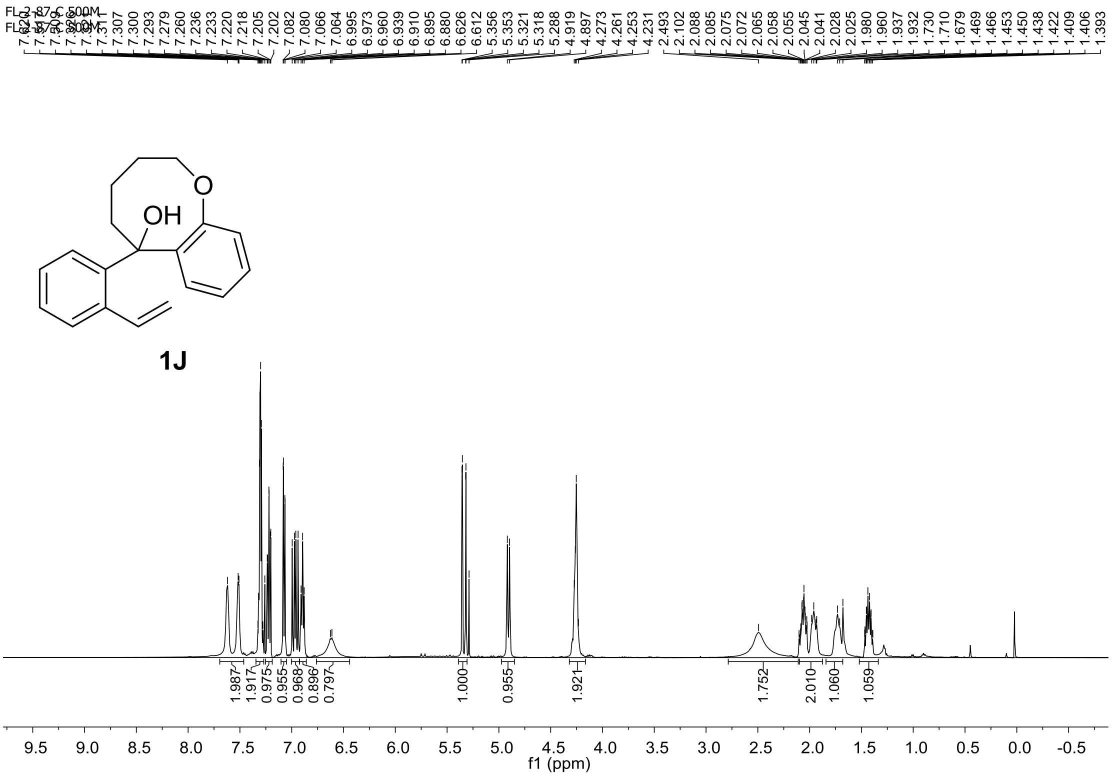

Supplementary Figure 31. ^{1}H NMR of **1J**

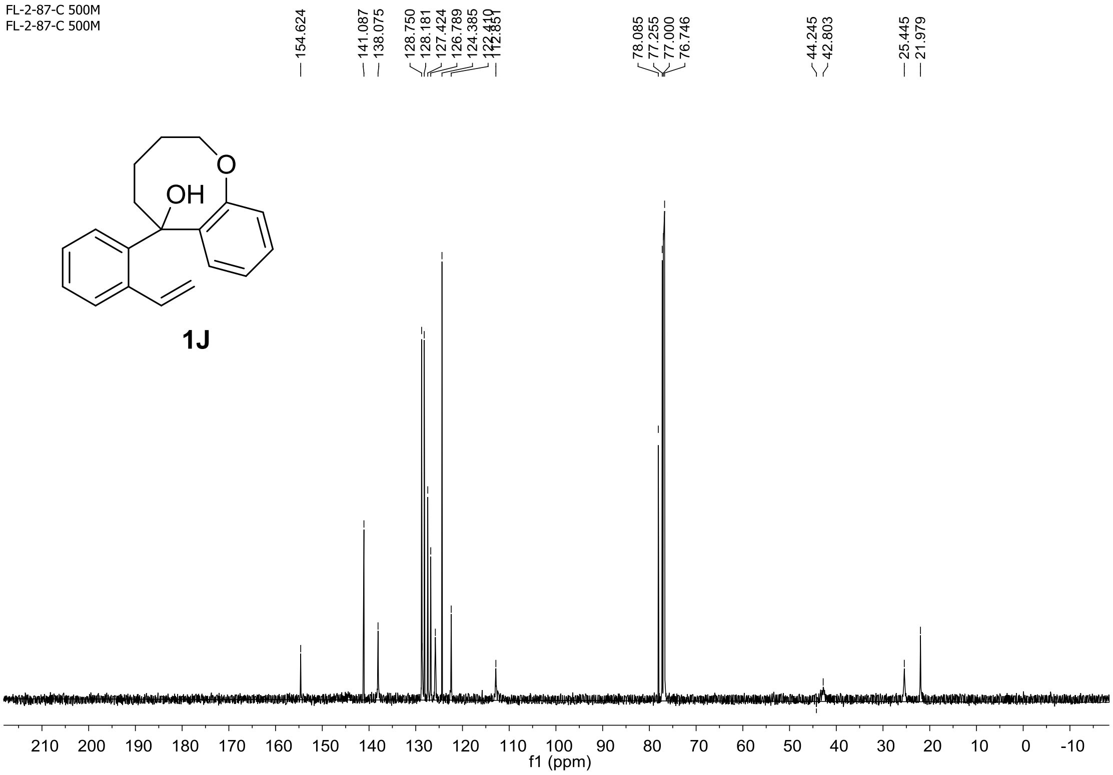

Supplementary Figure 32. ^{13}C NMR of **1J**

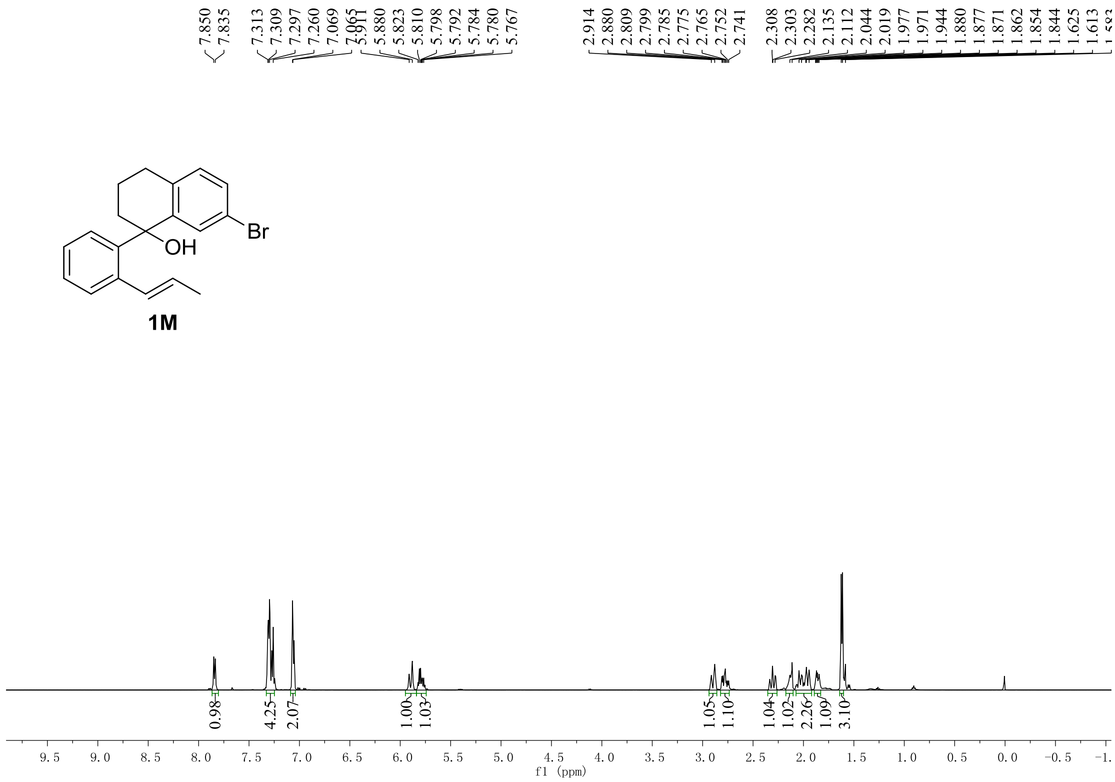

Supplementary Figure 33. ^{1}H NMR of **1M**

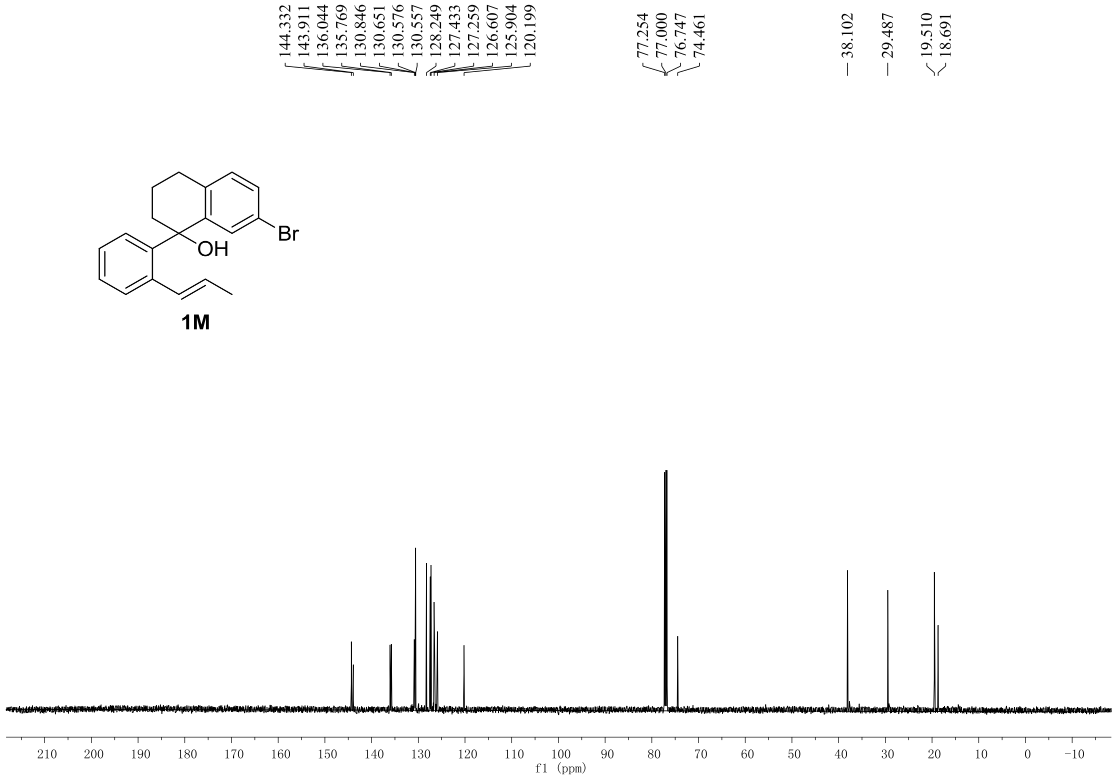

Supplementary Figure 34. ^{13}C NMR of **1M**

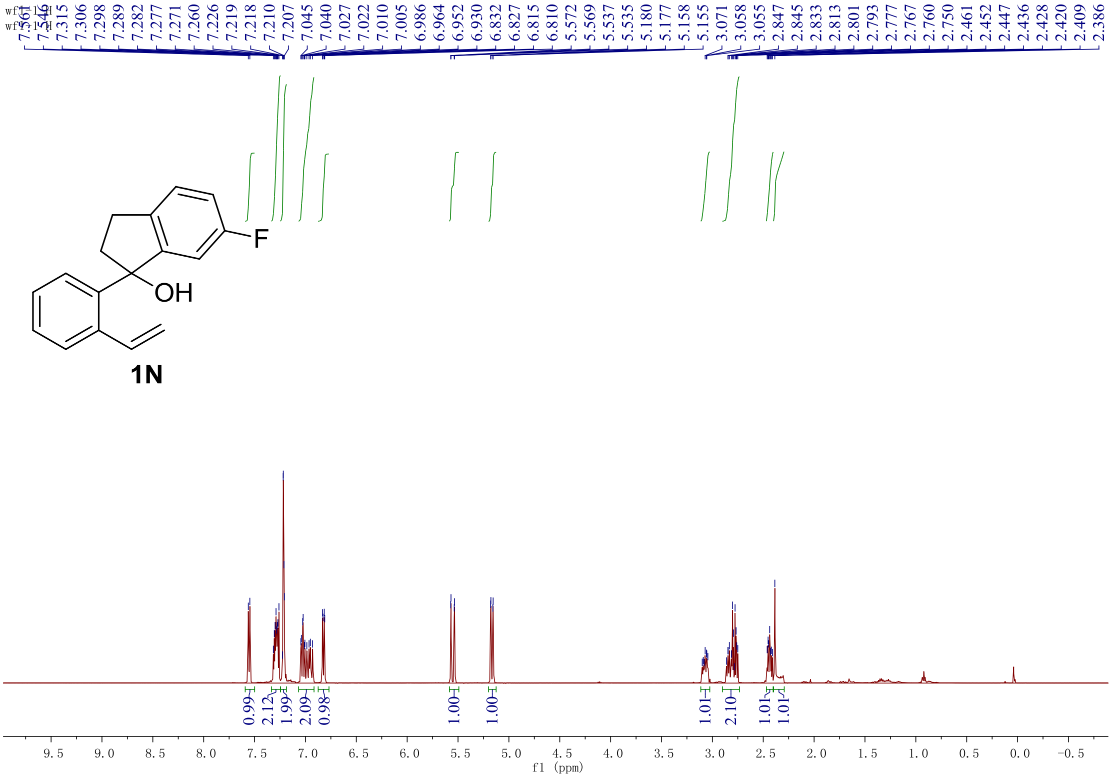

Supplementary Figure 35. ^{1}H NMR of **1N**

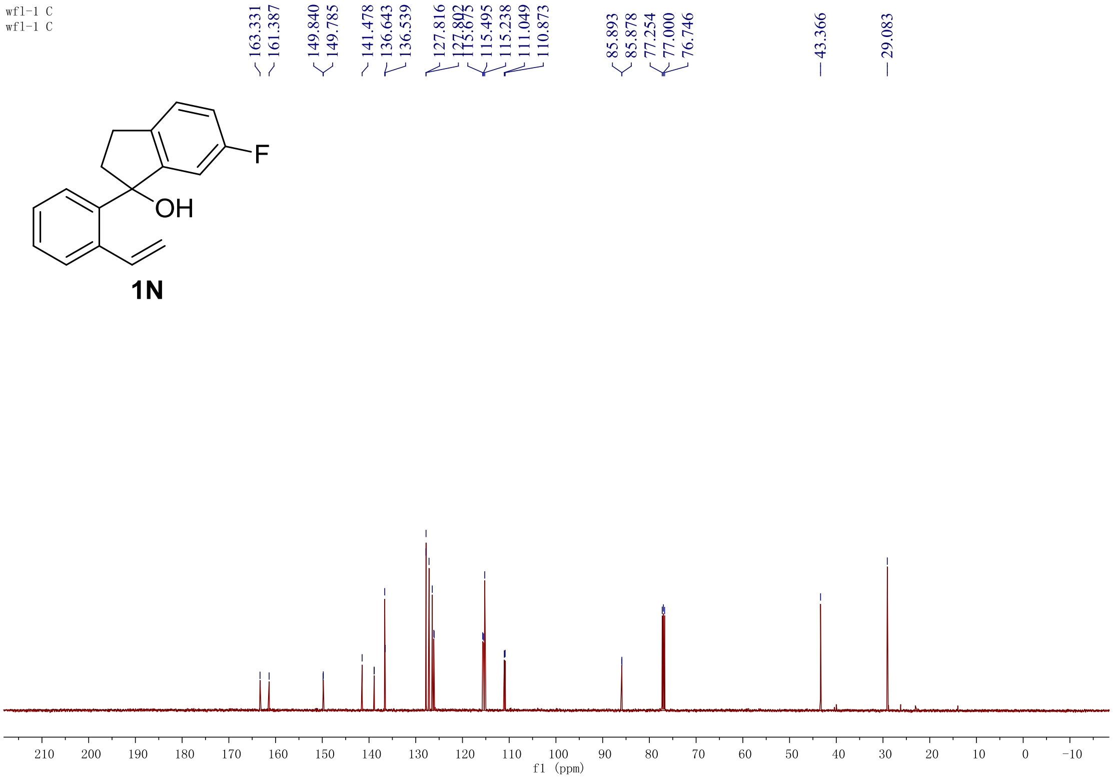

Supplementary Figure 36. ^{13}C NMR of **1N**

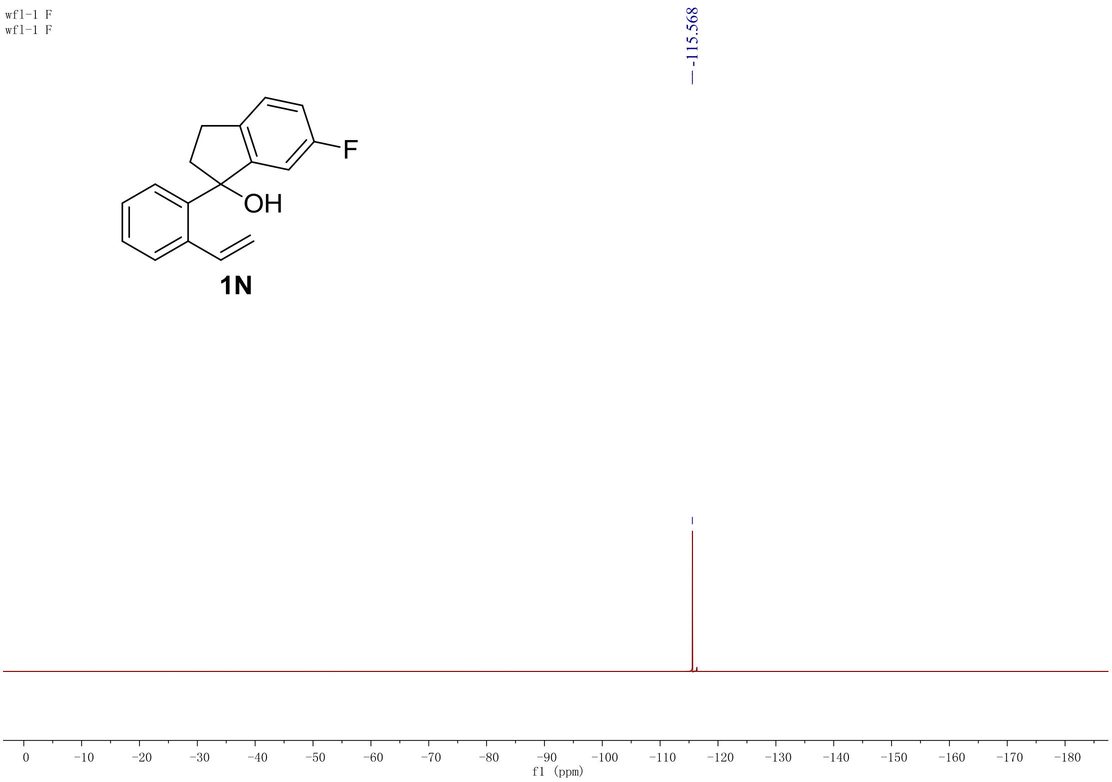

Supplementary Figure 37. ^{19}F NMR of **1N**

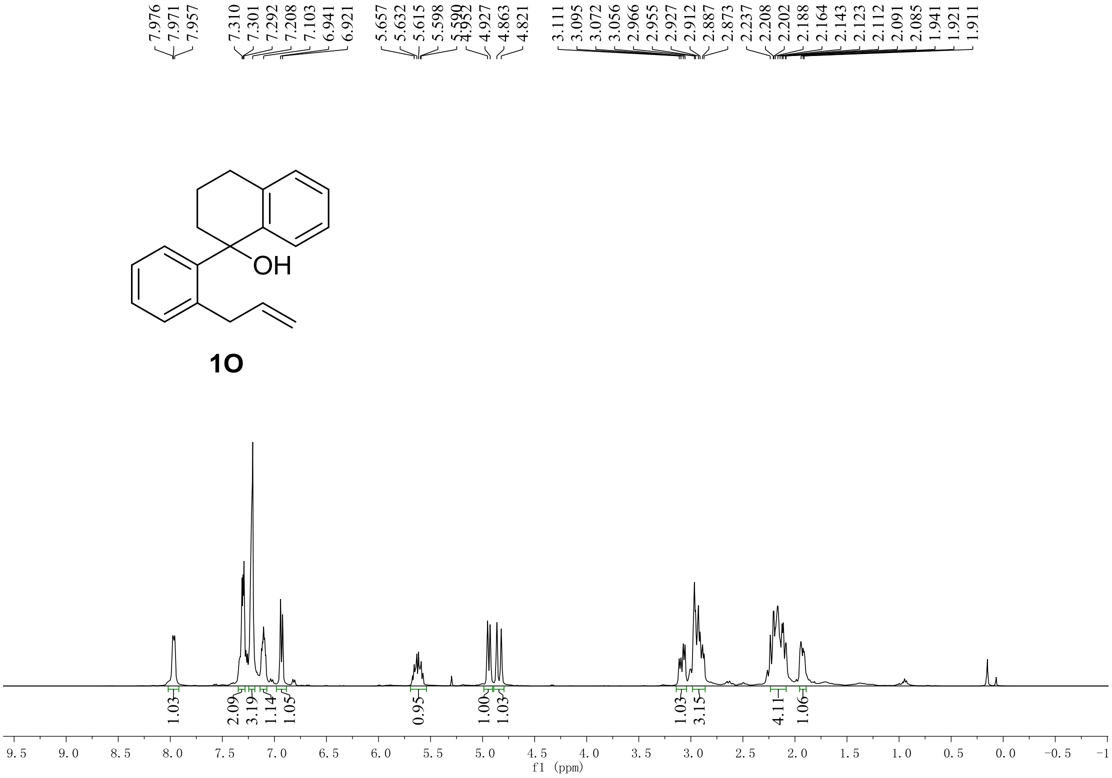

Supplementary Figure 38. ^{1}H NMR of **1O**

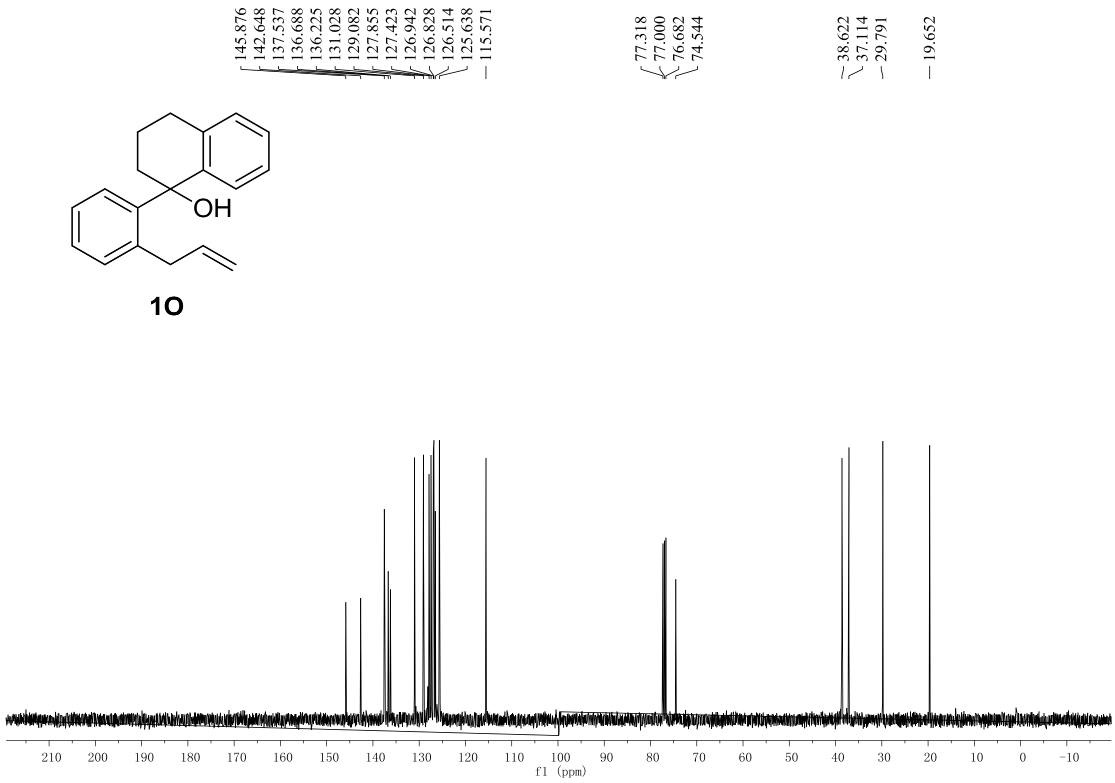

Supplementary Figure 39. ^{13}C NMR of **1O**

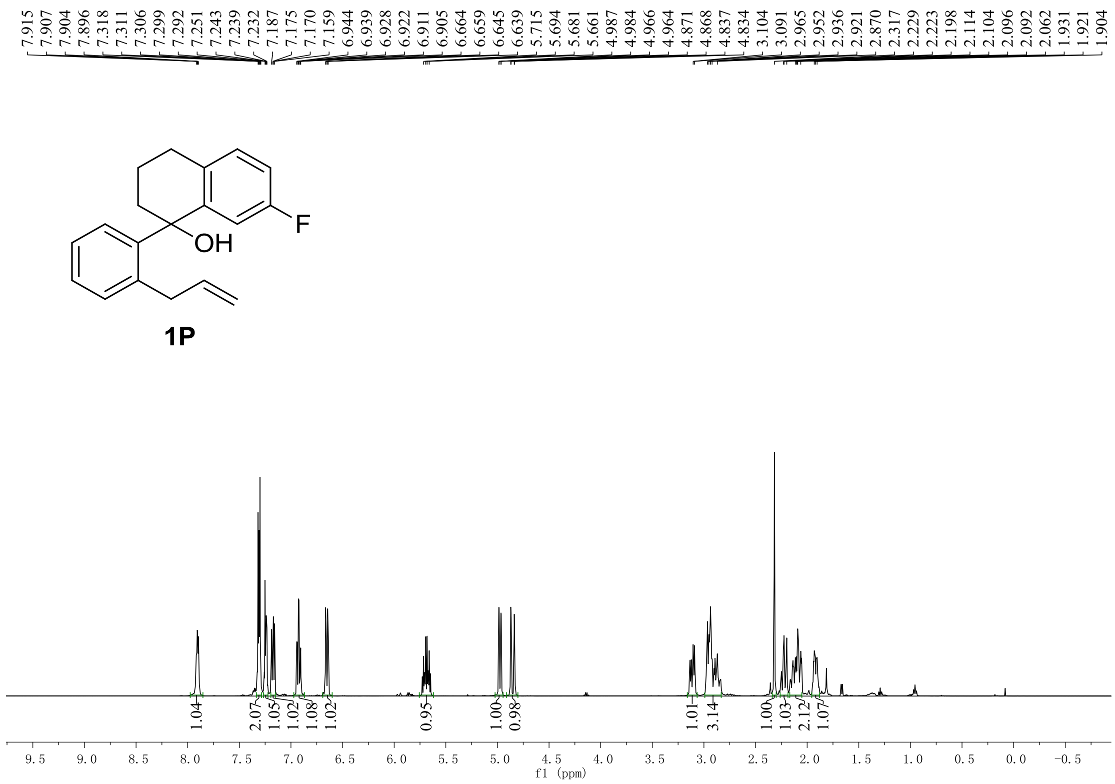

Supplementary Figure 40. ^{1}H NMR of **1P**

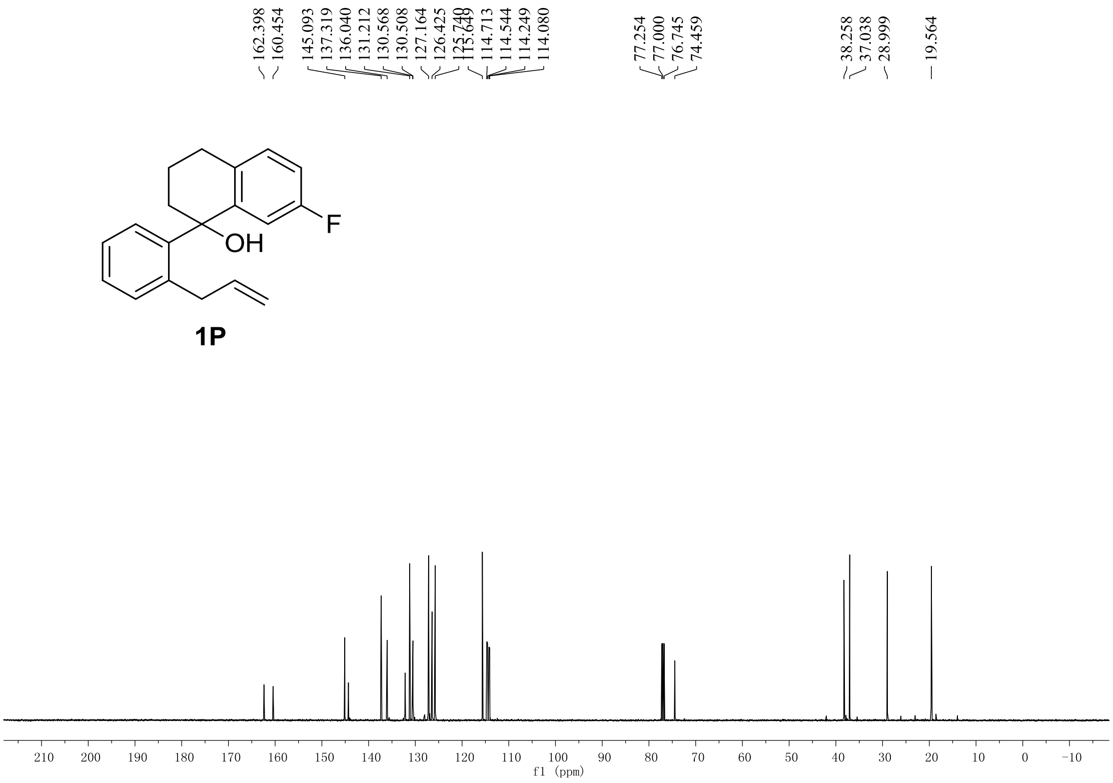

Supplementary Figure 41. ^{13}C NMR of **1P**

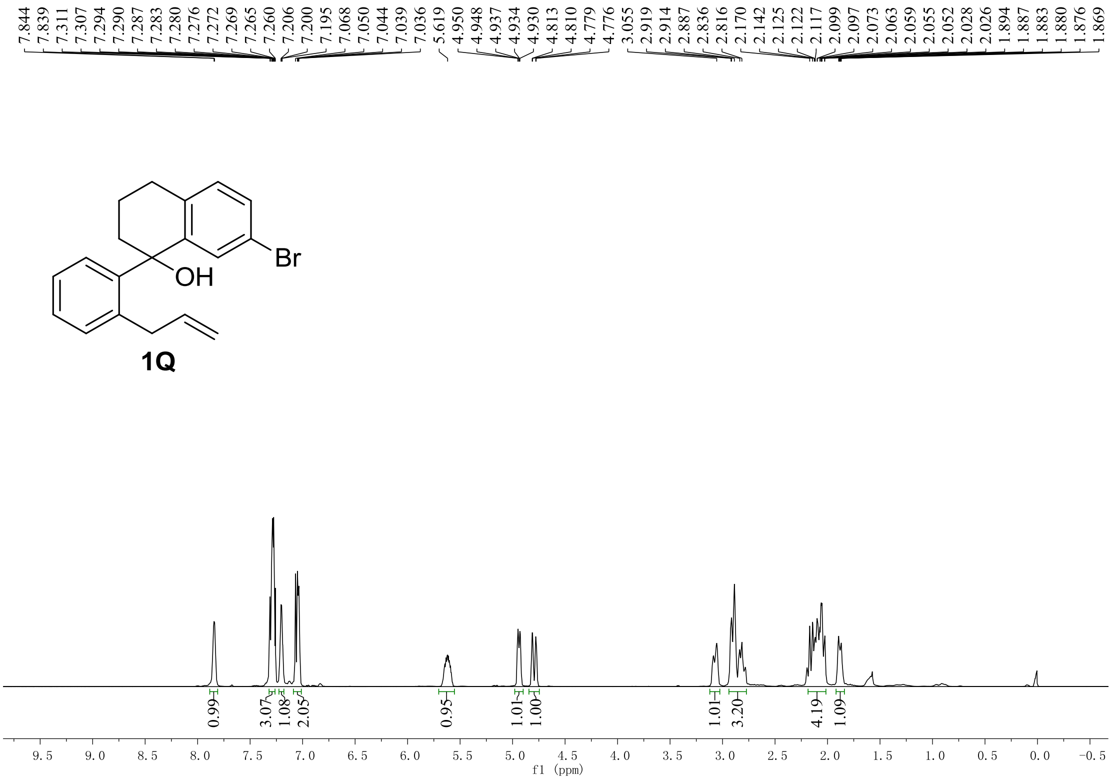

Supplementary Figure 42. ^{1}H NMR of **1Q**

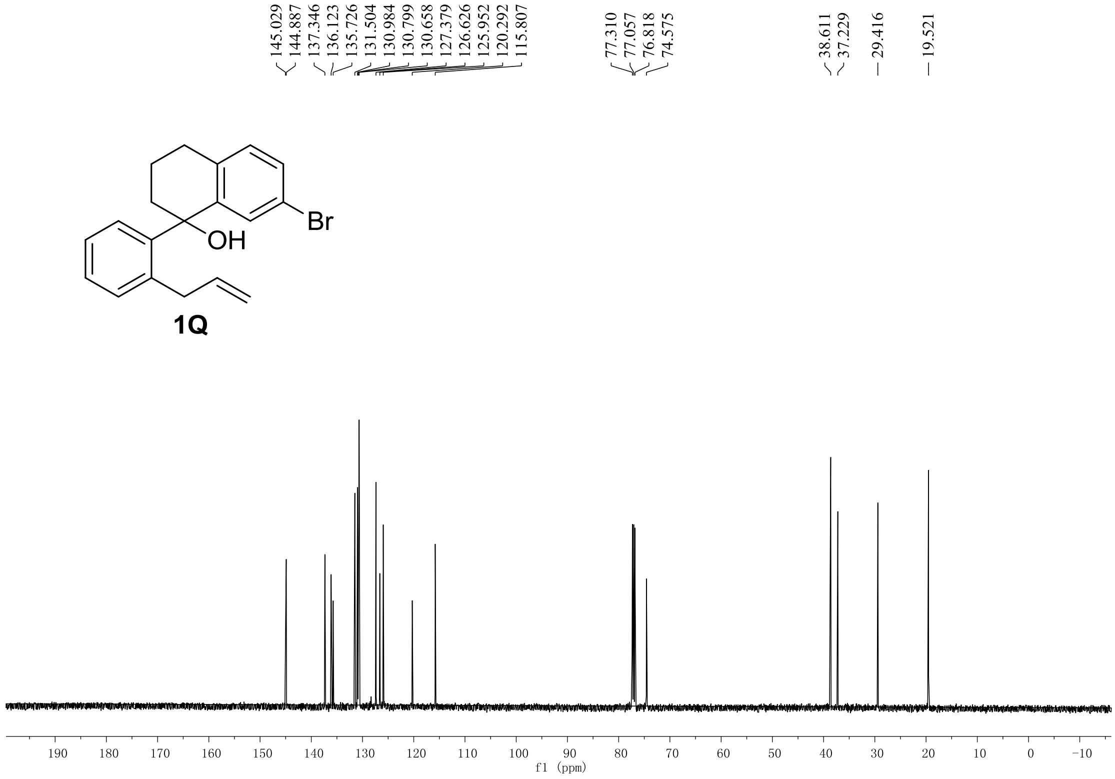

Supplementary Figure 43. ^{13}C NMR of **1Q**

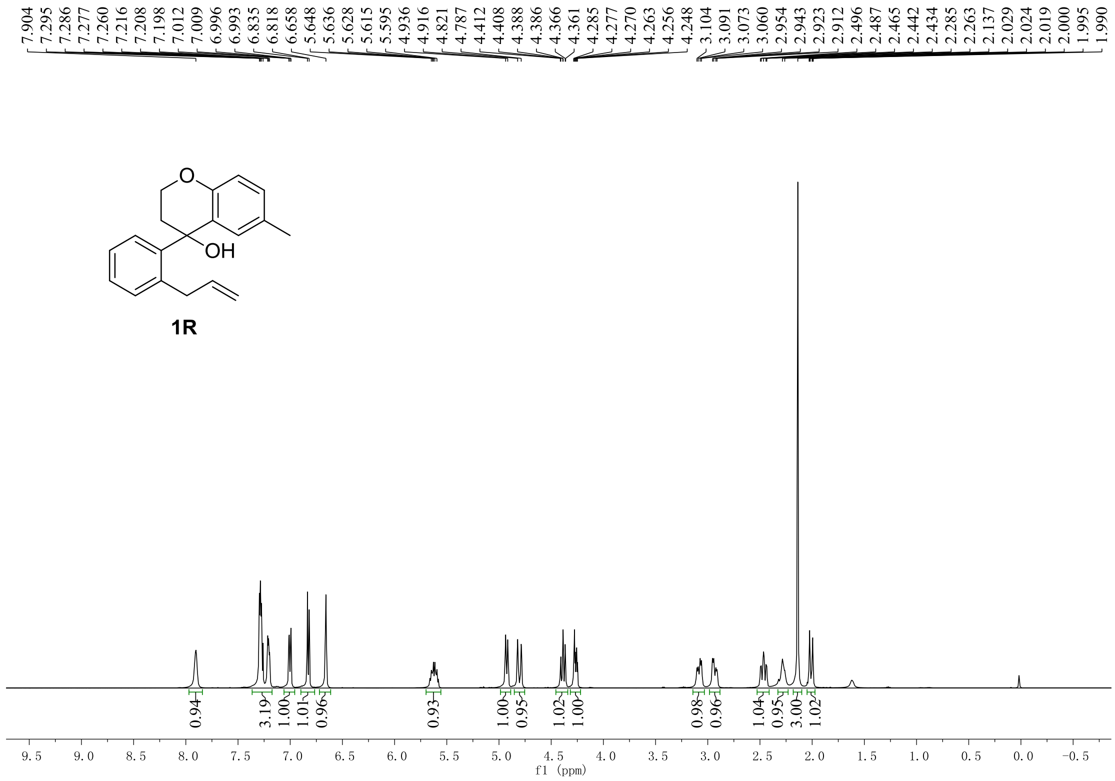

Supplementary Figure 44. ^{13}C NMR of **1R**

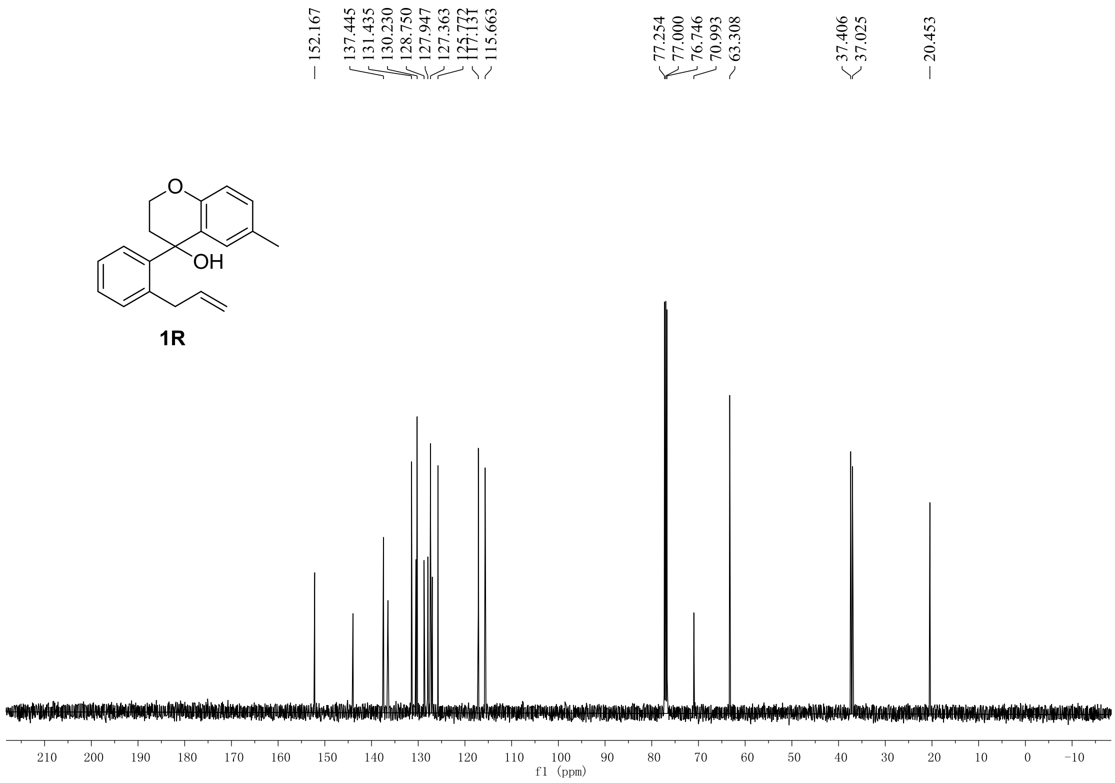

Supplementary Figure 45. ^{13}C NMR of **1R**

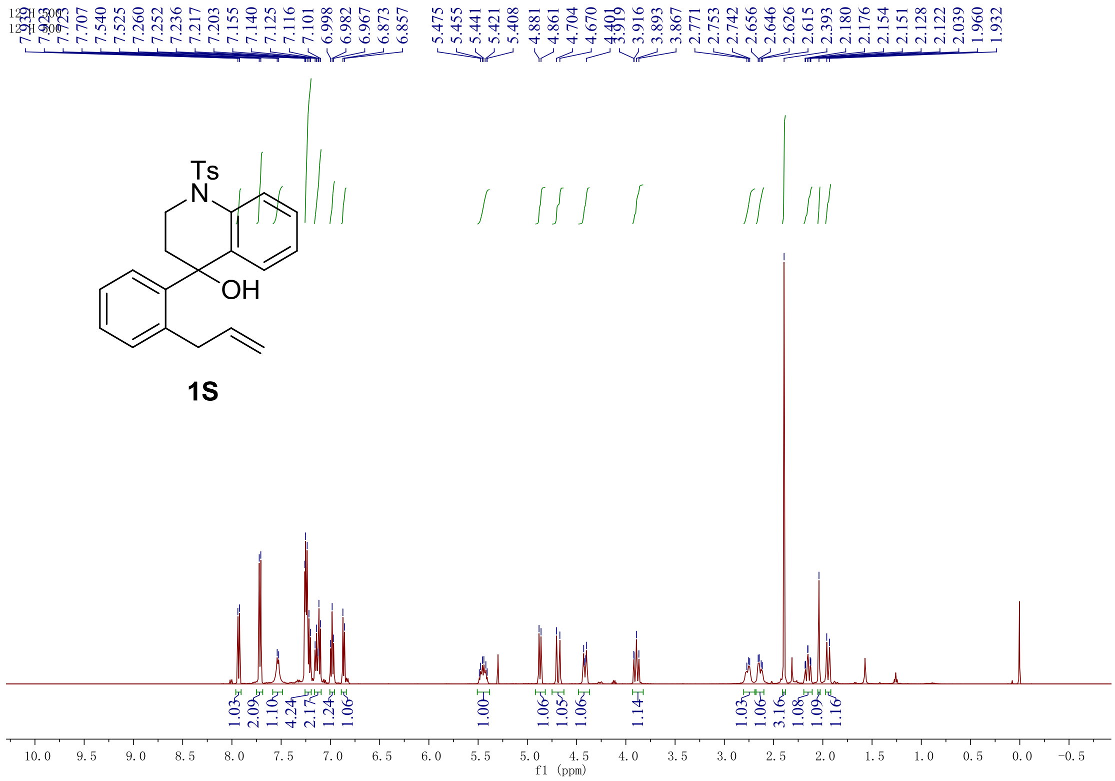

Supplementary Figure 46. ^{1}H NMR of **1S**

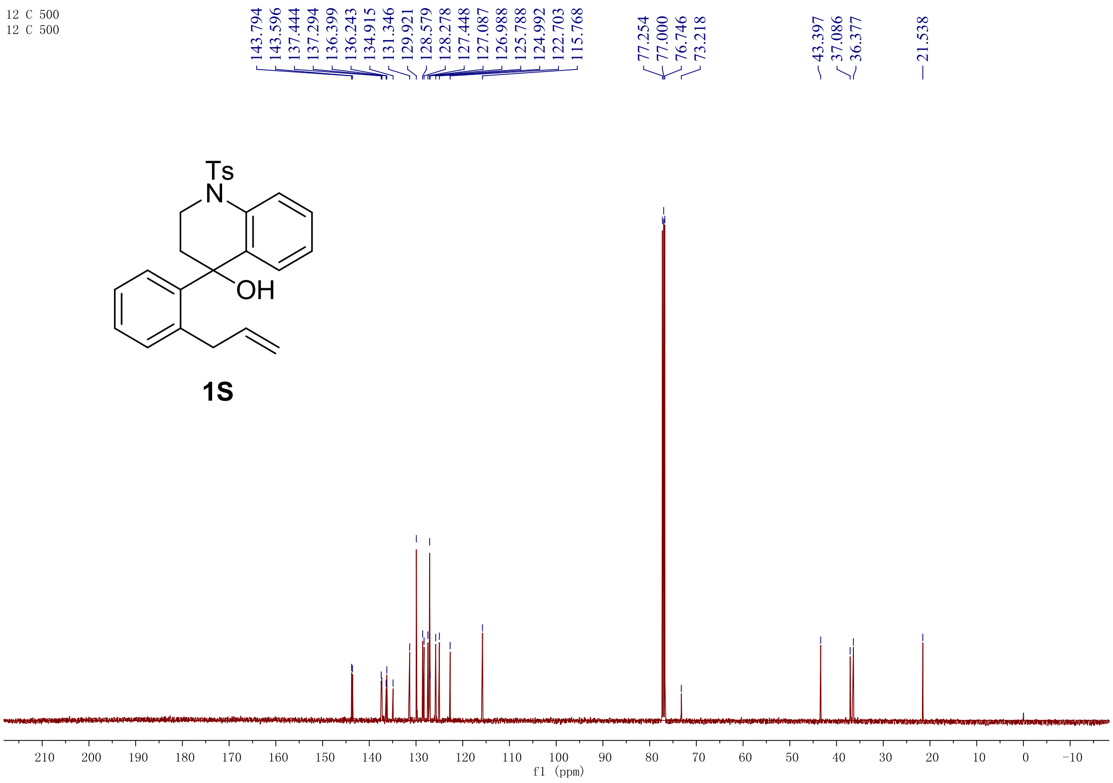

Supplementary Figure 47. ^{13}C NMR of **1S**

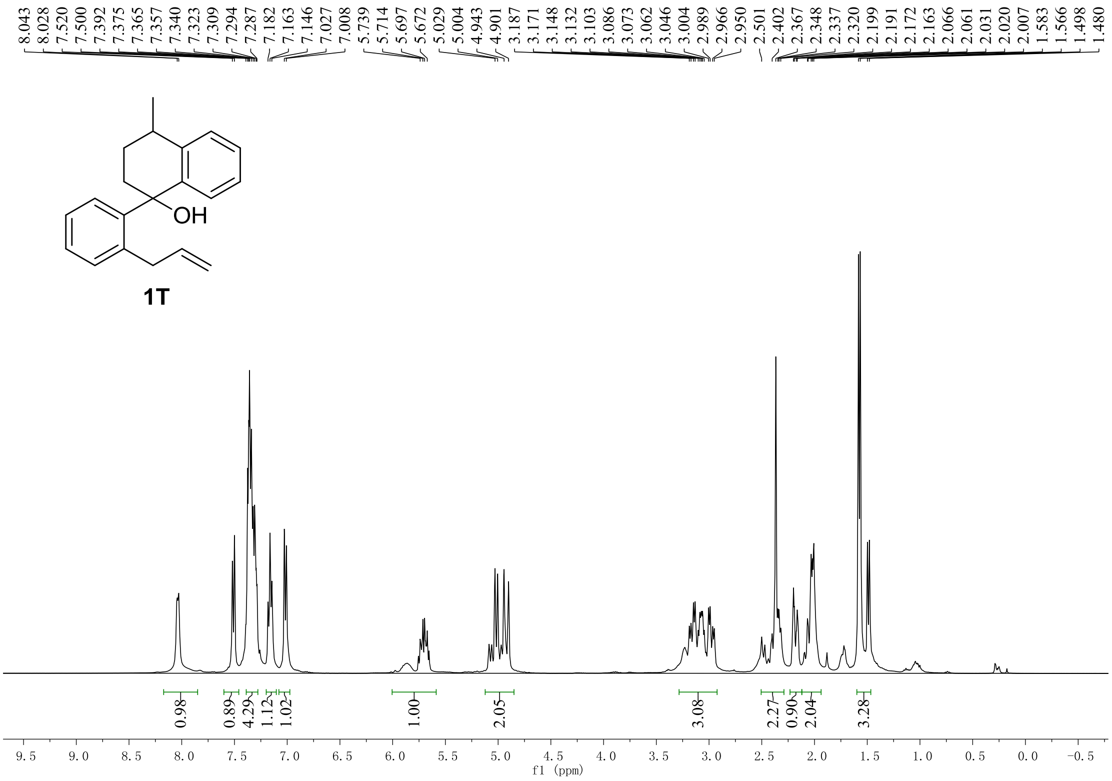

Supplementary Figure 48. ^{1}H NMR of **1T**

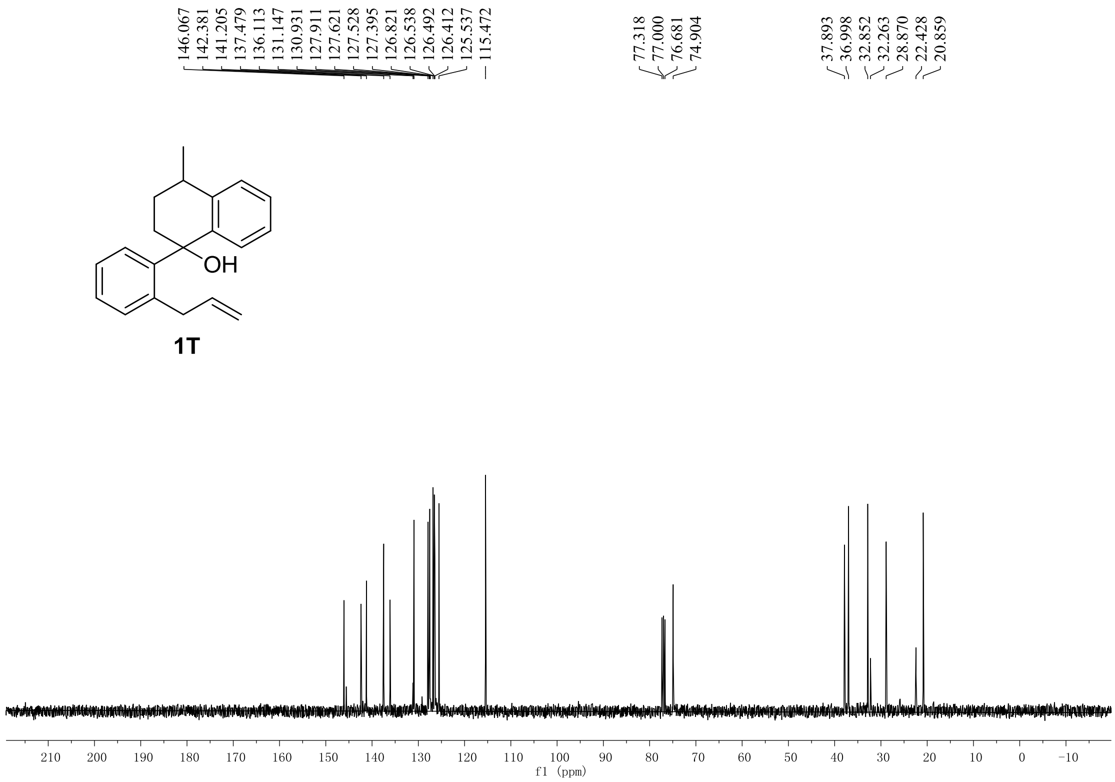

Supplementary Figure 49. ^{13}C NMR of **1T**

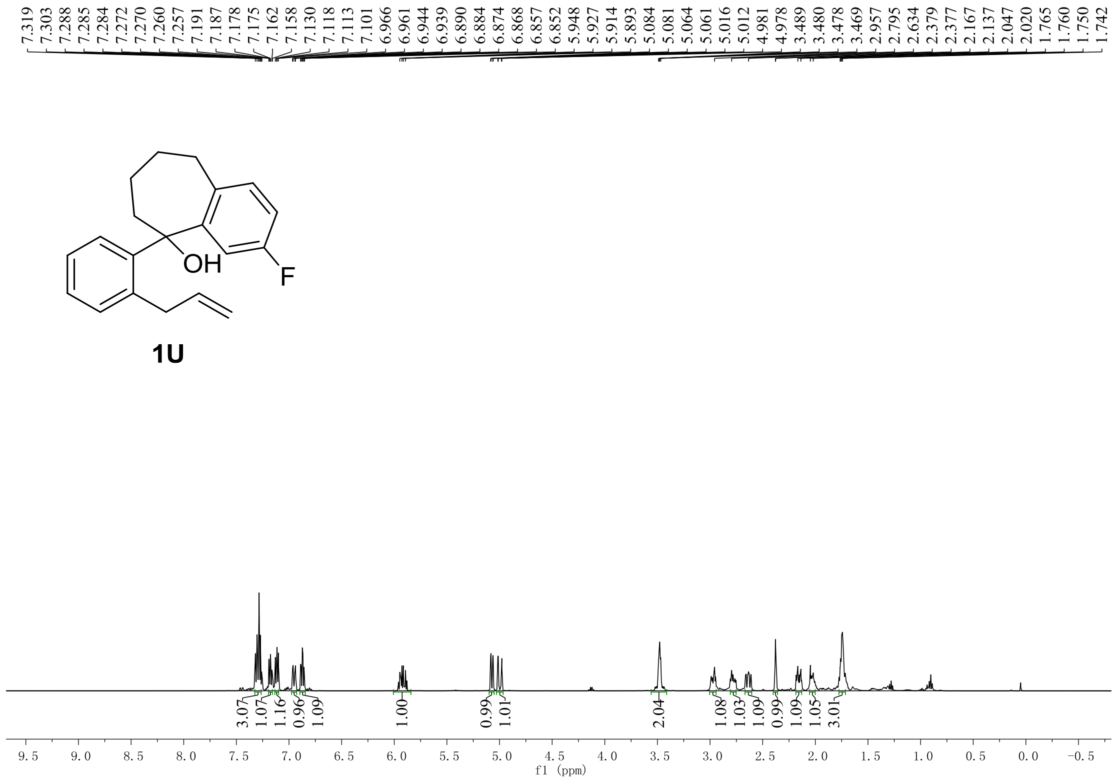

Supplementary Figure 50. ^{1}H NMR of **1U**

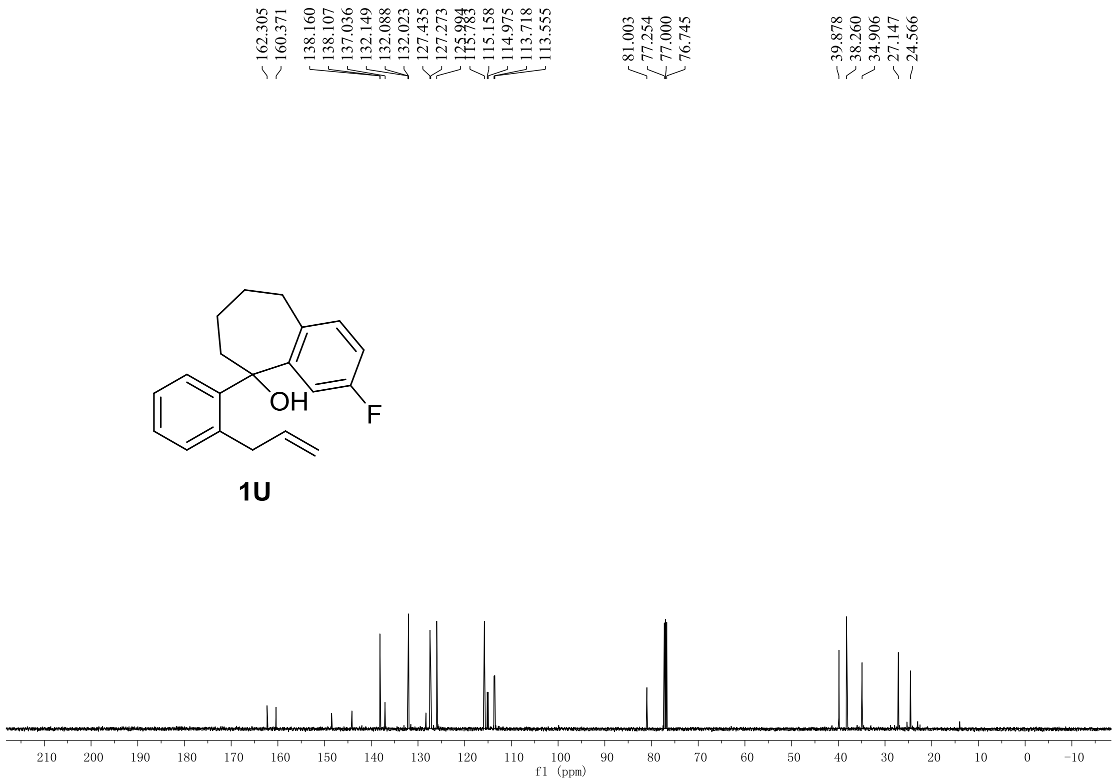

Supplementary Figure 51. ^{13}C NMR of **1U**

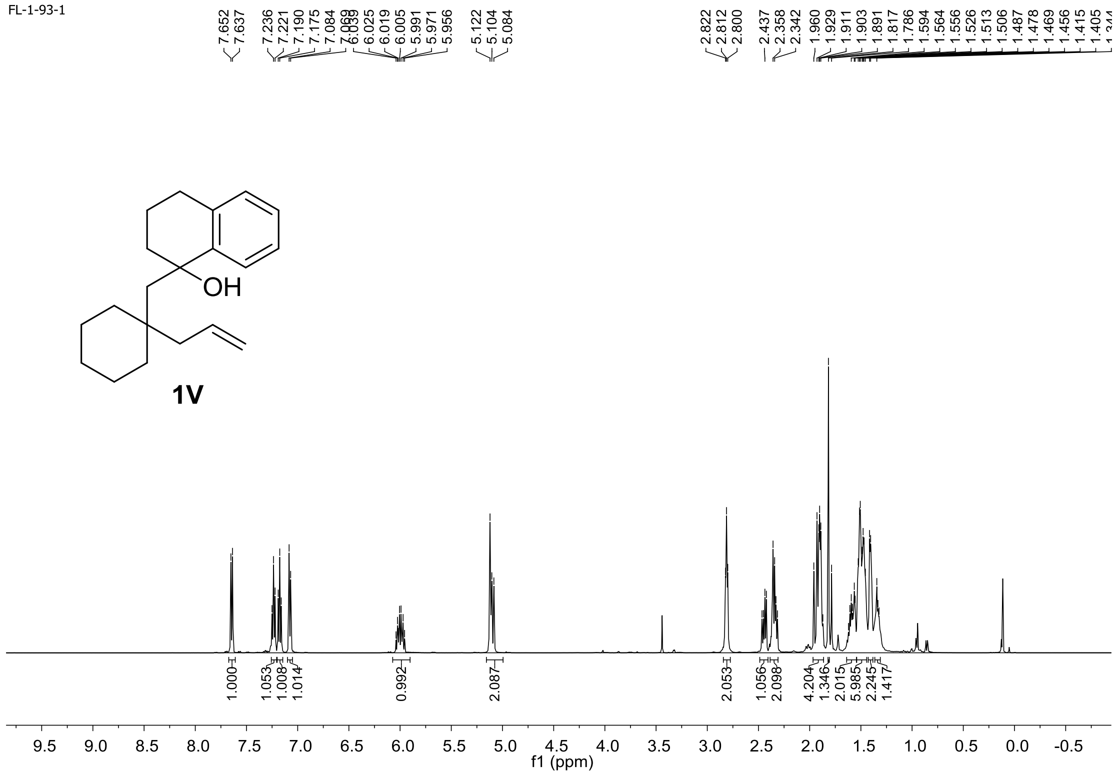

Supplementary Figure 52. ^{1}H NMR of **1V**

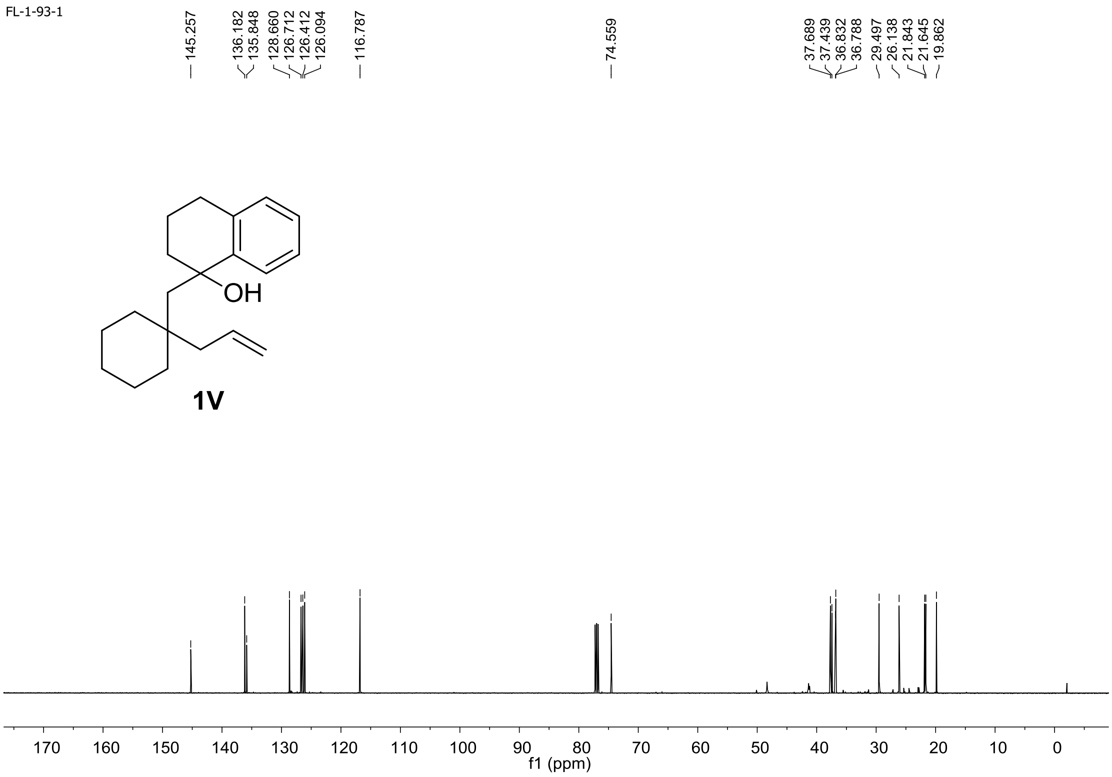

Supplementary Figure 53. ^{13}C NMR of **1V**

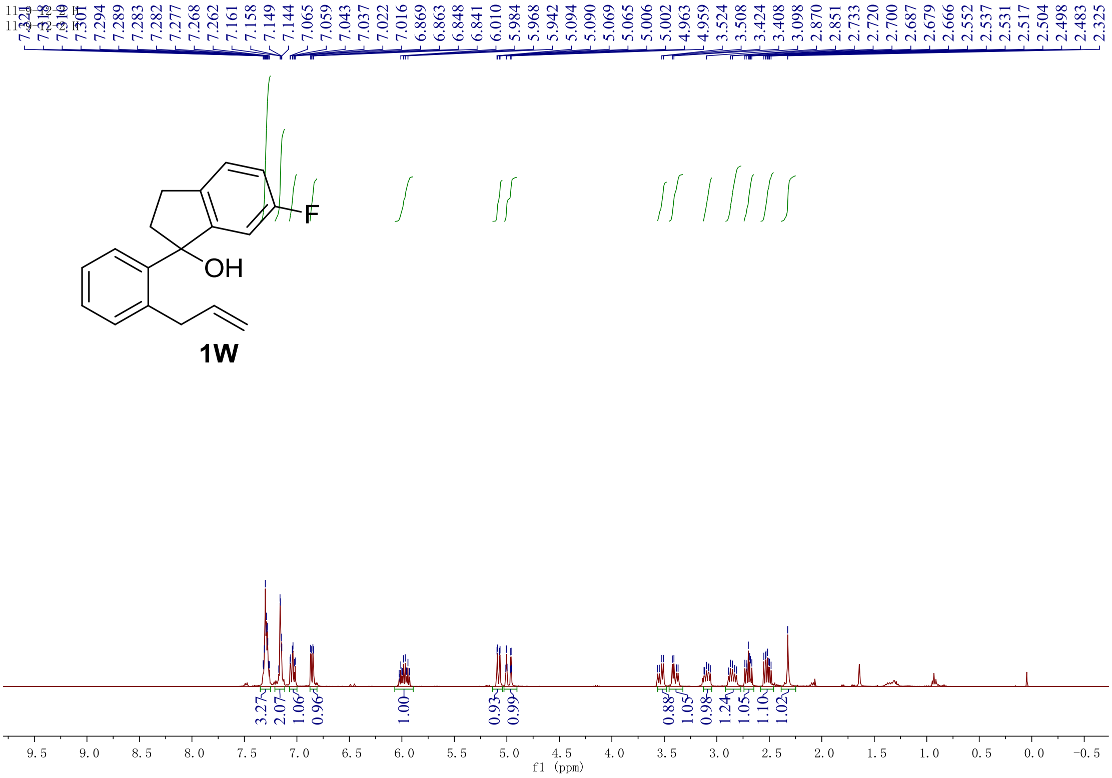

Supplementary Figure 54. ^{1}H NMR of **1W**

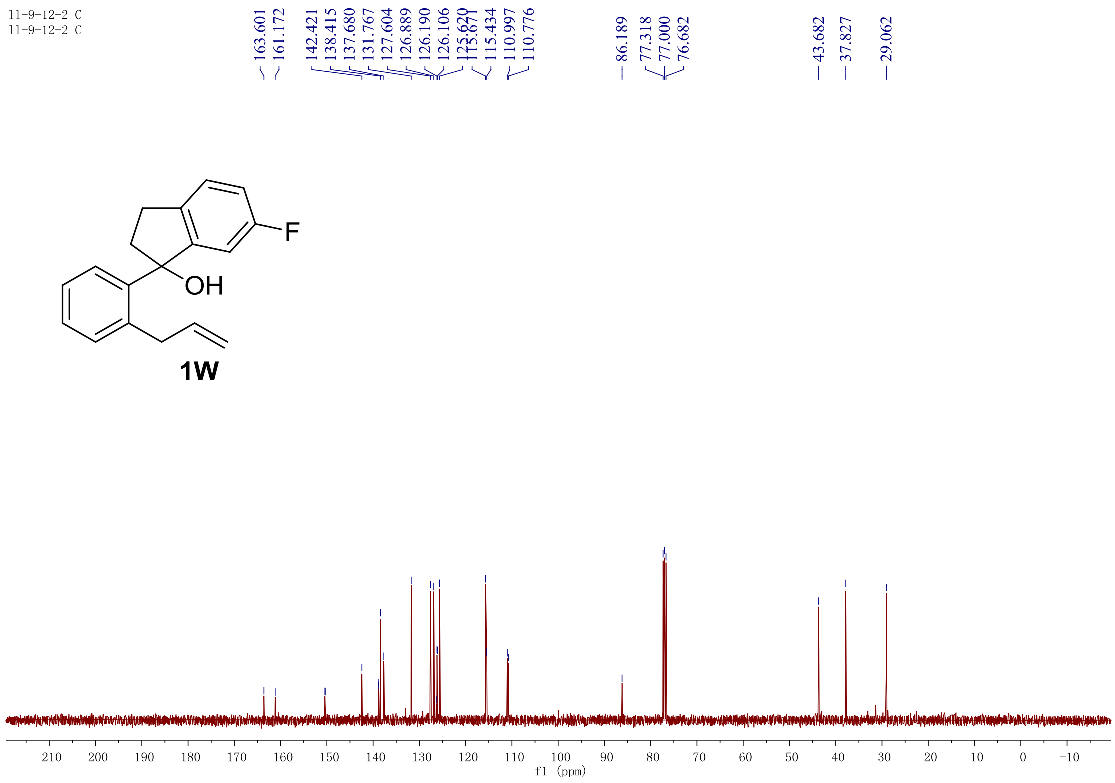

Supplementary Figure 55. ^{13}C NMR of **1W**

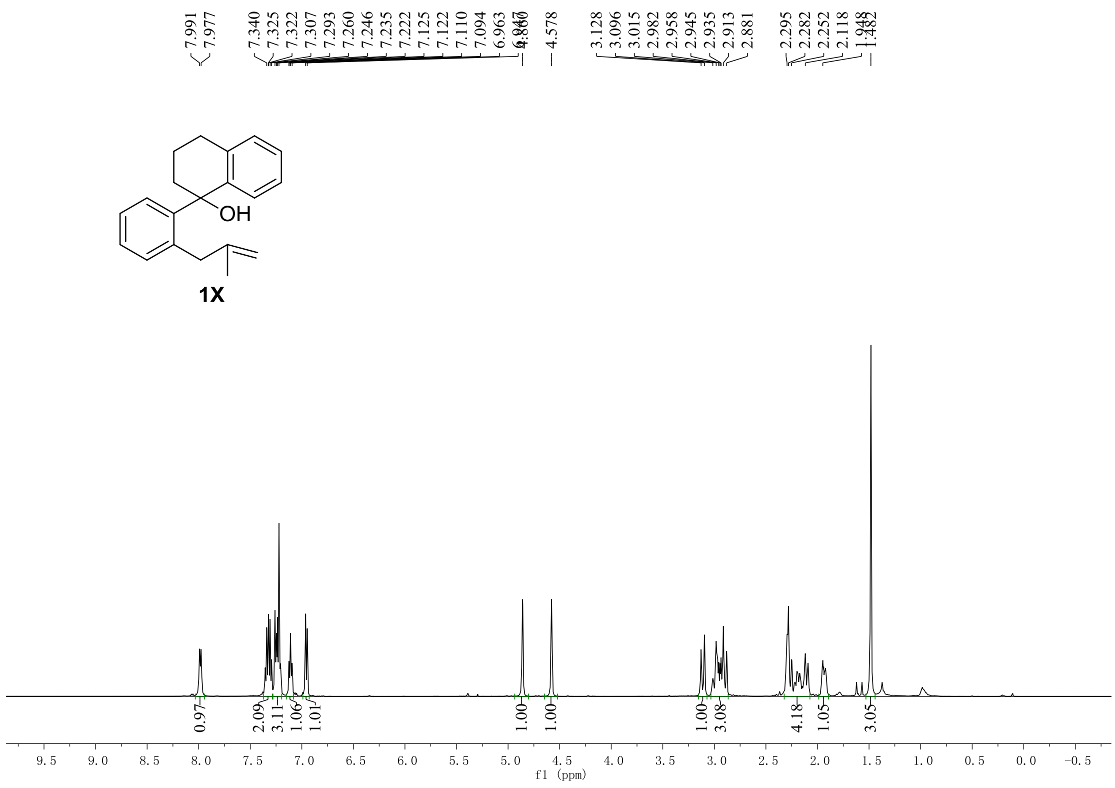

Supplementary Figure 56. ^{1}H NMR of **1X**

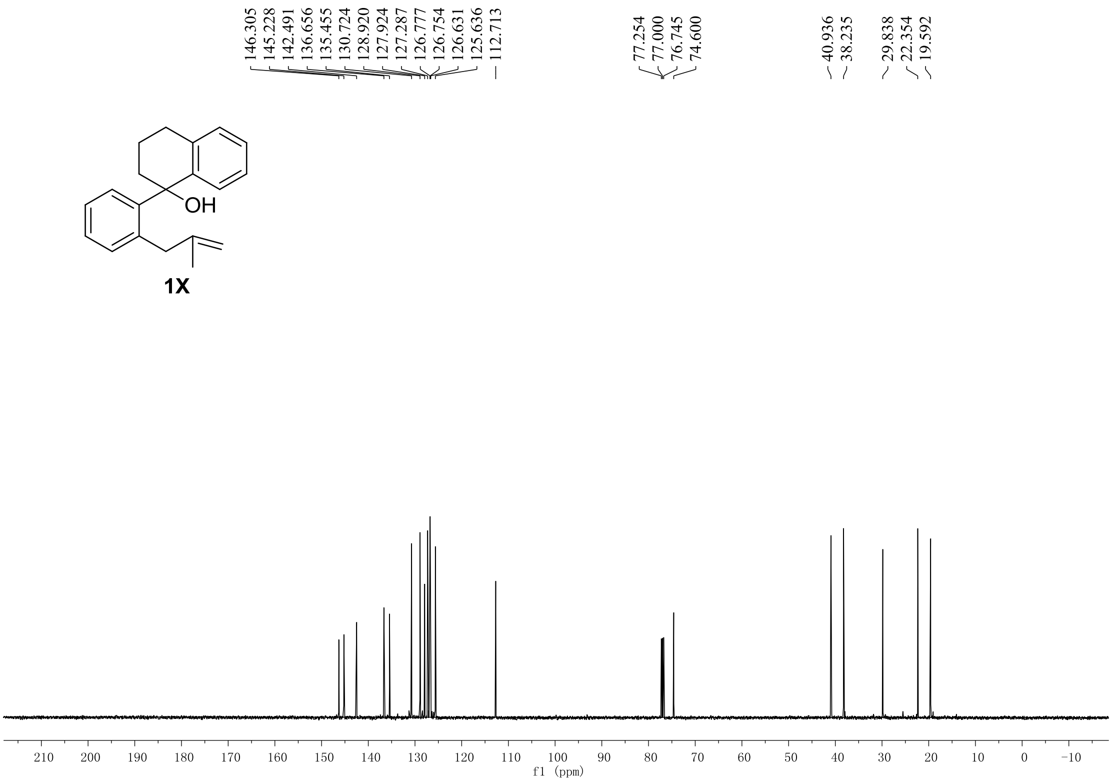

Supplementary Figure 57. ^{13}C NMR of **1X**

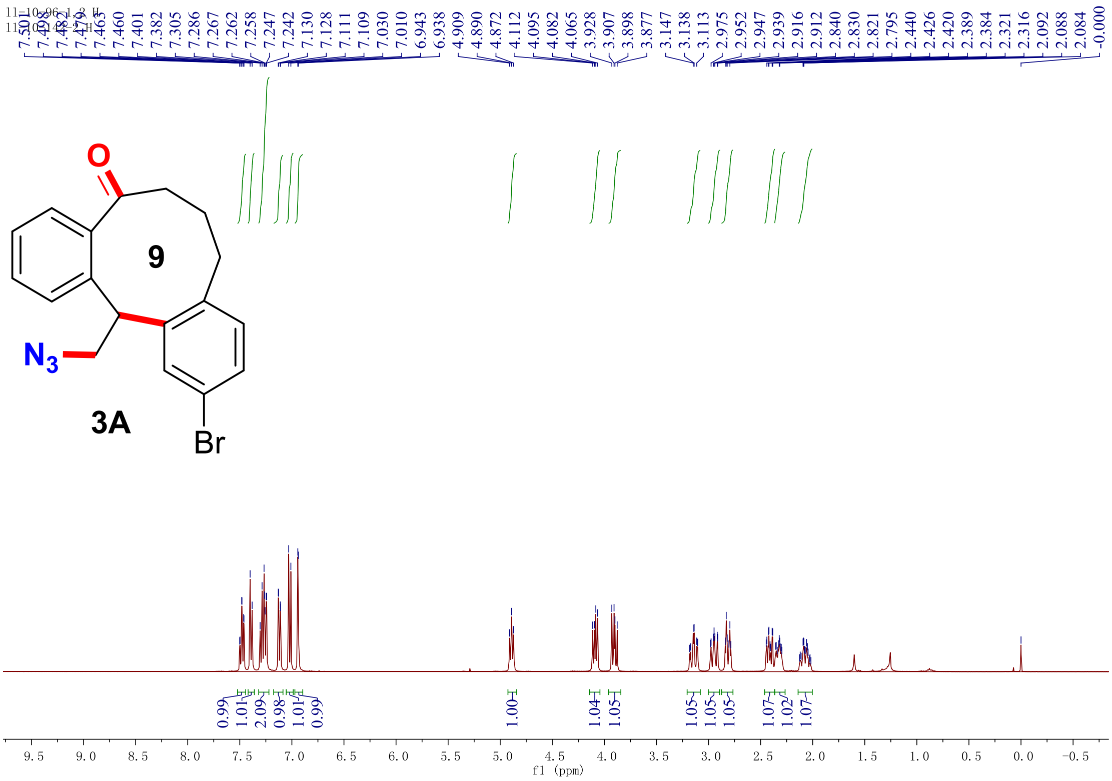

Supplementary Figure 58. ^{1}H NMR of **3A**

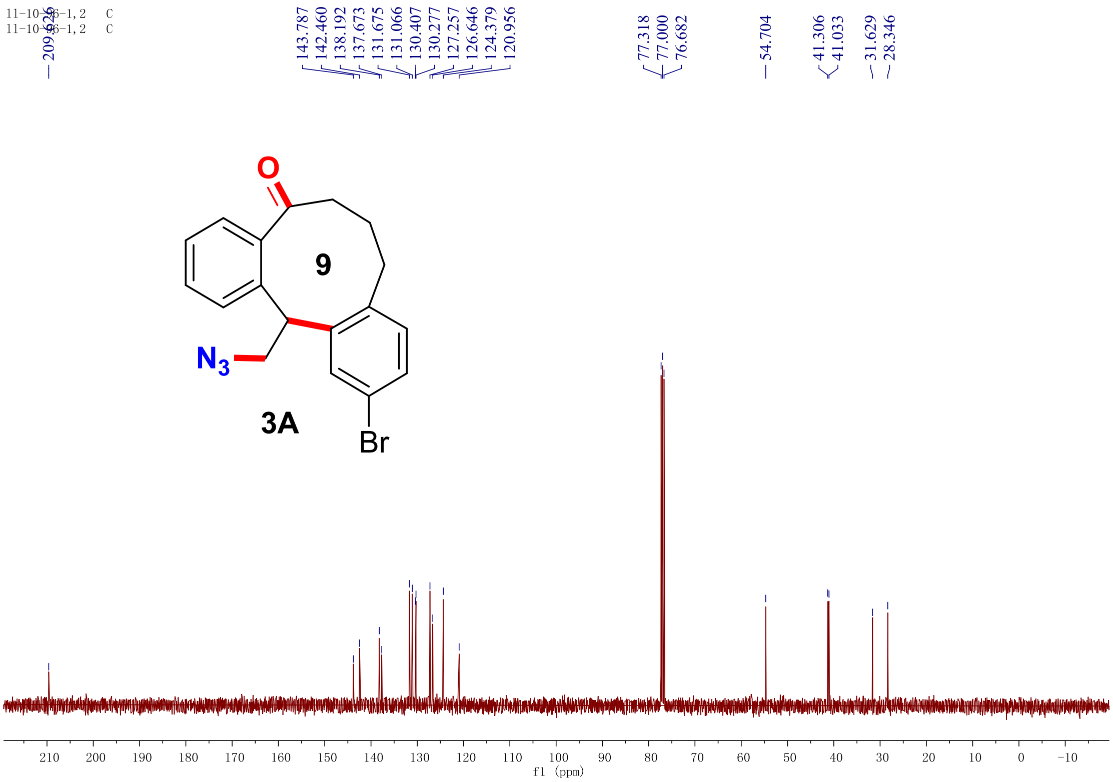

Supplementary Figure 59. ^{13}C NMR of **3A**

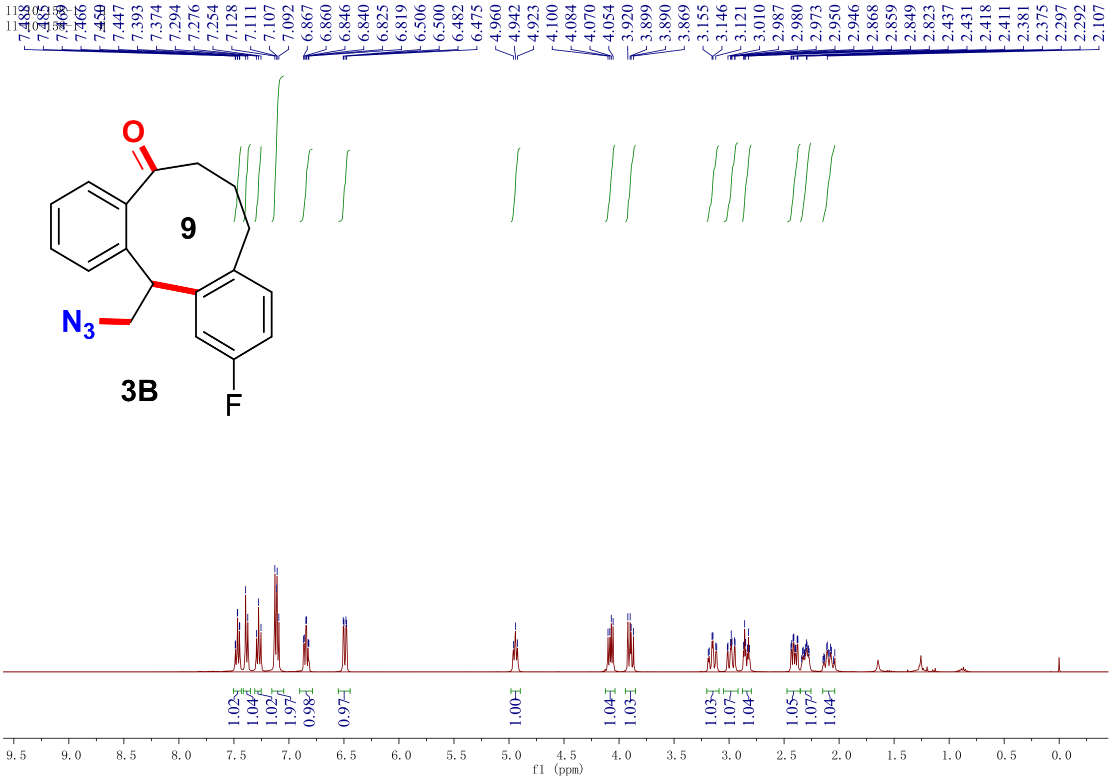

Supplementary Figure 60. ^{1}H NMR of **3B**

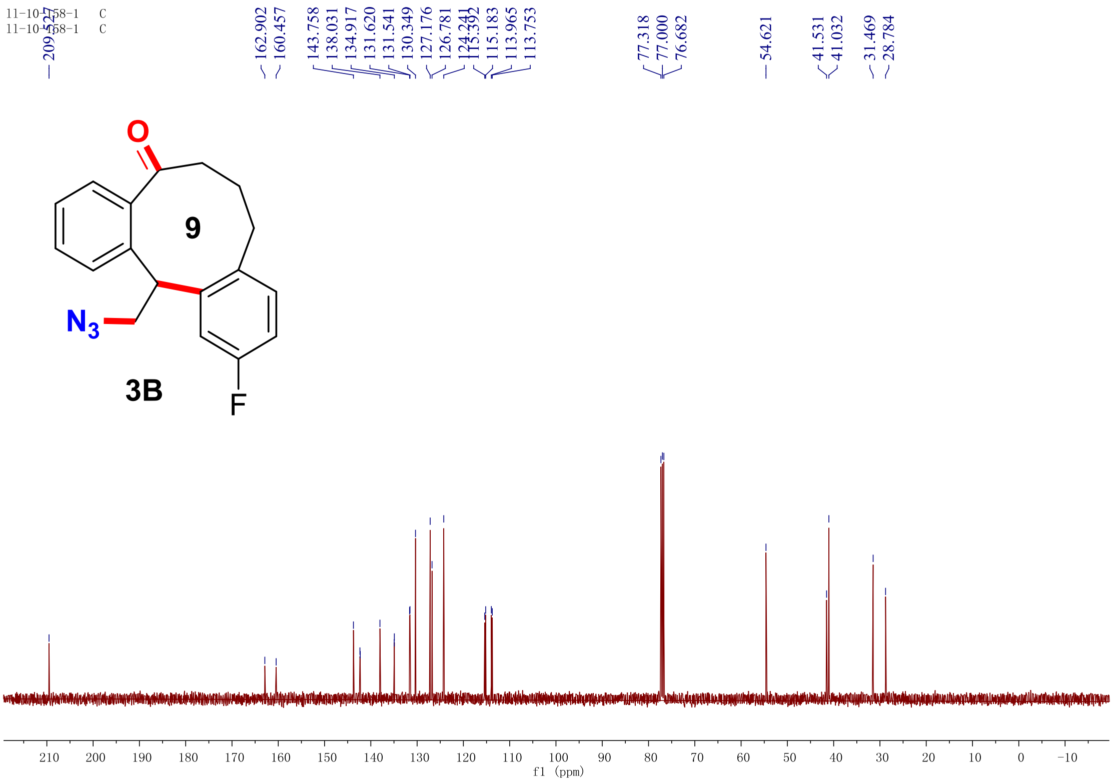

Supplementary Figure 61. ^{13}C NMR of **3B**

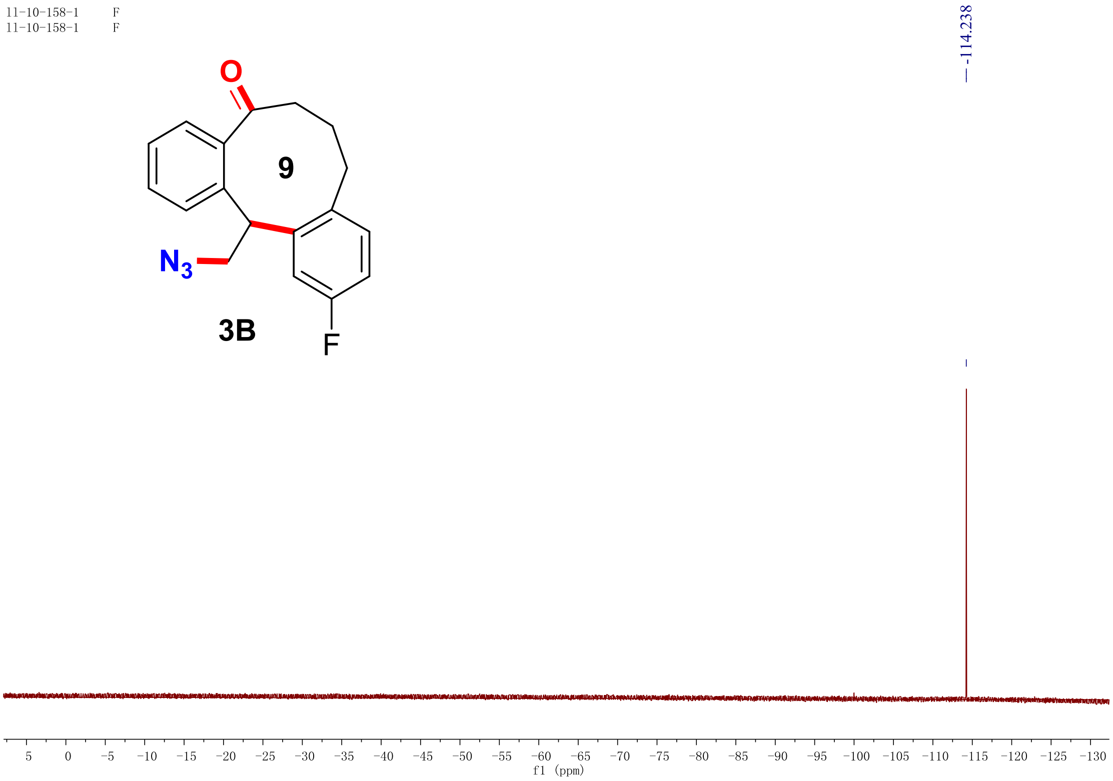

Supplementary Figure 62. ^{19}F NMR of **3B**

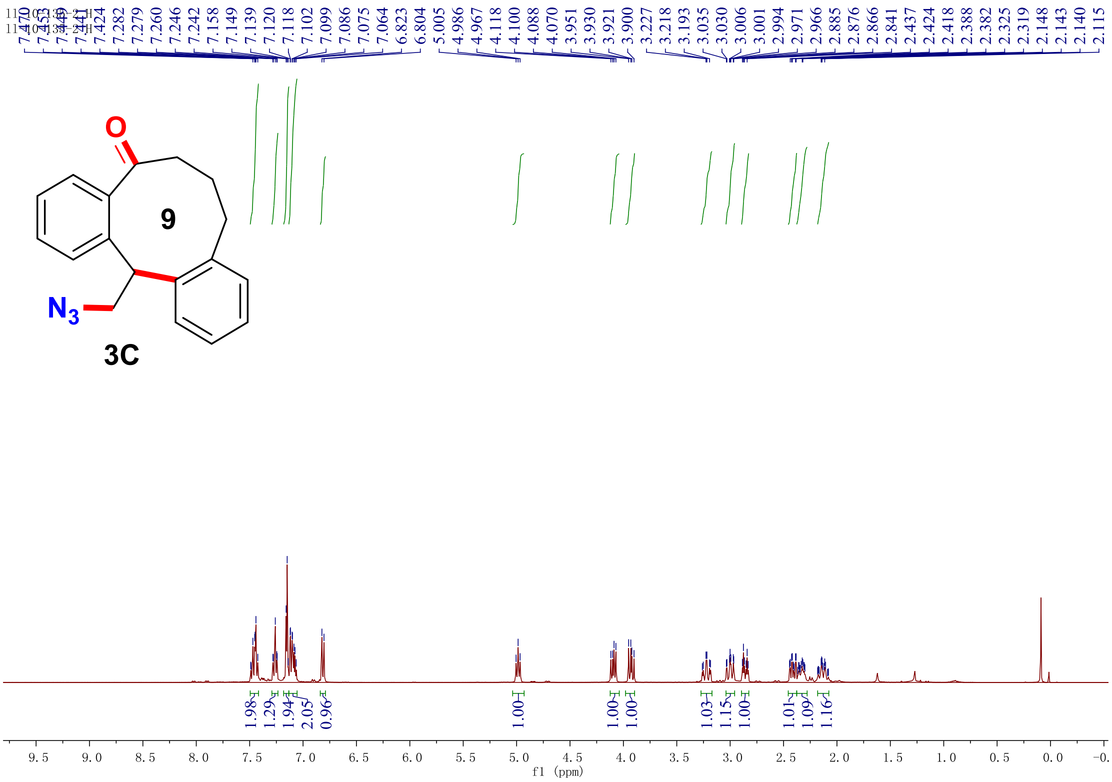

Supplementary Figure 63. ^{1}H NMR of **3C**

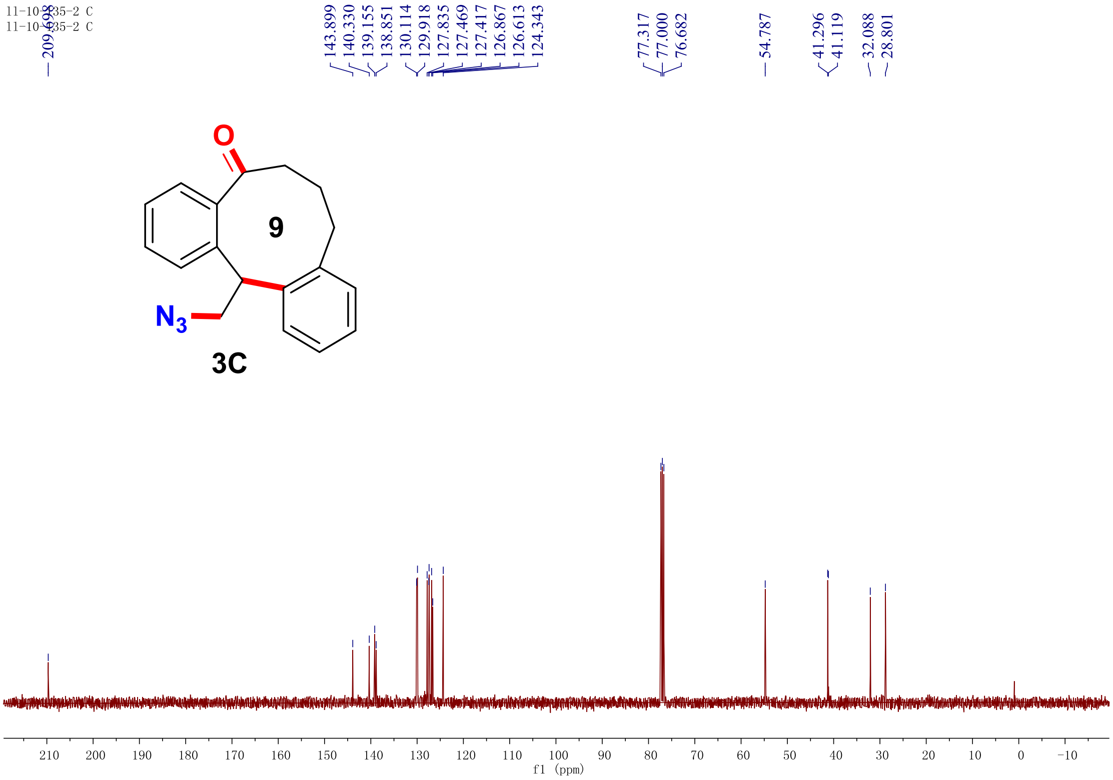

Supplementary Figure 64. ^{13}C NMR of **3C**

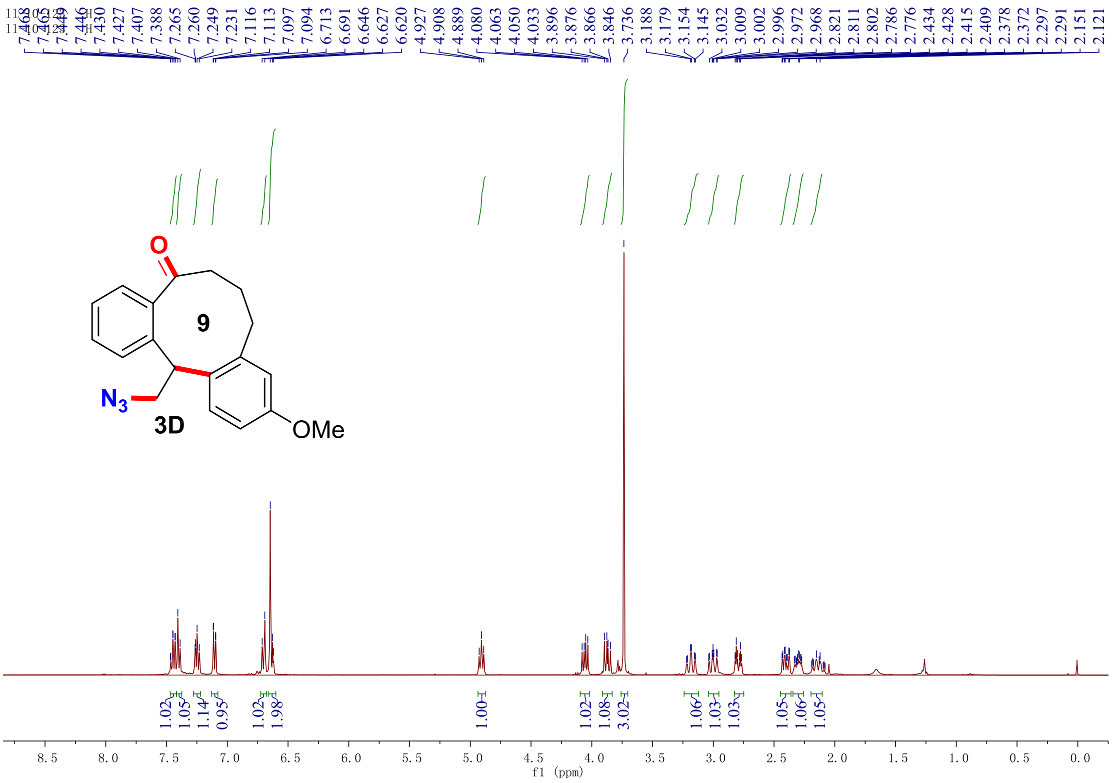

Supplementary Figure 65. ^{1}H NMR of **3D**

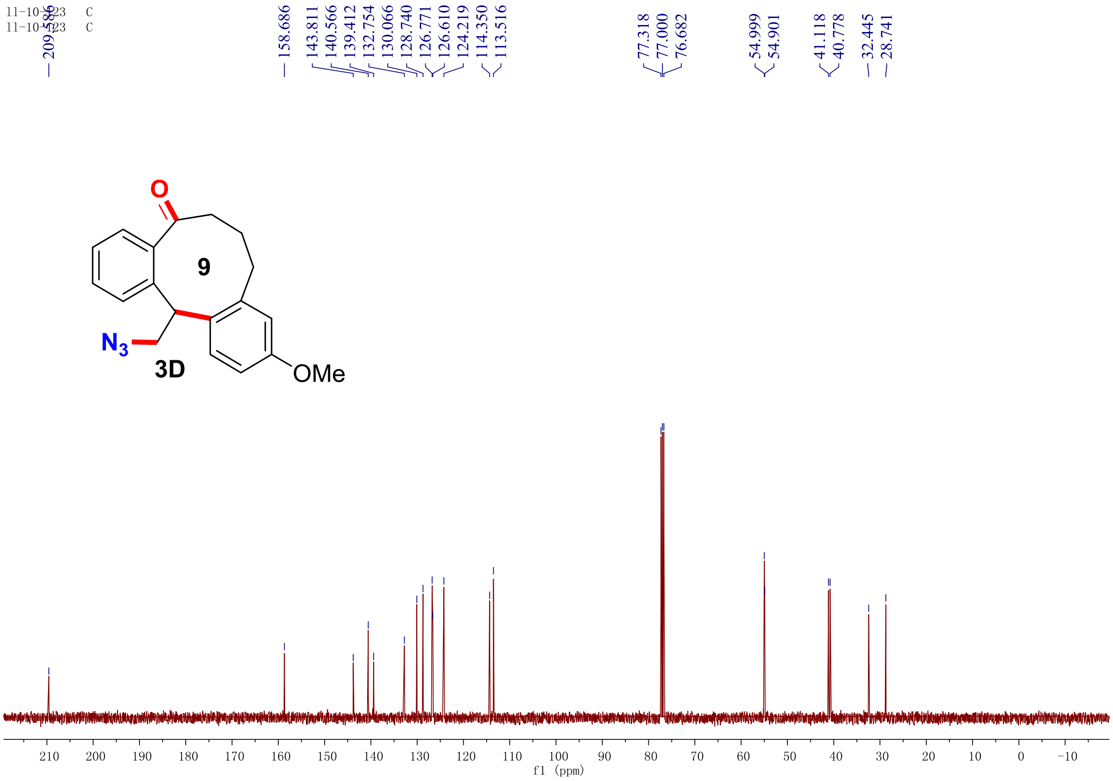

Supplementary Figure 66. ^{13}C NMR of **3D**

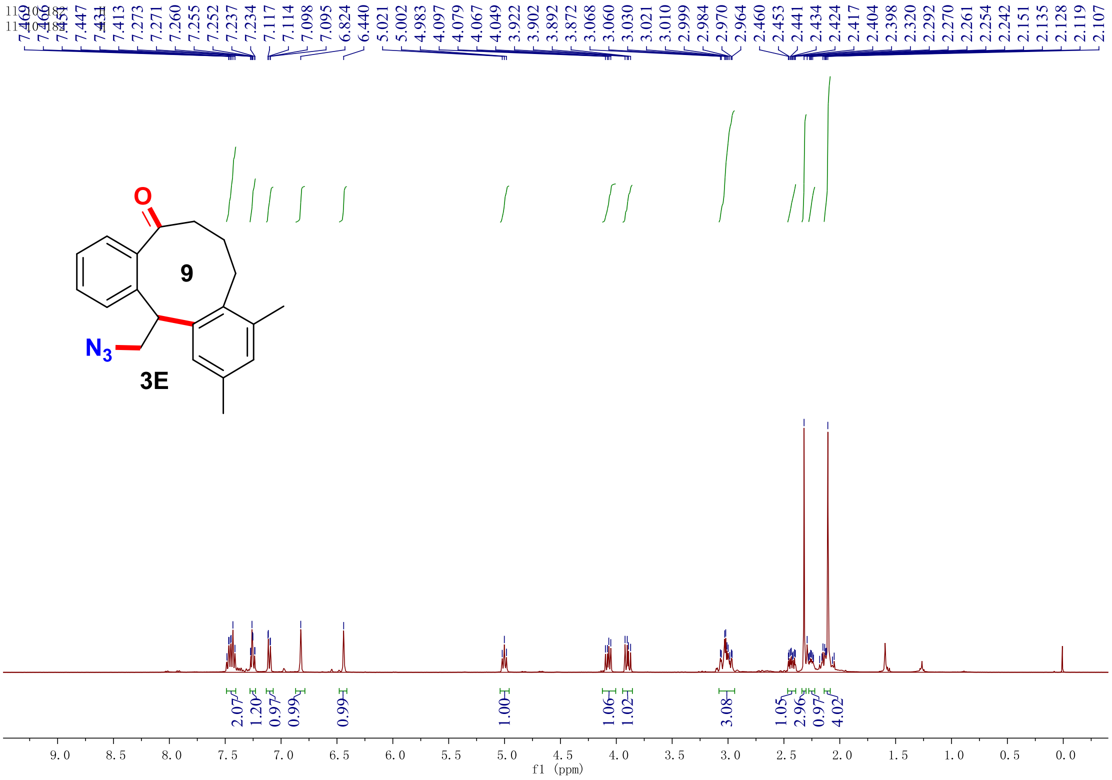

Supplementary Figure 67. ^{1}H NMR of **3E**

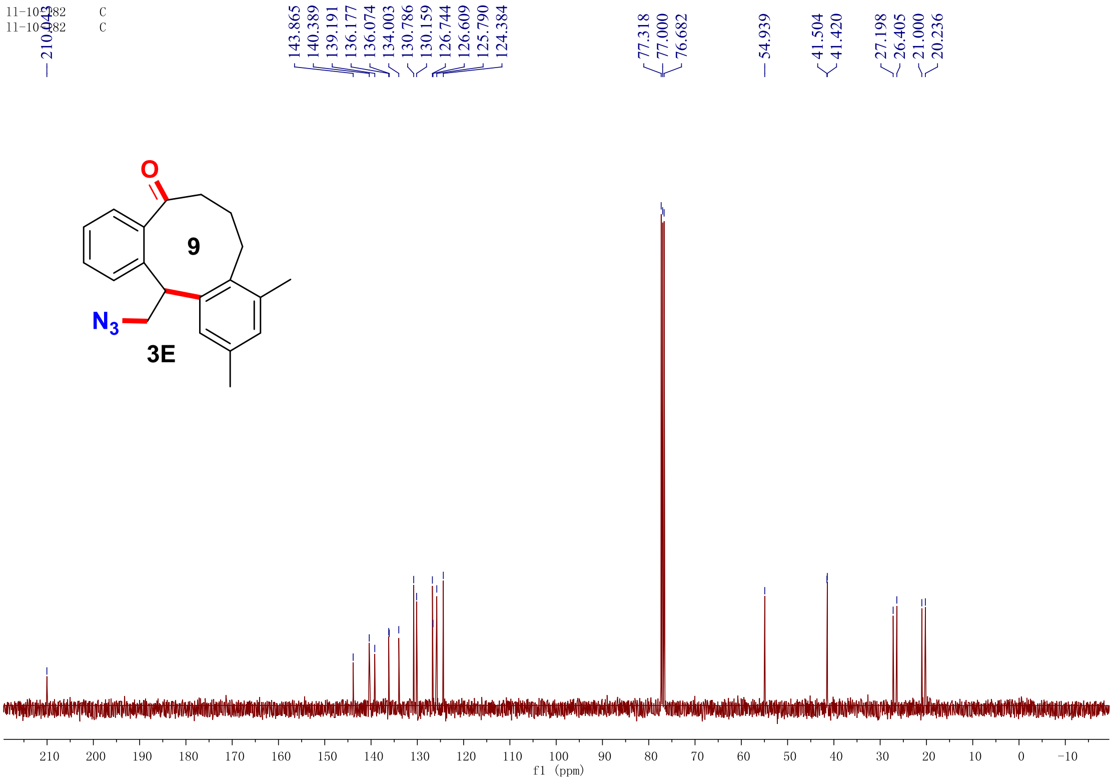

Supplementary Figure 68. ^{13}C NMR of **3E**

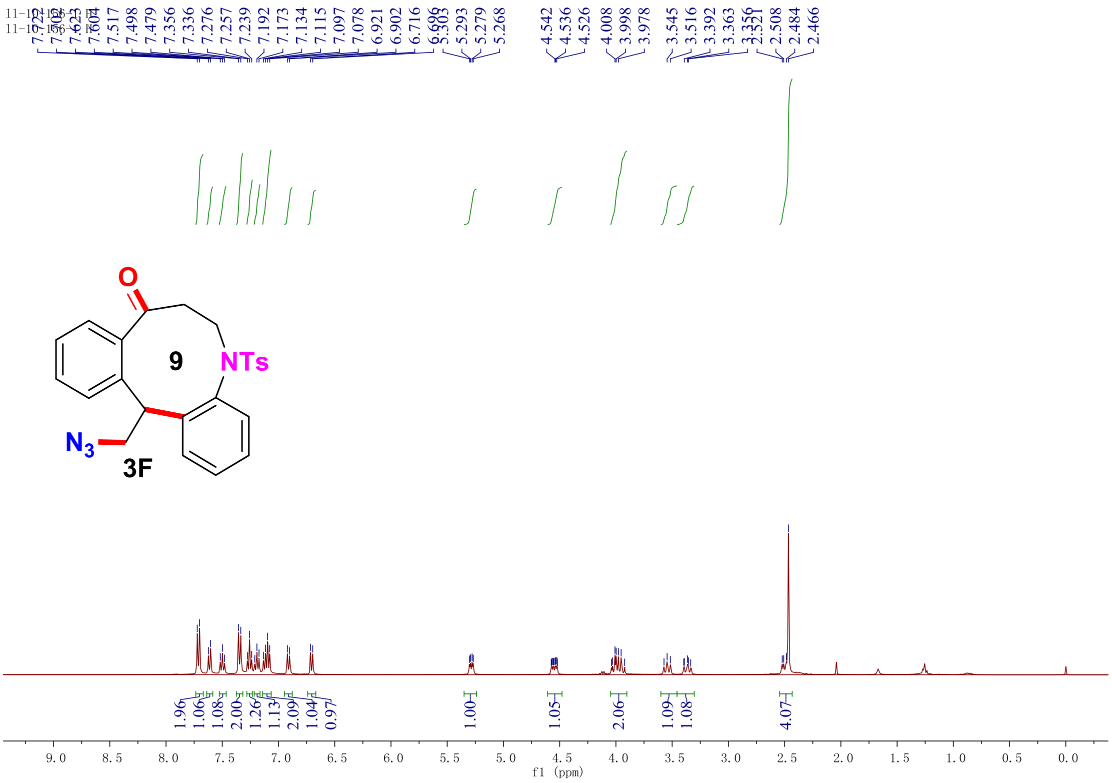

Supplementary Figure 69. ^{1}H NMR of **3F**

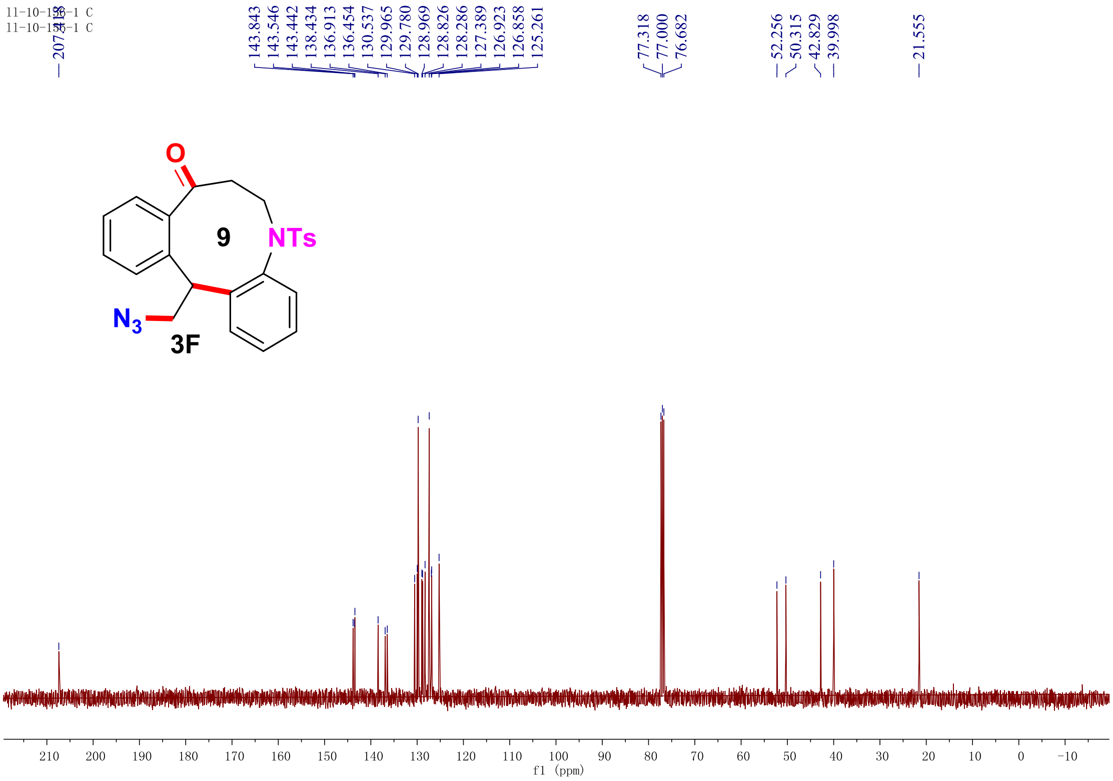

Supplementary Figure 70. ^{13}C NMR of **3F**

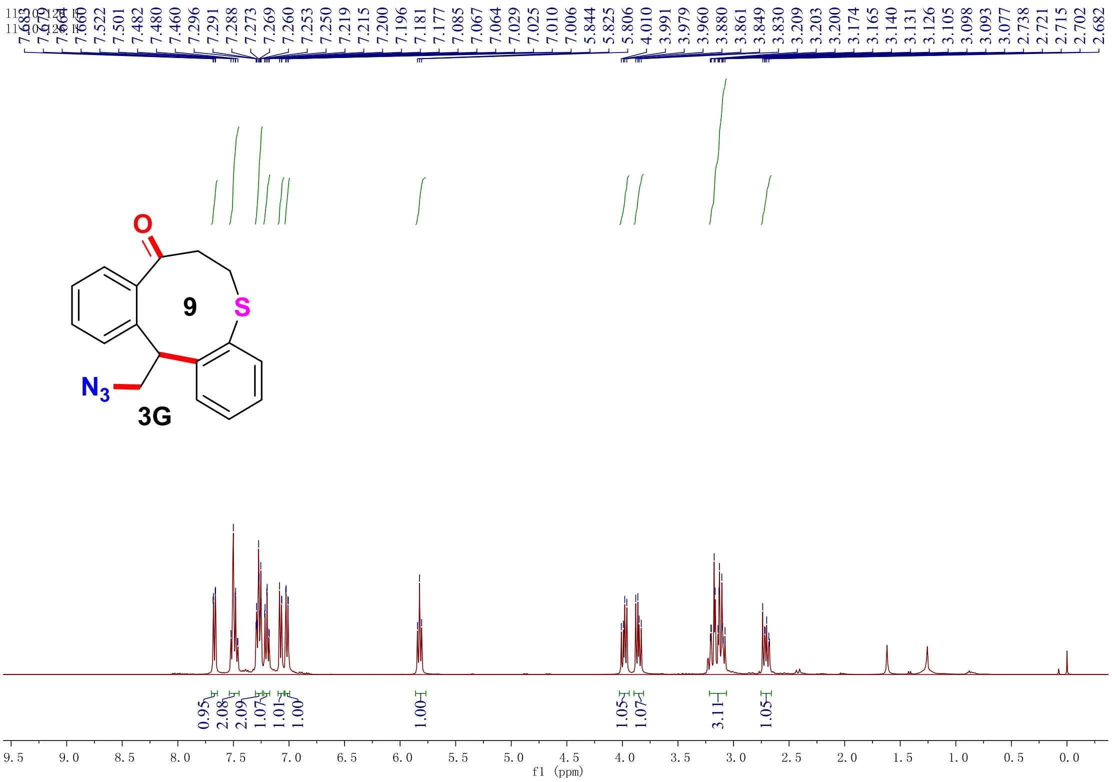

Supplementary Figure 71. ^{1}H NMR of **3G**

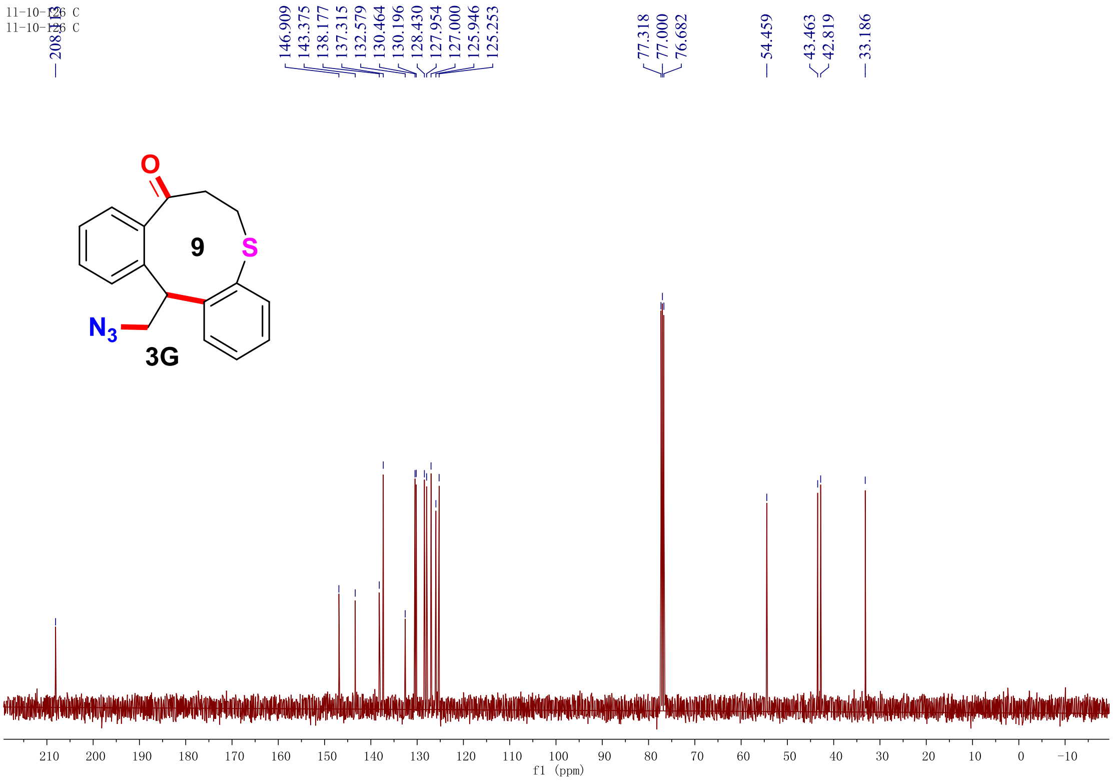

Supplementary Figure 72. ^{13}C NMR of **3G**

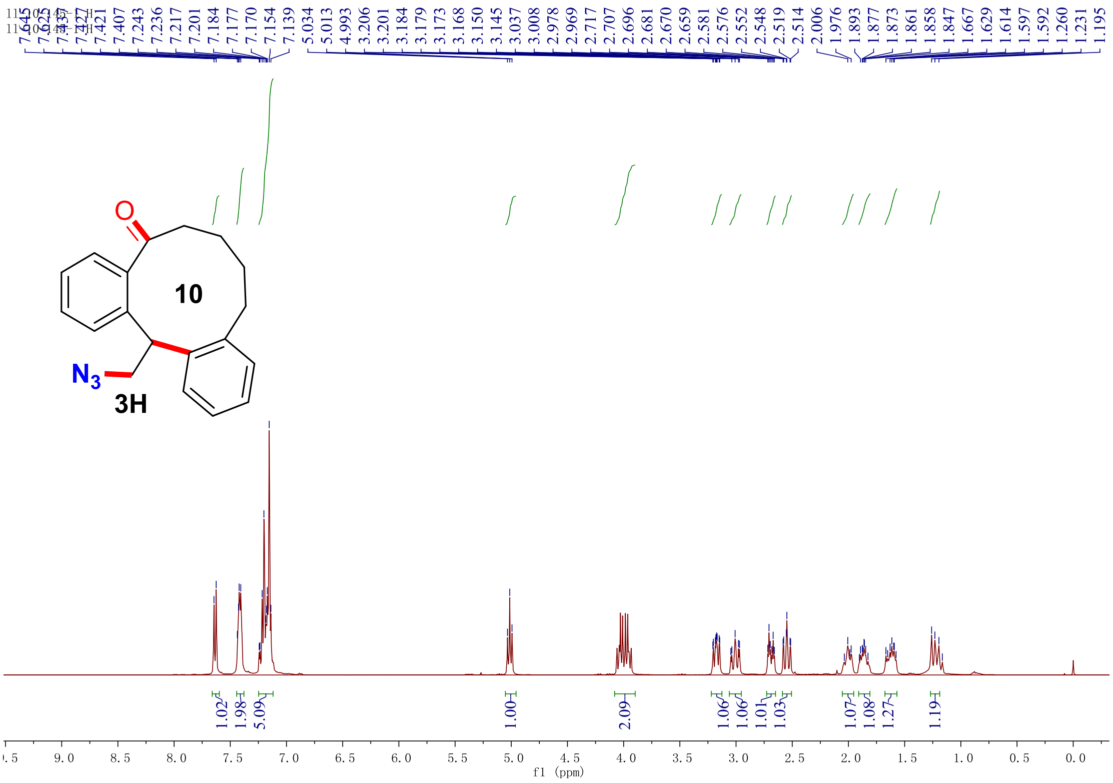

Supplementary Figure 73. ^{1}H NMR of **3H**

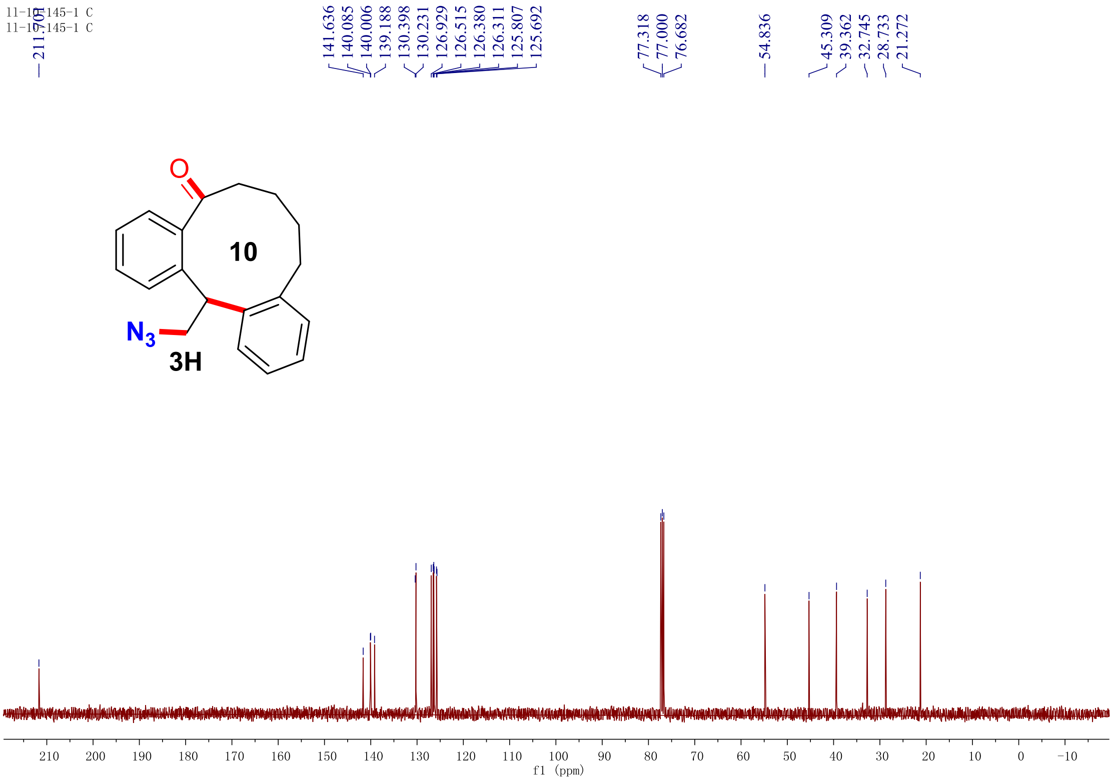

Supplementary Figure 74. ^{13}C NMR of **3H**

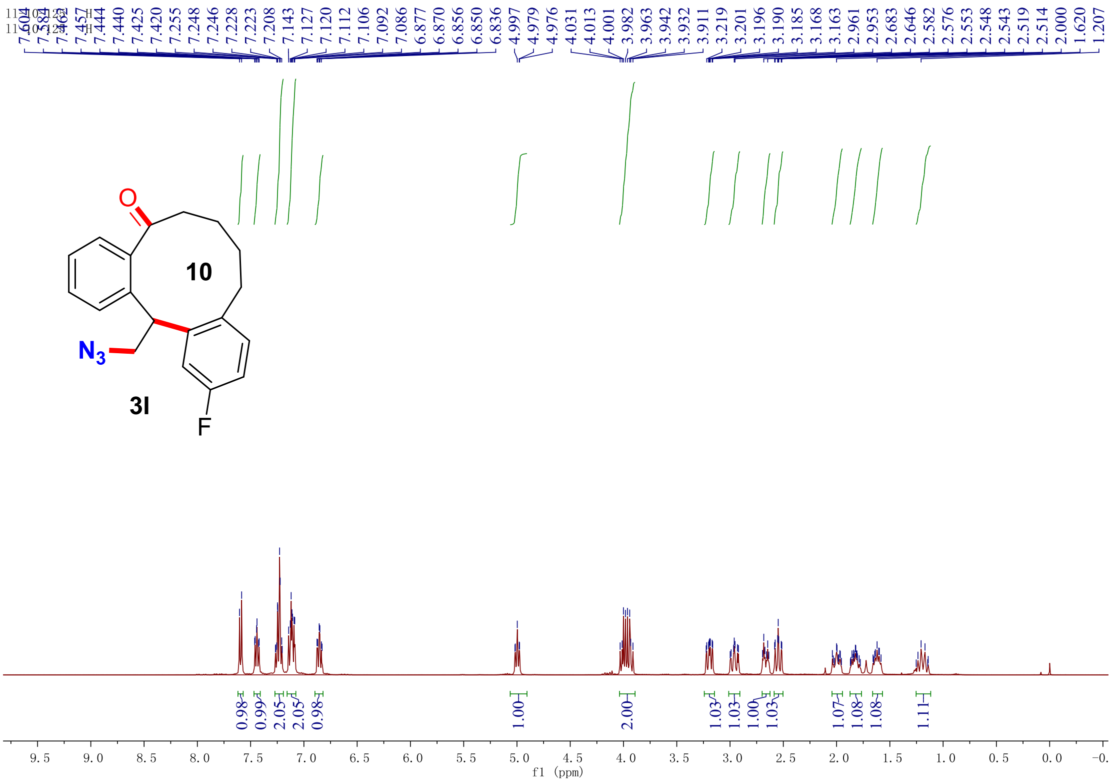

Supplementary Figure 75. ^{1}H NMR of **3l**

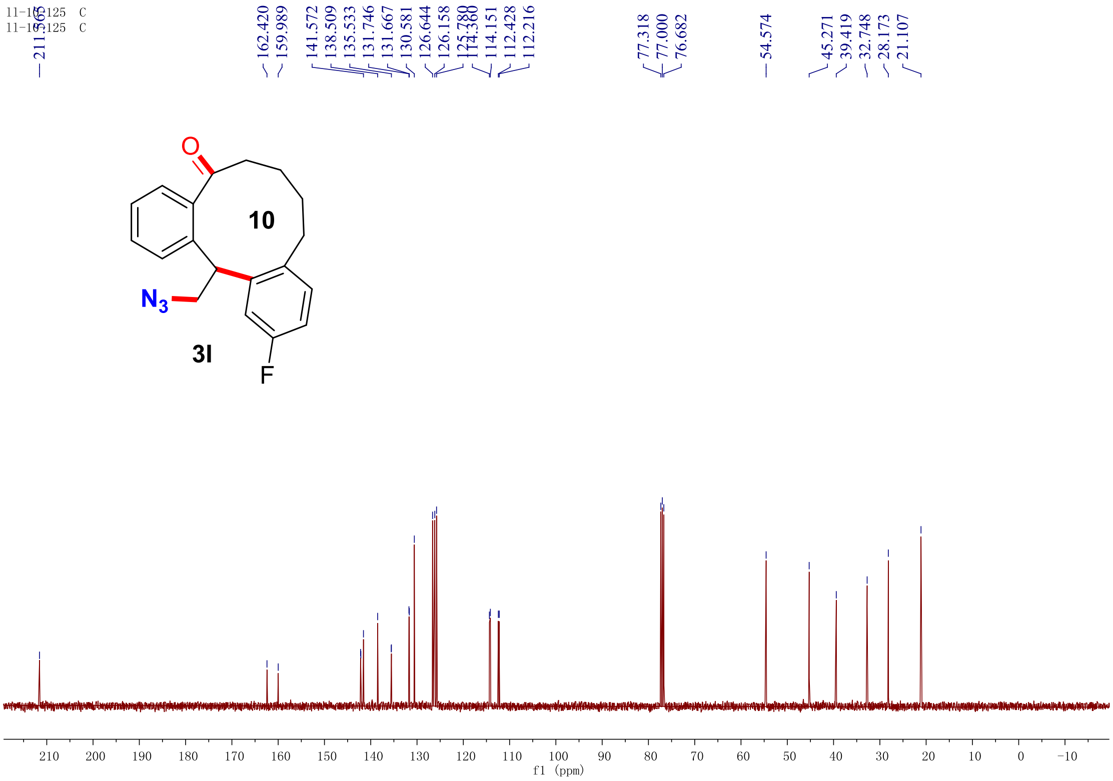

Supplementary Figure 76. ^{13}C NMR of **3l**

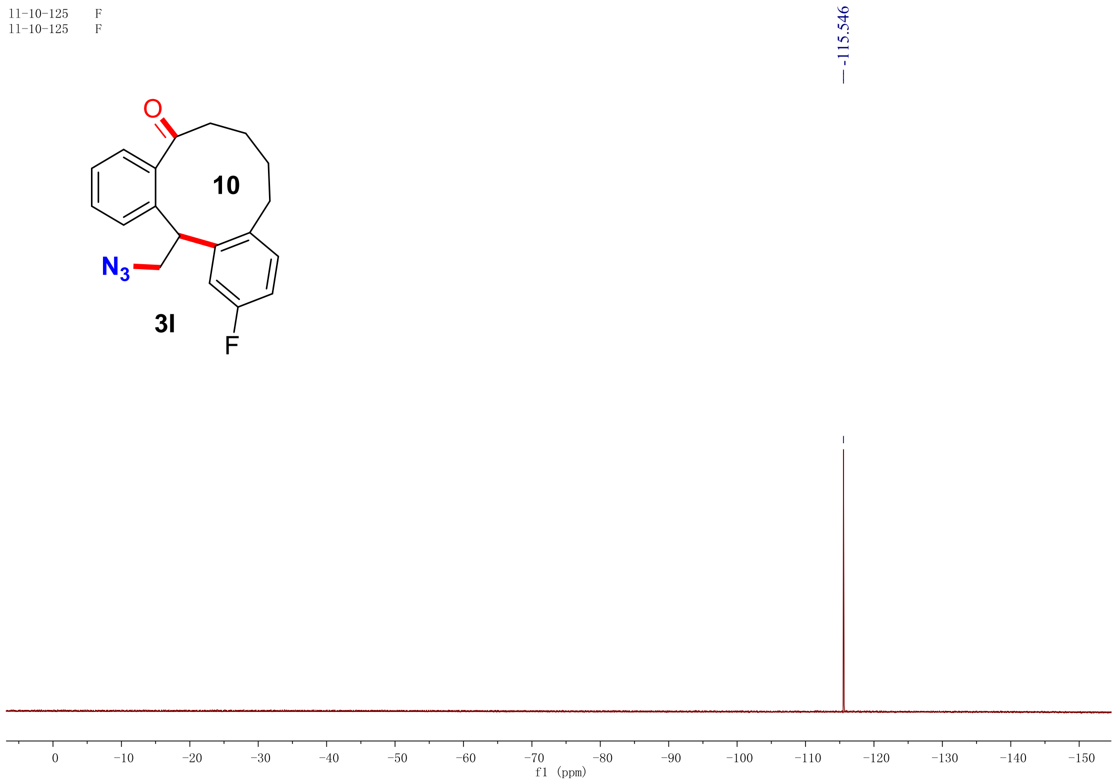

Supplementary Figure 77. ^{19}F NMR of **3I**

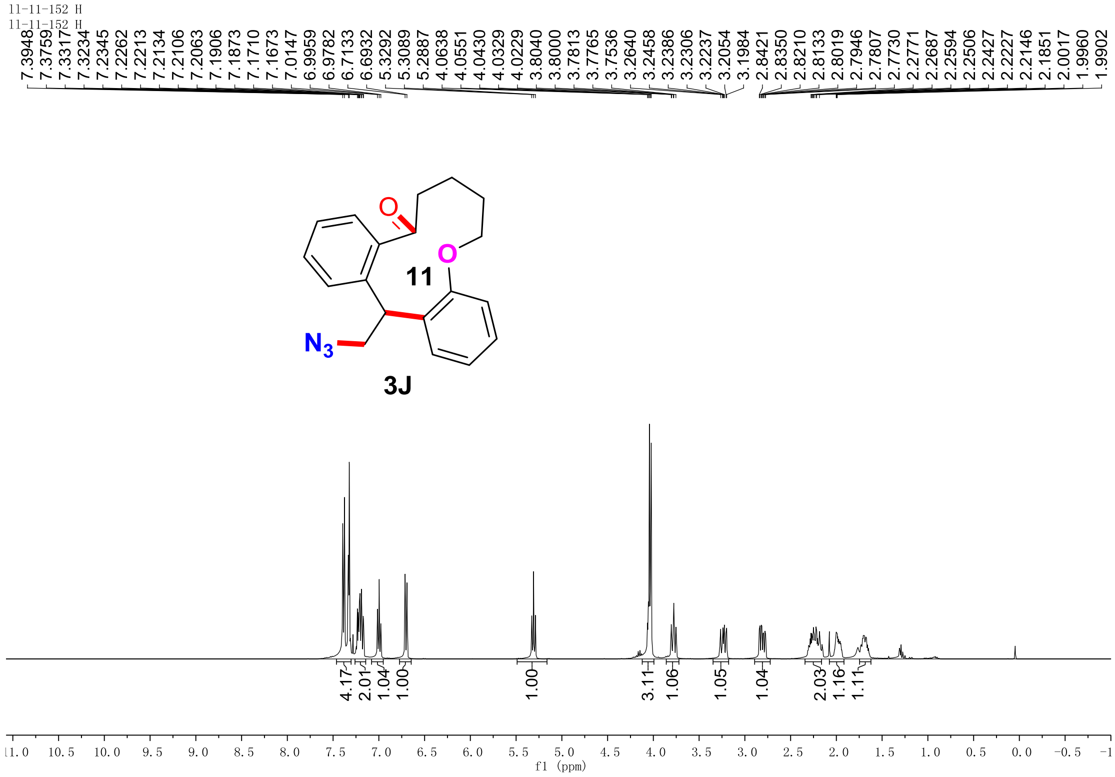

Supplementary Figure 78. ^{1}H NMR of **3J**

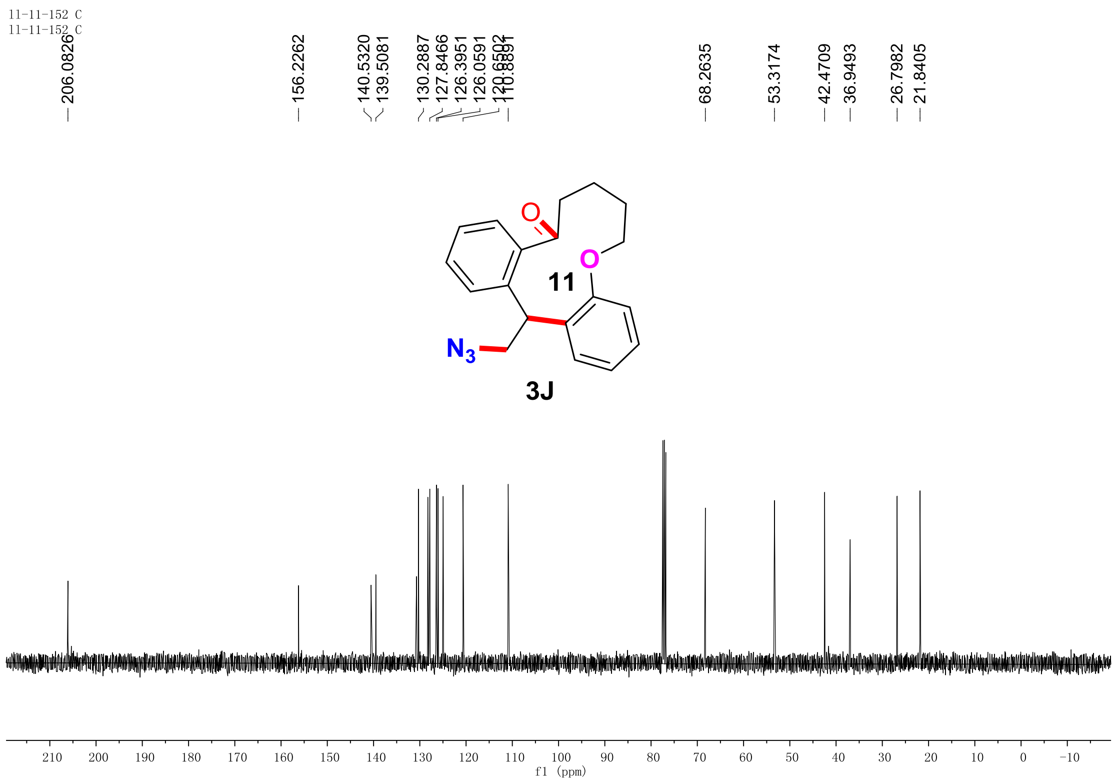

Supplementary Figure 79. ^{13}C NMR of **3J**

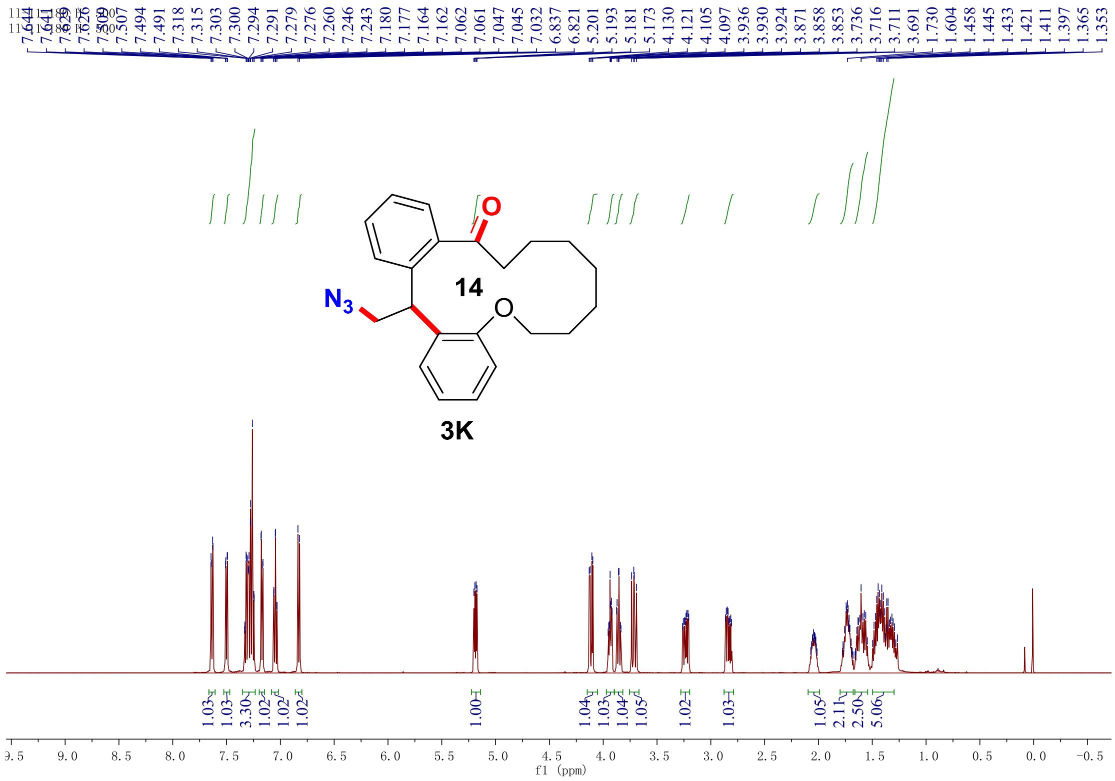

Supplementary Figure 80. ^{1}H NMR of **3K**

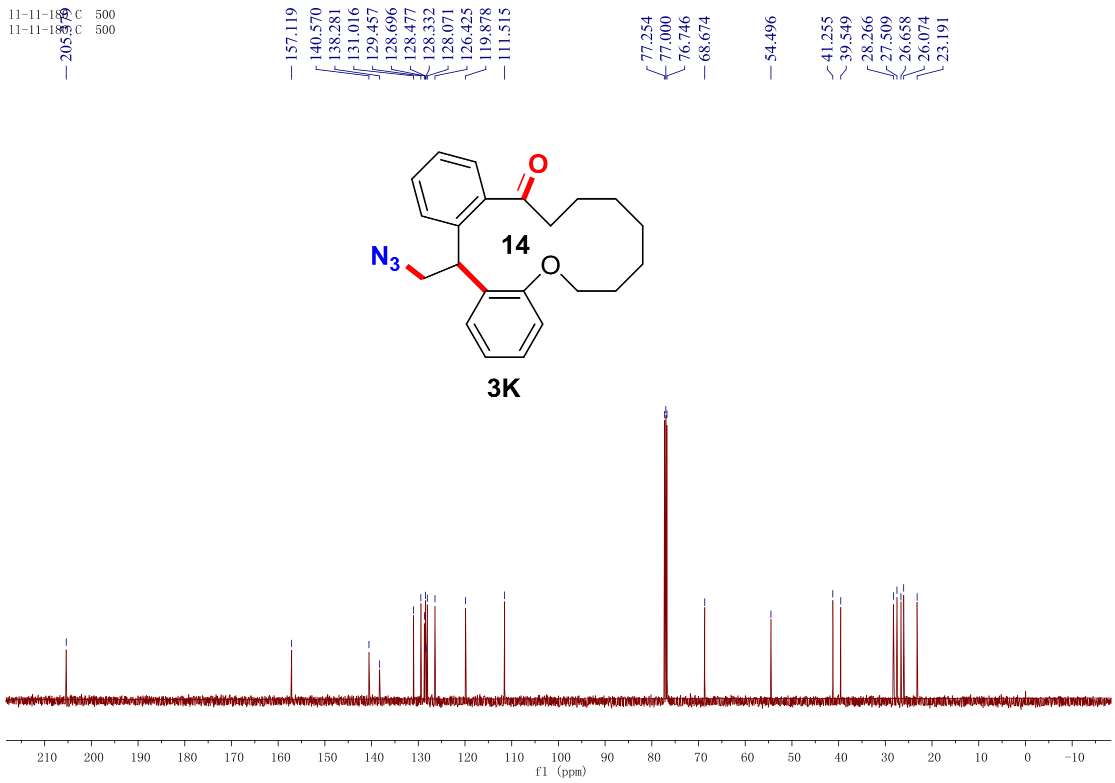

Supplementary Figure 81. ^{13}C NMR of **3K**

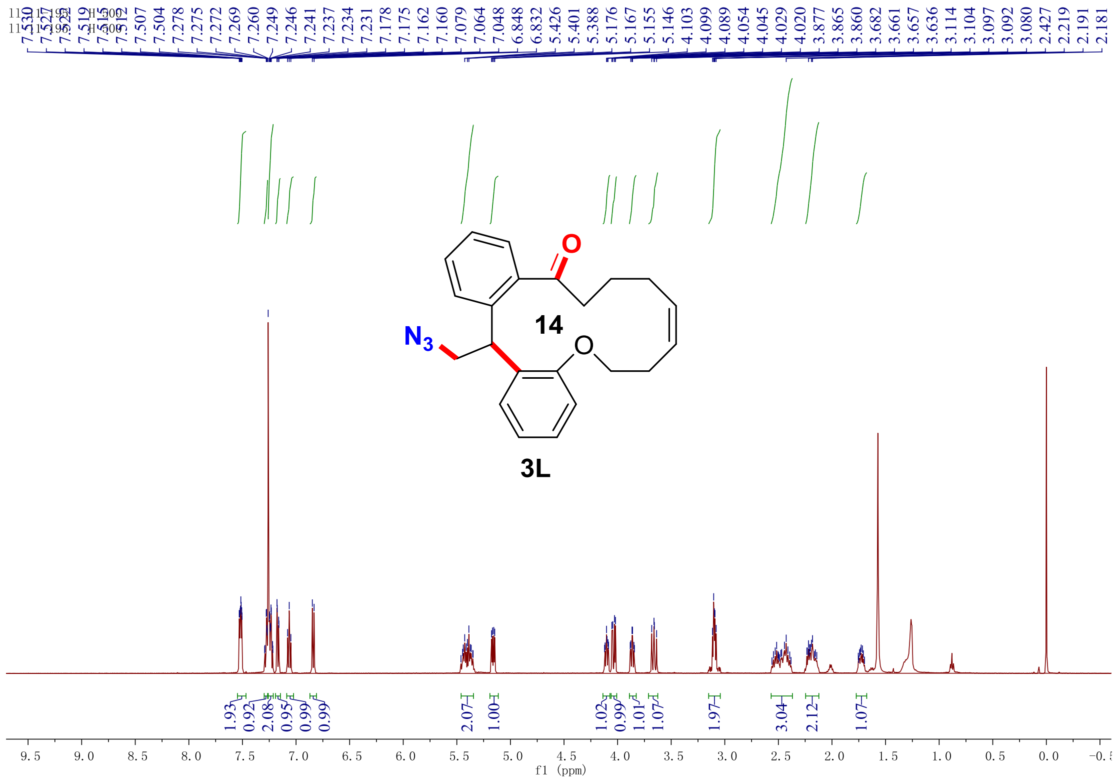

Supplementary Figure 82. ^{1}H NMR of **3L**

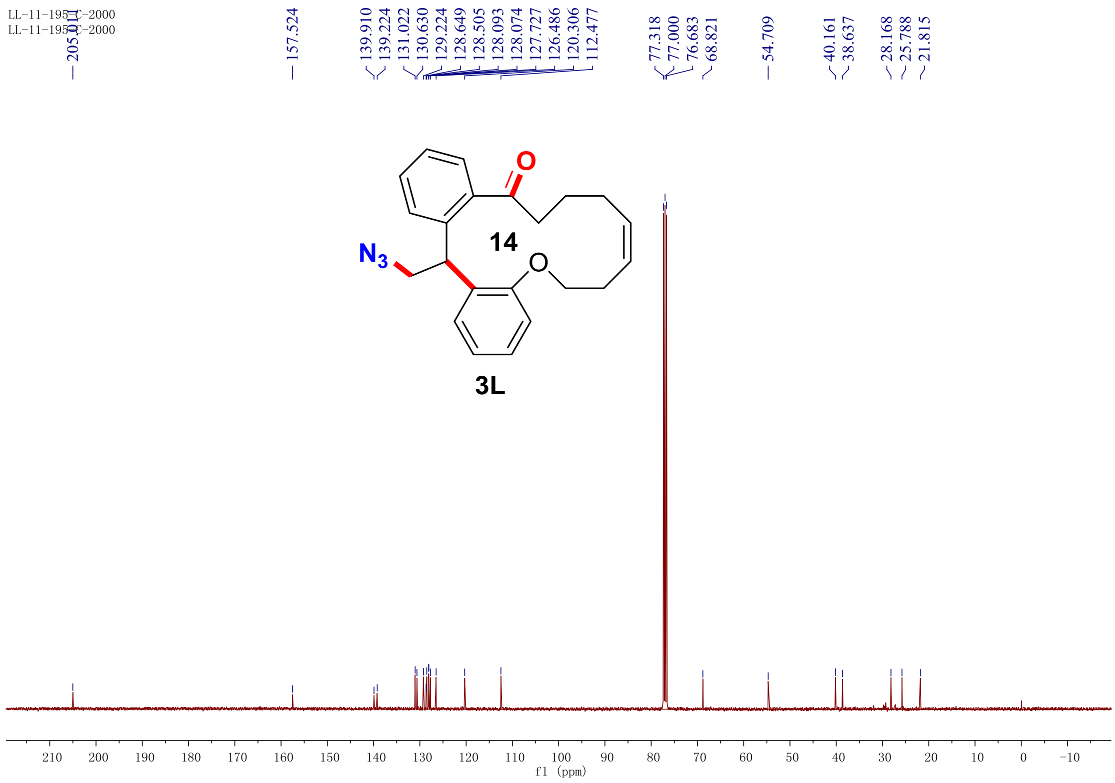

Supplementary Figure 83. ^{13}C NMR of **3L**

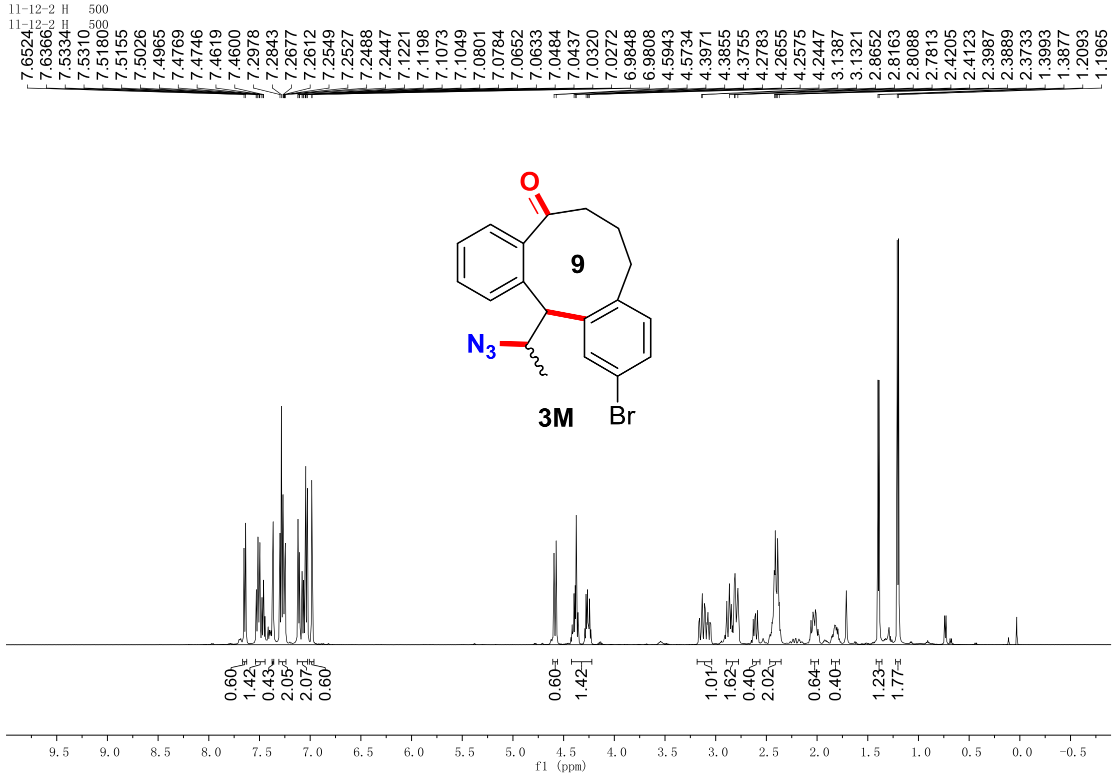

Supplementary Figure 84. ^{1}H NMR of **3M**

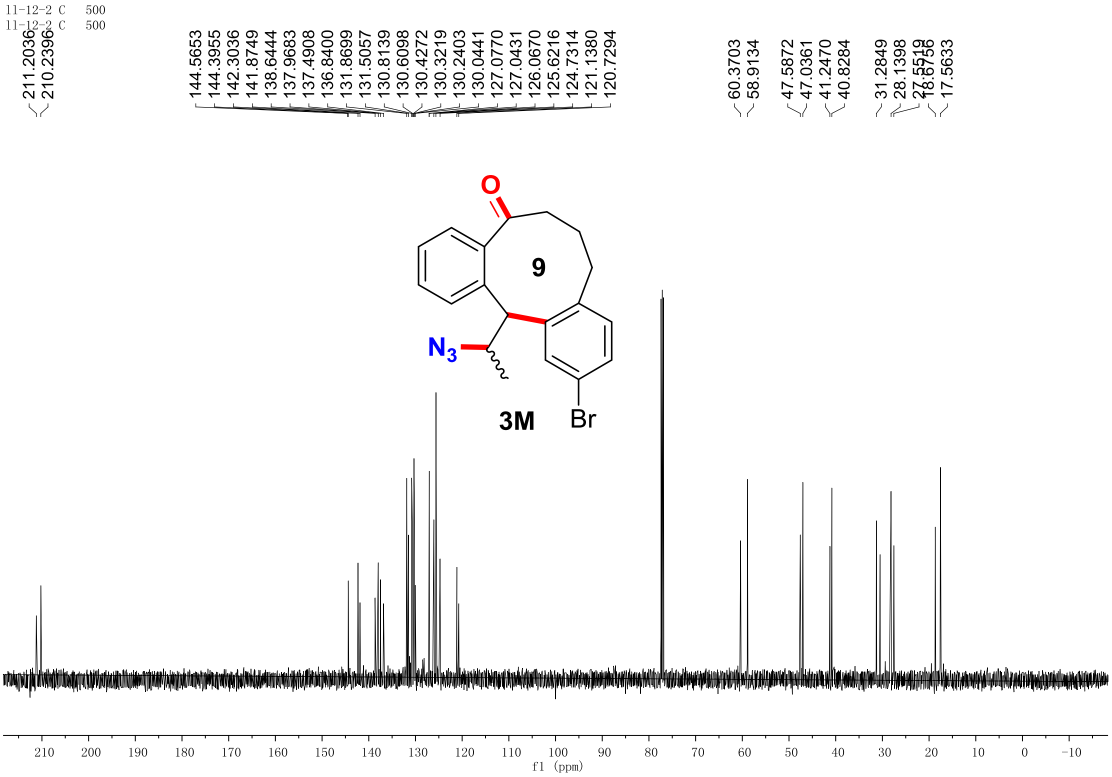

Supplementary Figure 85. ^{13}C NMR of **3M**

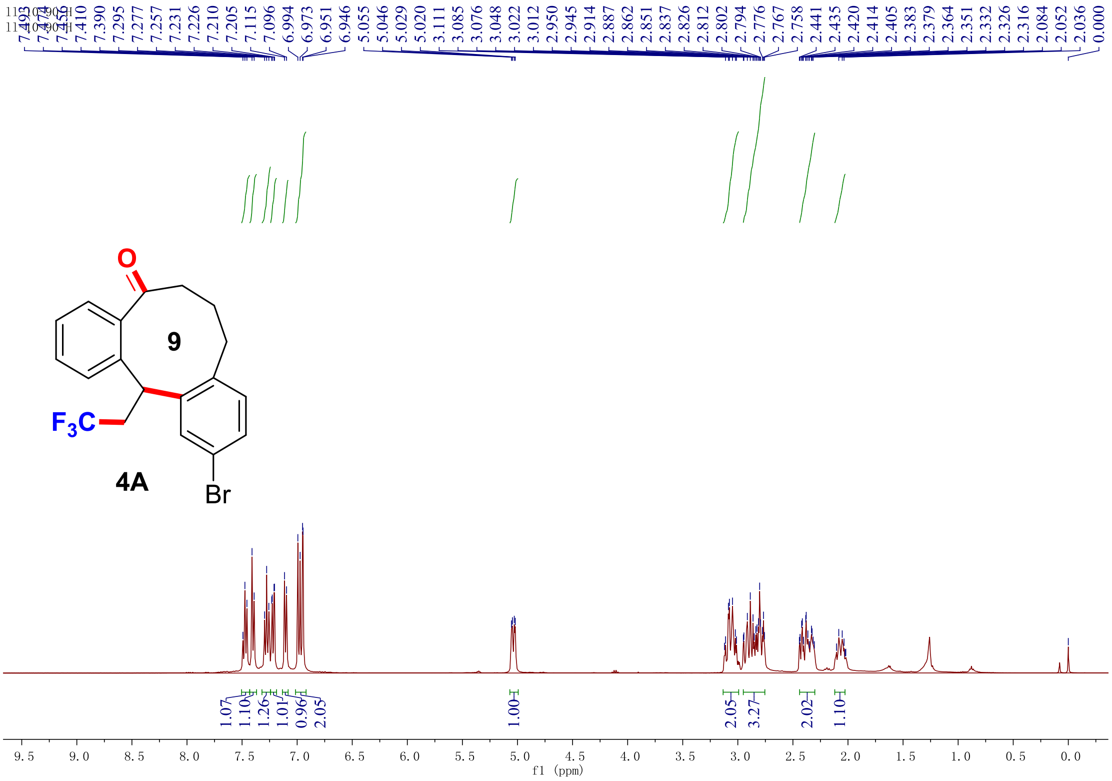

Supplementary Figure 86. ^{1}H NMR of **4A**

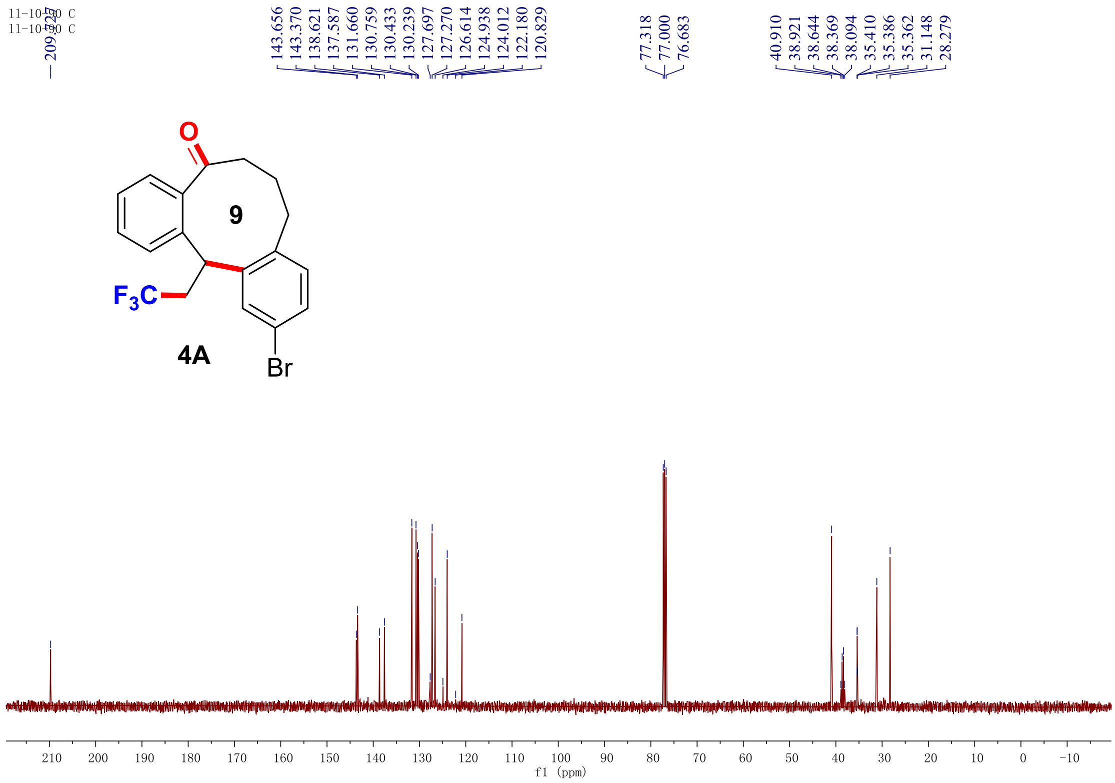

Supplementary Figure 87. ^{13}C NMR of **4A**

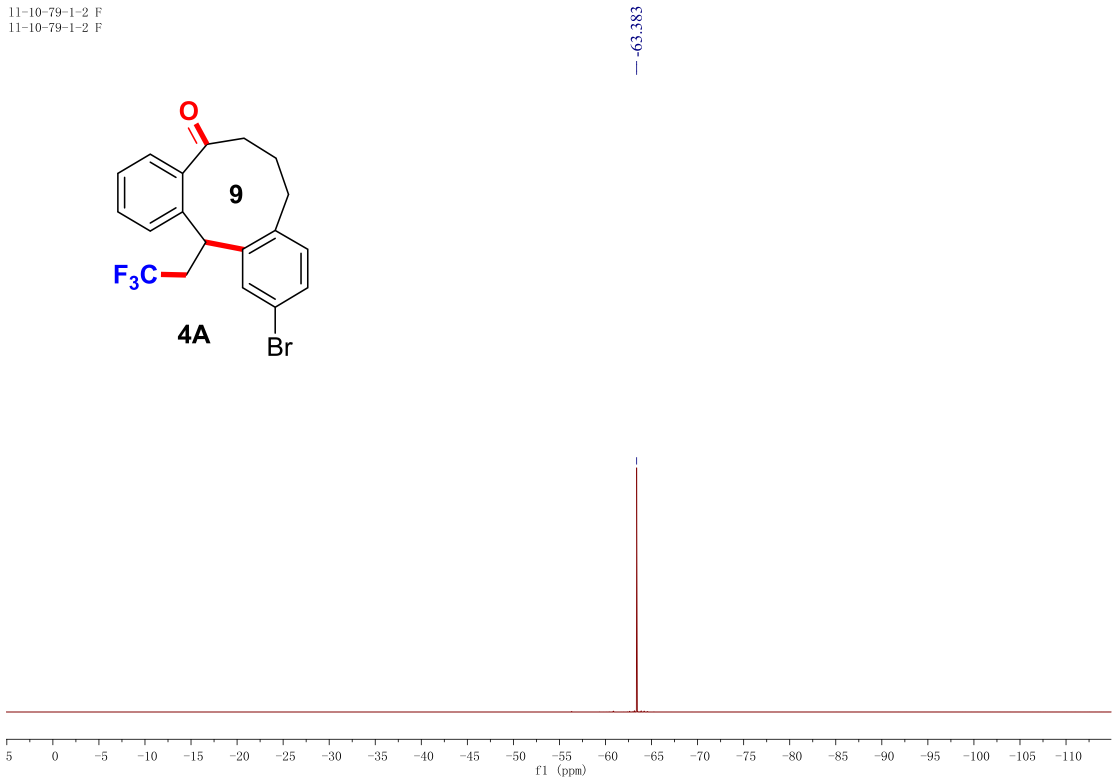

Supplementary Figure 88. ^{19}F NMR of **4A**

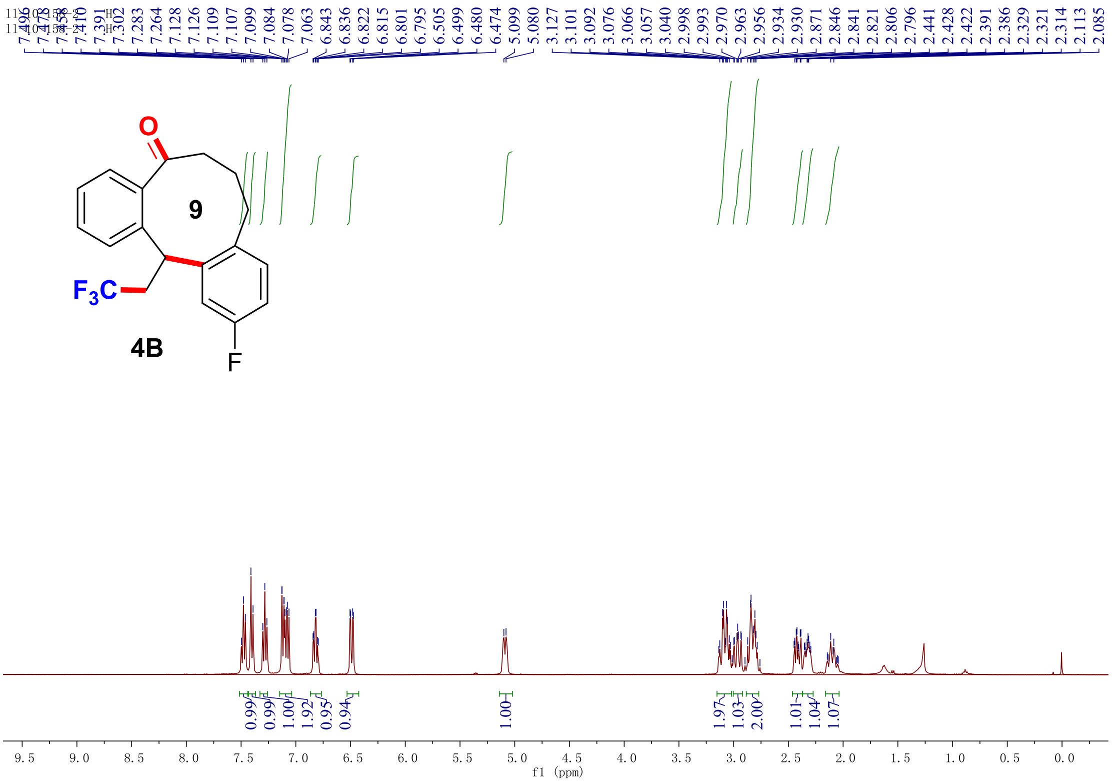

Supplementary Figure 89. ^{1}H NMR of **4B**

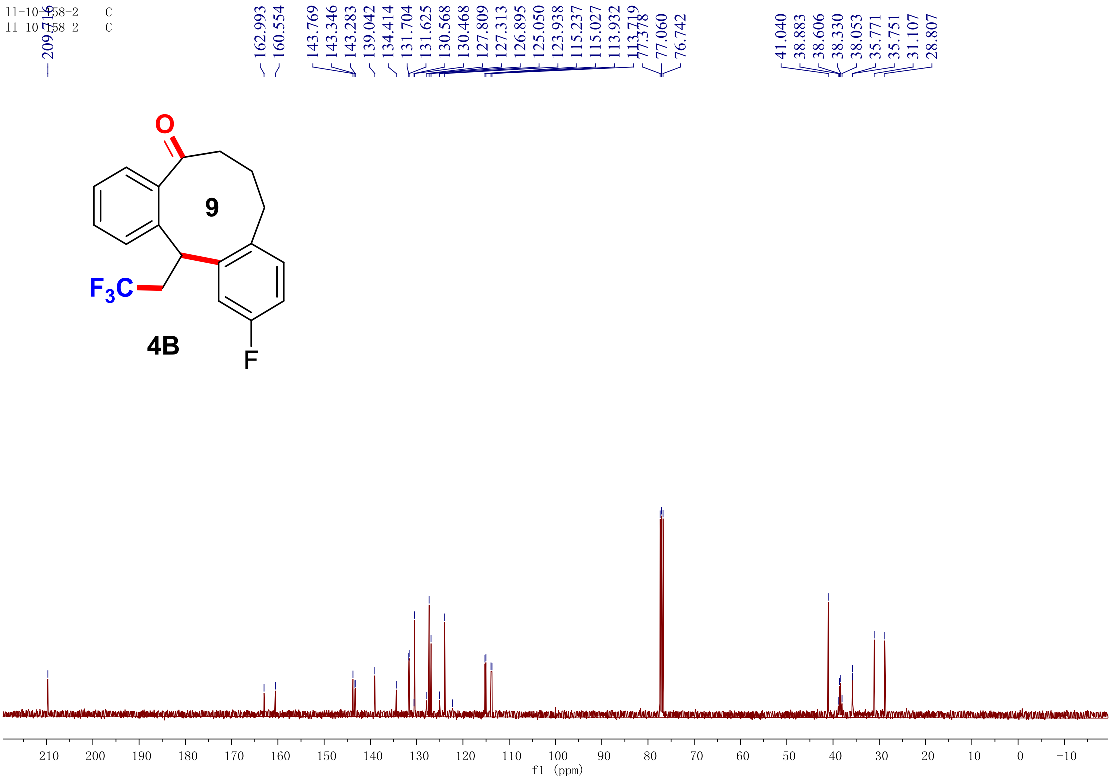

Supplementary Figure 90. ^{13}C NMR of **4B**

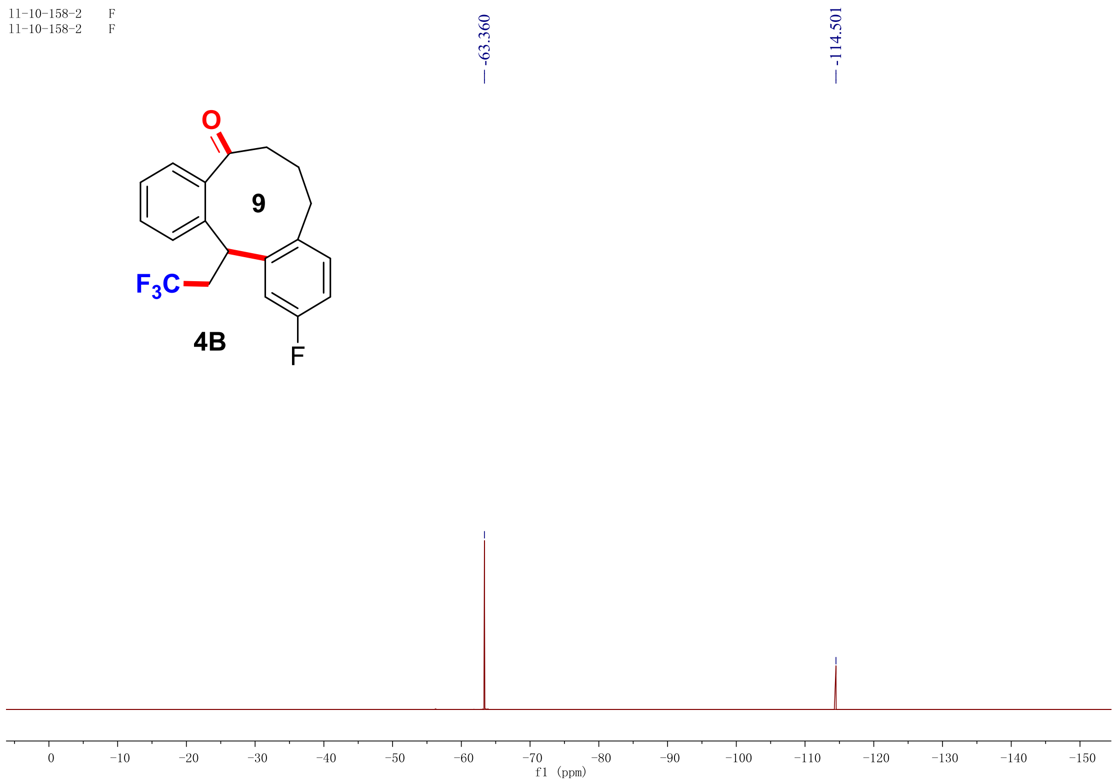

Supplementary Figure 91. ^{19}F NMR of **4B**

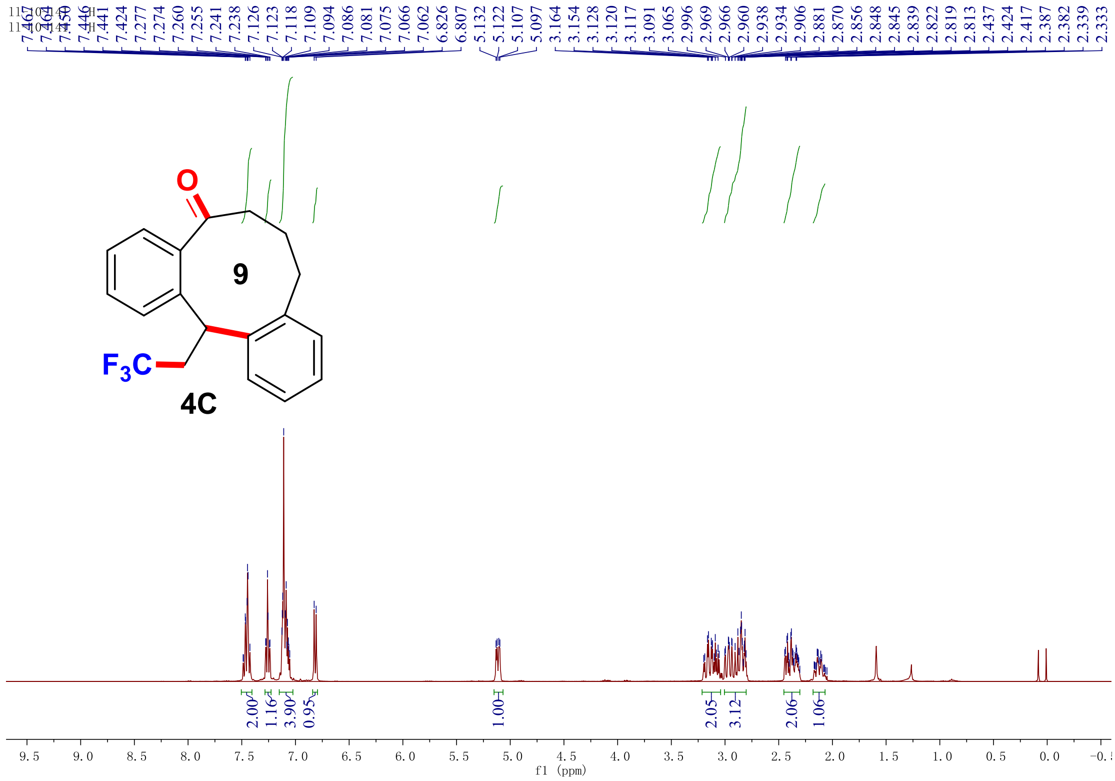

Supplementary Figure 92. ^{1}H NMR of **4C**

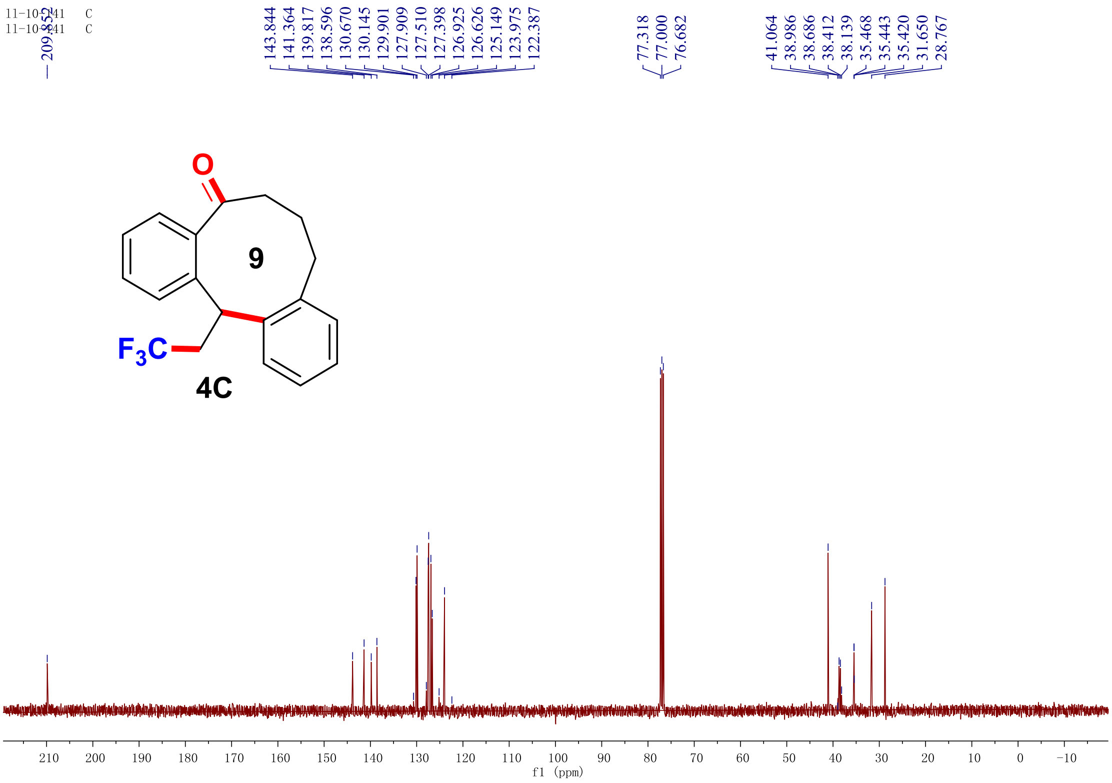

Supplementary Figure 93. ^{13}C NMR of **4C**

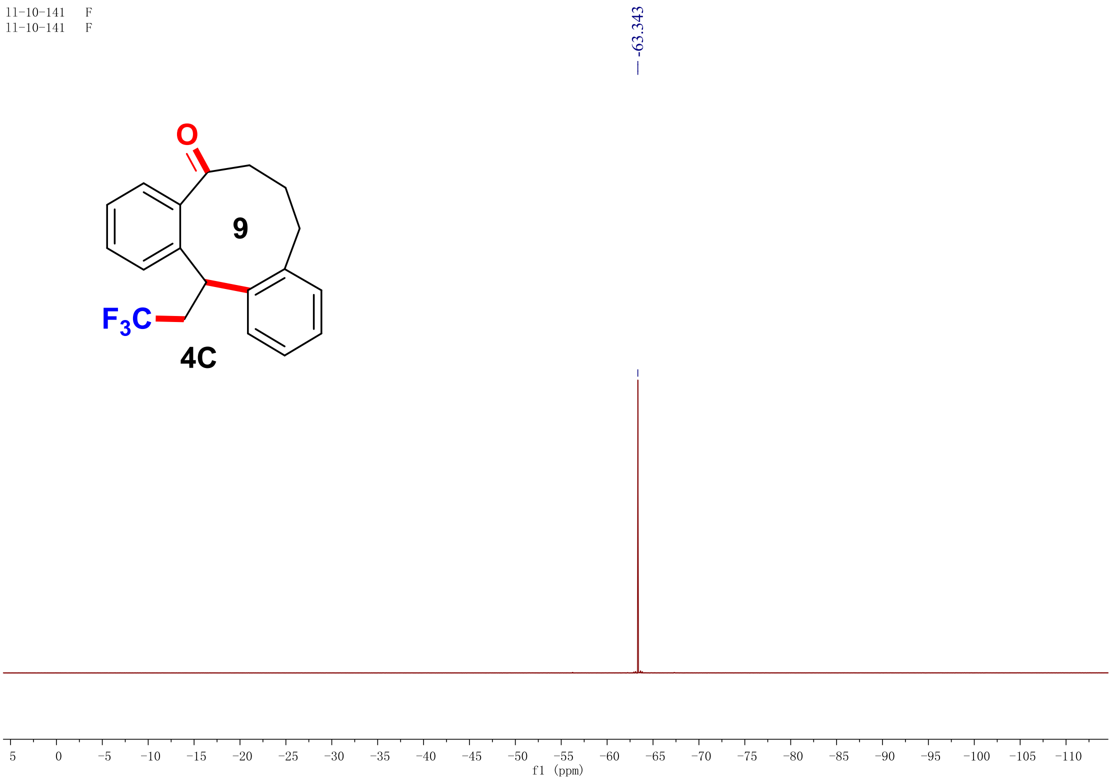

Supplementary Figure 94. ^{19}F NMR of **4C**

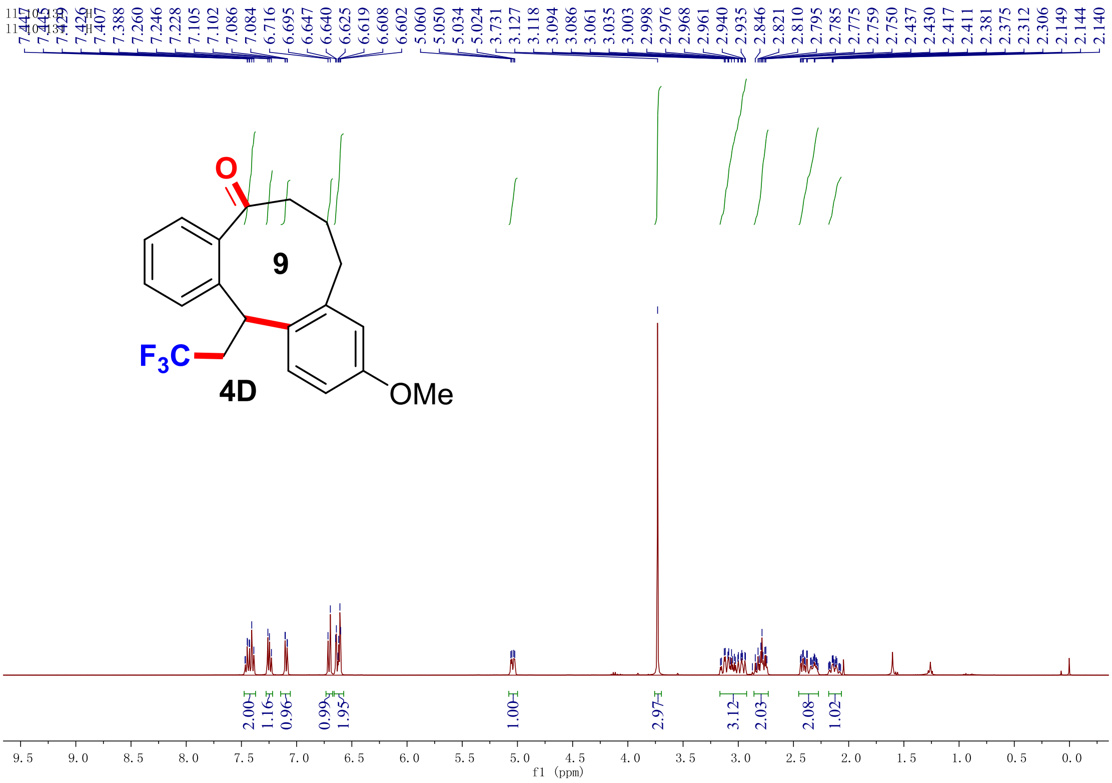

Supplementary Figure 95. ^{1}H NMR of **4D**

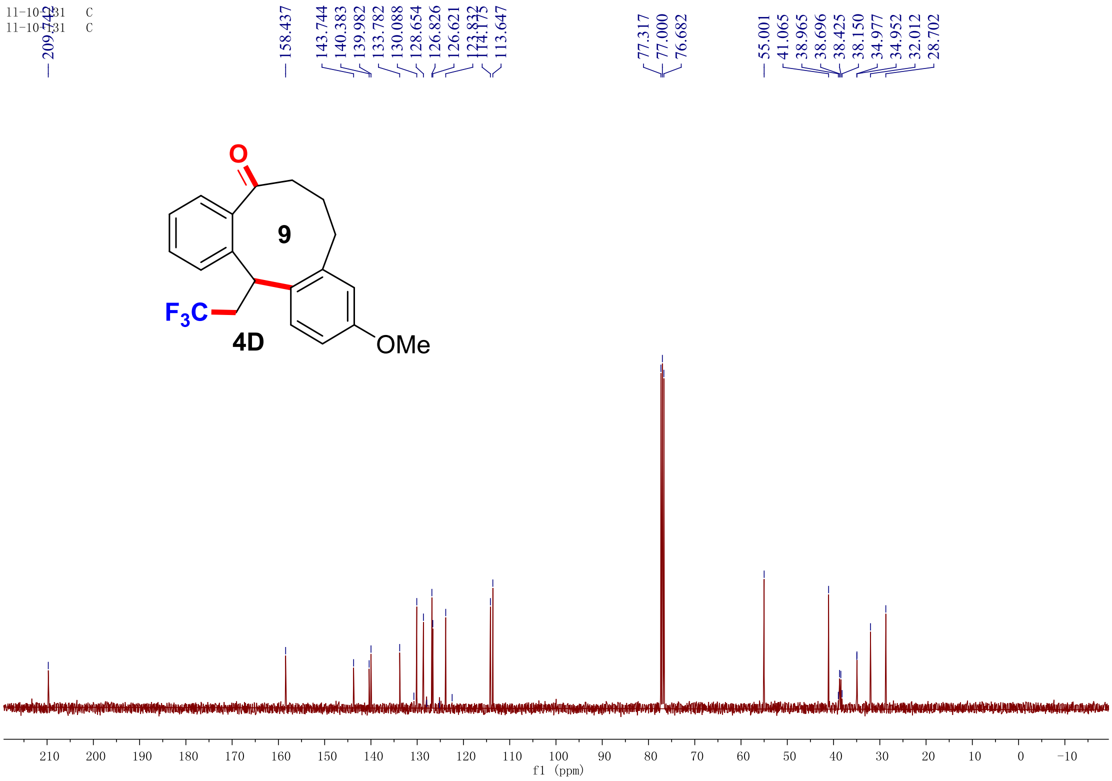

Supplementary Figure 96. ^{13}C NMR of **4D**

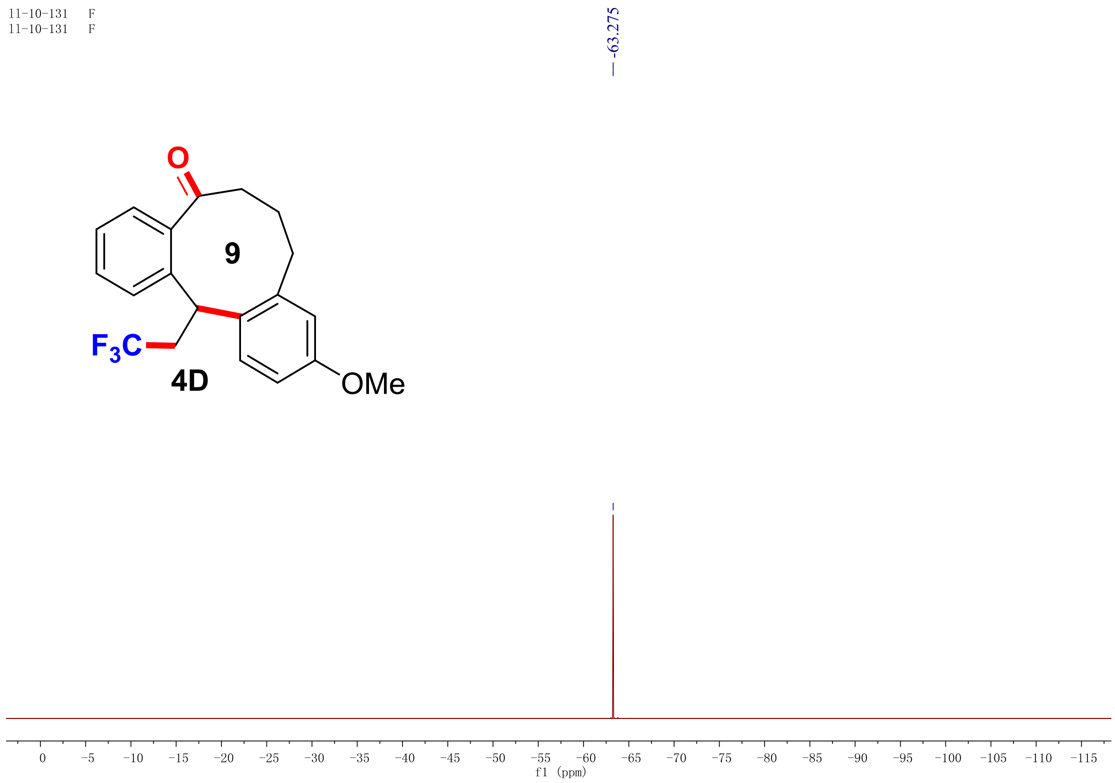

Supplementary Figure 97. ^{19}F NMR of **4D**

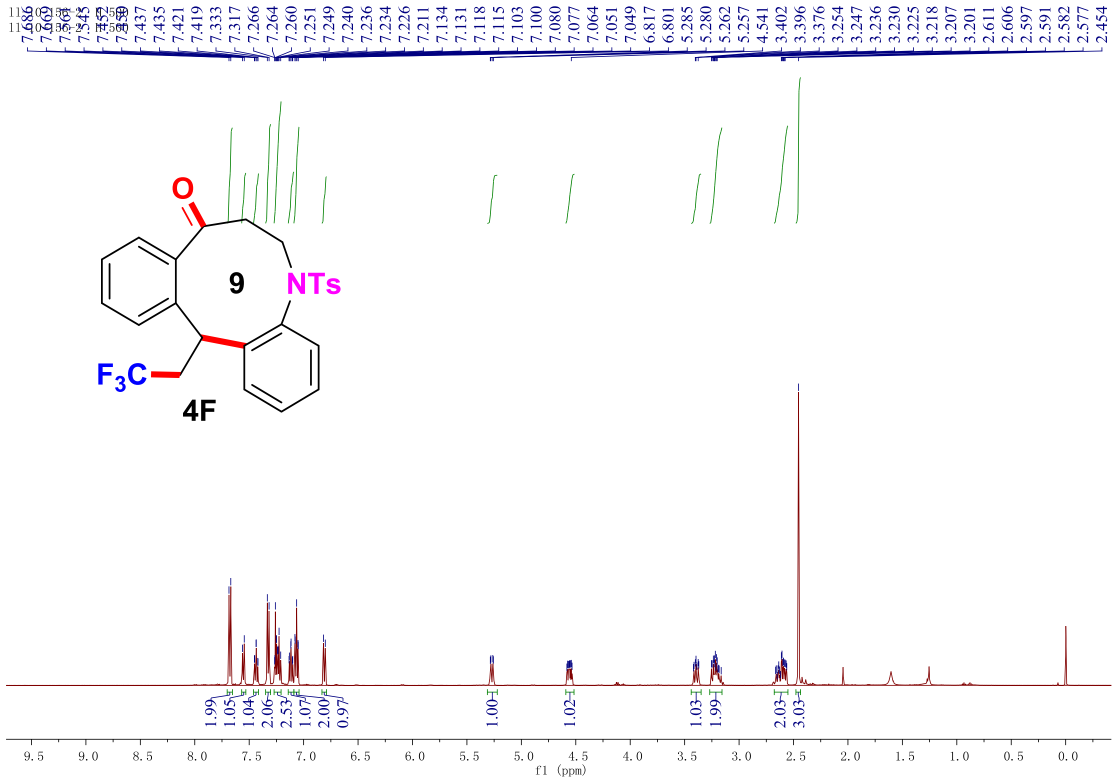

Supplementary Figure 98. ^{1}H NMR of **4F**

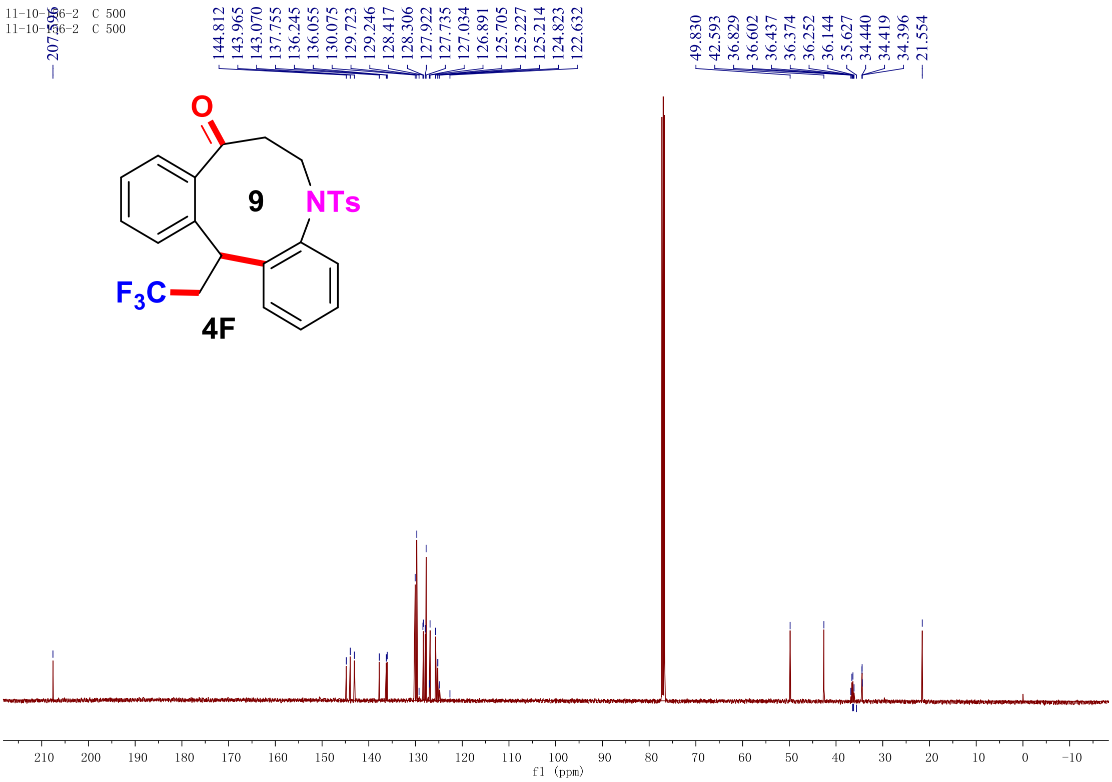

Supplementary Figure 99. ^{13}C NMR of **4F**

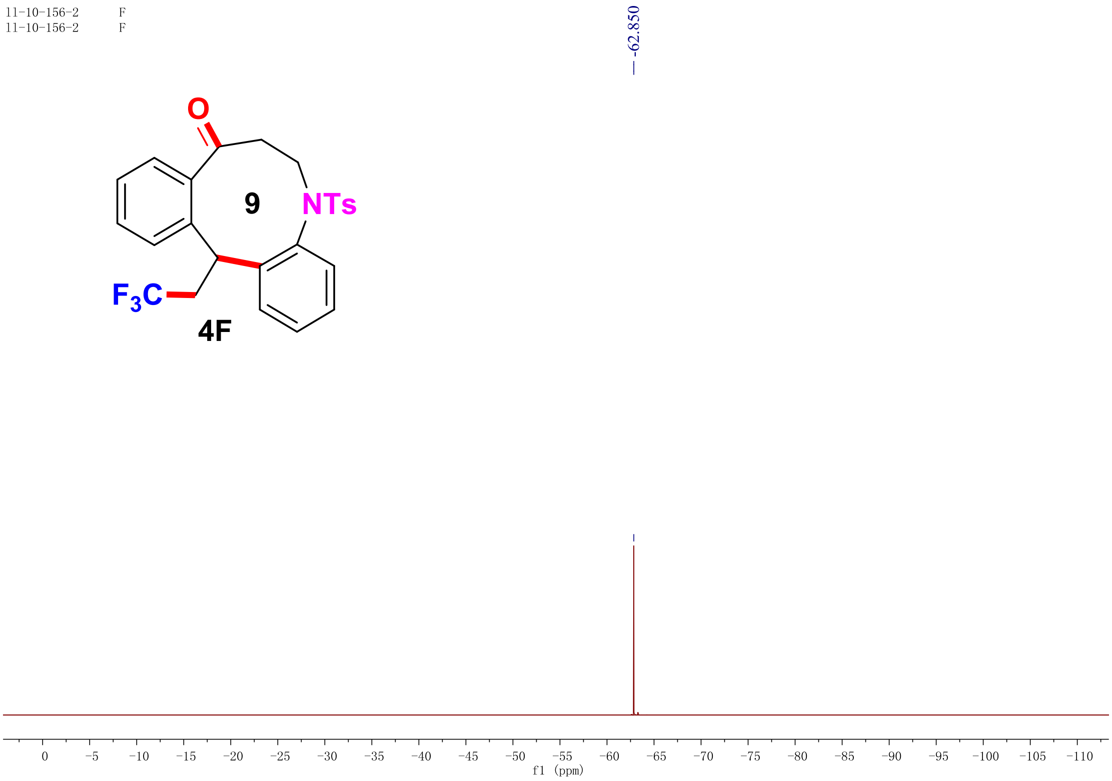

Supplementary Figure 100. ^{19}F NMR of **4F**

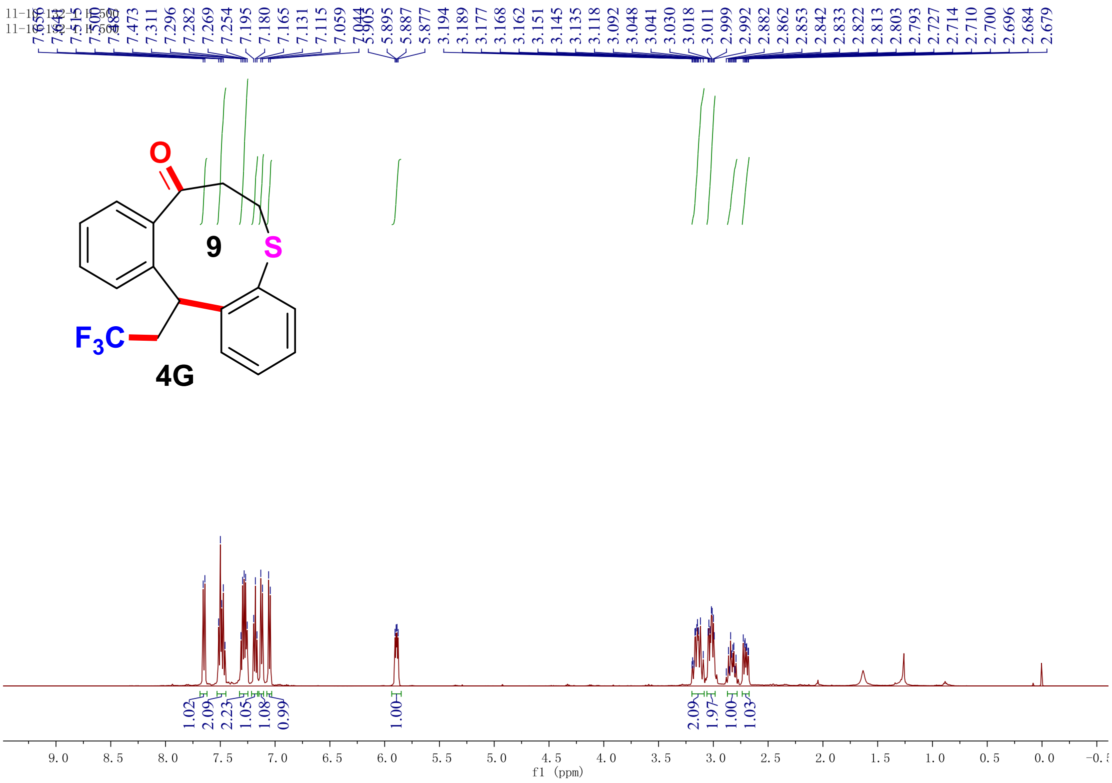

Supplementary Figure 101. ^{1}H NMR of **4G**

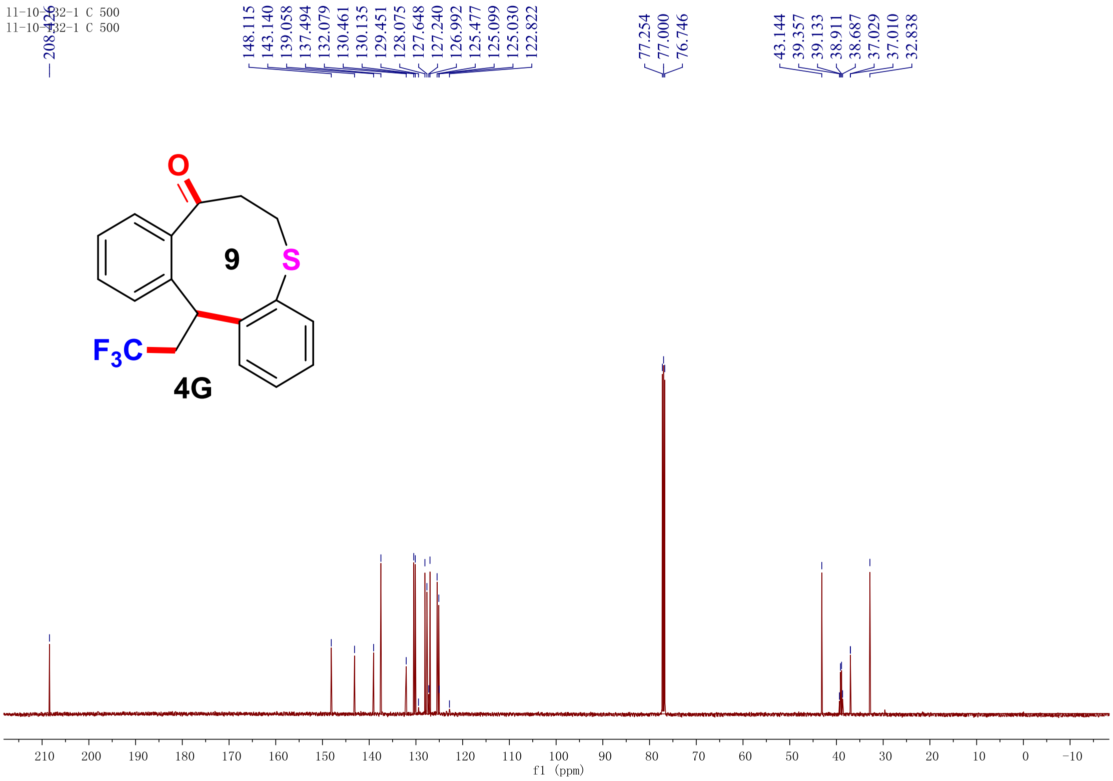

Supplementary Figure 102. ^{13}C NMR of **4G**

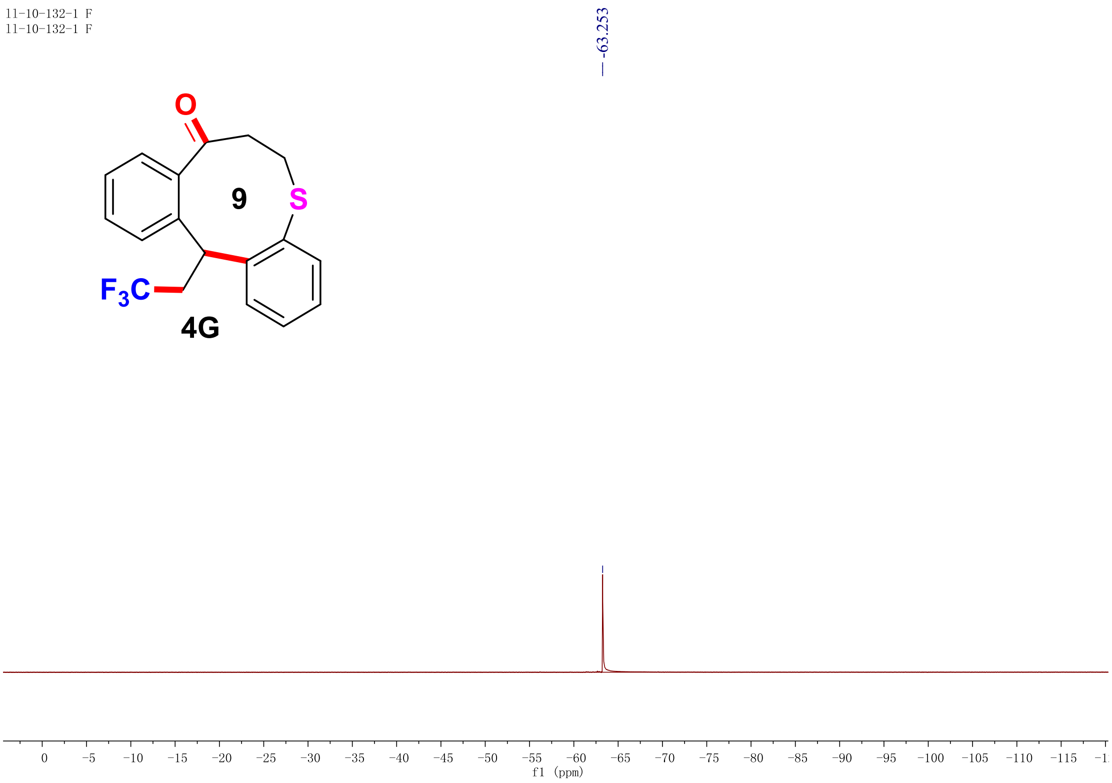

Supplementary Figure 103. ^{19}F NMR of **4G**

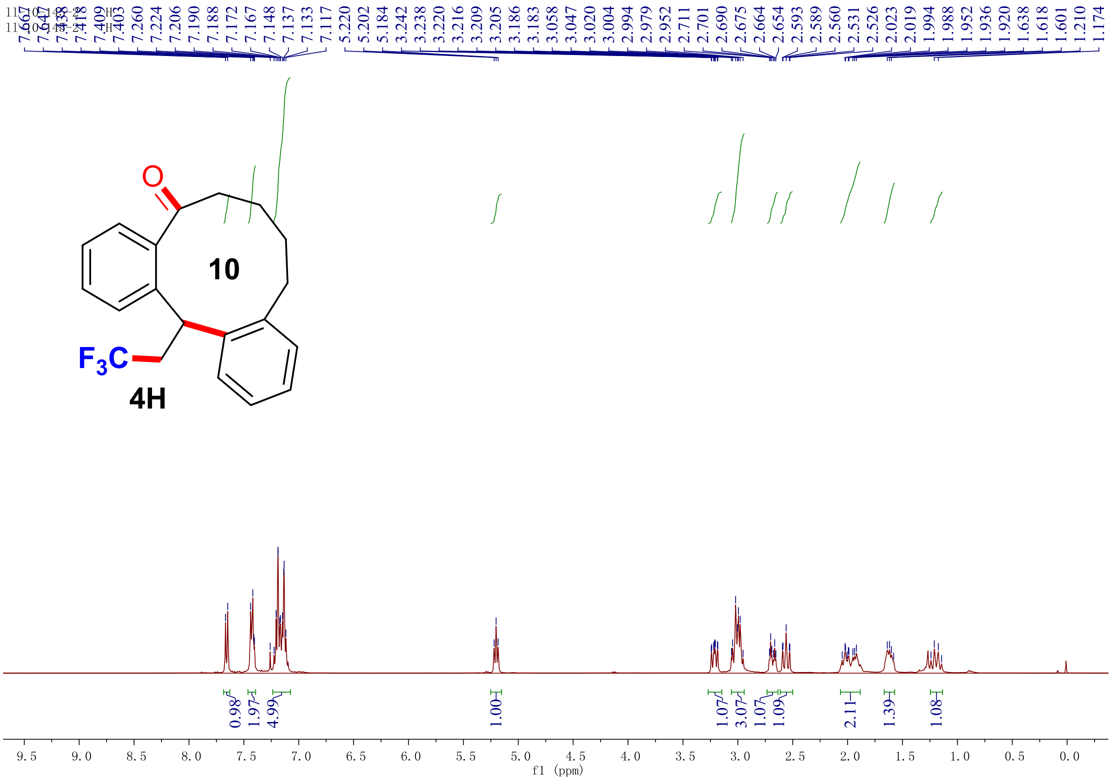

Supplementary Figure 104. ^{1}H NMR of **4H**

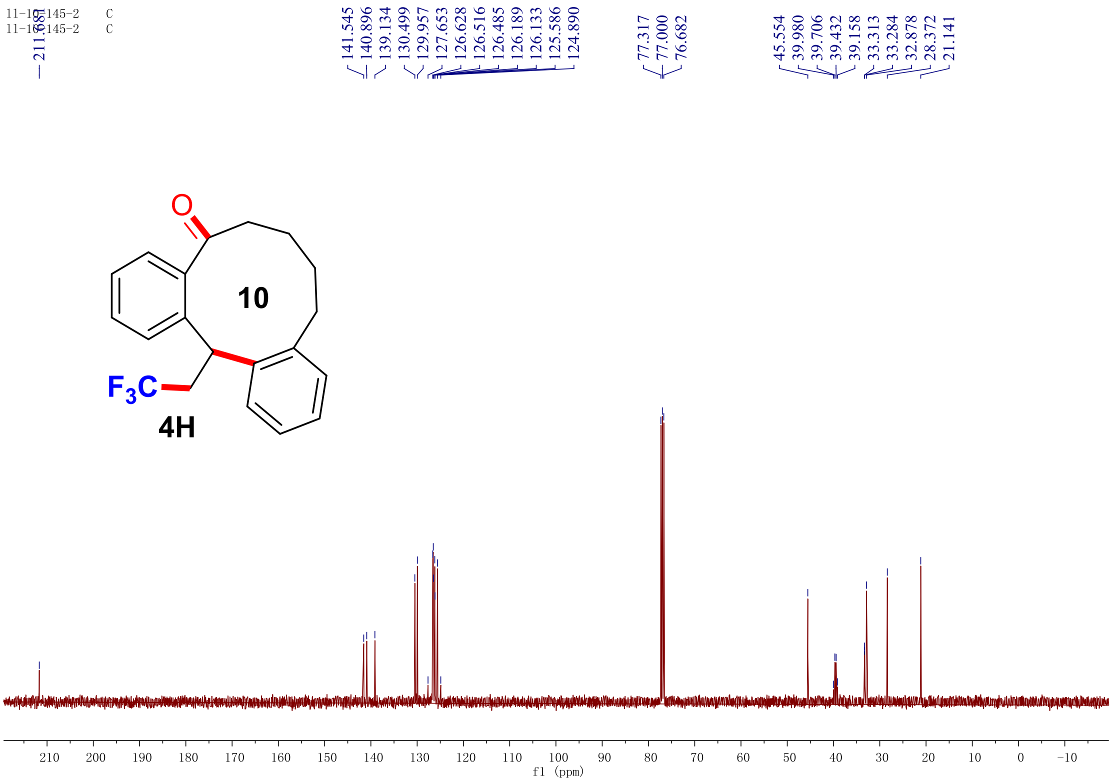

Supplementary Figure 105. ^{13}C NMR of **4H**

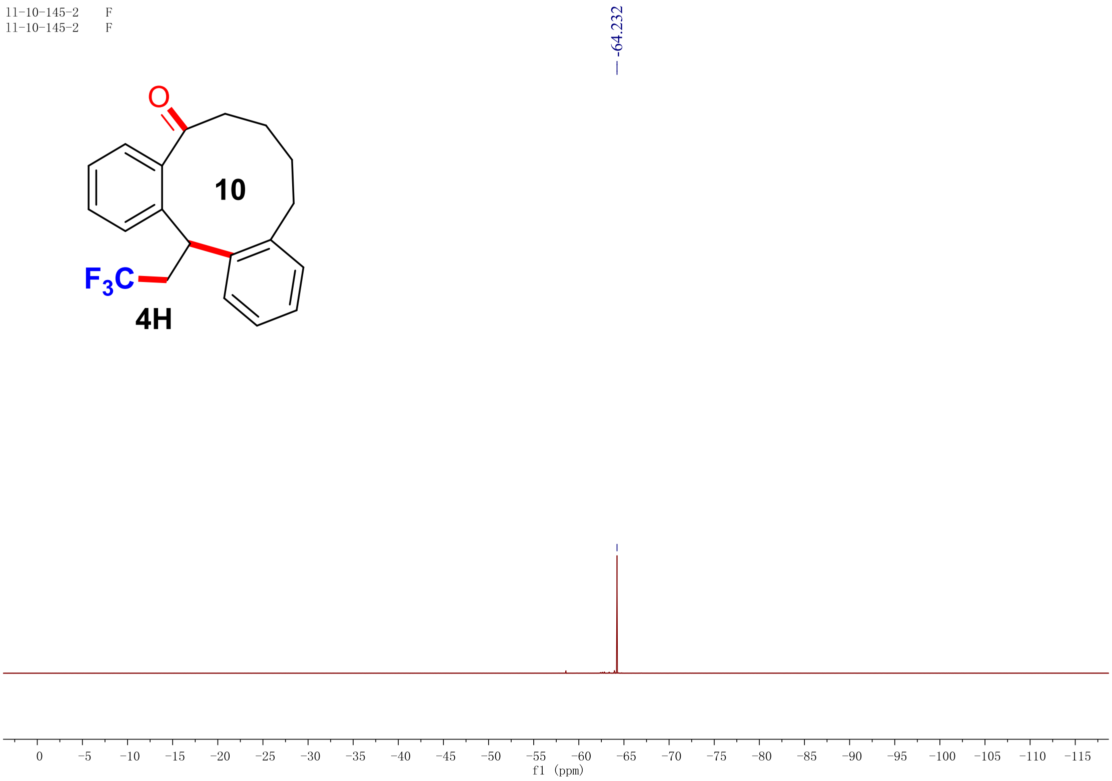

Supplementary Figure 106. ^{19}F NMR of **4H**

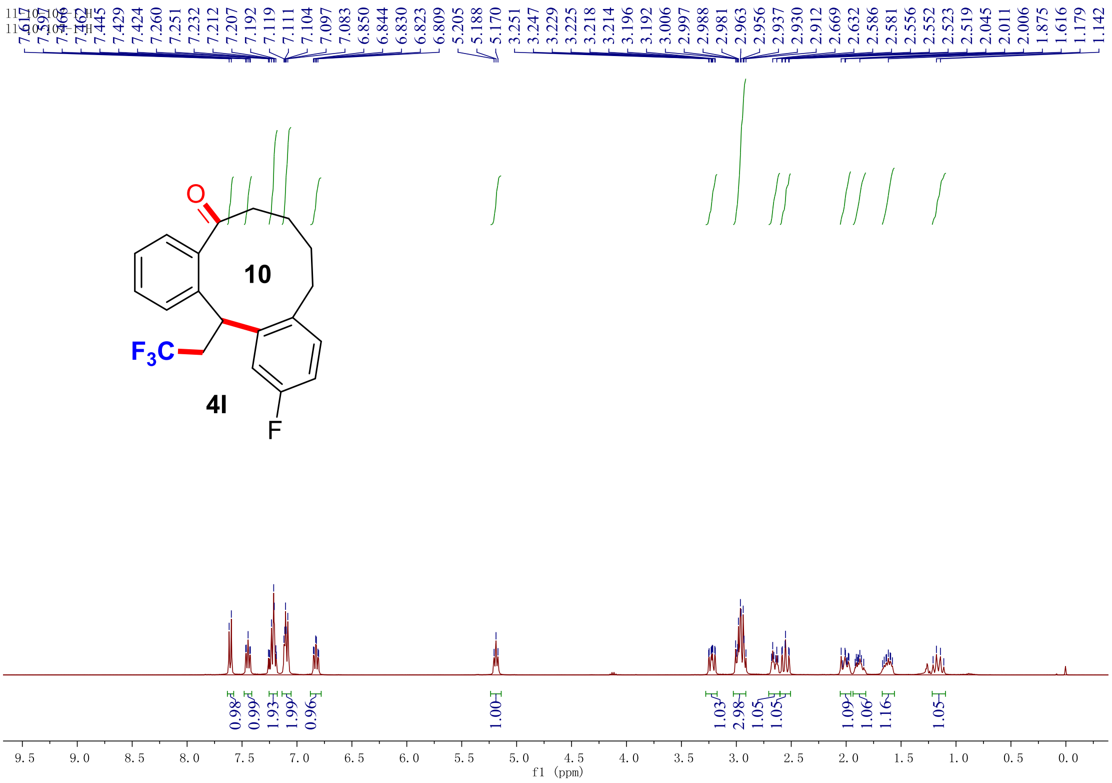

Supplementary Figure 107. ^{1}H NMR of **4l**

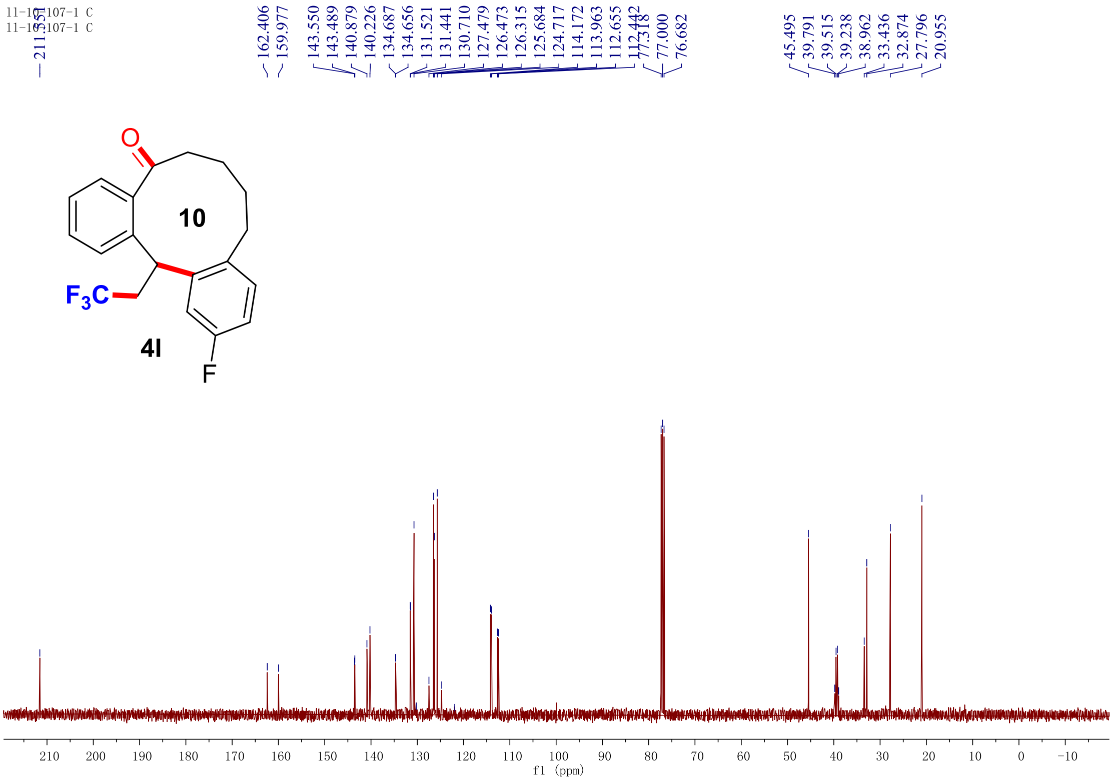

Supplementary Figure 108. ^{13}C NMR of **4l**

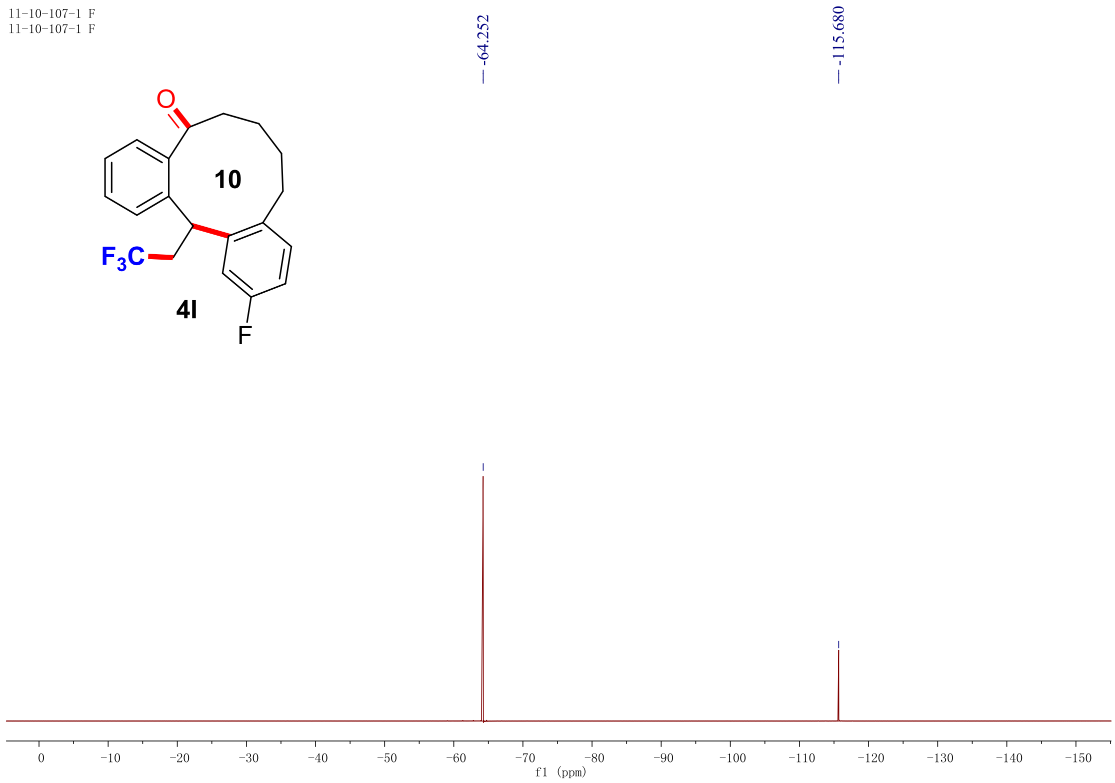

Supplementary Figure 109. ^{19}F NMR of **4I**

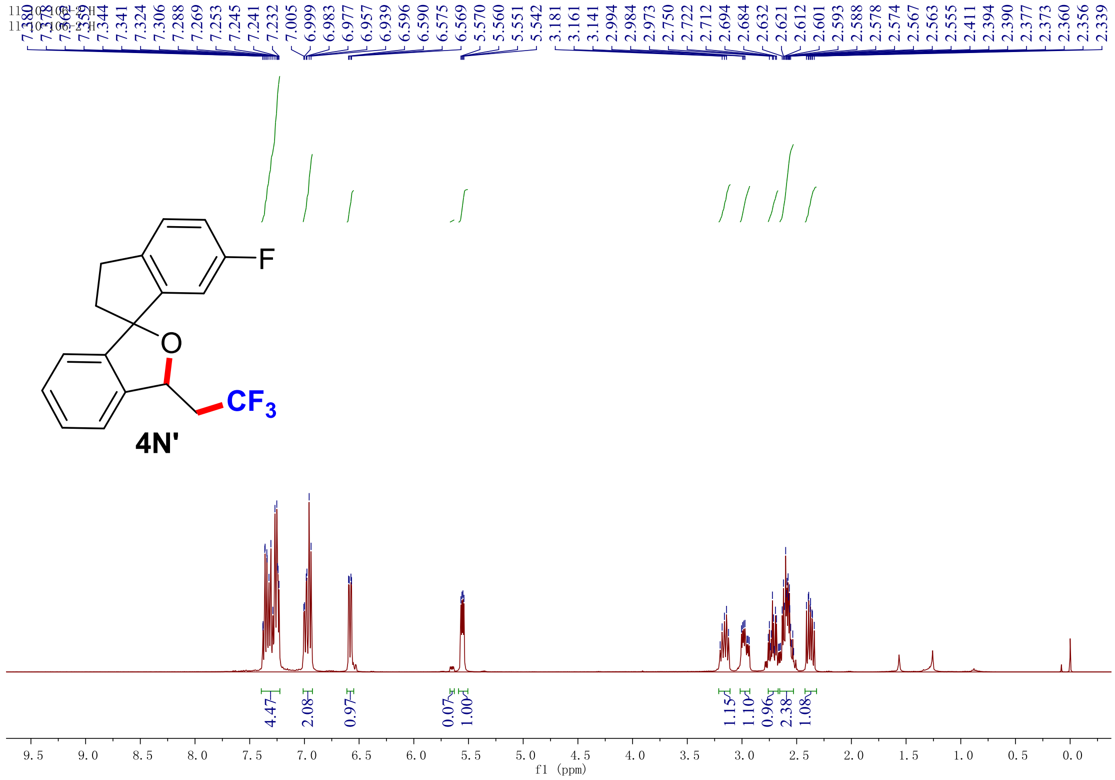

Supplementary Figure 110. ^{1}H NMR of **4N'**

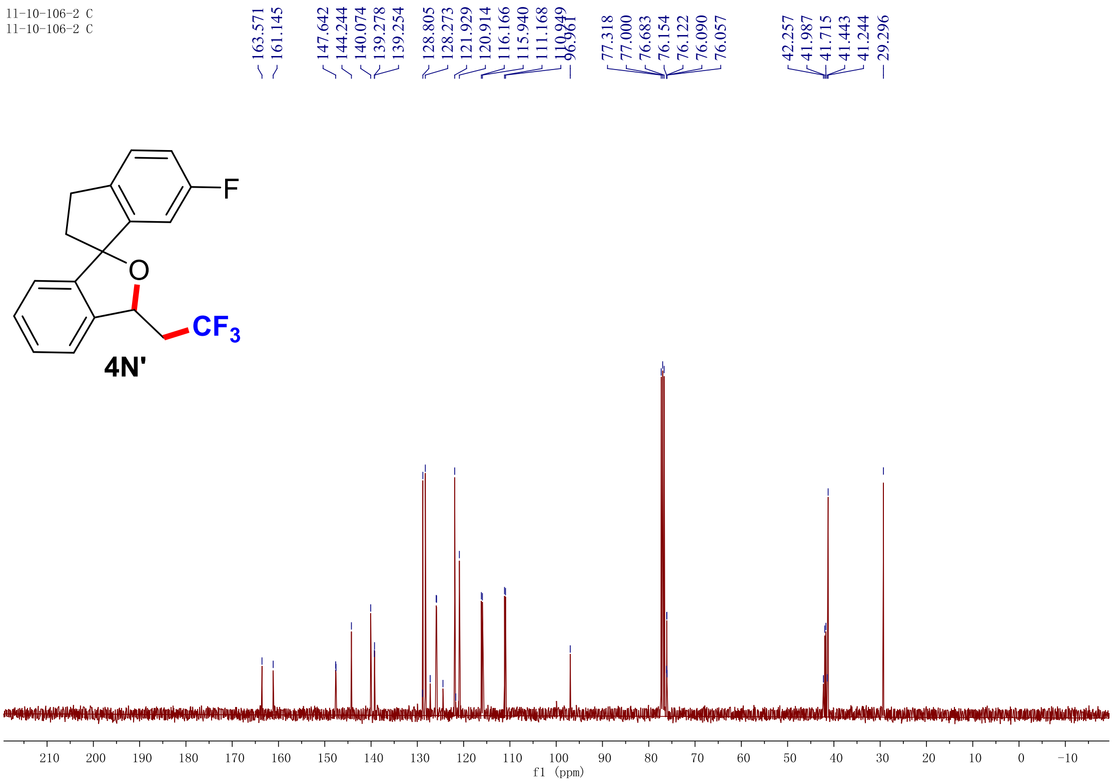

Supplementary Figure 111. ^{13}C NMR of **4N’**

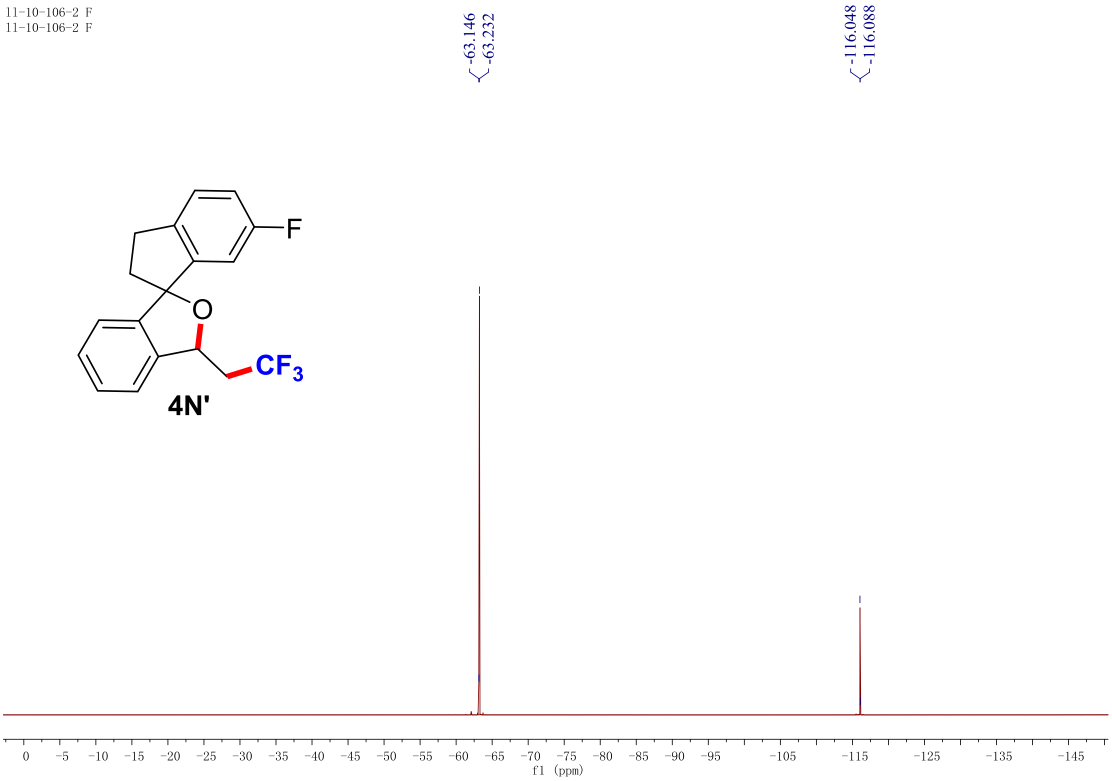

Supplementary Figure 112. ^{19}F NMR of **4N’**

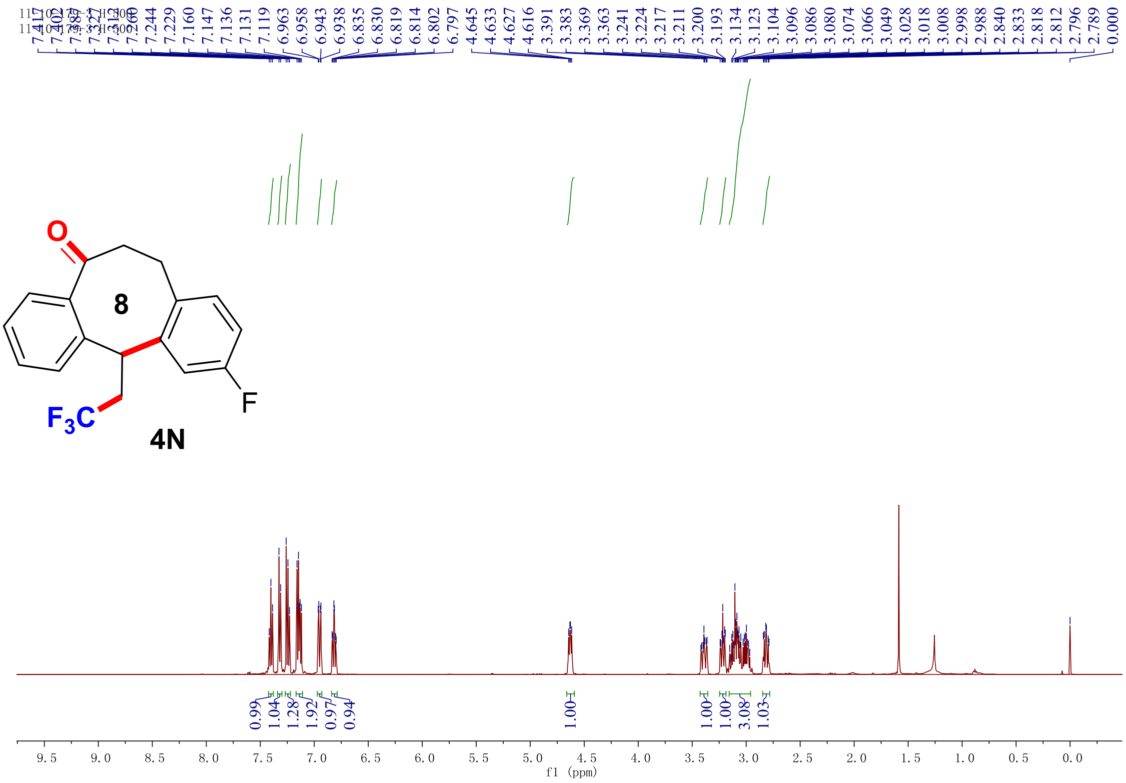

Supplementary Figure 113. ^{1}H NMR of **4N**

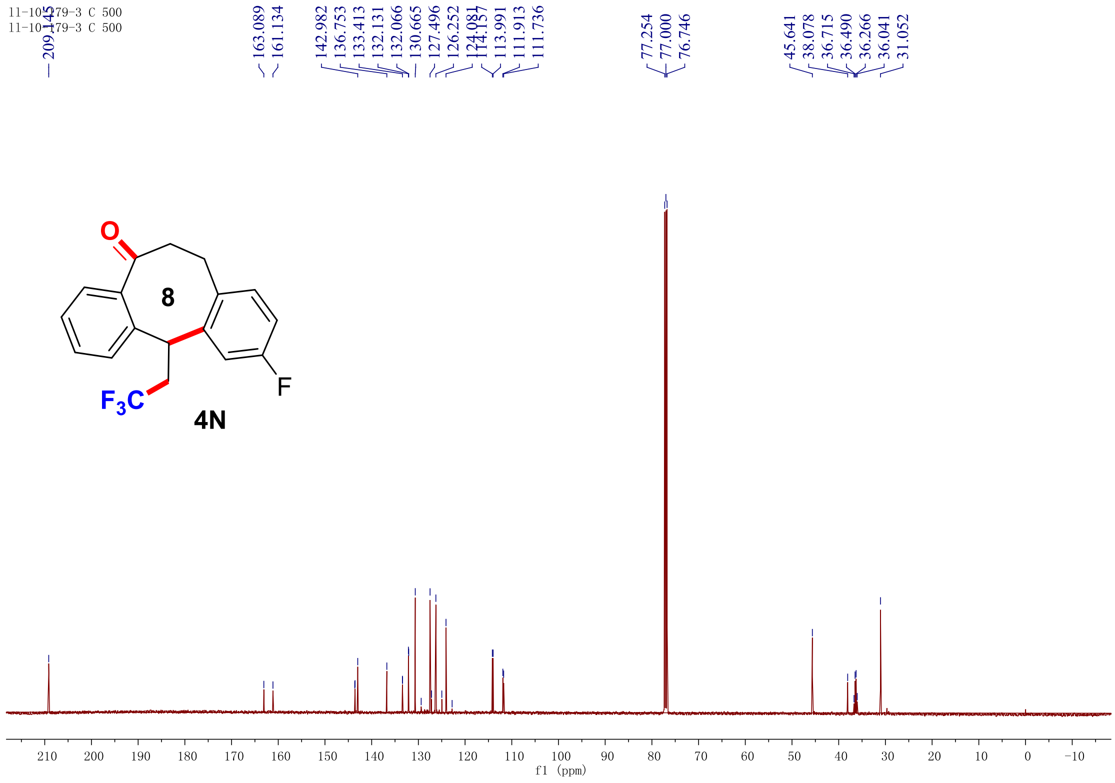

Supplementary Figure 114. ^{13}C NMR of **4N**

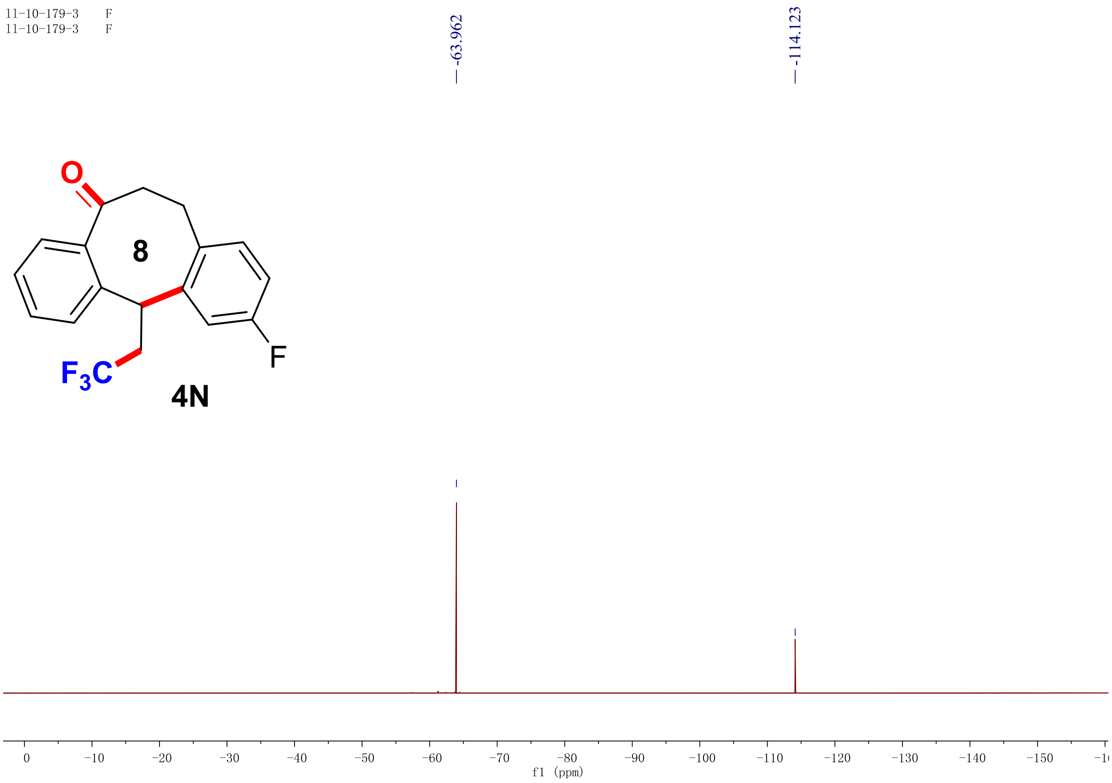

Supplementary Figure 115. ^{19}F NMR of **4N**

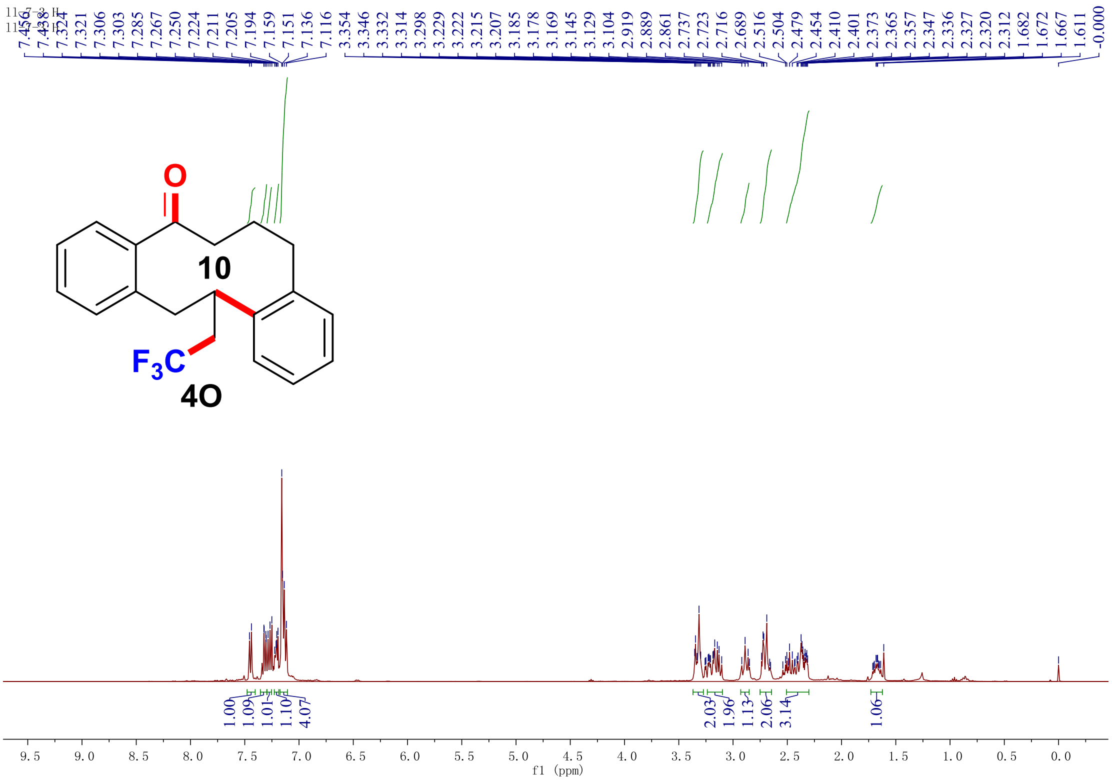

Supplementary Figure 116. ^{1}H NMR of **4O**

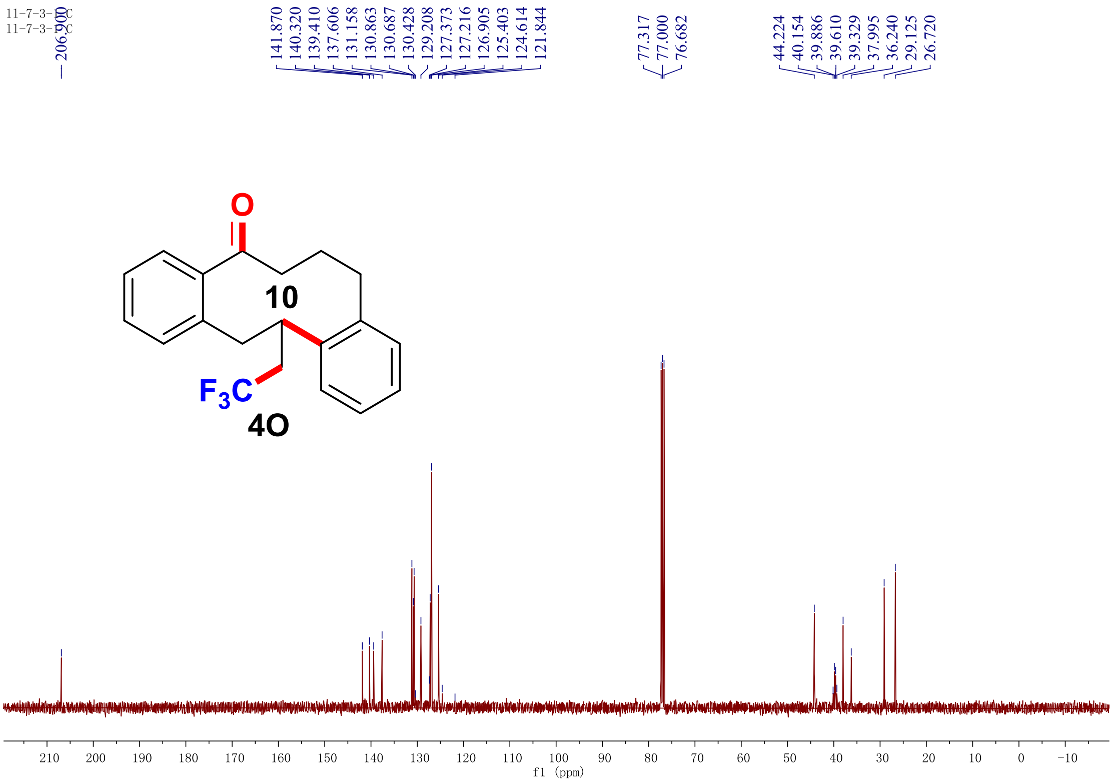

Supplementary Figure 117. ^{13}C NMR of **4O**

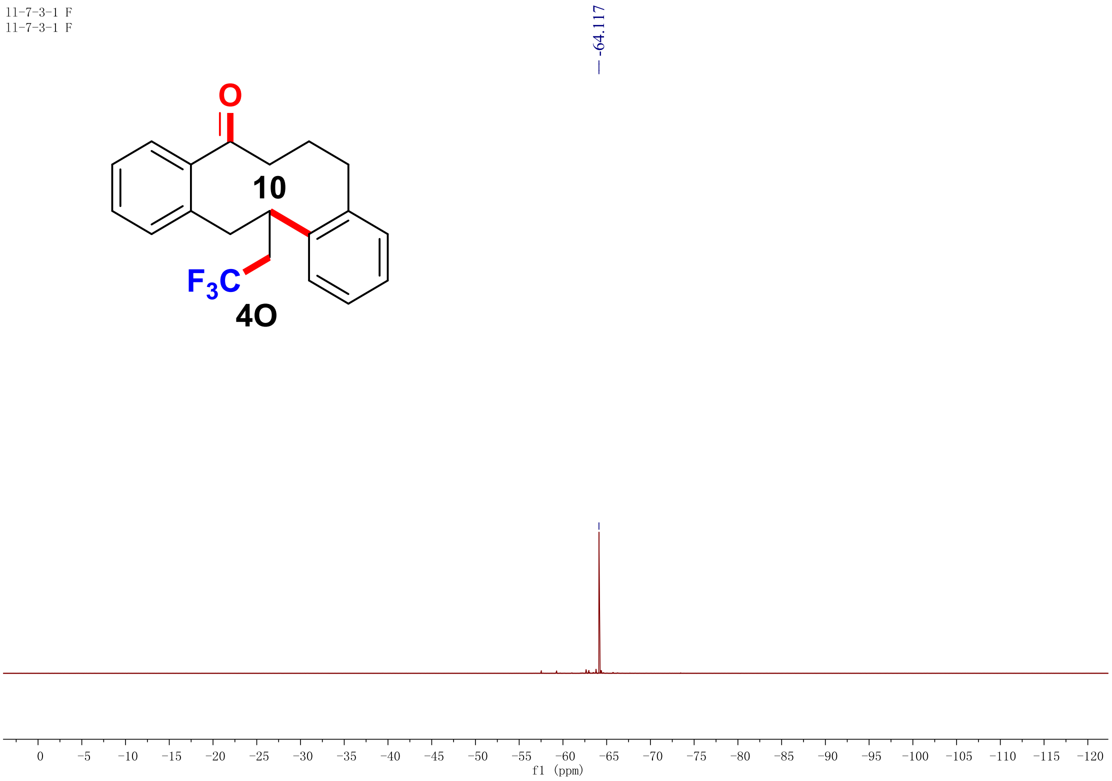

Supplementary Figure 118. ^{19}F NMR of **4O**

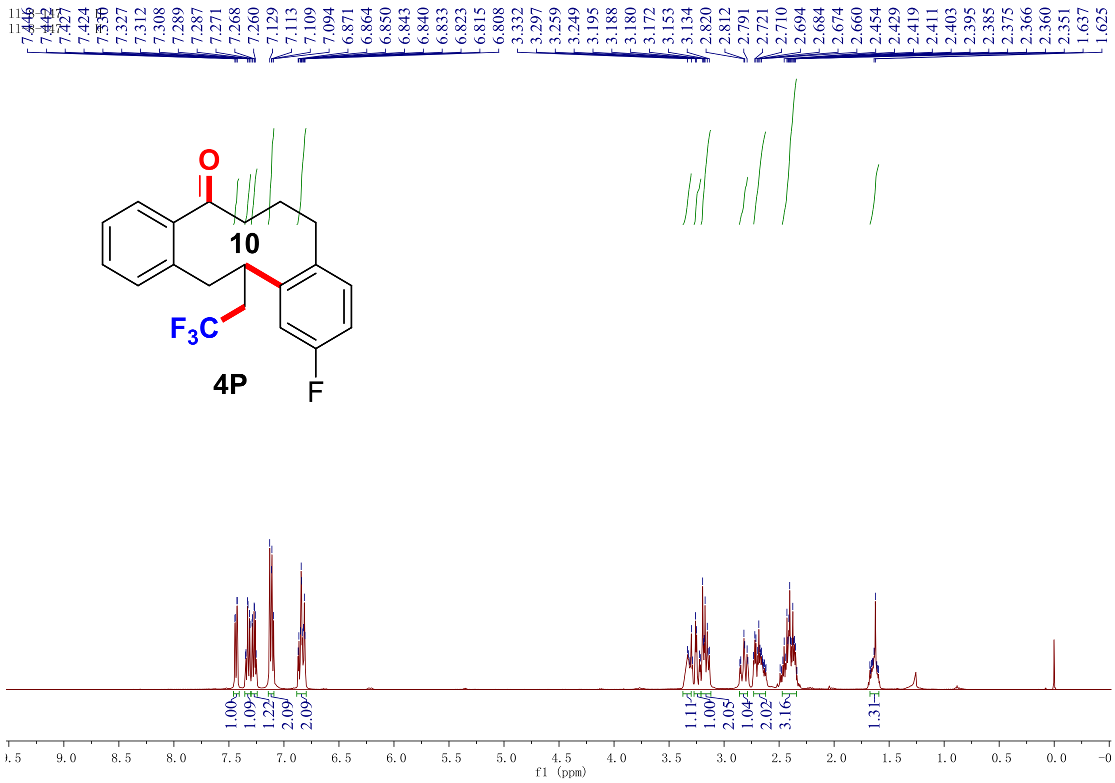

Supplementary Figure 119. ^{1}H NMR of **4P**

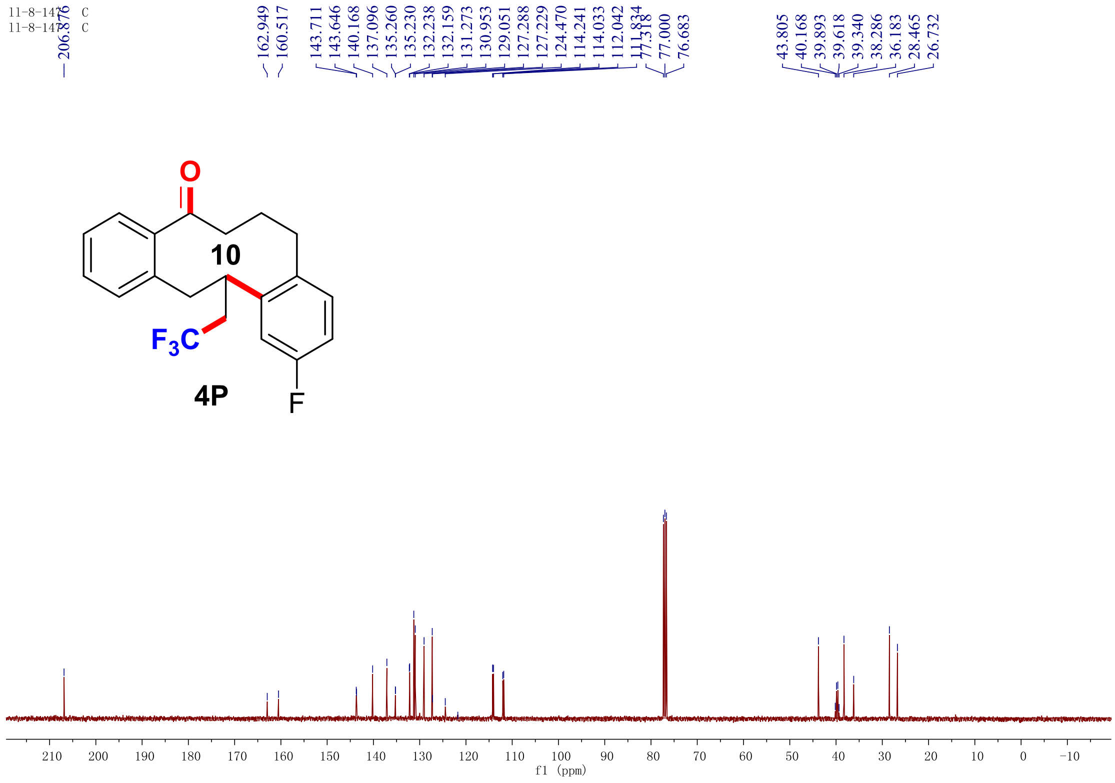

Supplementary Figure 120. ^{13}C NMR of **4P**

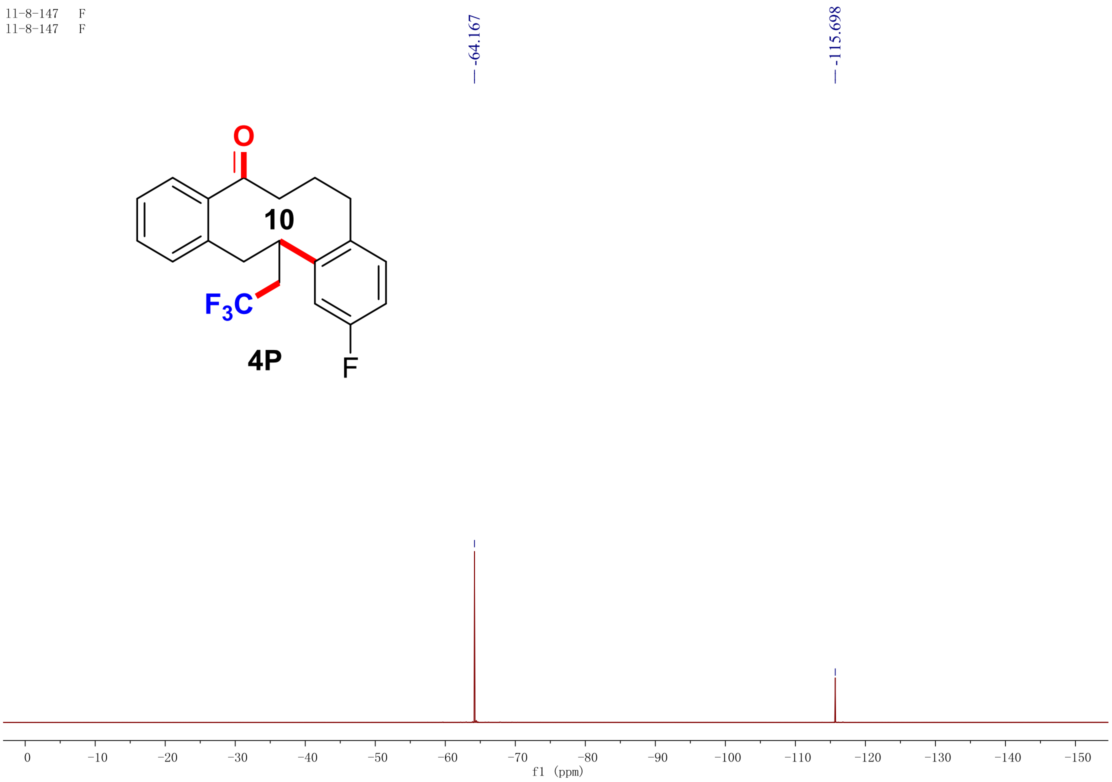

Supplementary Figure 121. ^{19}F NMR of **4P**

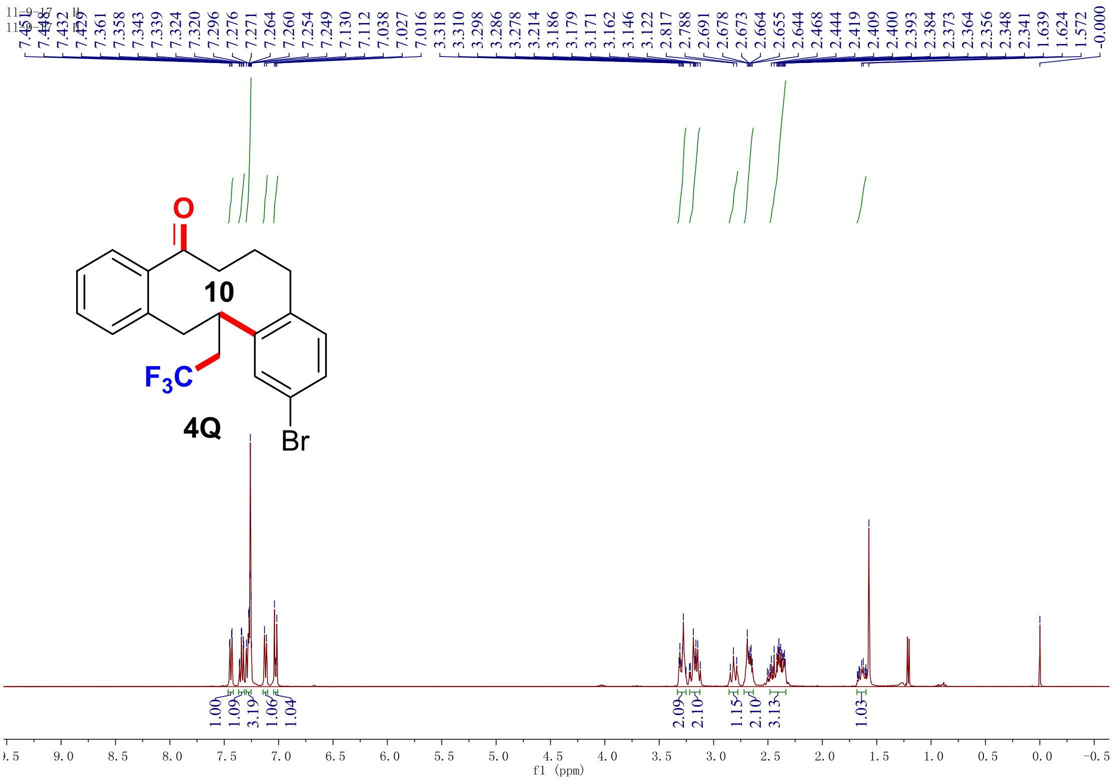

Supplementary Figure 122. ^{1}H NMR of **4Q**

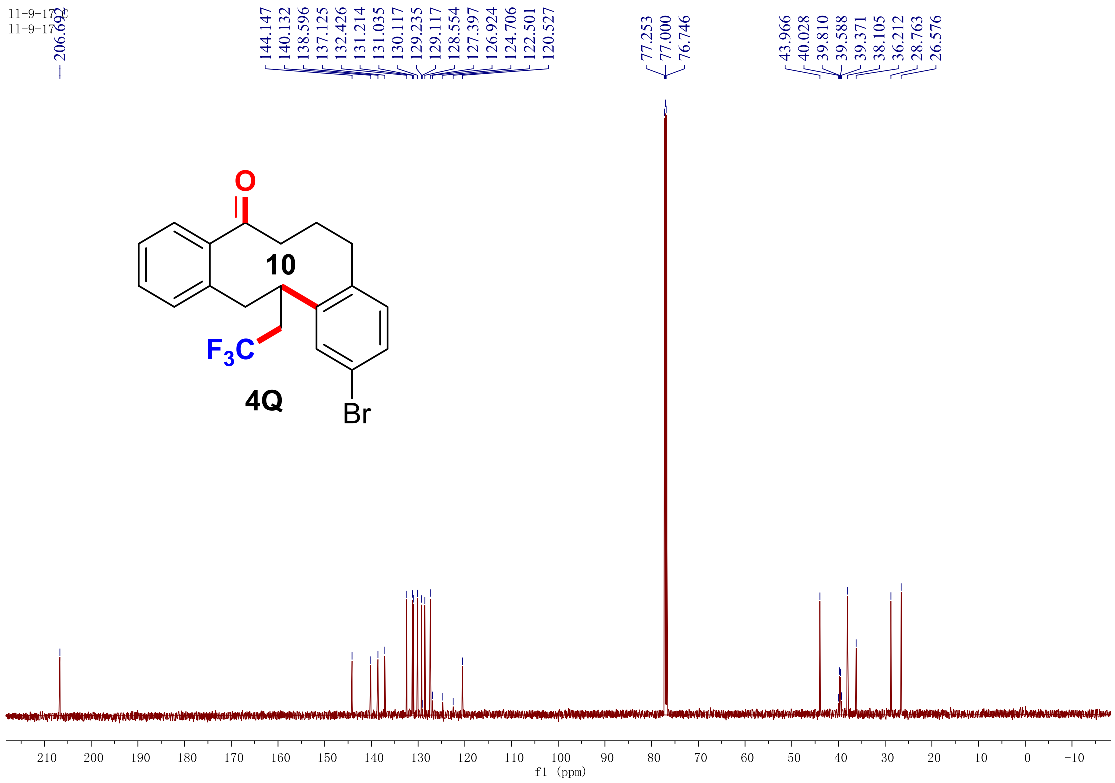

Supplementary Figure 123. ^{13}C NMR of **4Q**

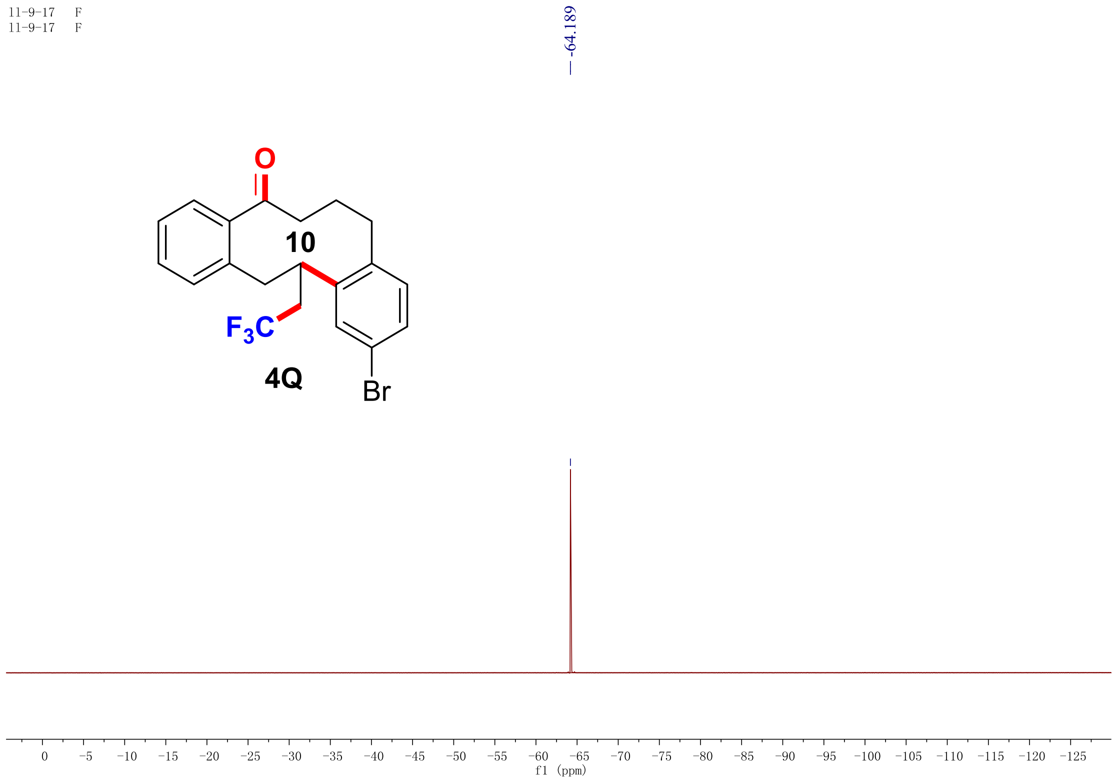

Supplementary Figure 124. ^{19}F NMR of **4Q**

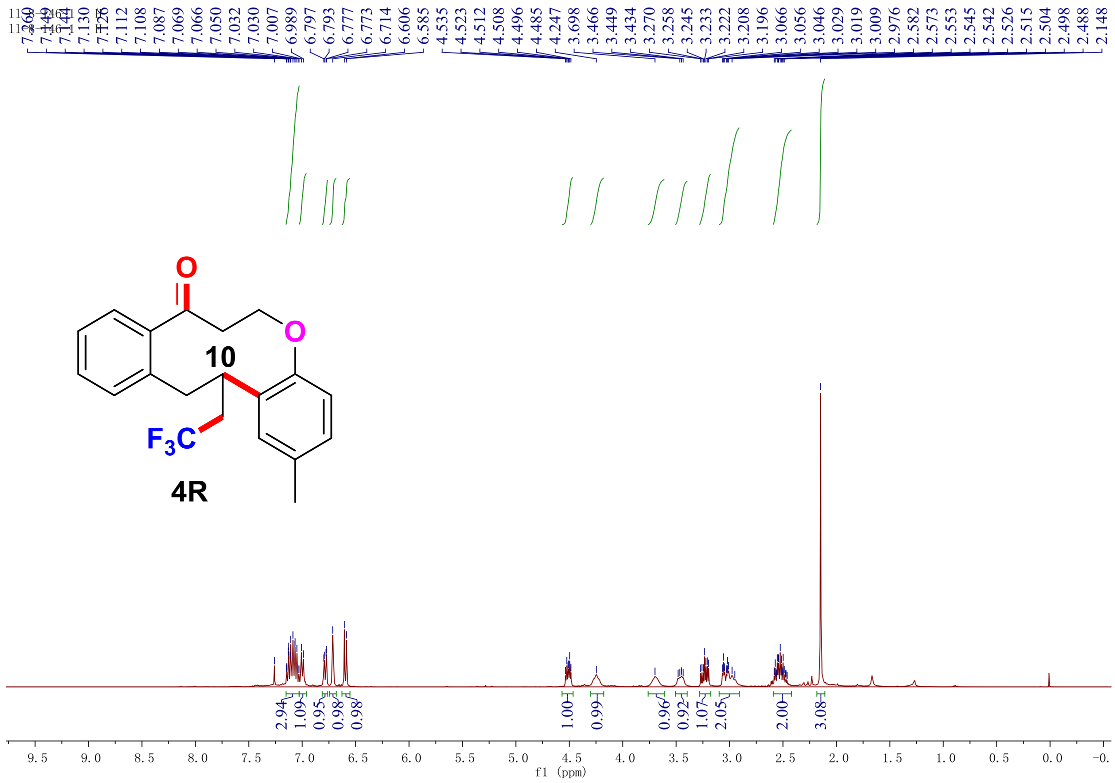

Supplementary Figure 125. ^{1}H NMR of **4R**

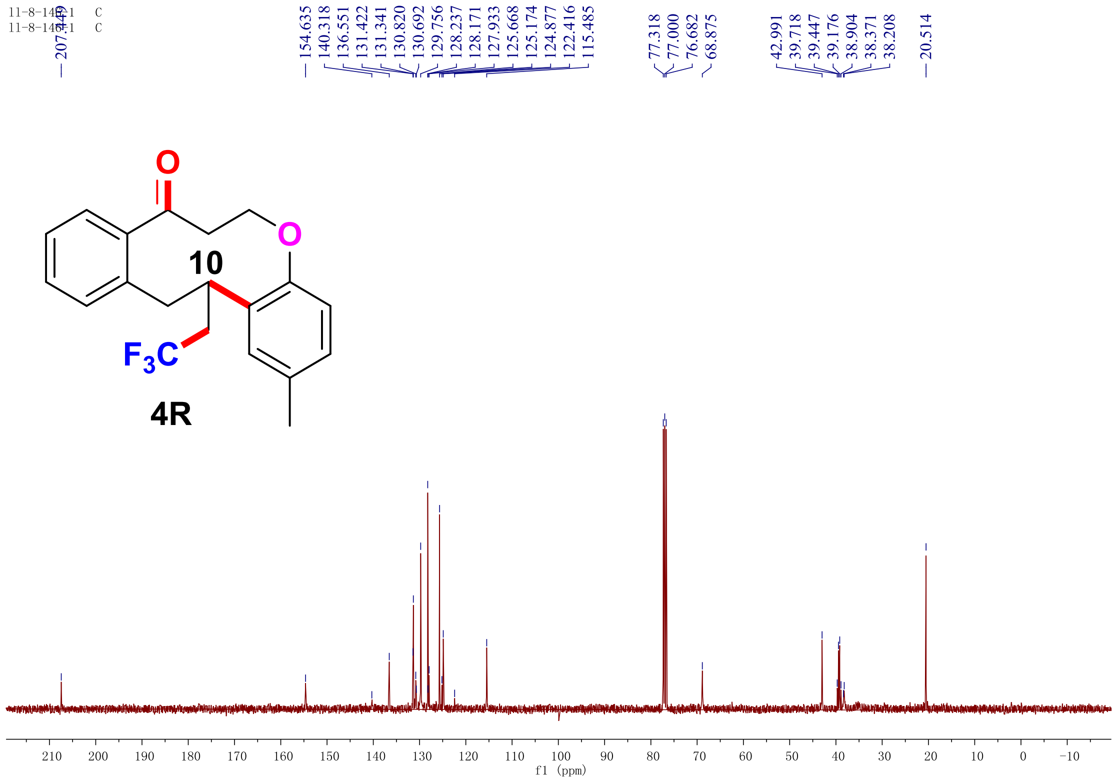

Supplementary Figure 126. ^{13}C NMR of **4R**

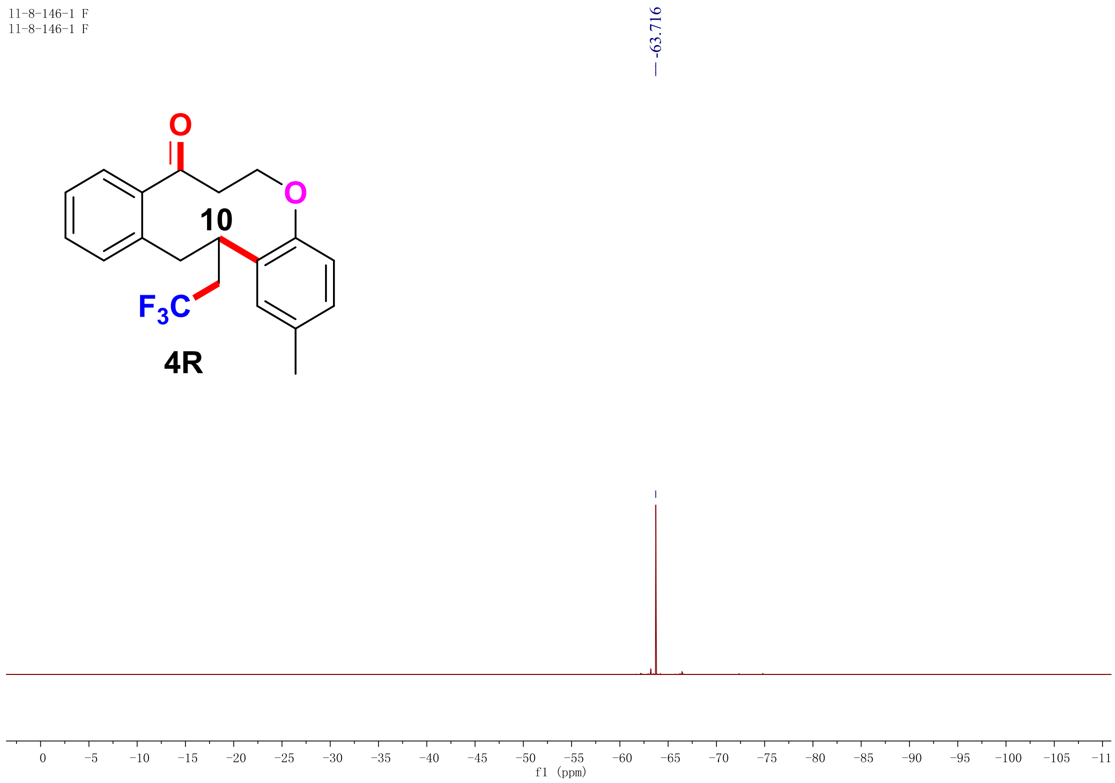

Supplementary Figure 127. ^{19}F NMR of **4R**

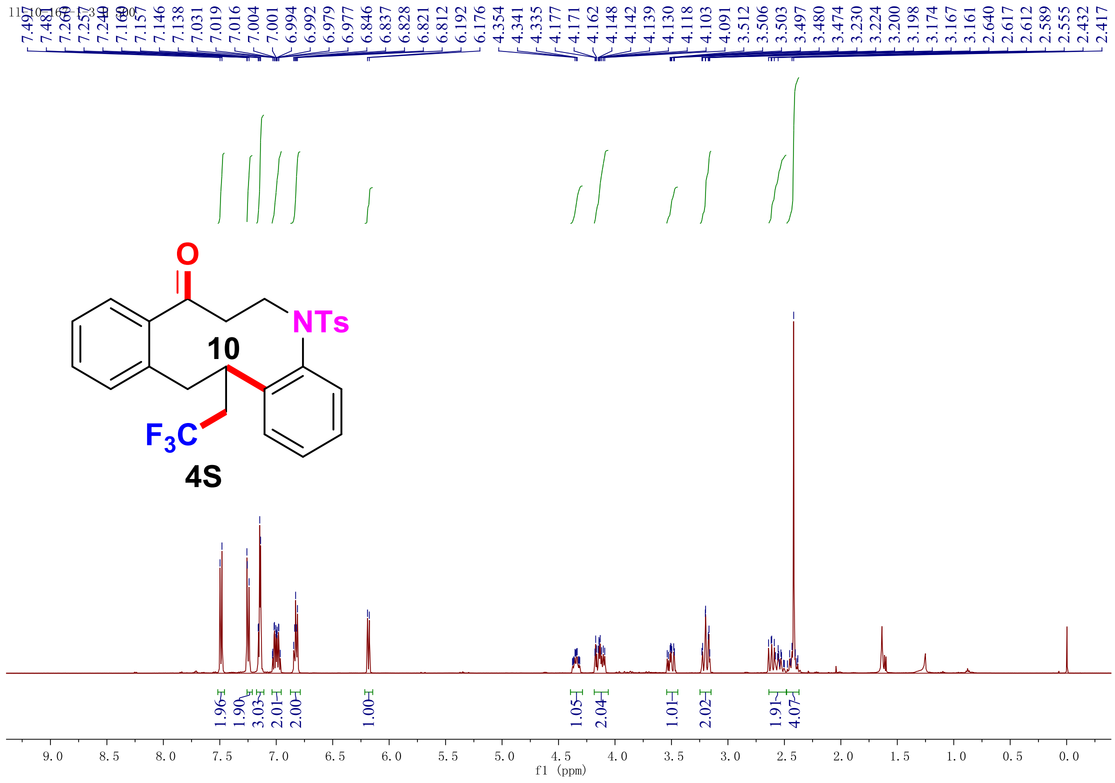

Supplementary Figure 128. ^{1}H NMR of **4S**

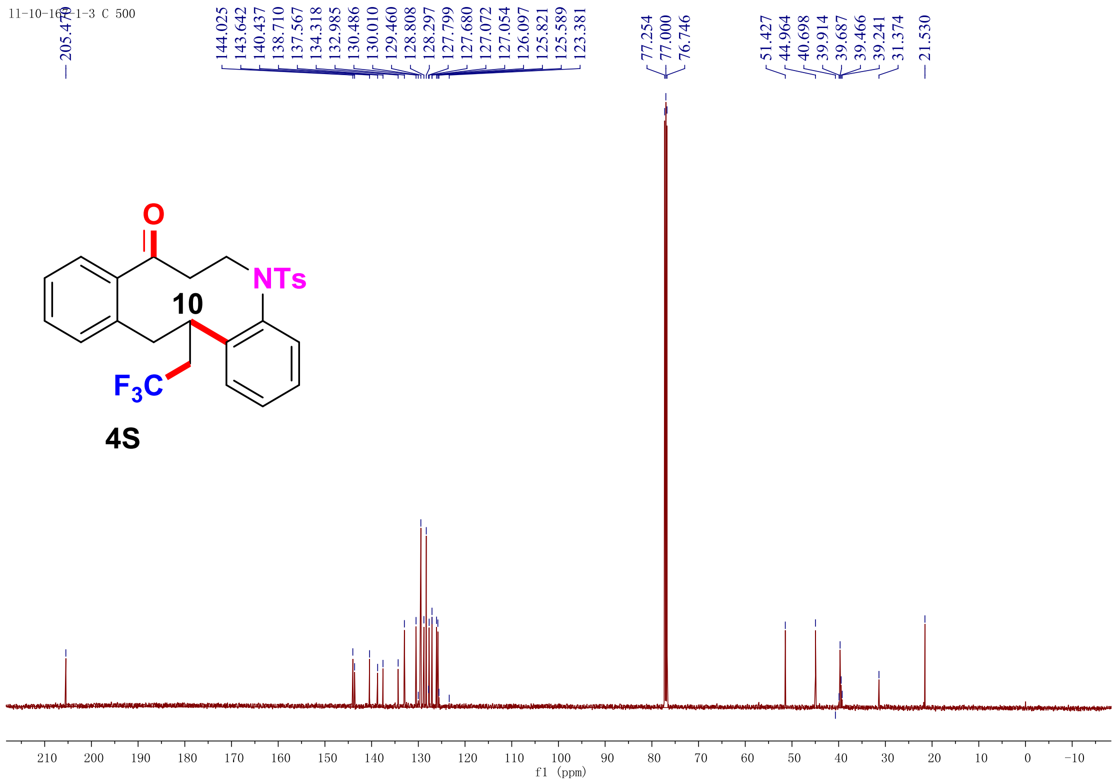

Supplementary Figure 129. ^{13}C NMR of **4S**

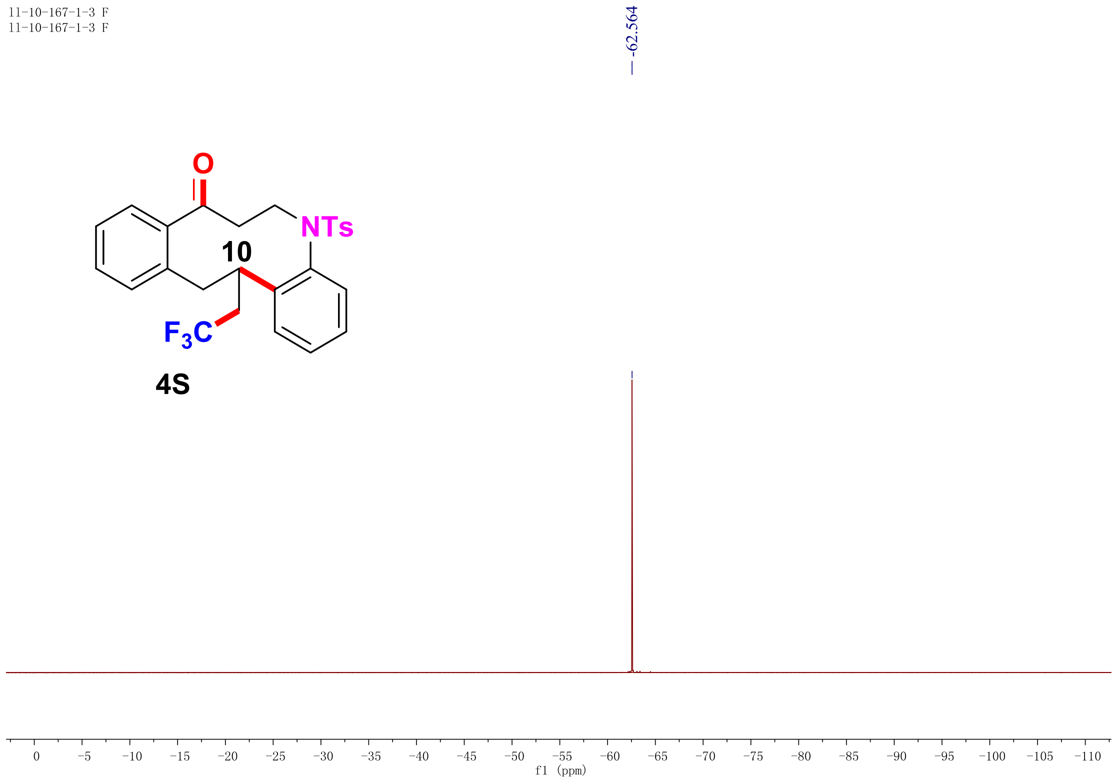

Supplementary Figure 130. ^{19}F NMR of **4S**

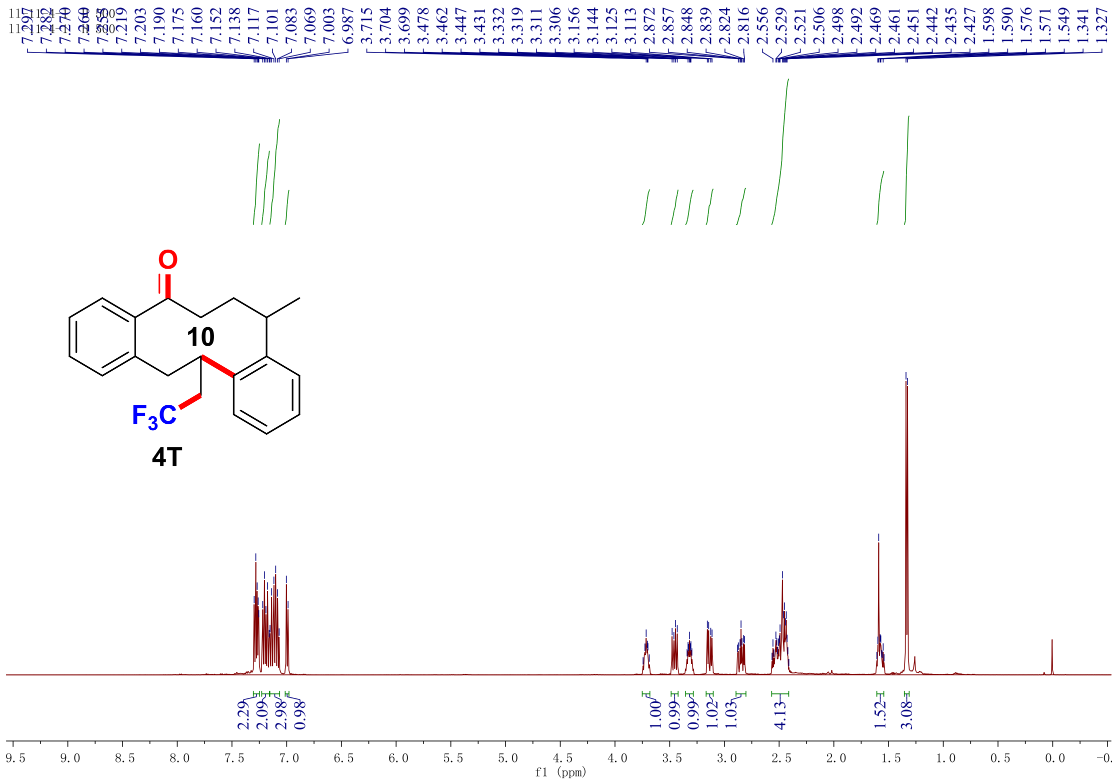

Supplementary Figure 131. ^{1}H NMR of **4T**

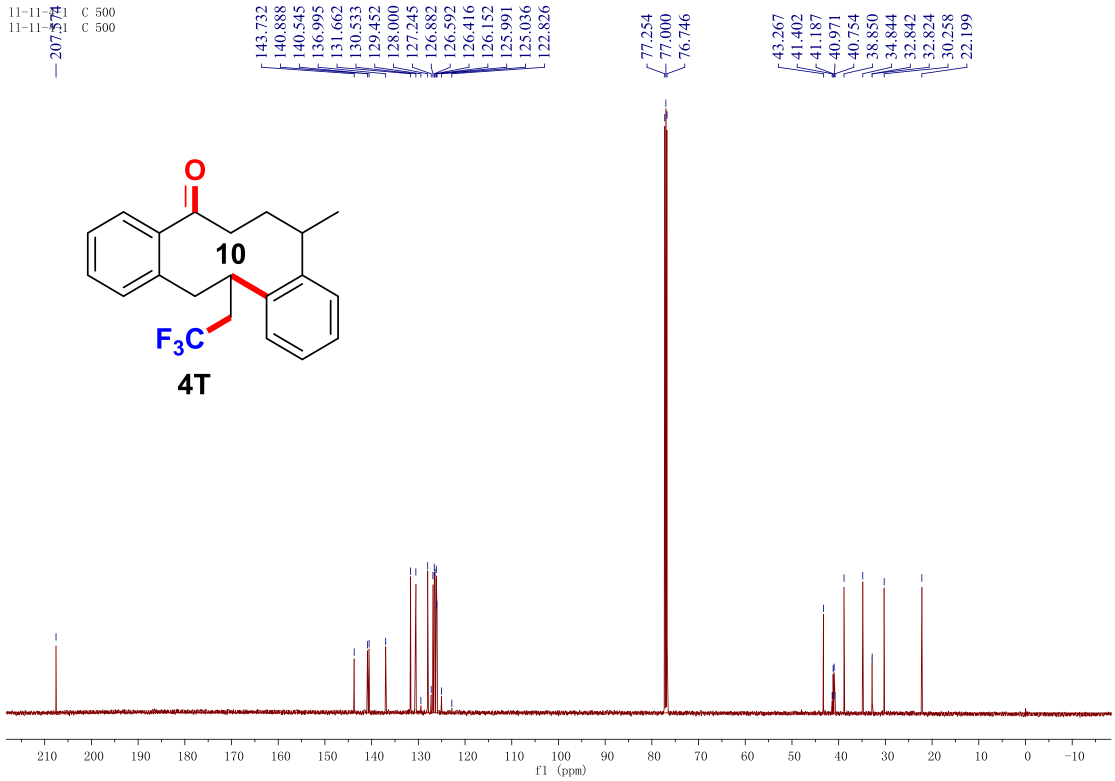

Supplementary Figure 132. ^{13}C NMR of **4T**

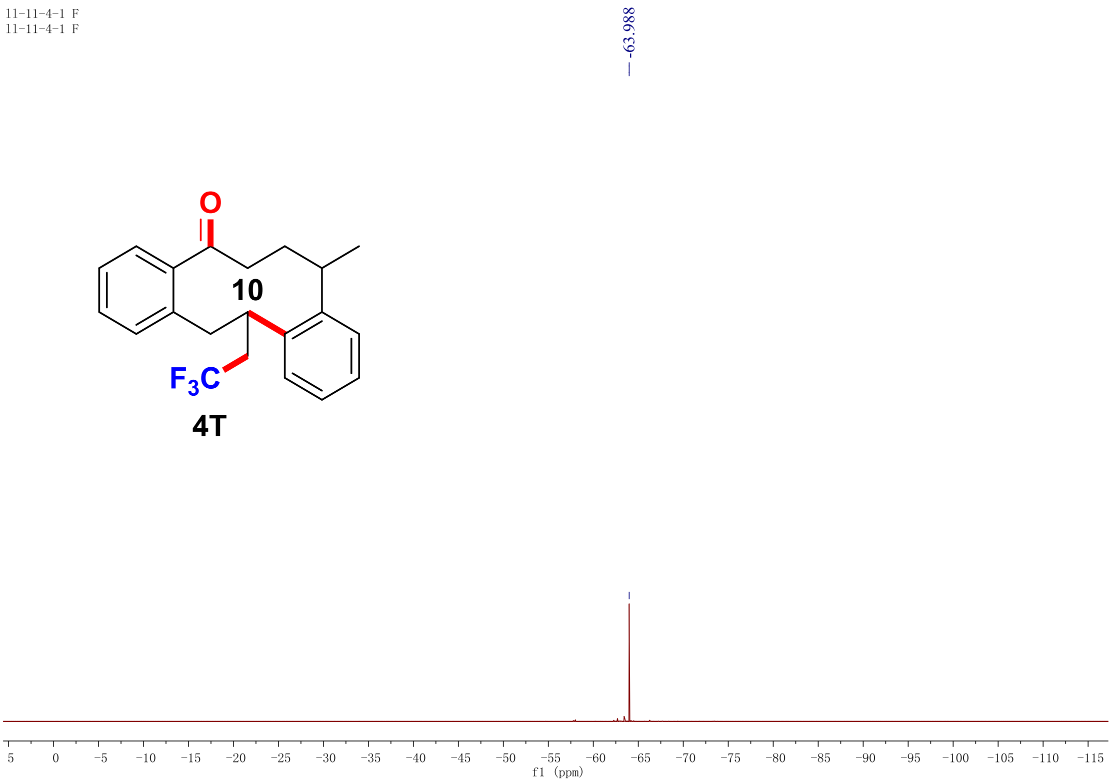

Supplementary Figure 133. ^{19}F NMR of **4T**

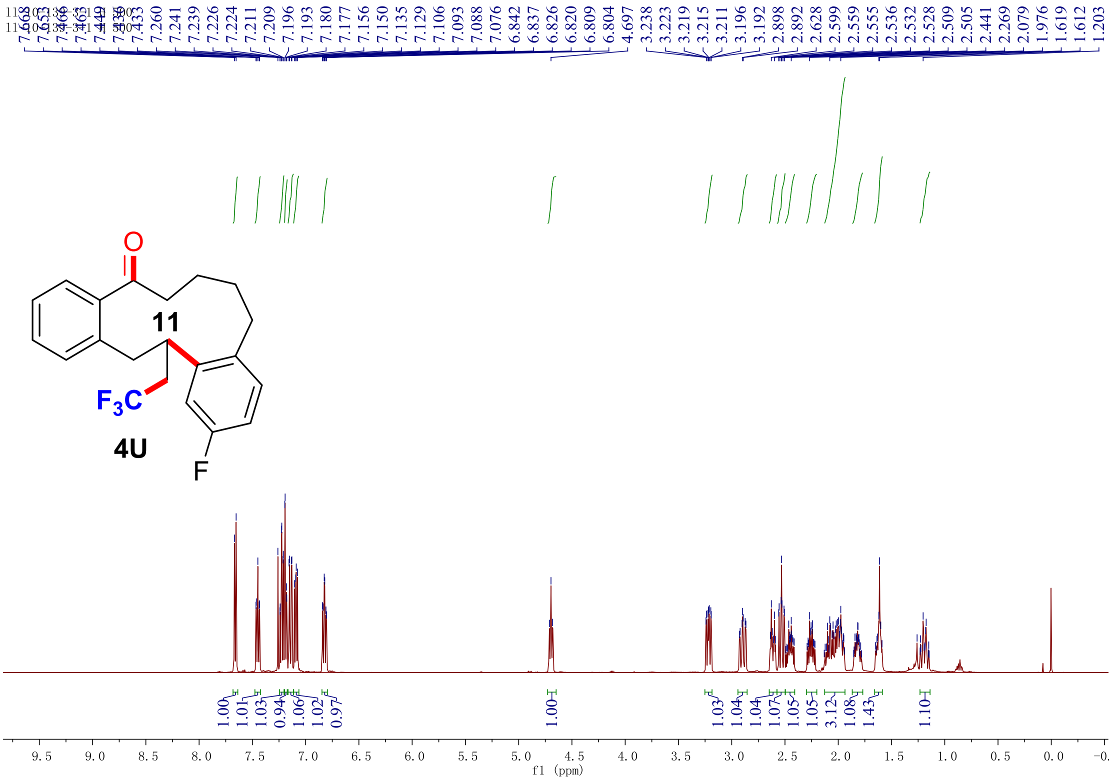

Supplementary Figure 134. ^{1}H NMR of **4U**

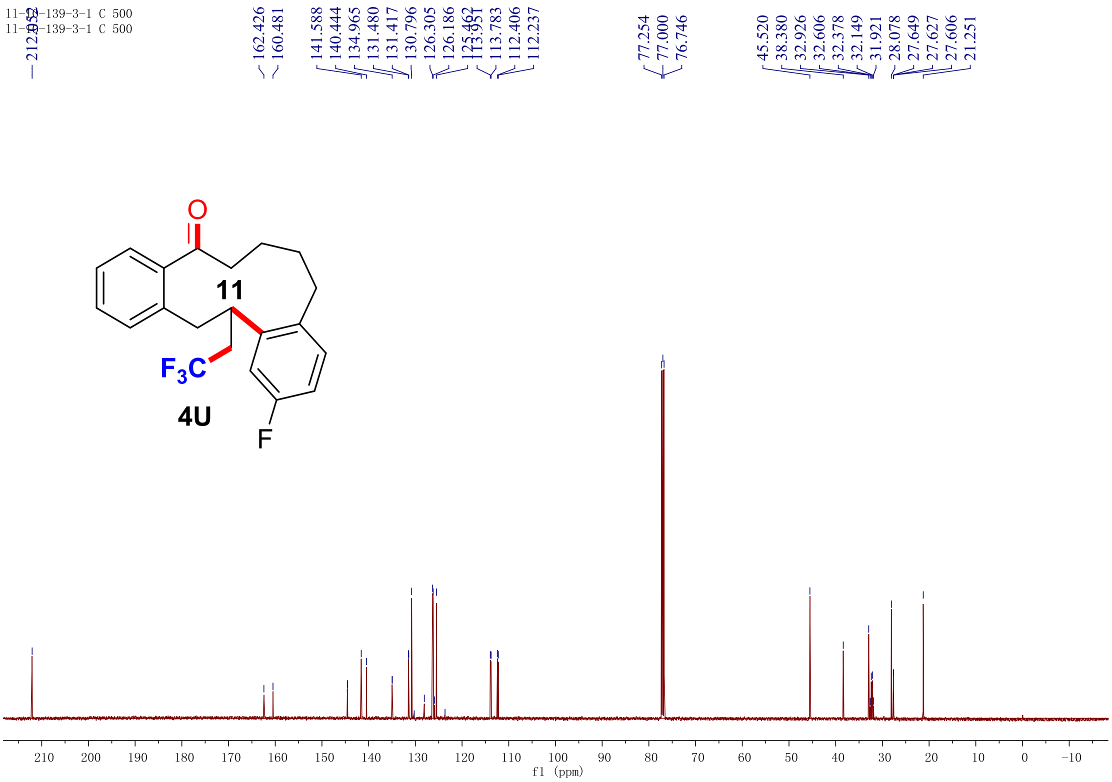

Supplementary Figure 135. ^{13}C NMR of **4U**

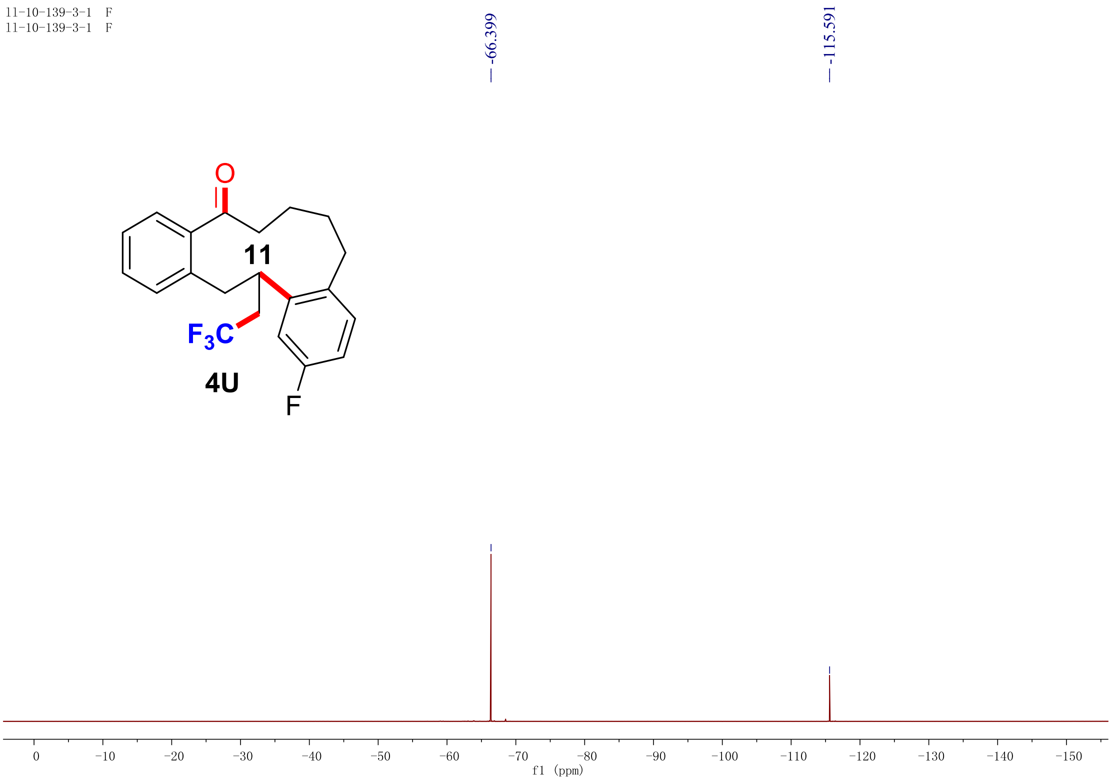

Supplementary Figure 136. ^{19}F NMR of **4U**

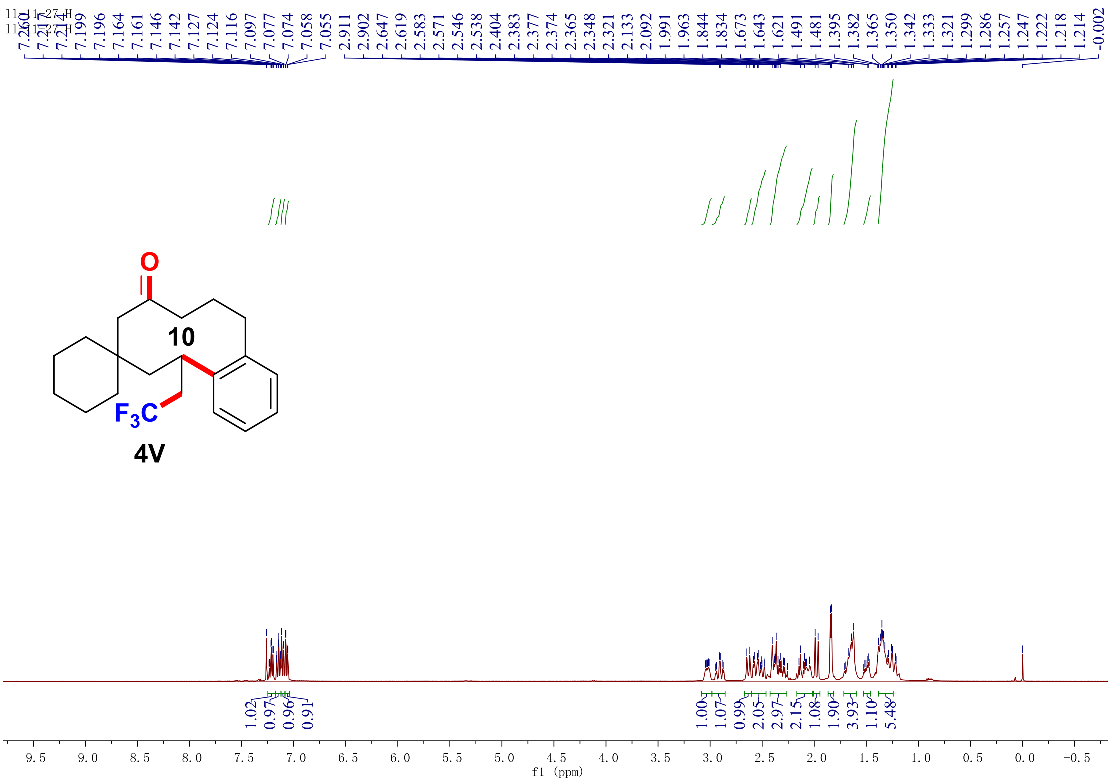

Supplementary Figure 137. ^{1}H NMR of **4V**

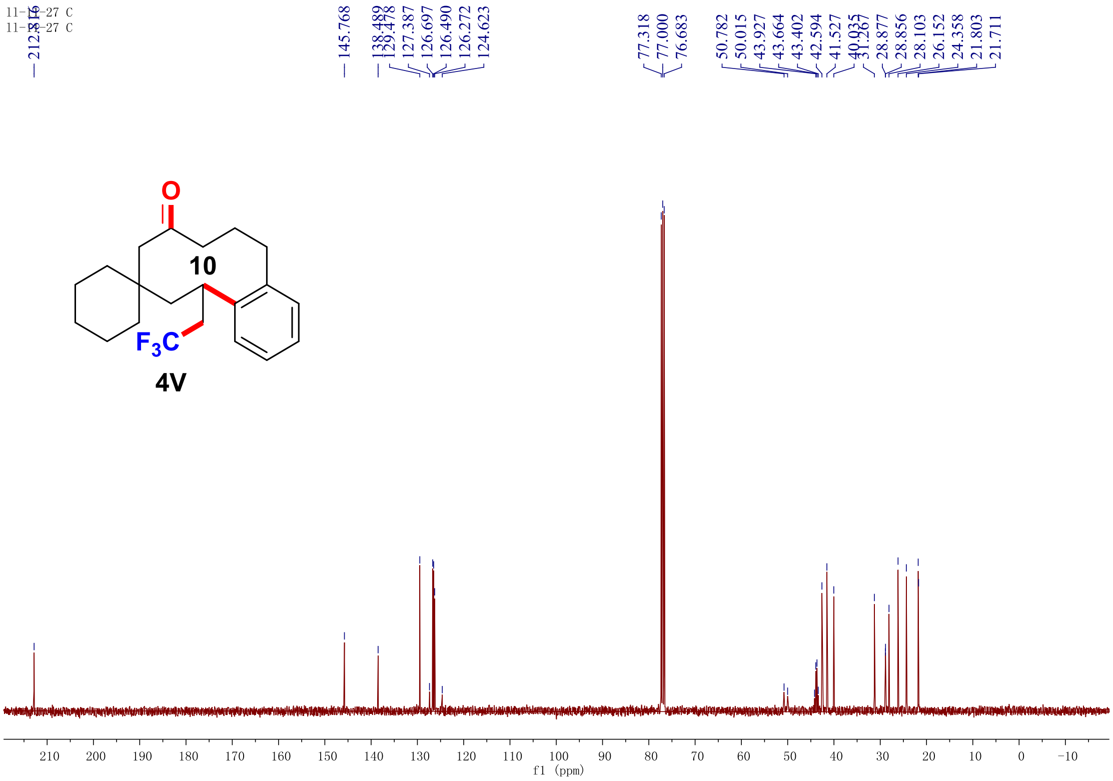

Supplementary Figure 138. ^{13}C NMR of **4V**

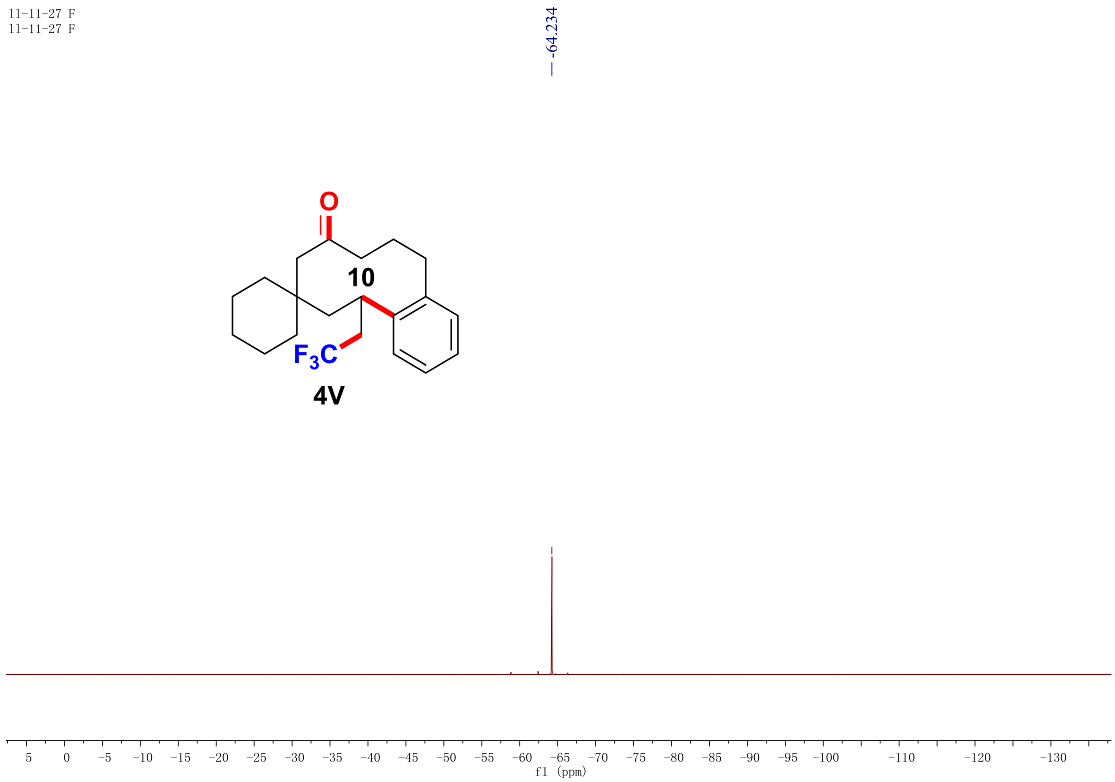

Supplementary Figure 139. ^{19}F NMR of **4V**

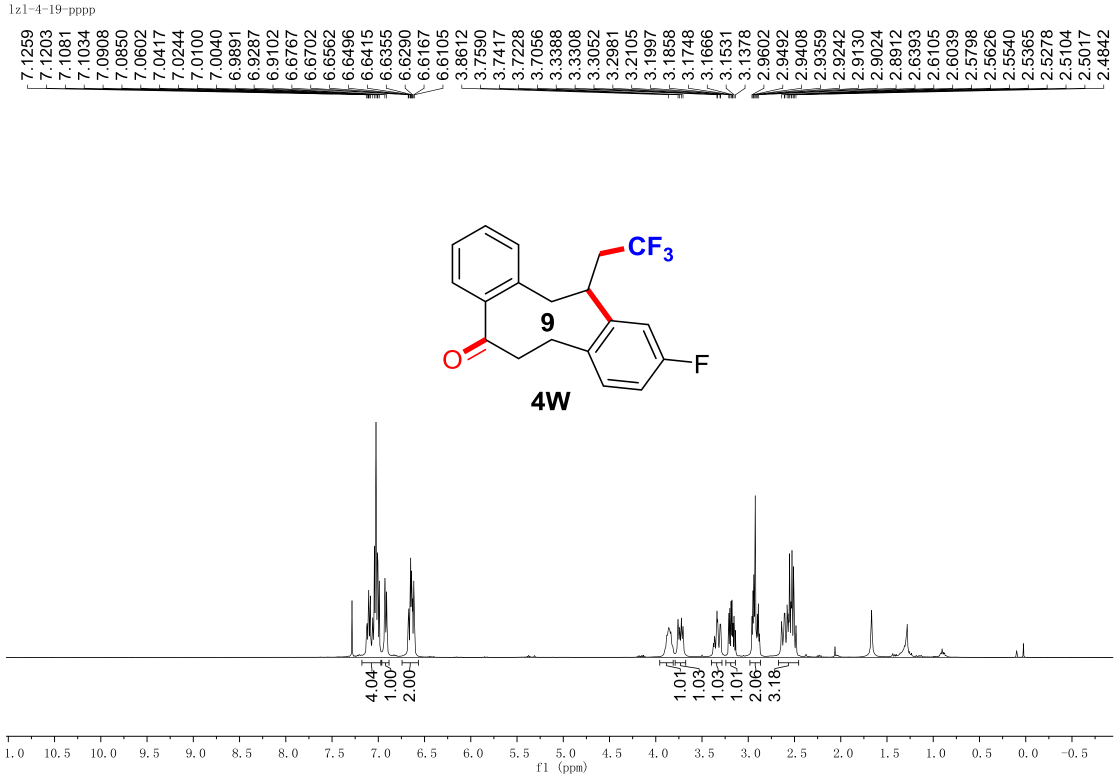

Supplementary Figure 140. ^{1}H NMR of **4W**

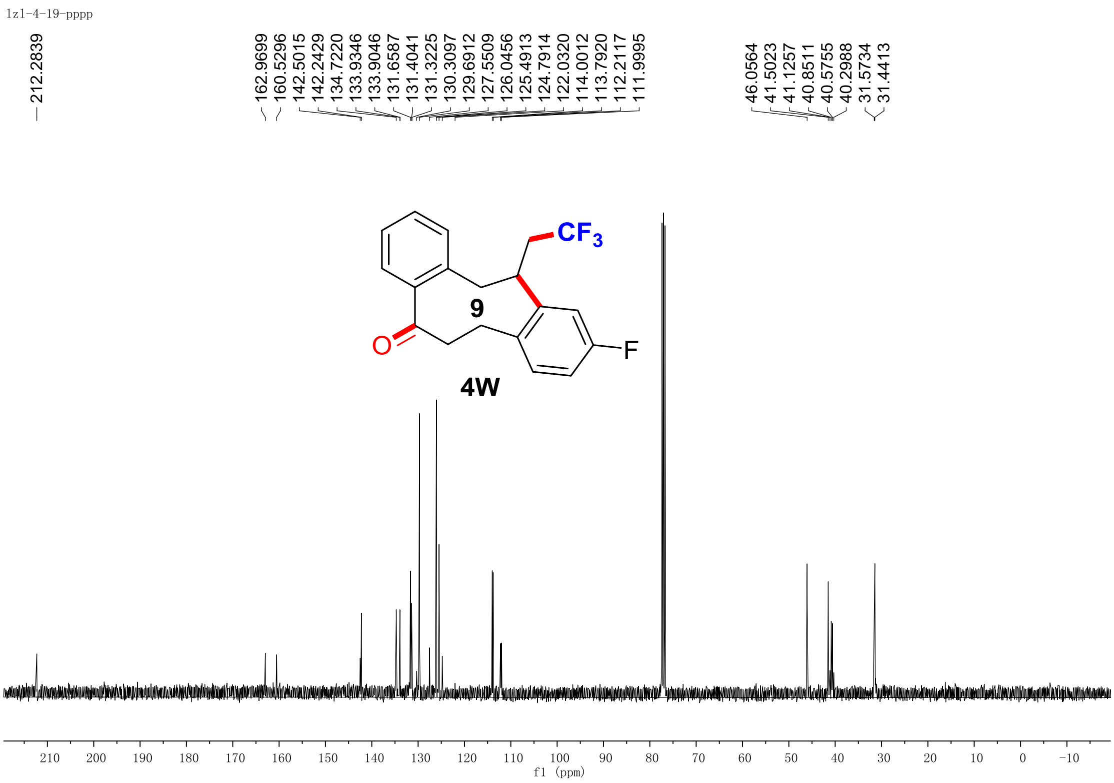

Supplementary Figure 141. ^{13}C NMR of **4W**

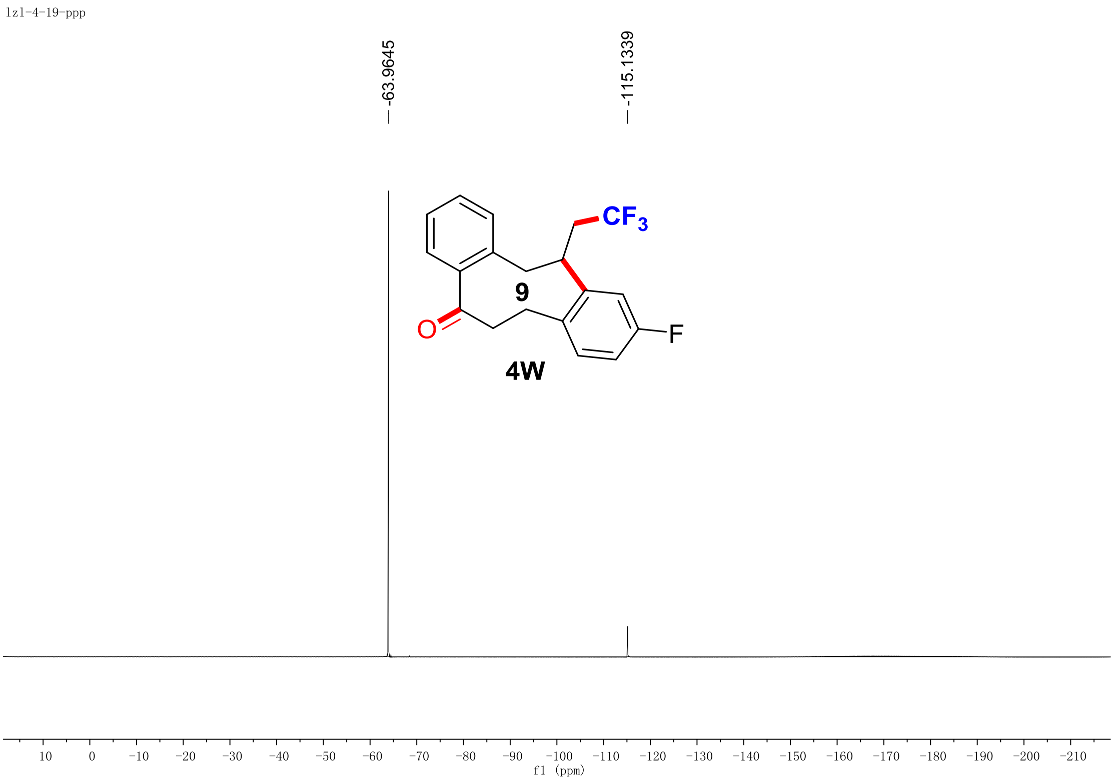

Supplementary Figure 142. ^{19}F NMR of **4W**

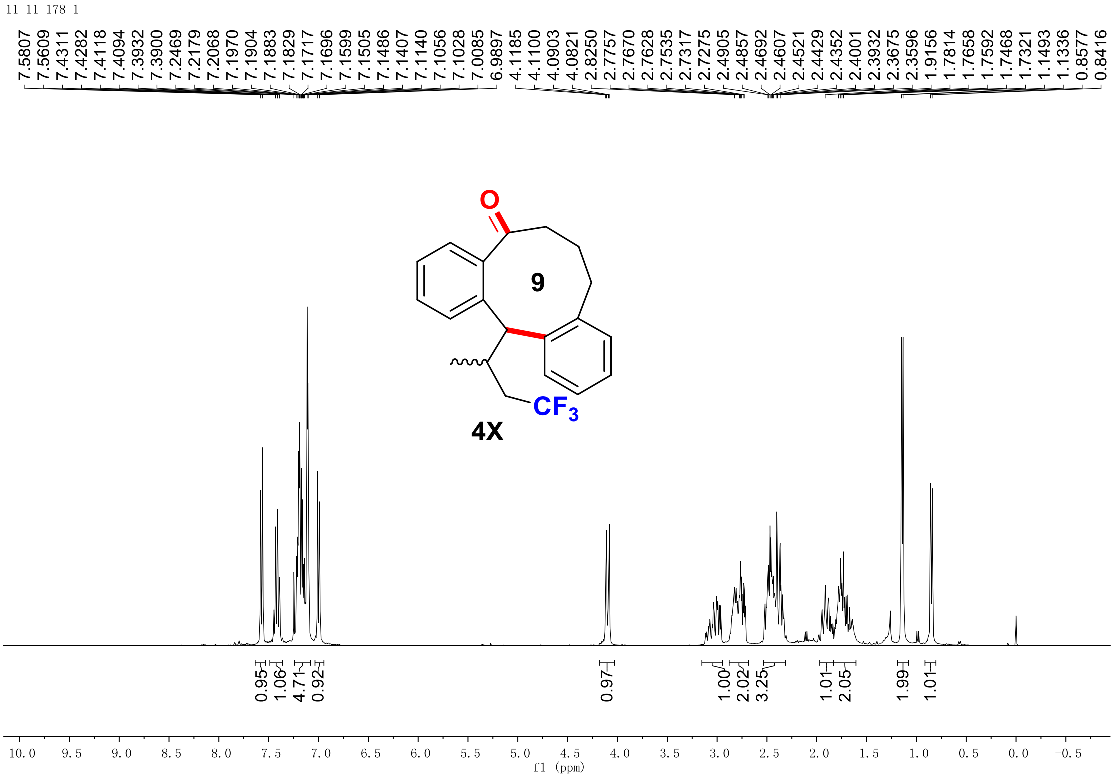

Supplementary Figure 143. ^{1}H NMR of **4X**

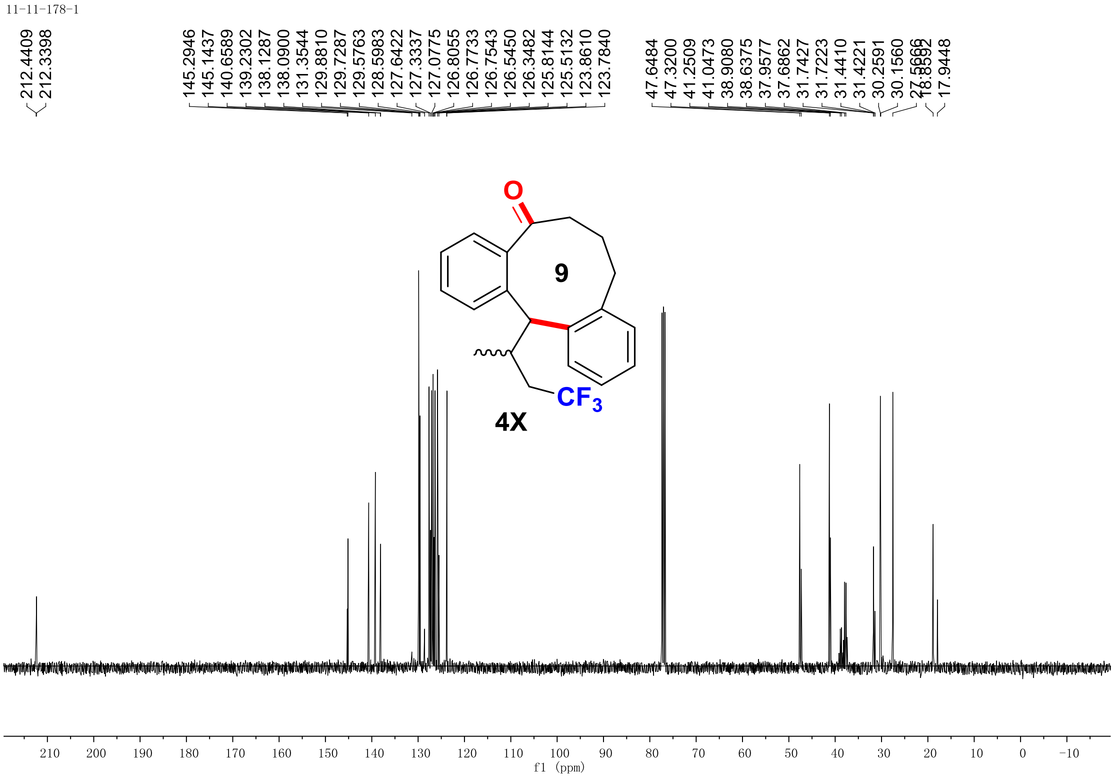

Supplementary Figure 144. ^{13}C NMR of **4X**

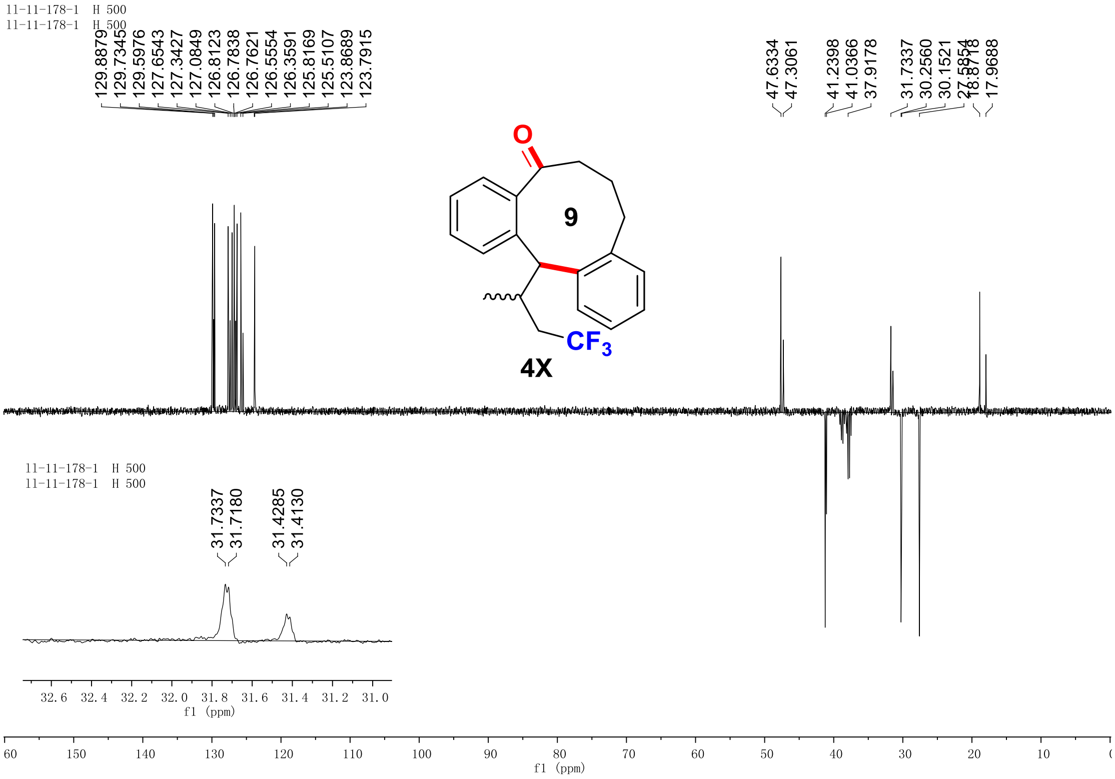

Supplementary Figure 145. DEPT135 of **4X**

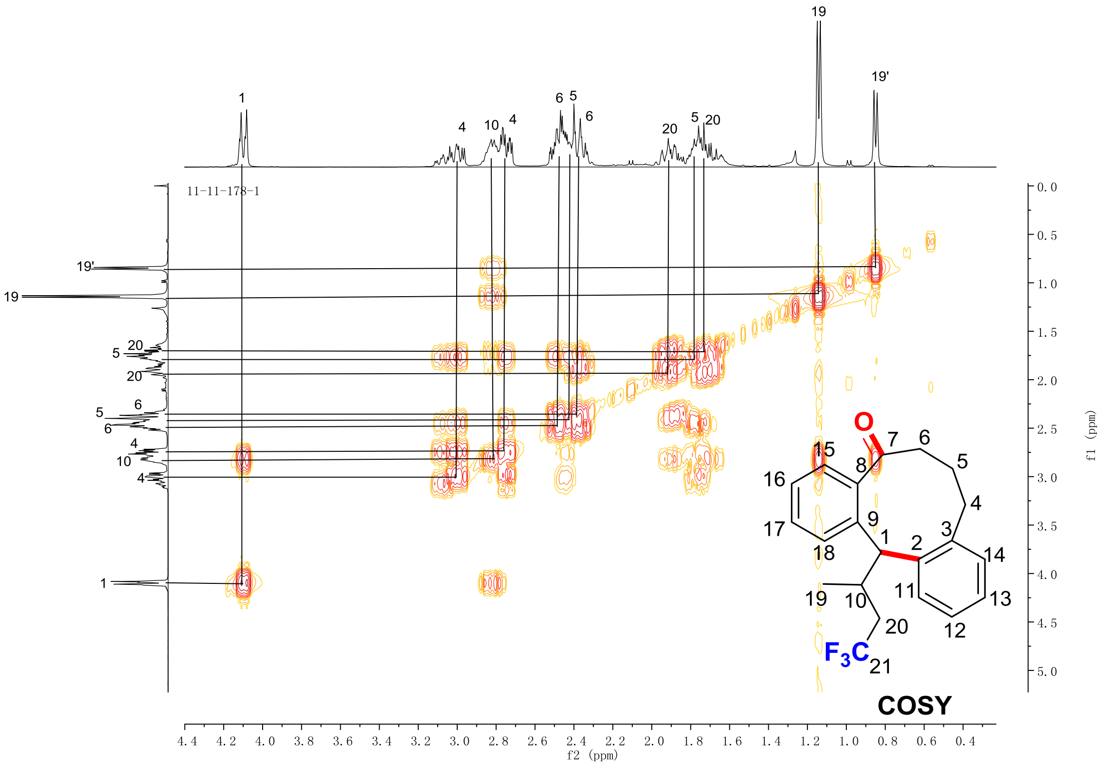

Supplementary Figure 146. COSY of **4X**

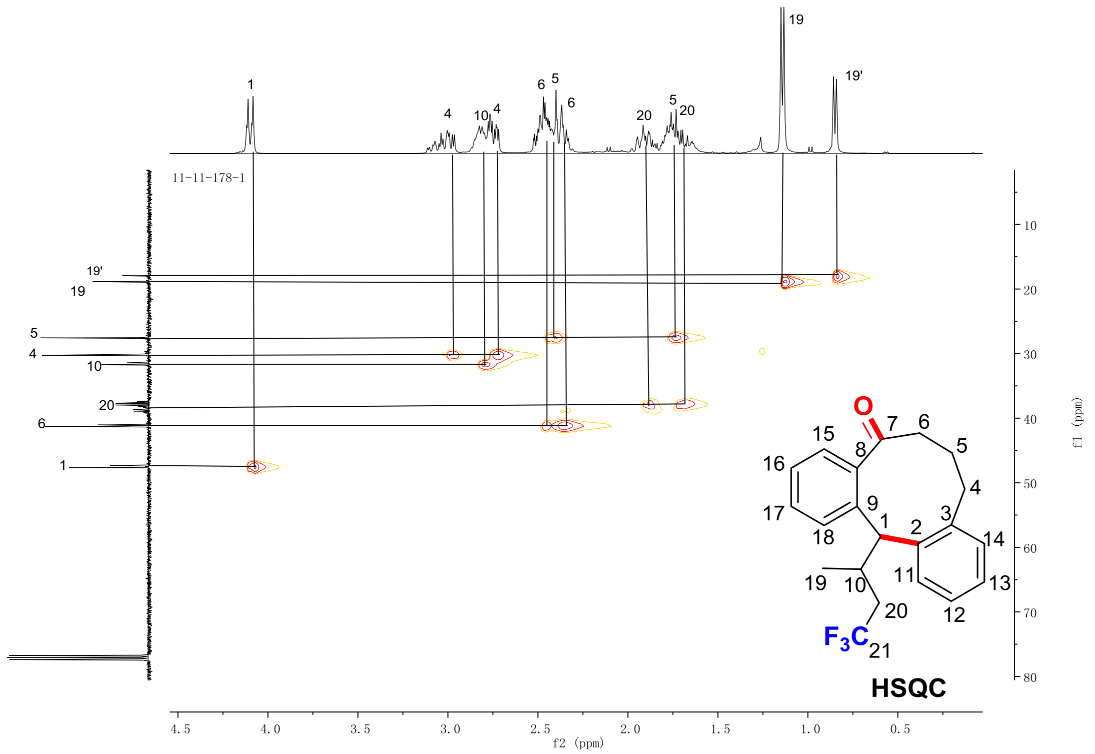

Supplementary Figure 147. HSQC of **4X**

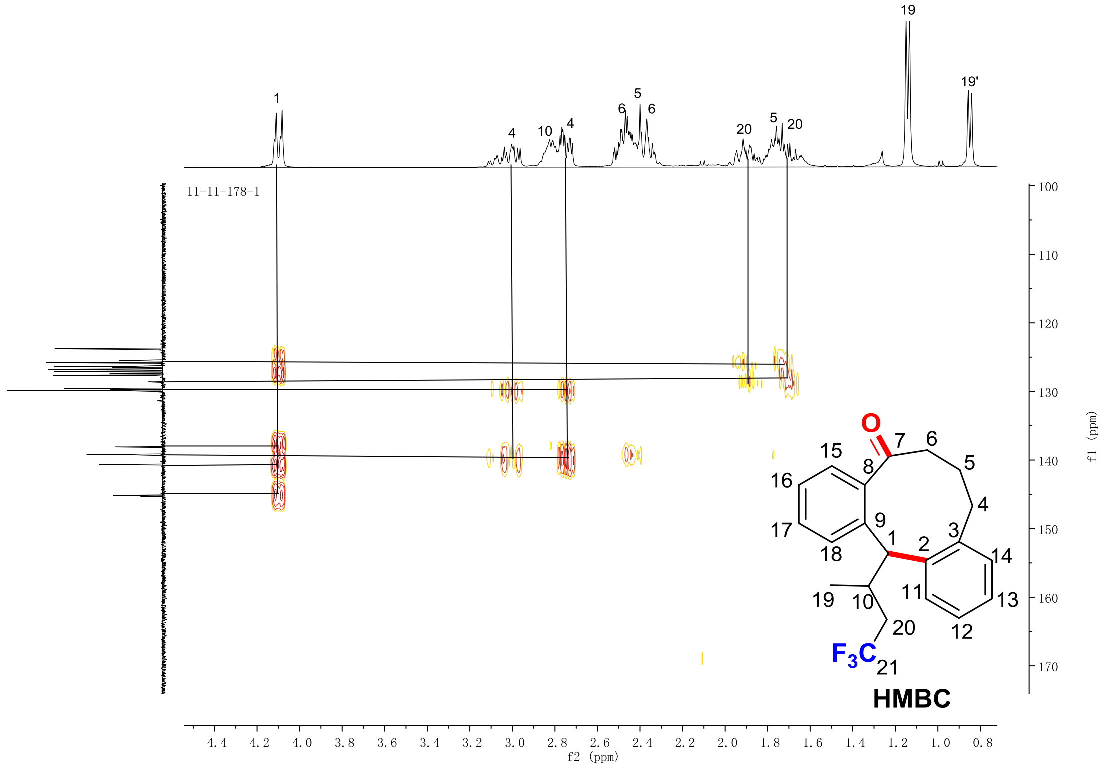

Supplementary Figure 148. HMBC NMR of **4X**

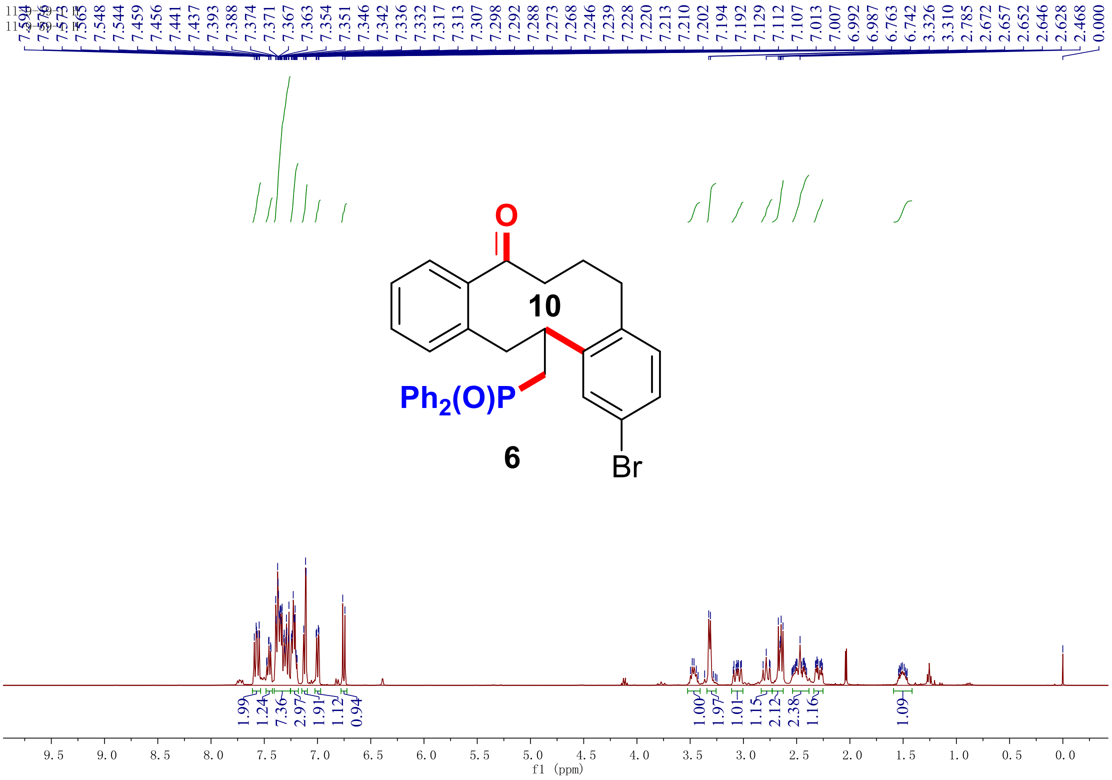

Supplementary Figure 149. ^{1}H NMR of **6**

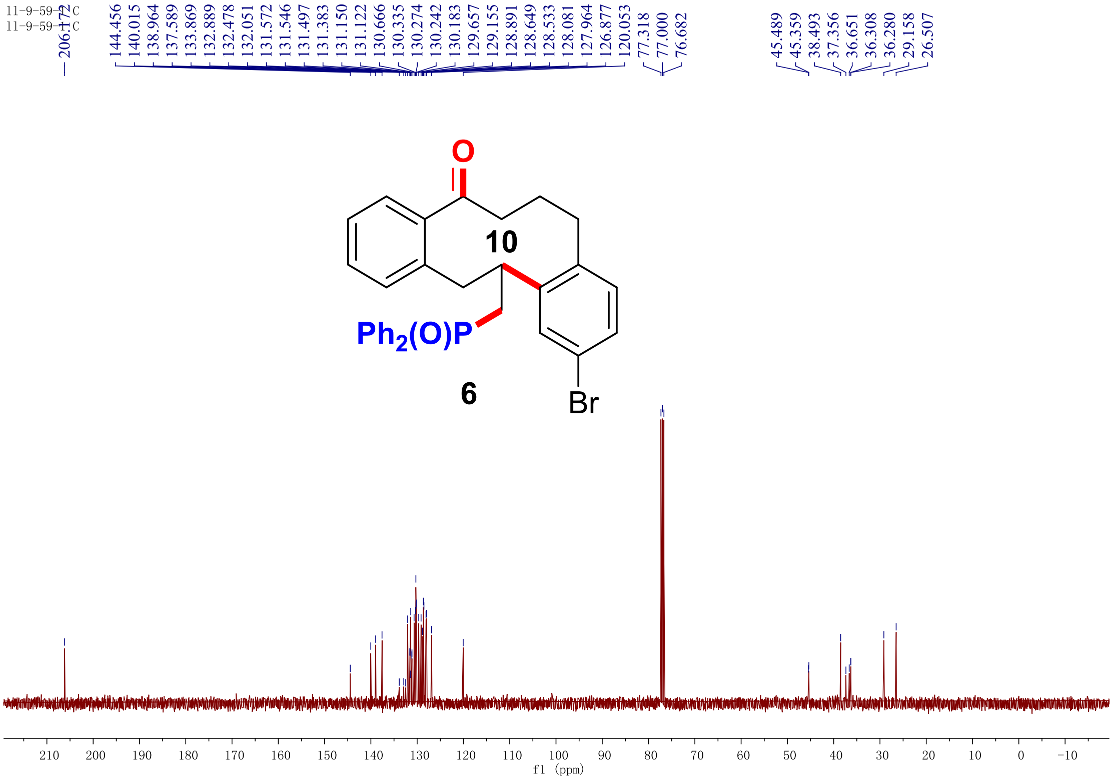

Supplementary Figure 150. ^{13}C NMR of **6**

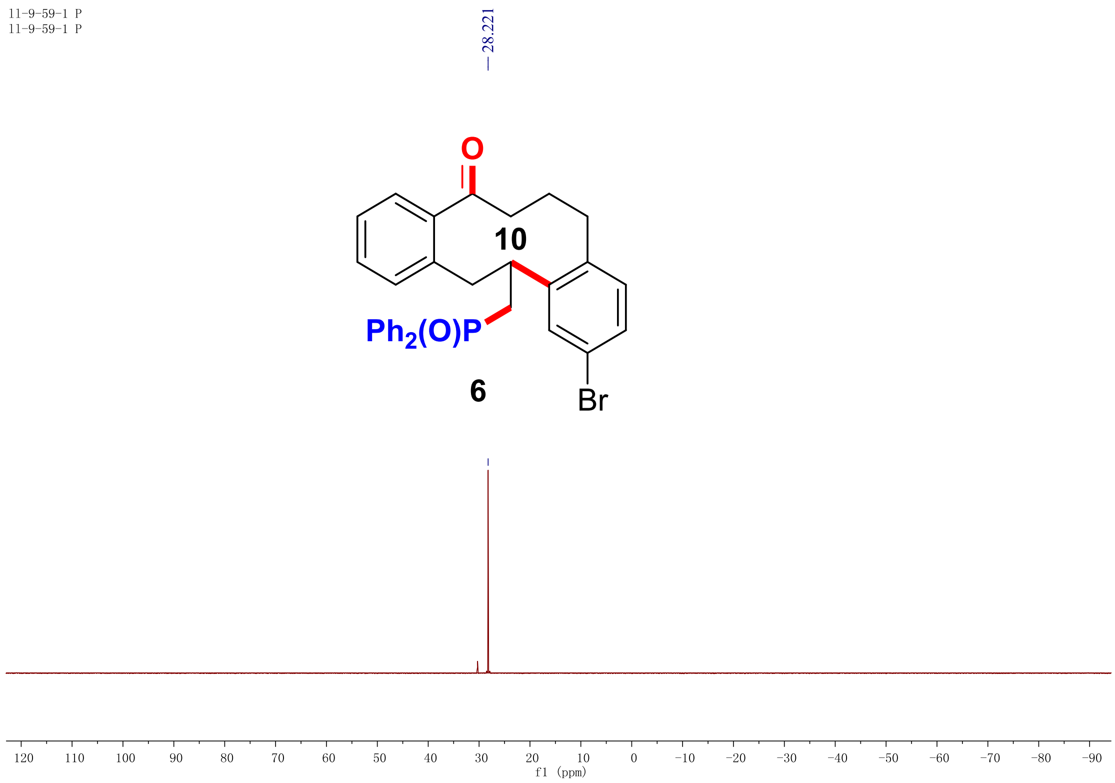

Supplementary Figure 151. ^{31}P NMR of **6**

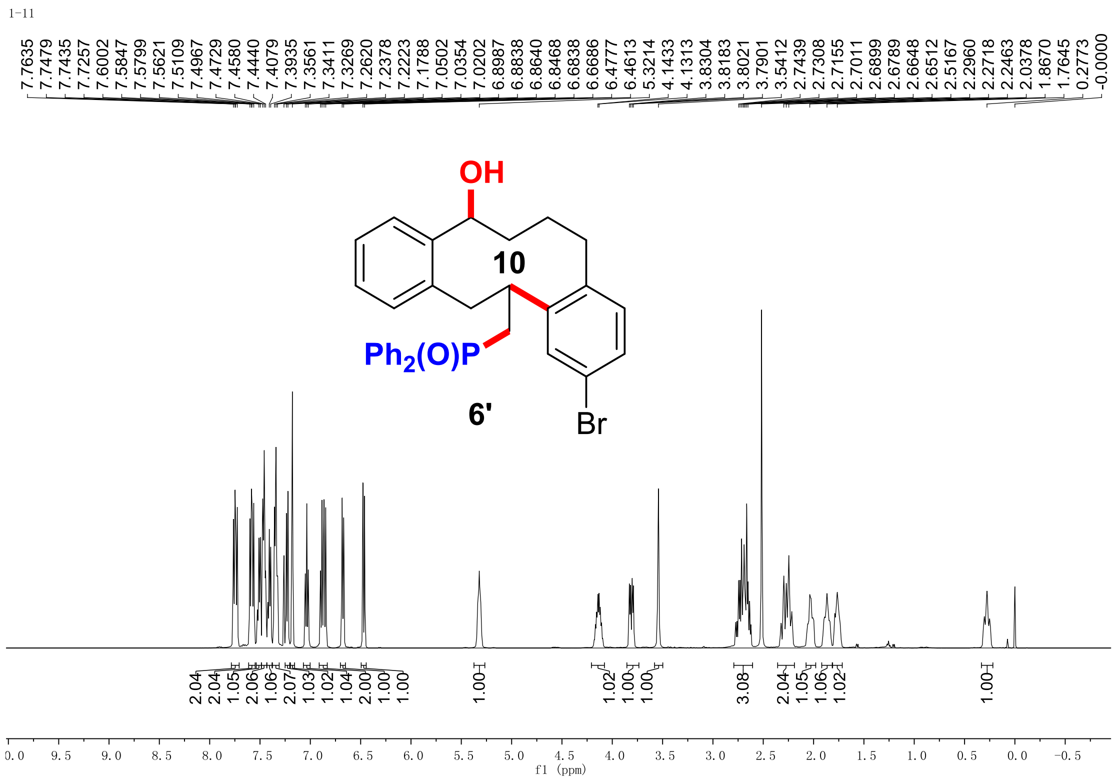

Supplementary Figure 152. ^{1}H NMR of **6’**

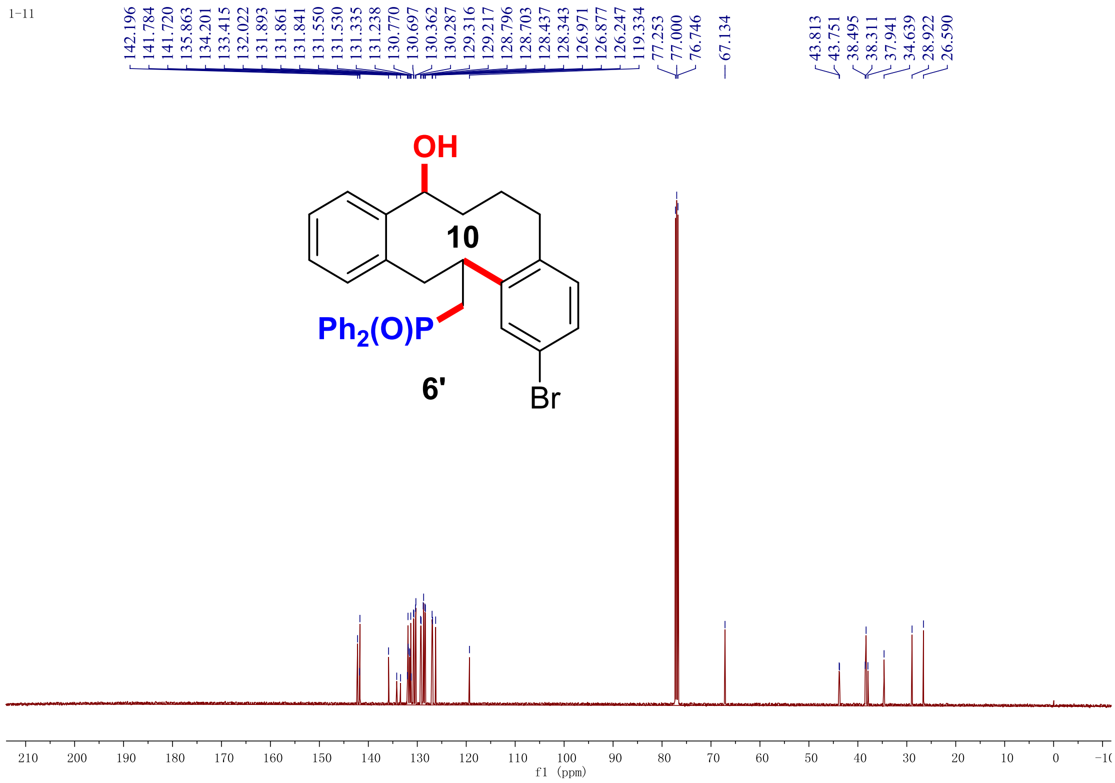

Supplementary Figure 153. ^{13}C NMR of **6’**

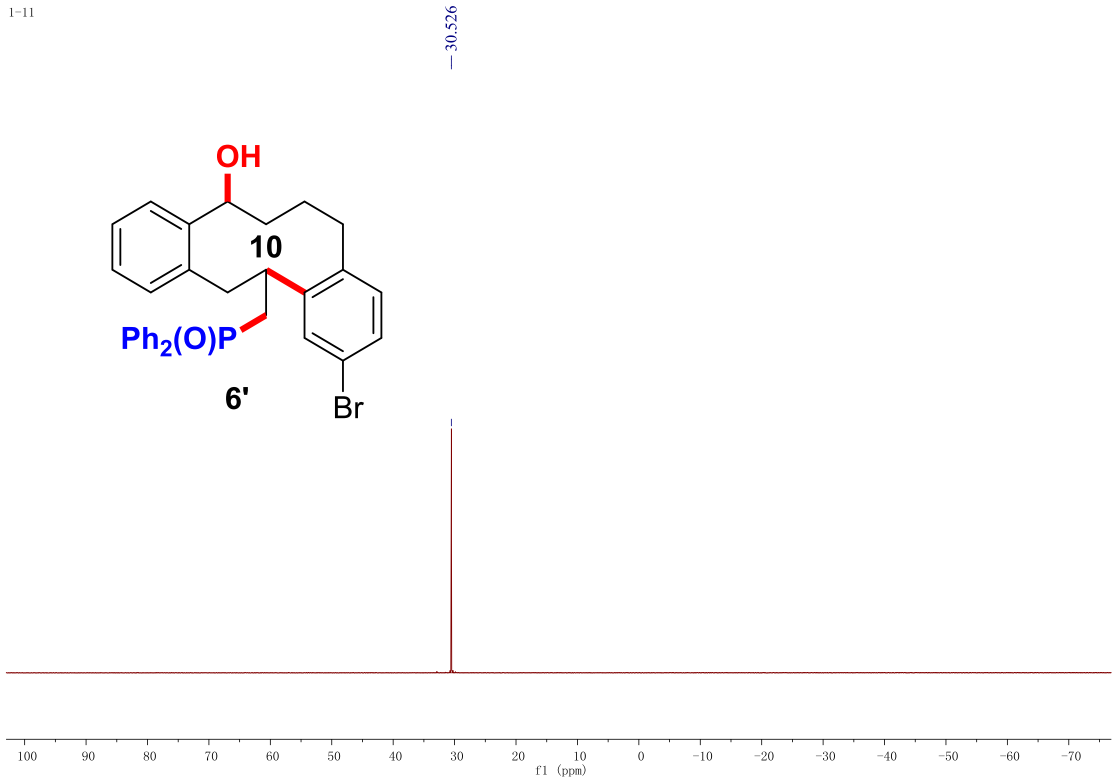

Supplementary Figure 154. ^{31}P NMR of **6’**

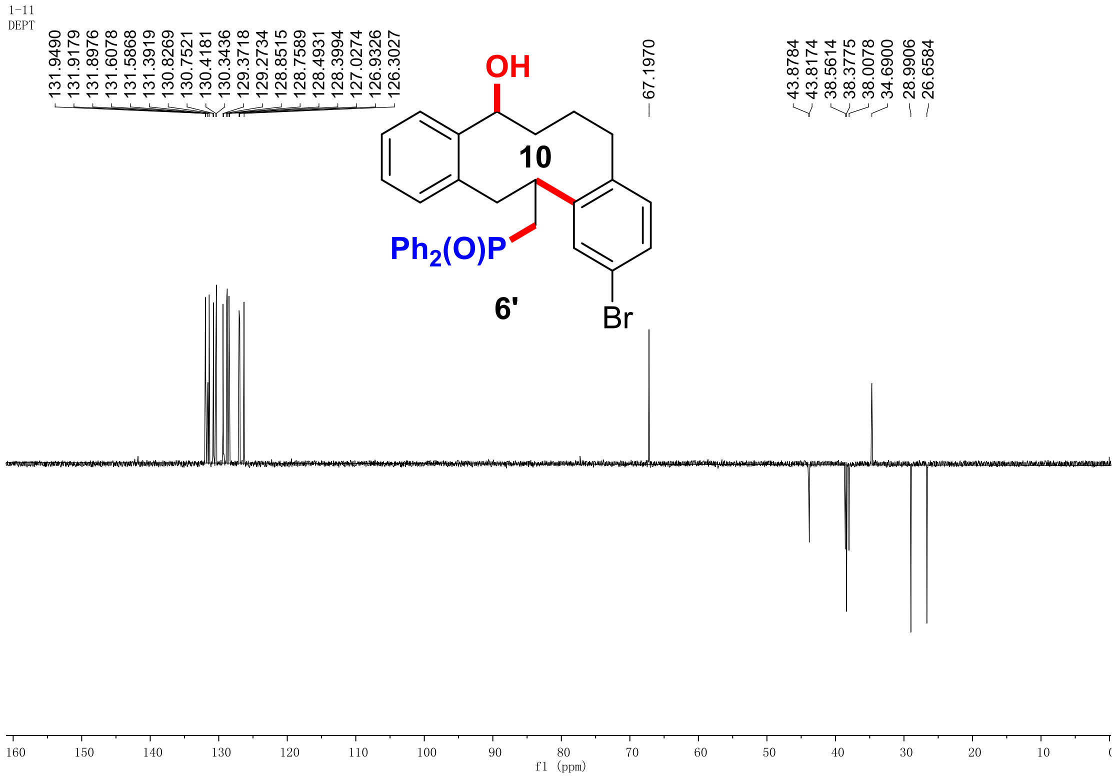

Supplementary Figure 155. DEPT135 of **6’**

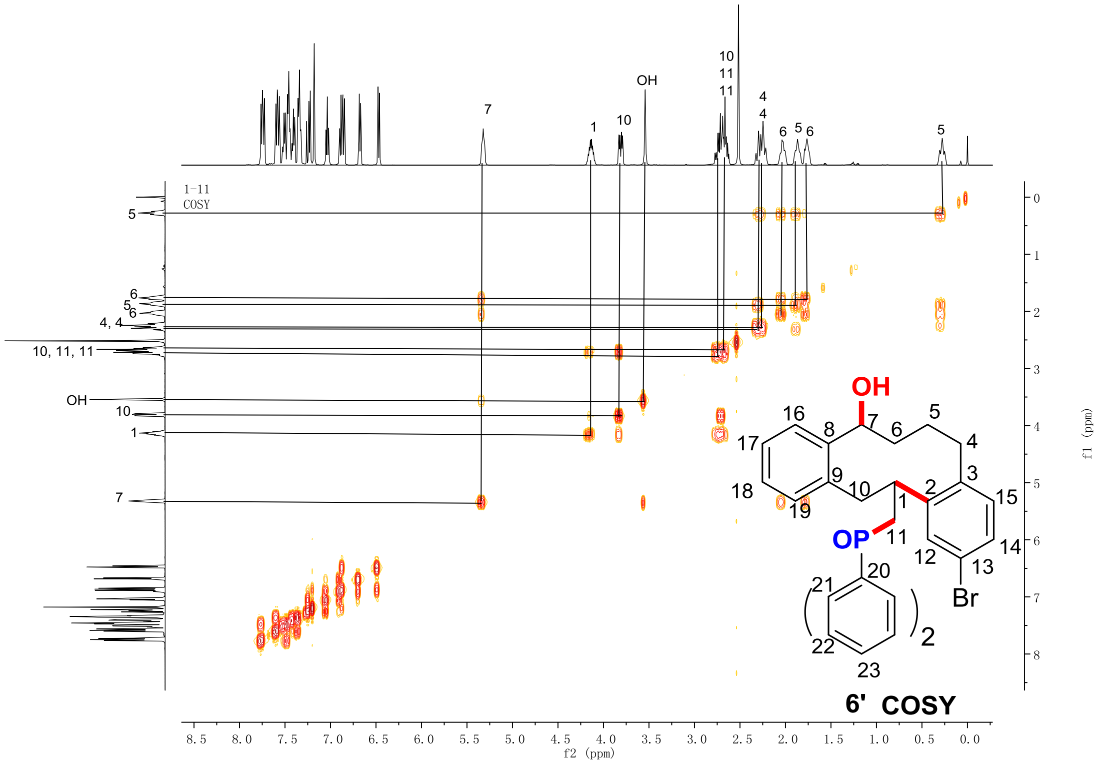

Supplementary Figure 156. COSY of **6’**

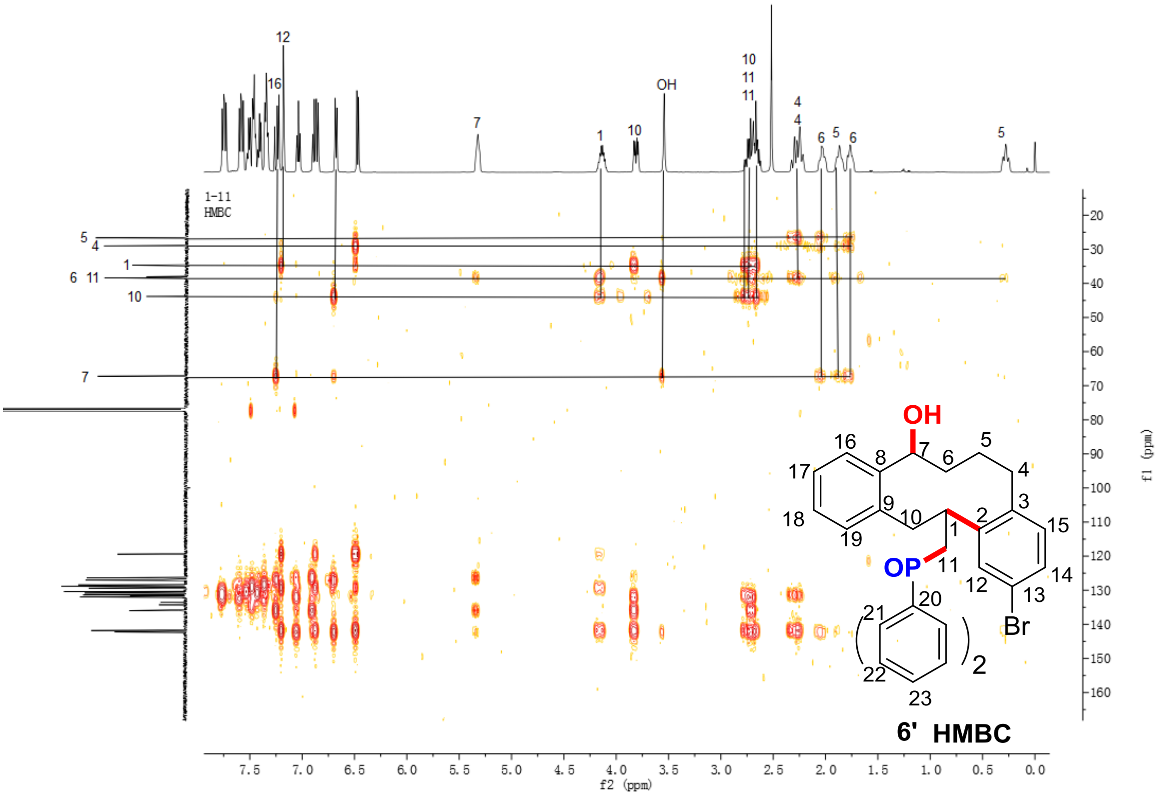

Supplementary Figure 157. HMBC of **6'**

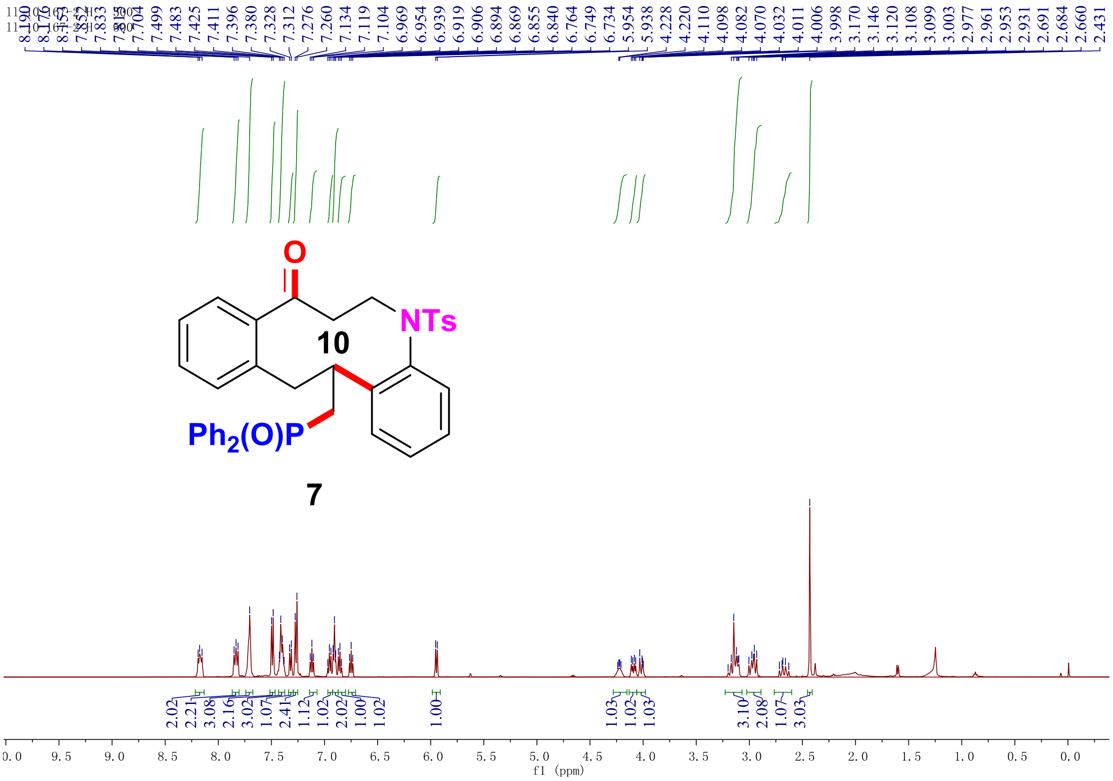

Supplementary Figure 158. ^{1}H NMR of **7**

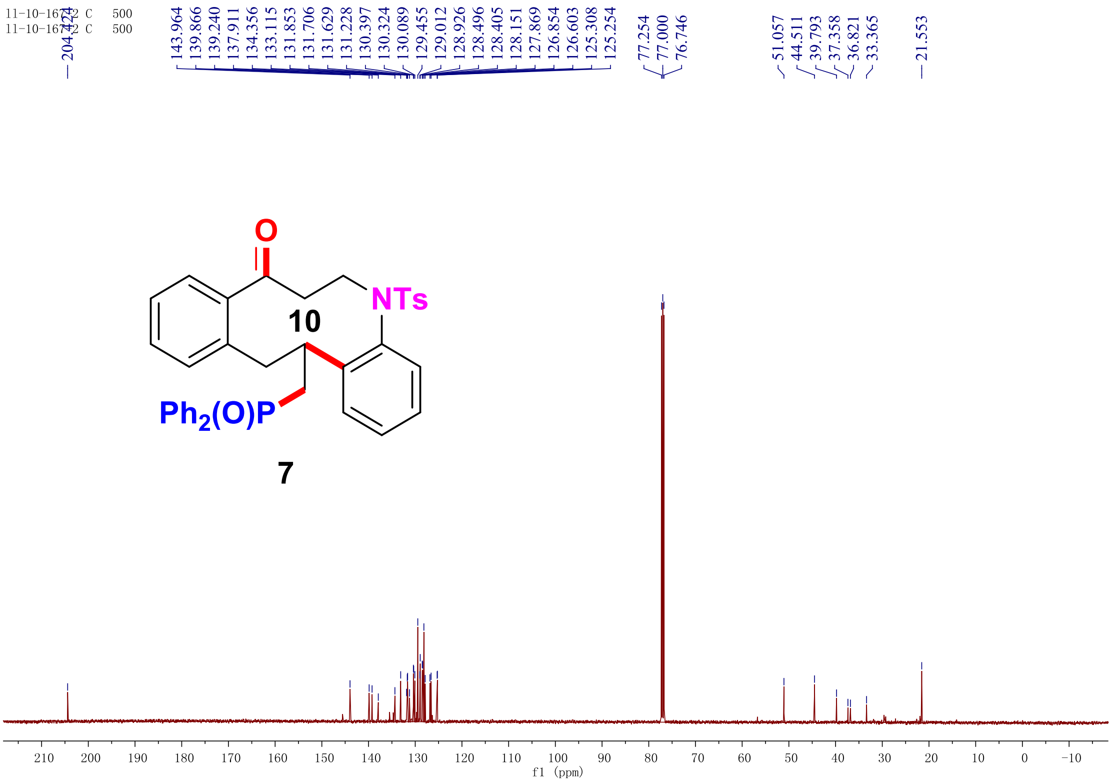

Supplementary Figure 159. ^{13}C NMR of **7**

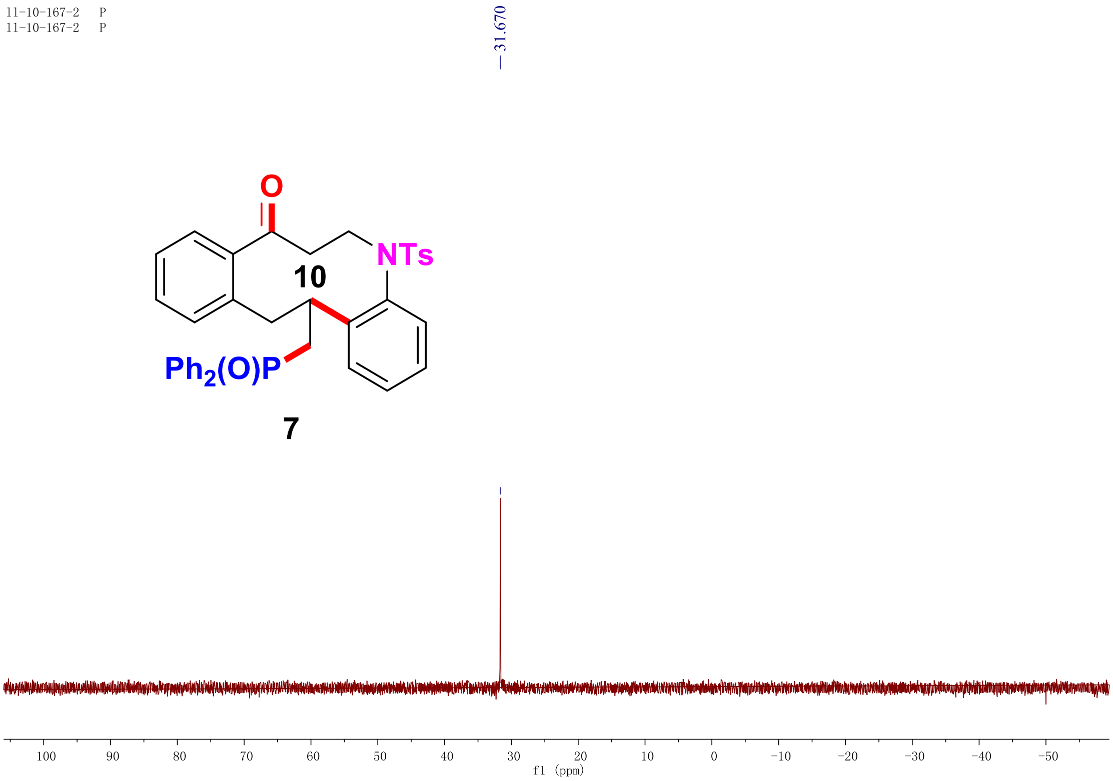

Supplementary Figure 160. ^{31}P NMR of **7**

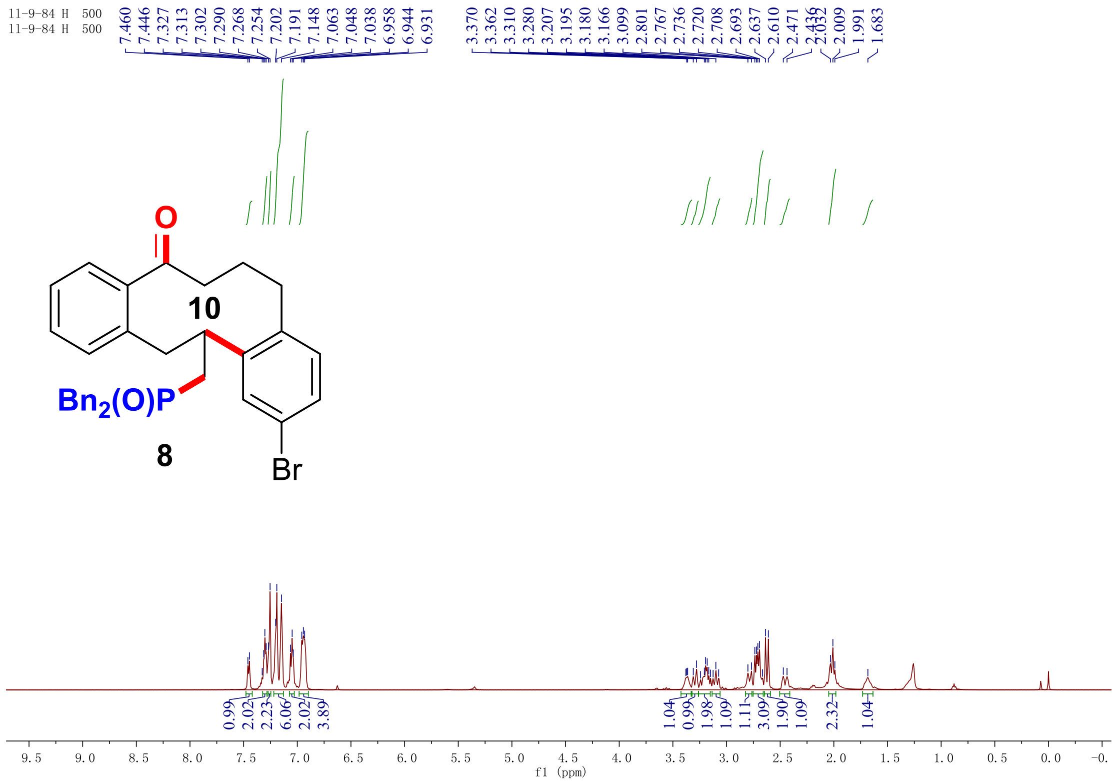

Supplementary Figure 161. ^{1}H NMR of **8**

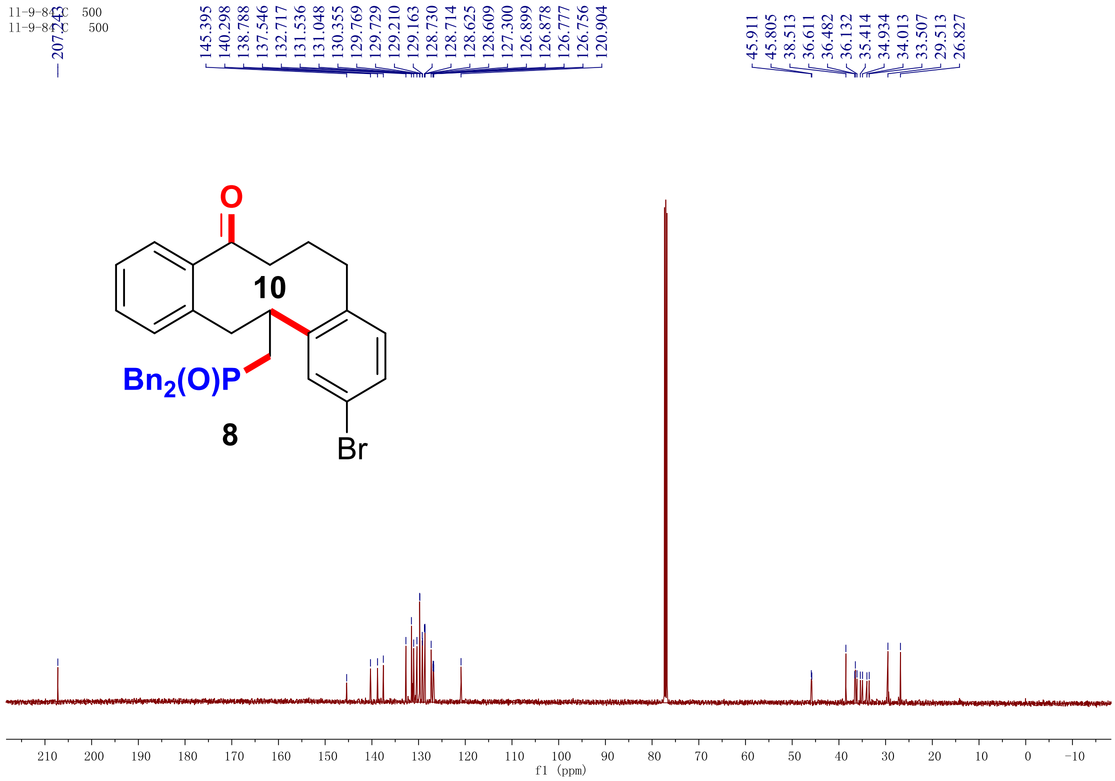

Supplementary Figure 162. ^{13}C NMR of **8**

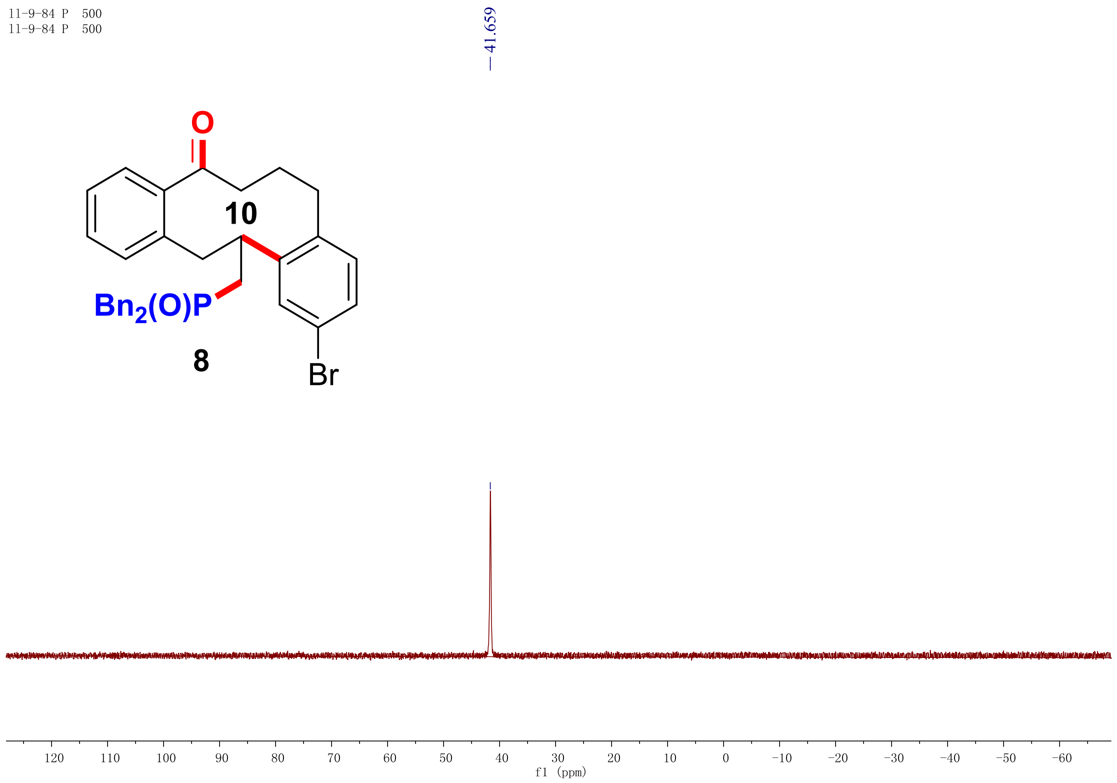

Supplementary Figure 163. ^{31}P NMR of **8**

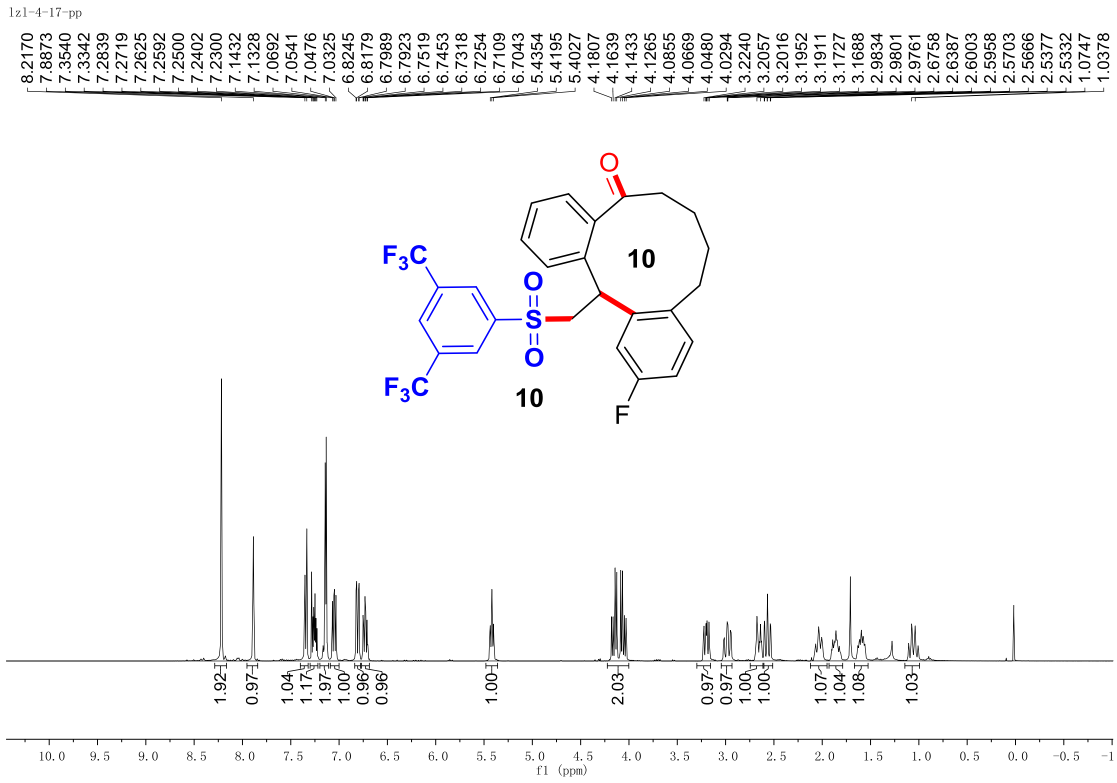

Supplementary Figure 164. ^{1}H NMR of **10**

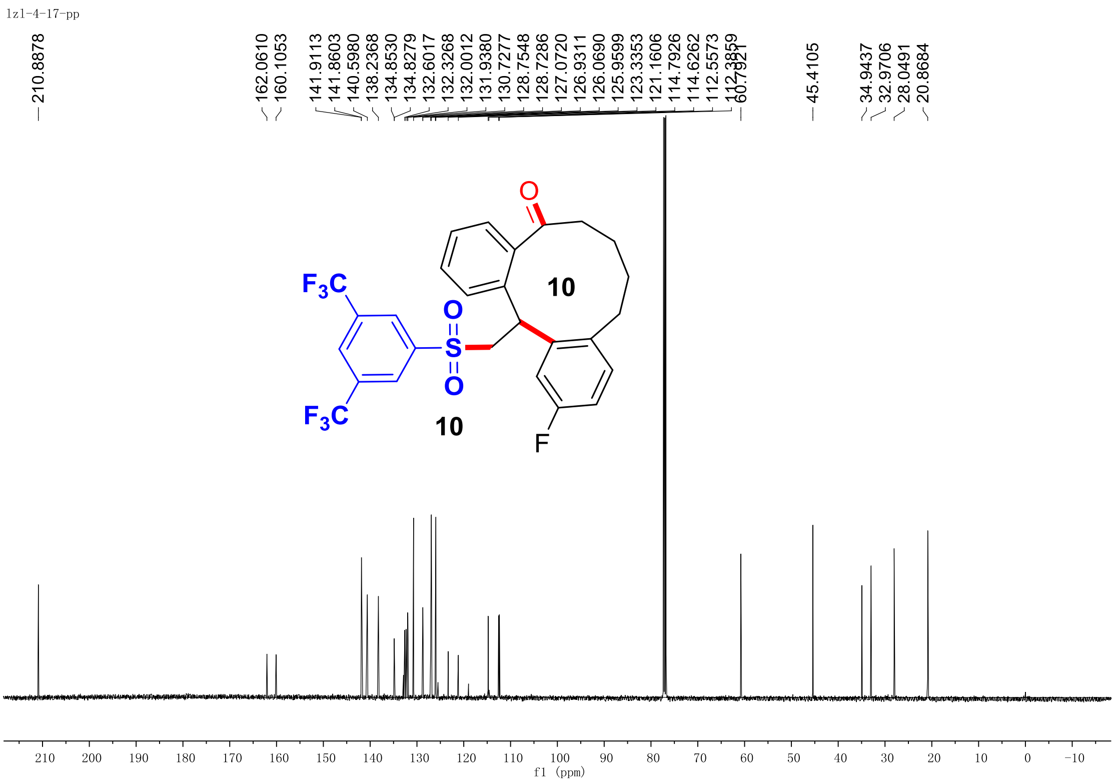

Supplementary Figure 165. ^{13}C NMR of **10**

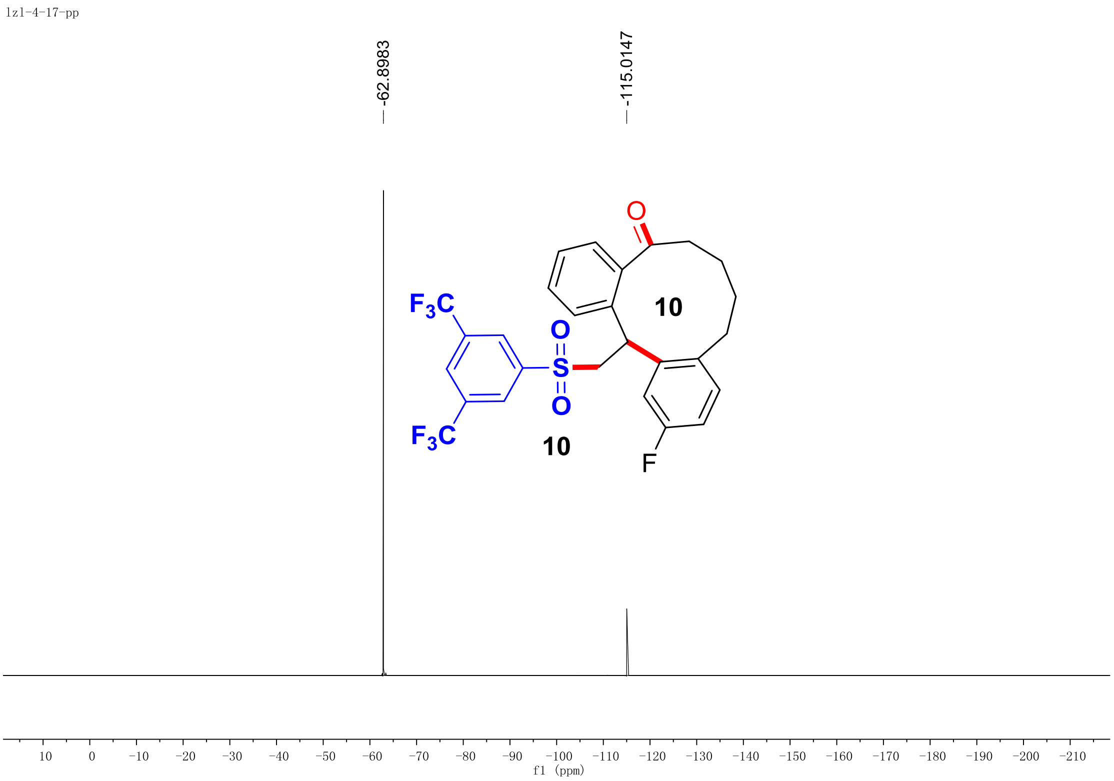

Supplementary Figure 166. ^{19}F NMR of **10**

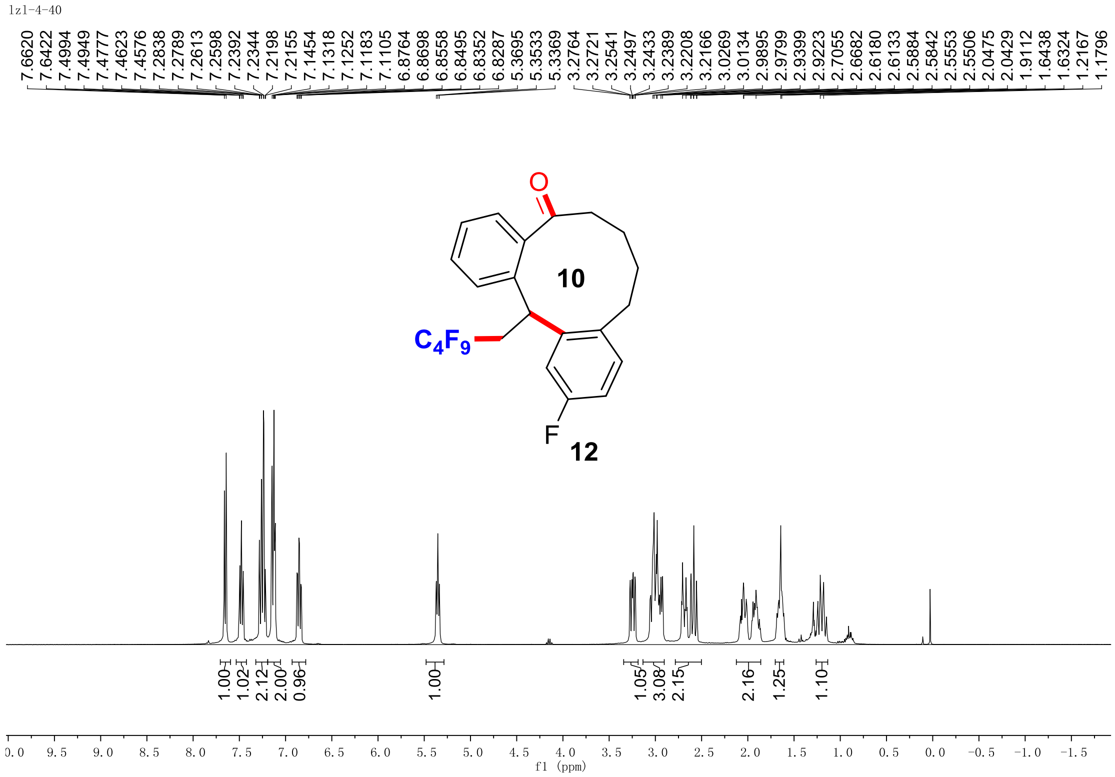

Supplementary Figure 167. ^{1}H NMR of **12**

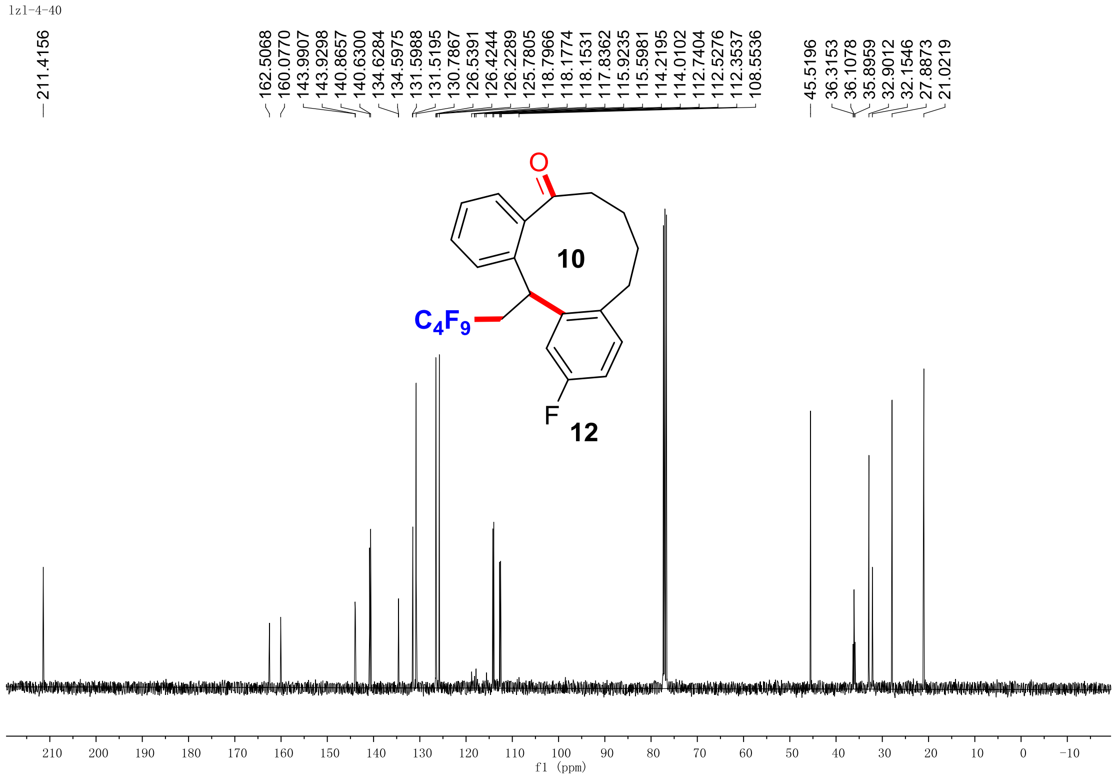

Supplementary Figure 168. ^{13}C NMR of **12**

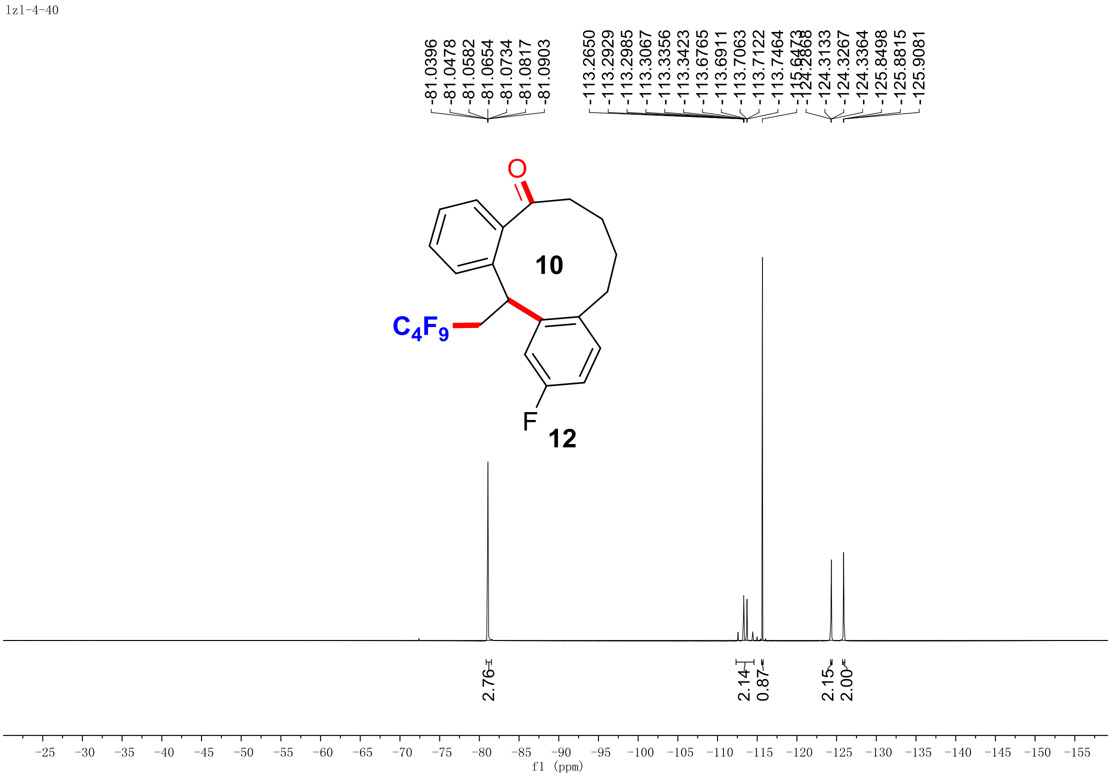

Supplementary Figure 169. ^{19}F NMR of **12**

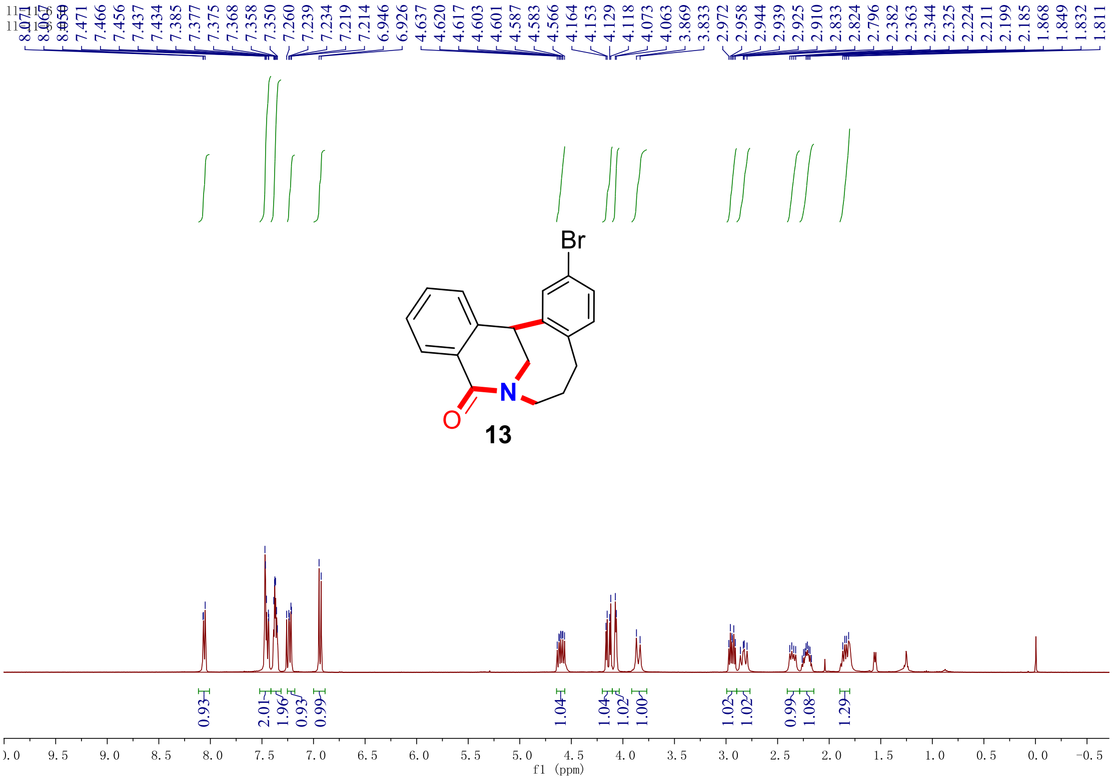

Supplementary Figure 170. ^{1}H NMR of **13**

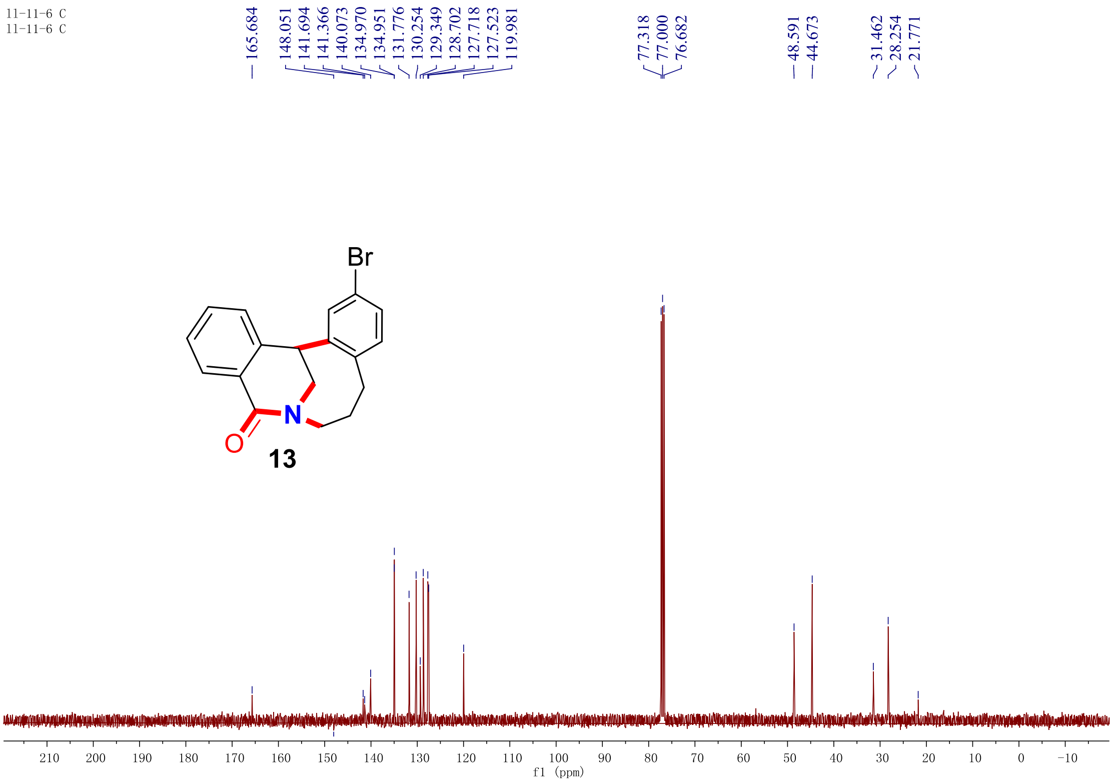

Supplementary Figure 171. ^{13}C NMR of **13**

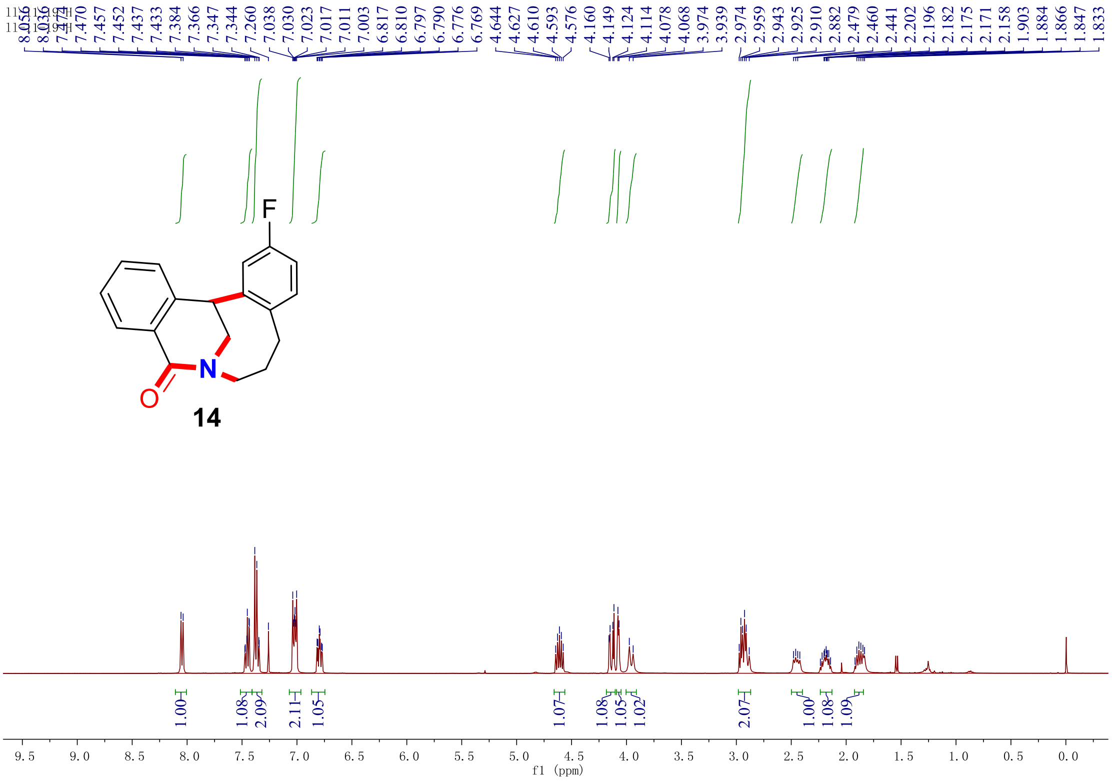

Supplementary Figure 172. ^{1}H NMR of **14**

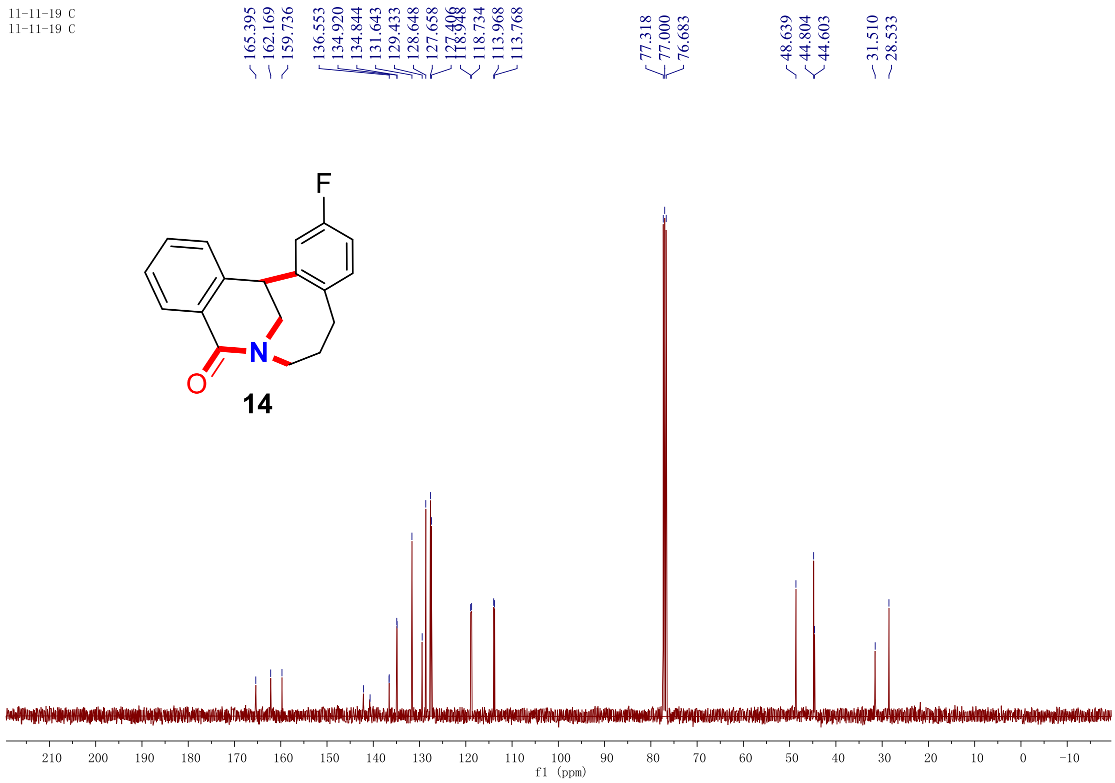

Supplementary Figure 173. ^{13}C NMR of **14**

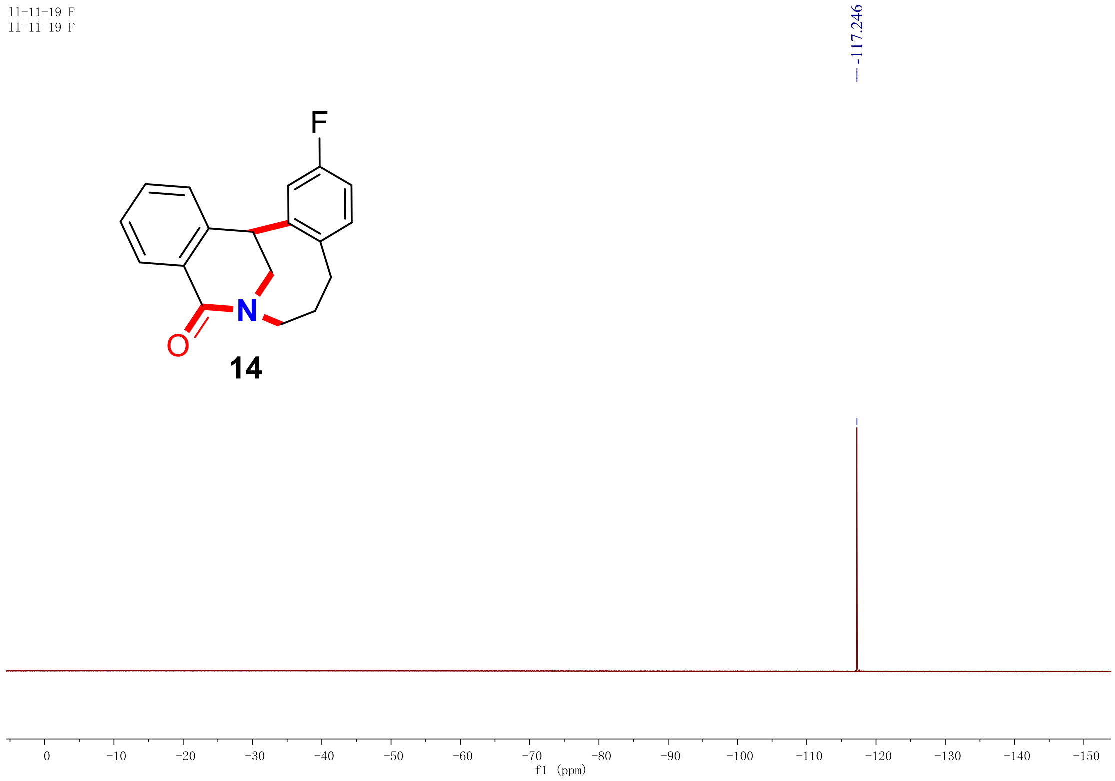

Supplementary Figure 174. ^{19}F NMR of **14**

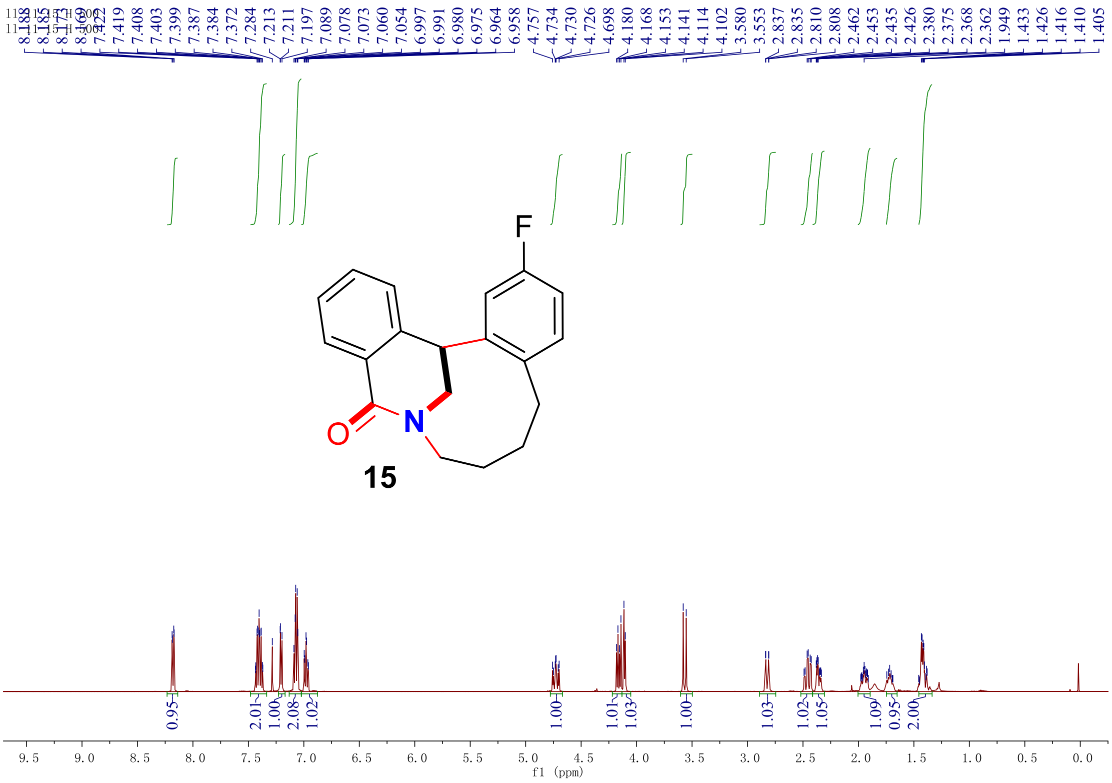

Supplementary Figure 175. ^{1}H NMR of **15**

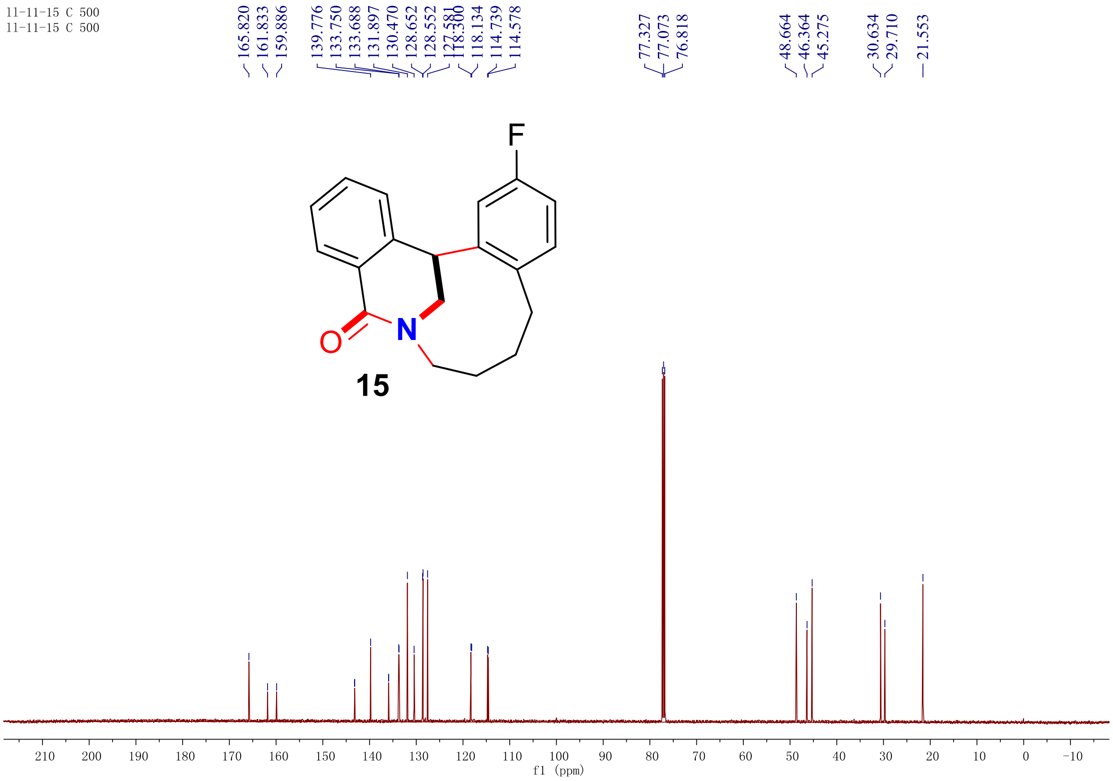

Supplementary Figure 176. ^{13}C NMR of **15**

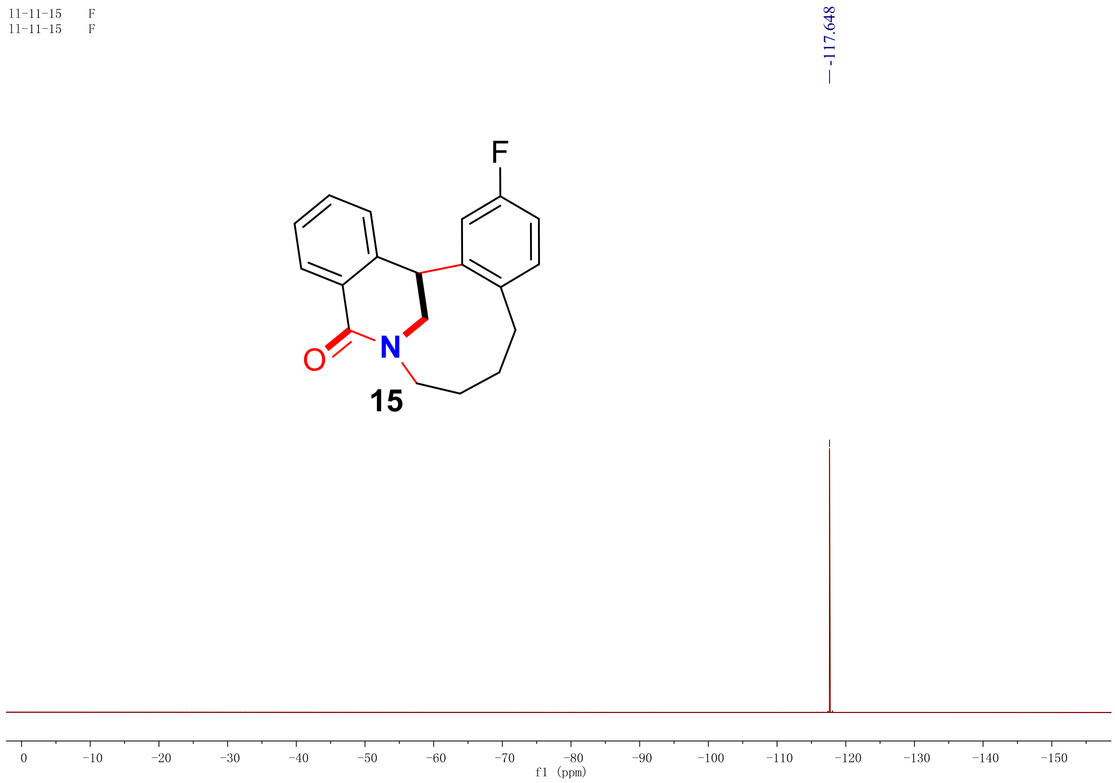

Supplementary Figure 177. ^{19}F NMR of **15**

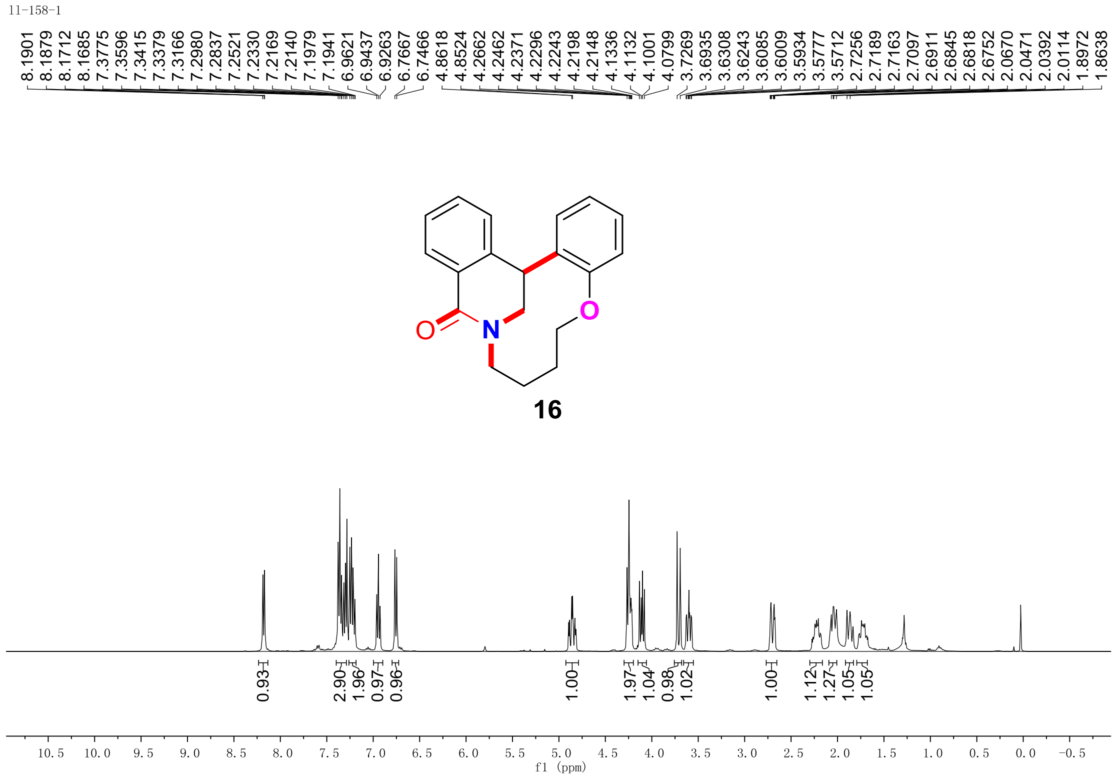

Supplementary Figure 178. ^{1}H NMR of **16**

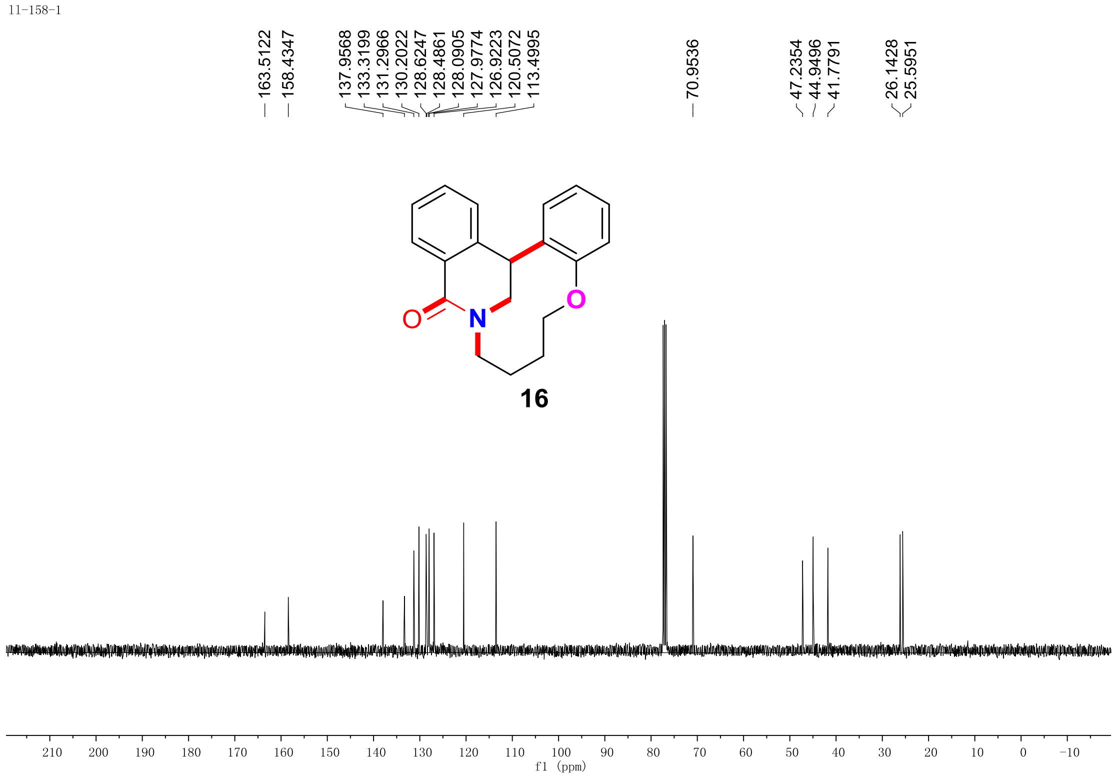

Supplementary Figure 179. ^{13}C NMR of **16**

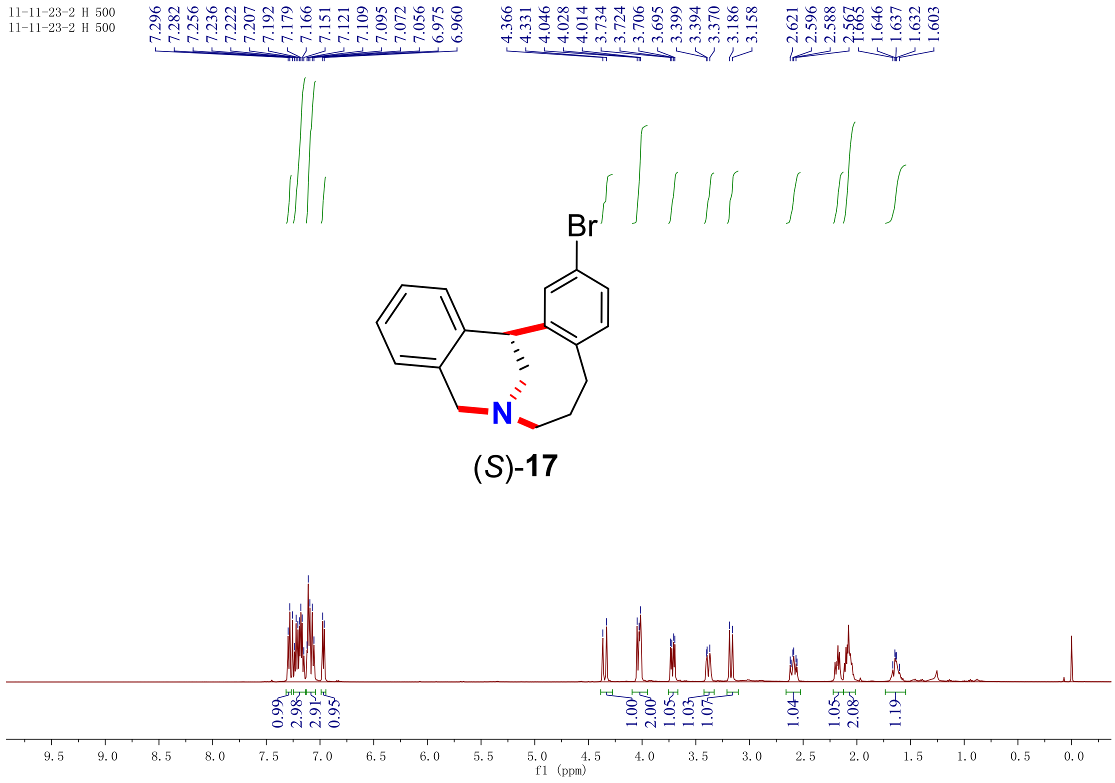

Supplementary Figure 180. ^{1}H NMR of (*S*)-**17**

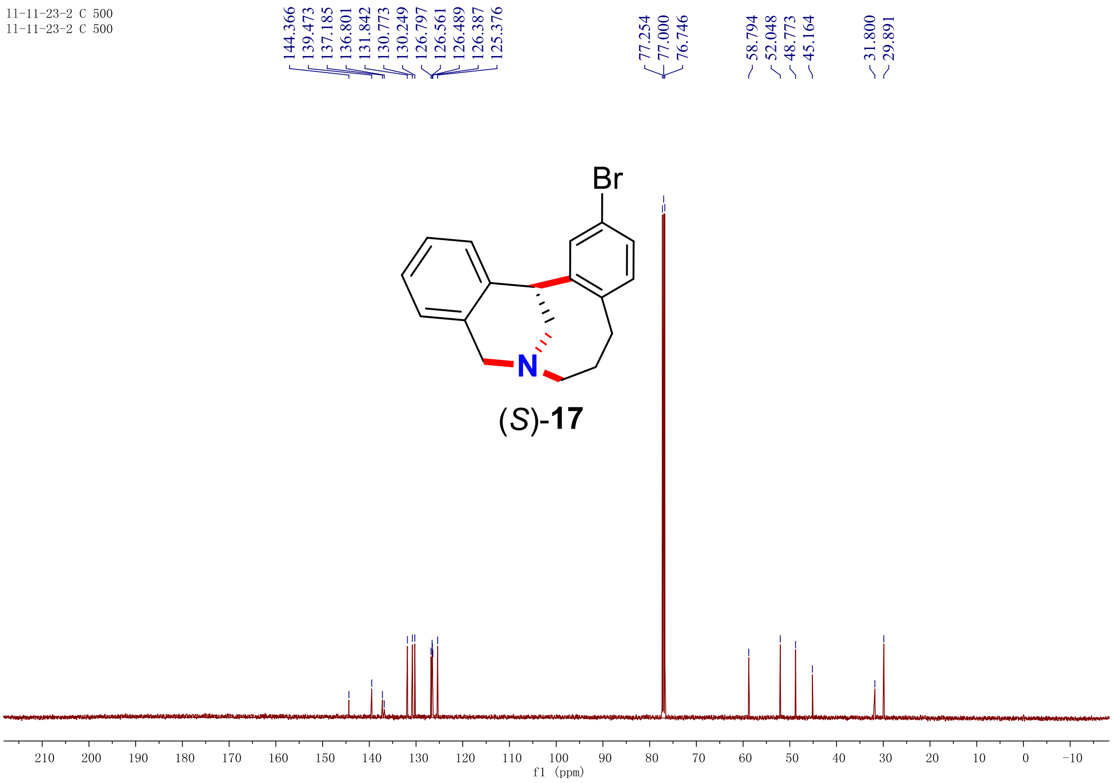

Supplementary Figure 181. ^{13}C NMR of (*S*)-**17**

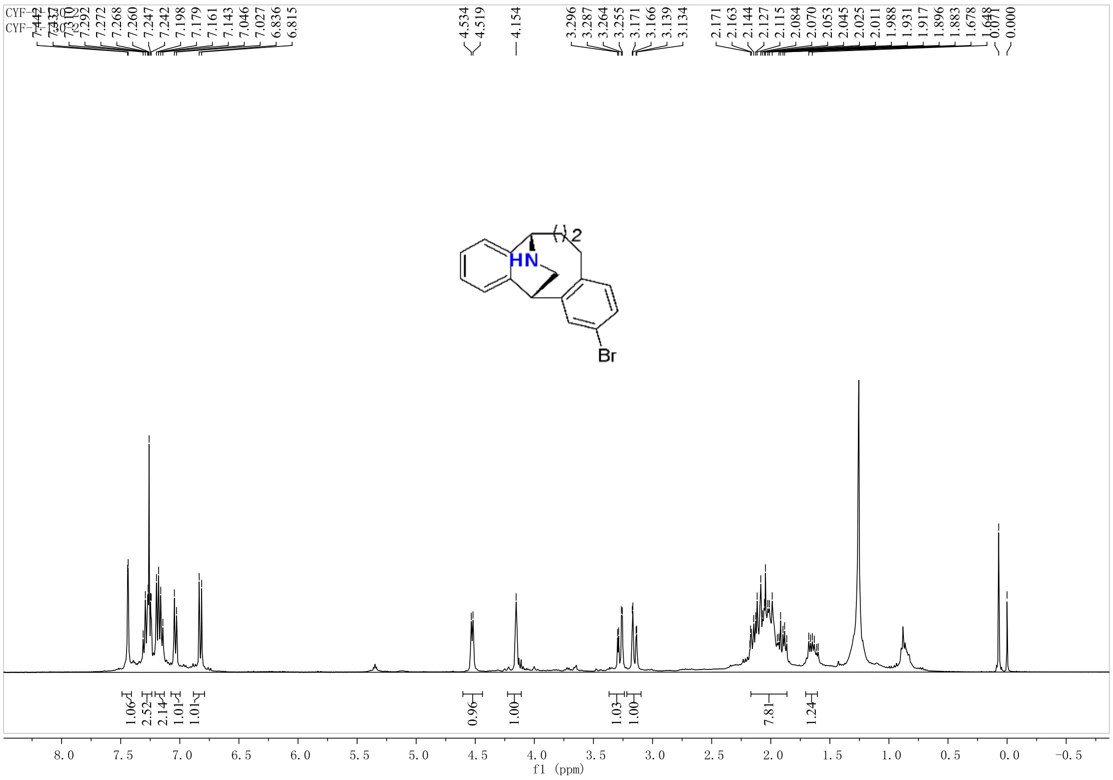

Supplementary Figure 182. ^{1}H NMR of **18**

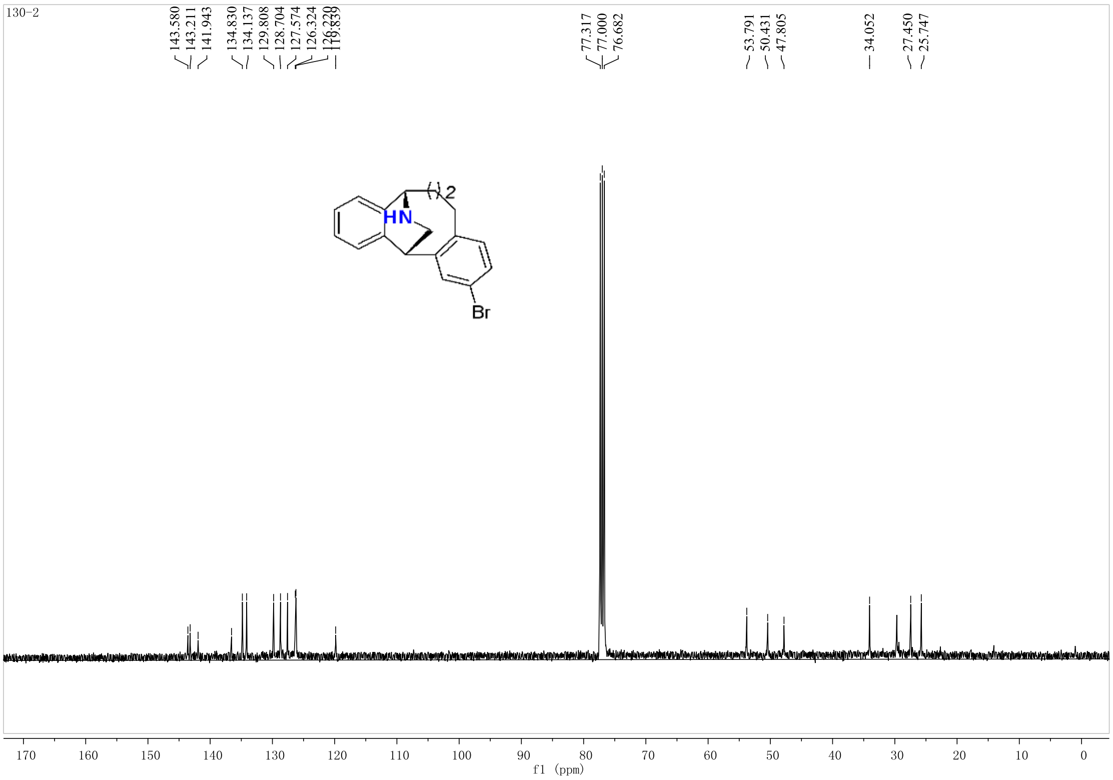

Supplementary Figure 183. ^{13}C NMR of **18**

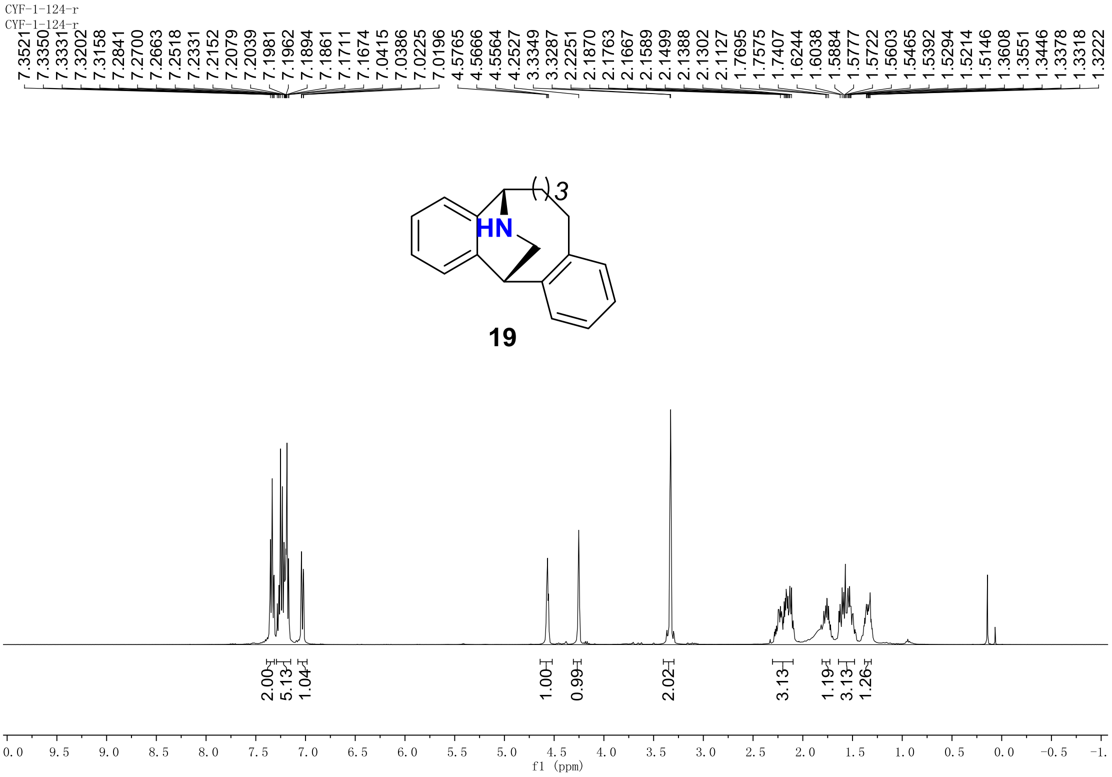

Supplementary Figure 184. ^{1}H NMR of **19**

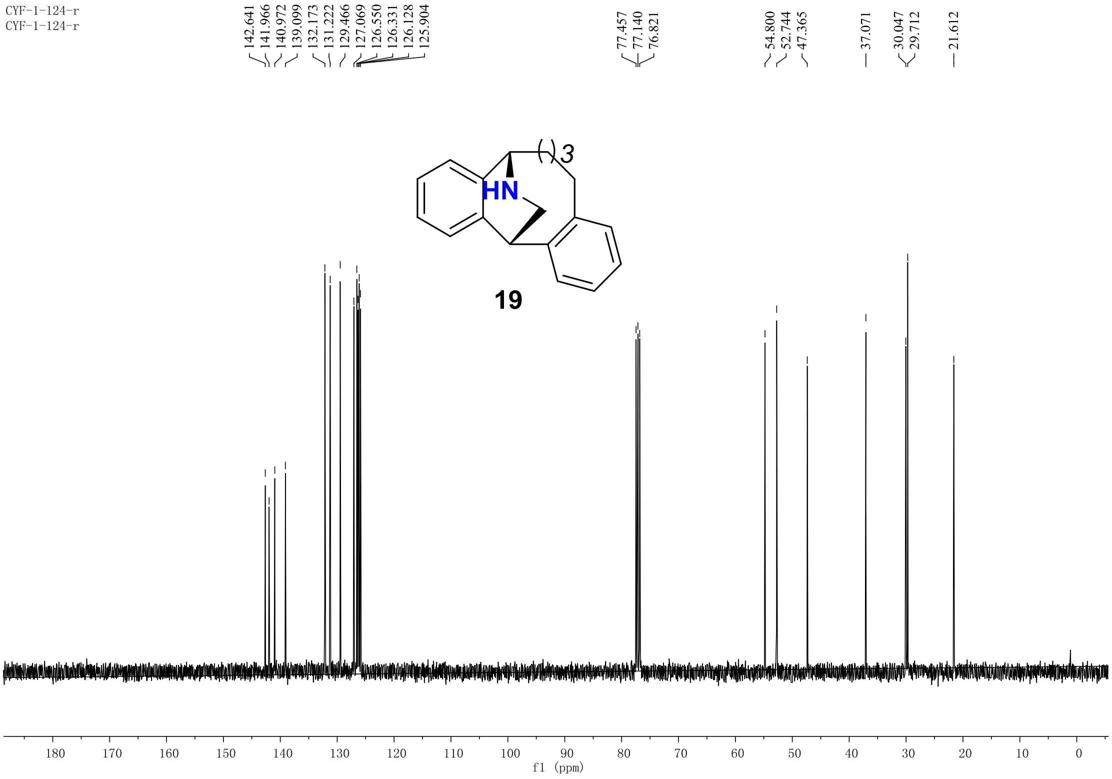

Supplementary Figure 185. ^{13}C NMR of **19**

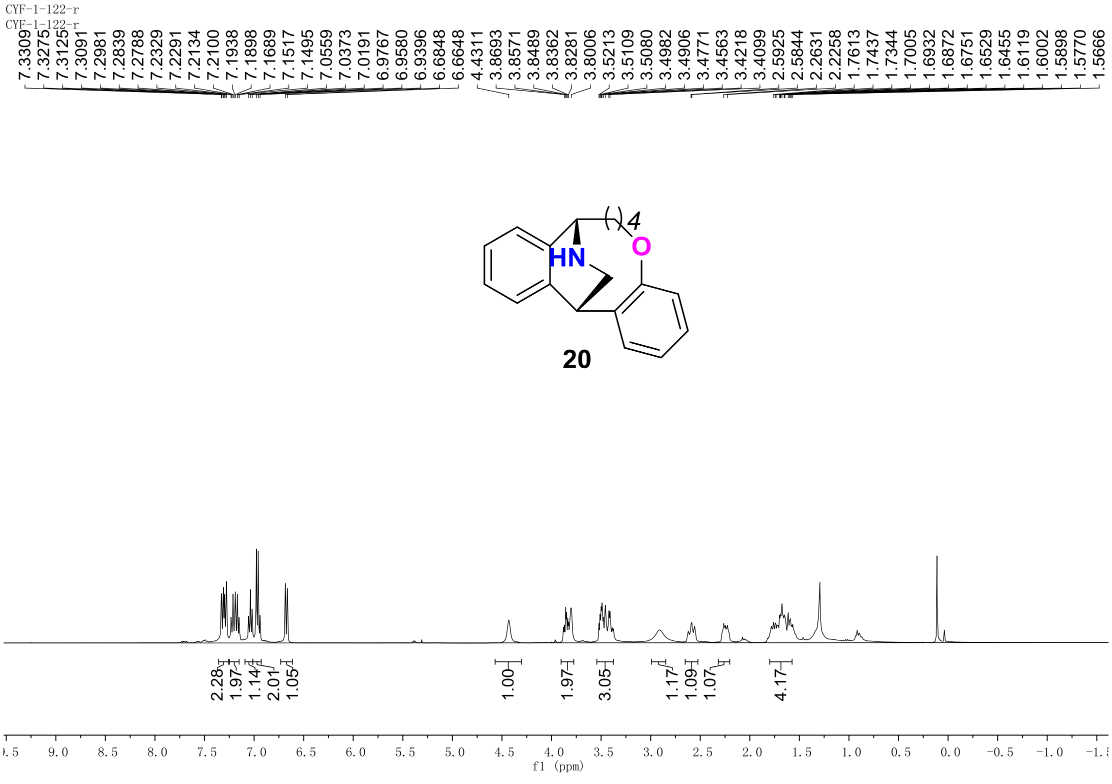

Supplementary Figure 186. ^{1}H NMR of **20**

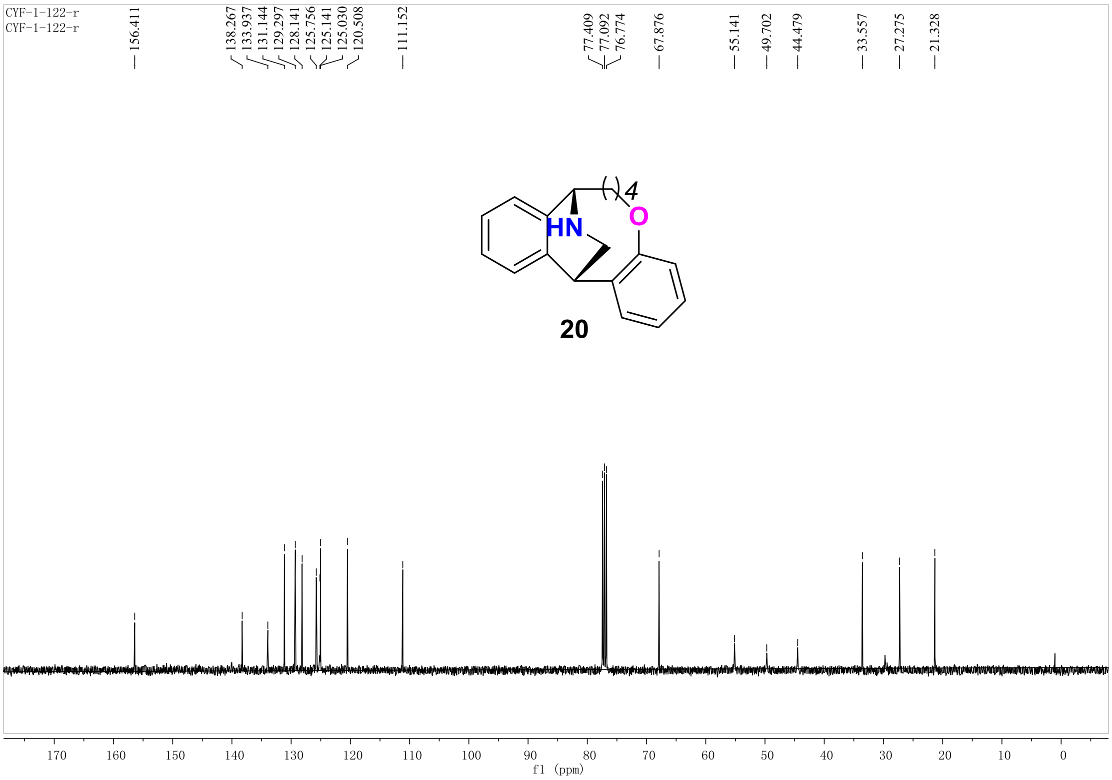

Supplementary Figure 187. ^{13}C NMR of **20**

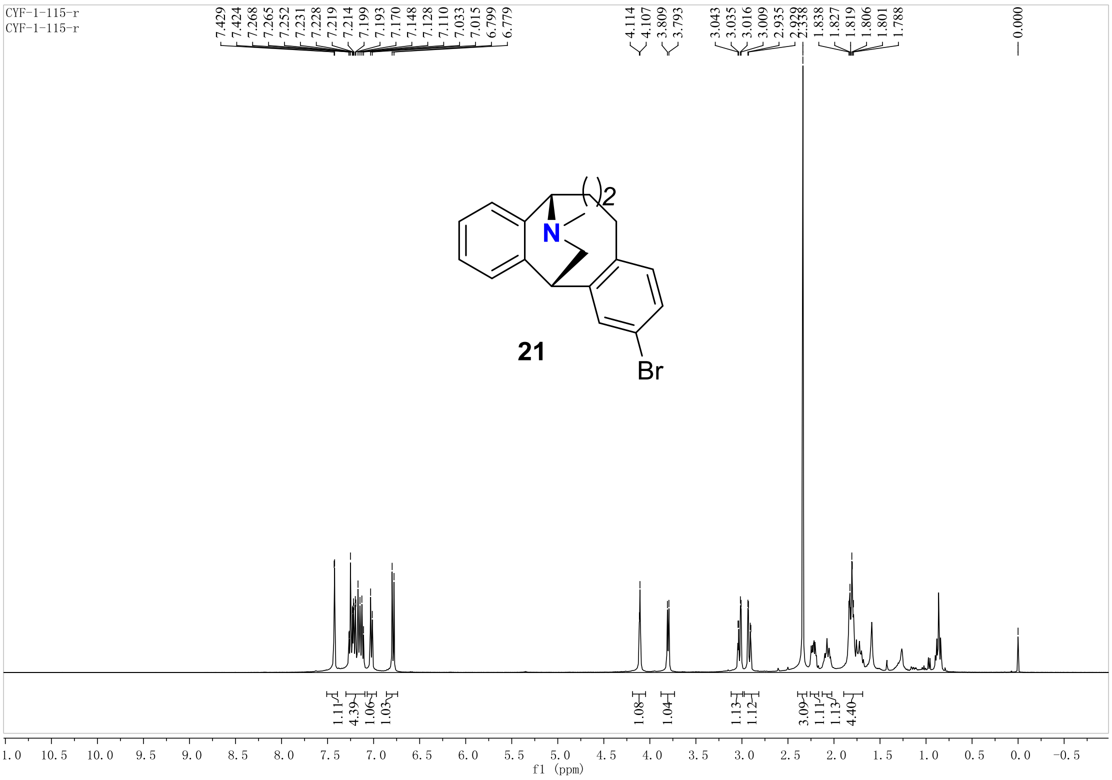

Supplementary Figure 188. ^{1}H NMR of **21**

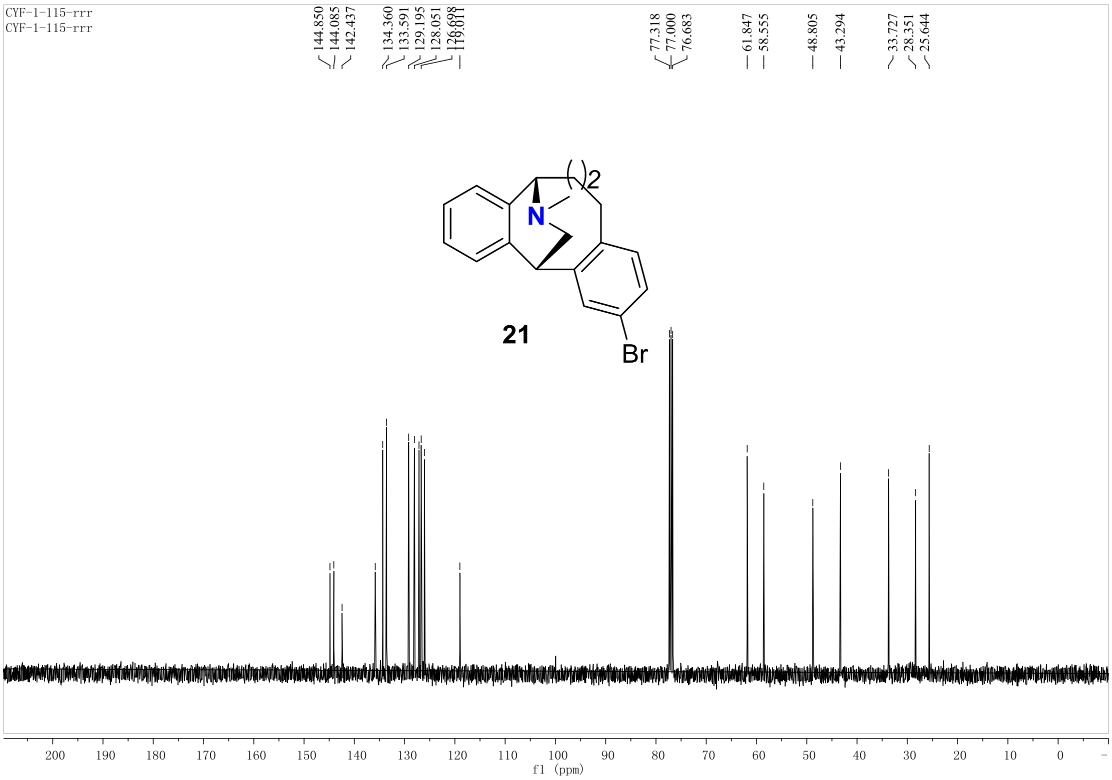

Supplementary Figure 189. ^{13}C NMR of **21**

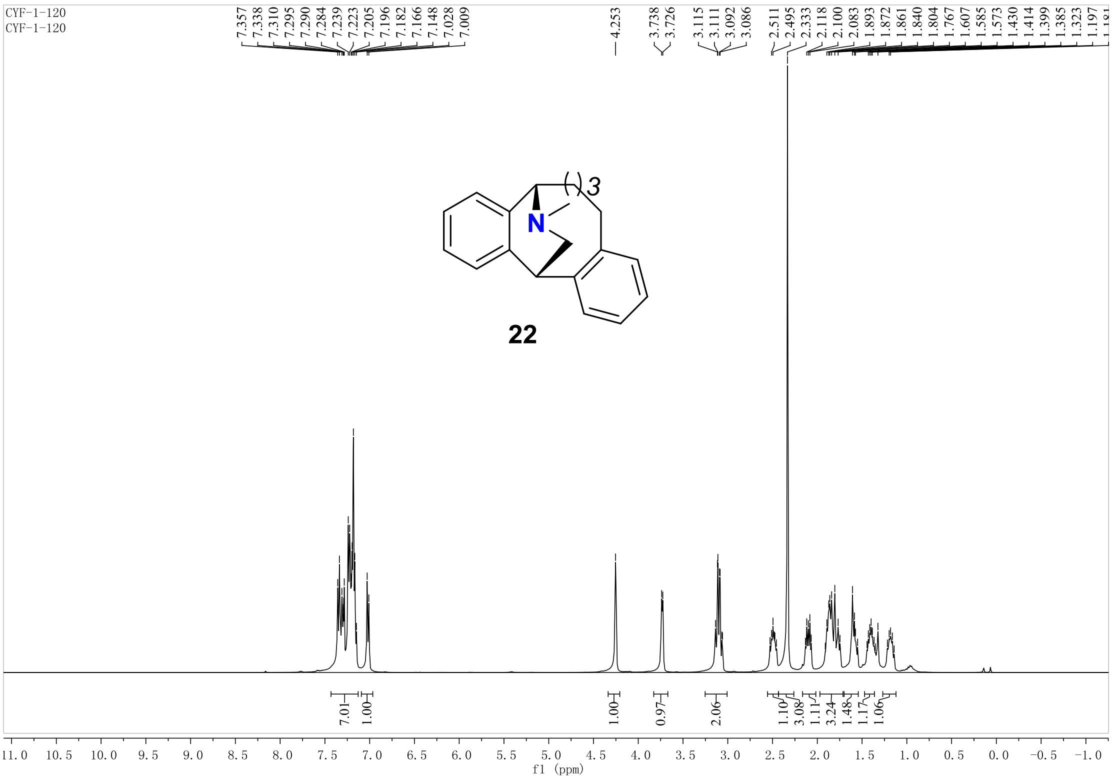

Supplementary Figure 190. ^{1}H NMR of **22**

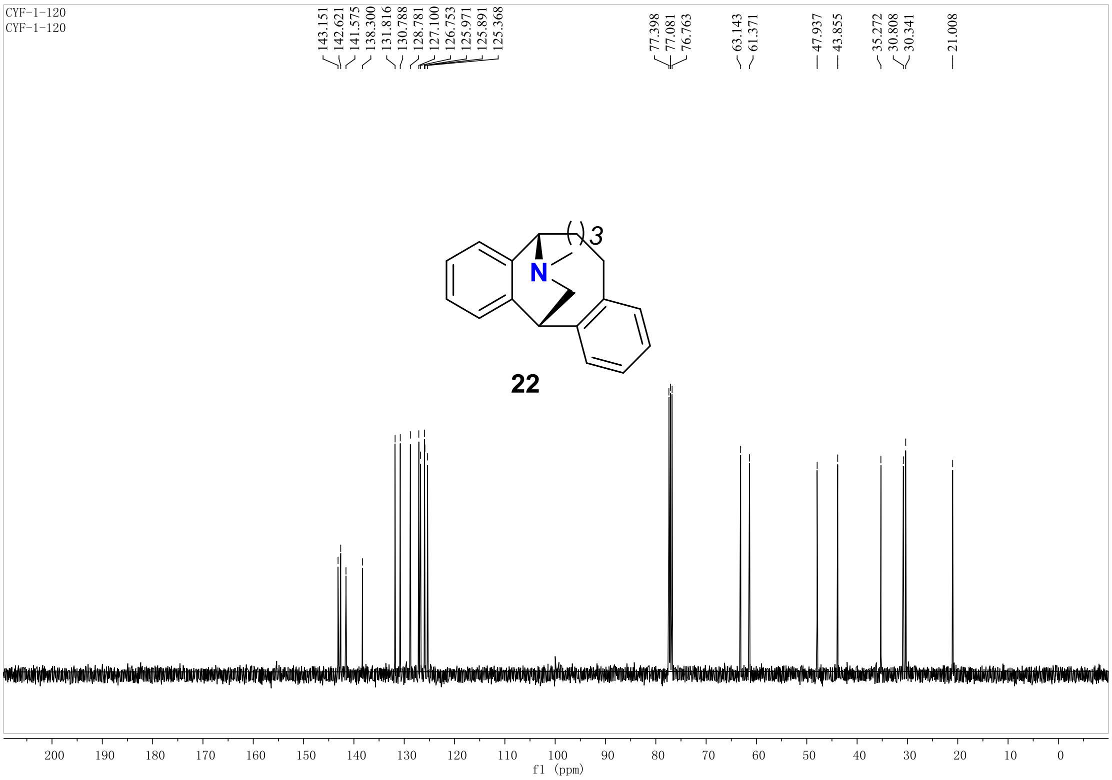

Supplementary Figure 191. ^{13}C NMR of **22**

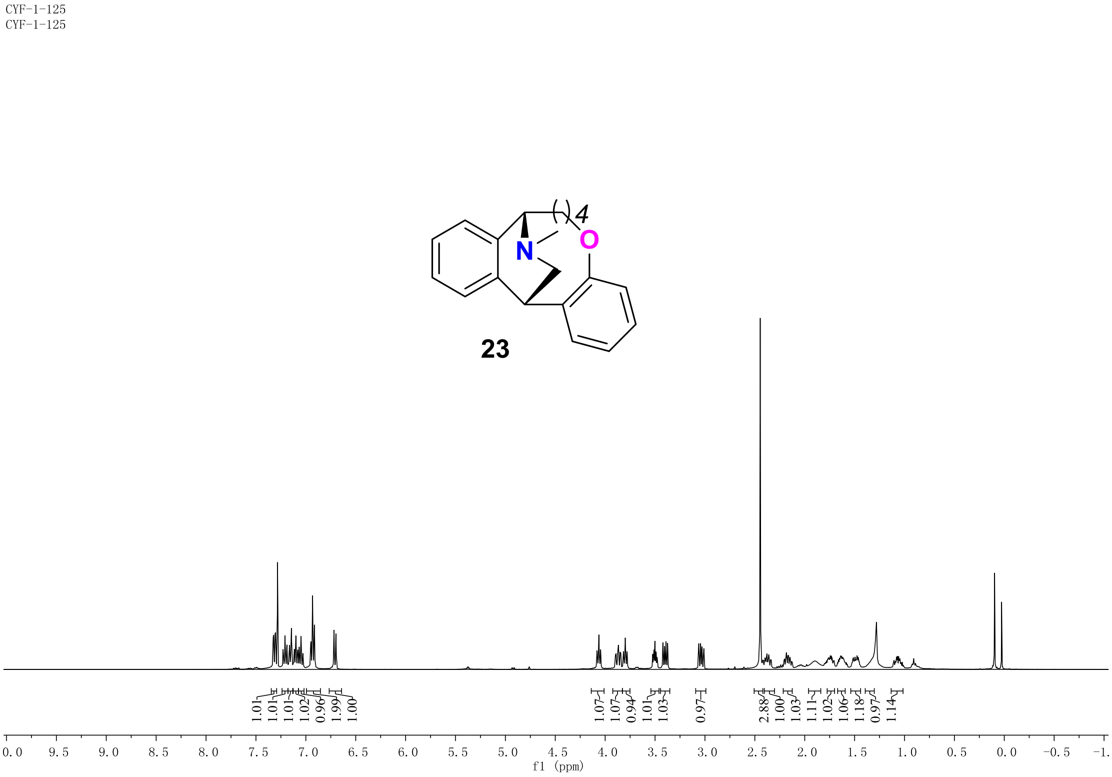

Supplementary Figure 192. ^{1}H NMR of **23**

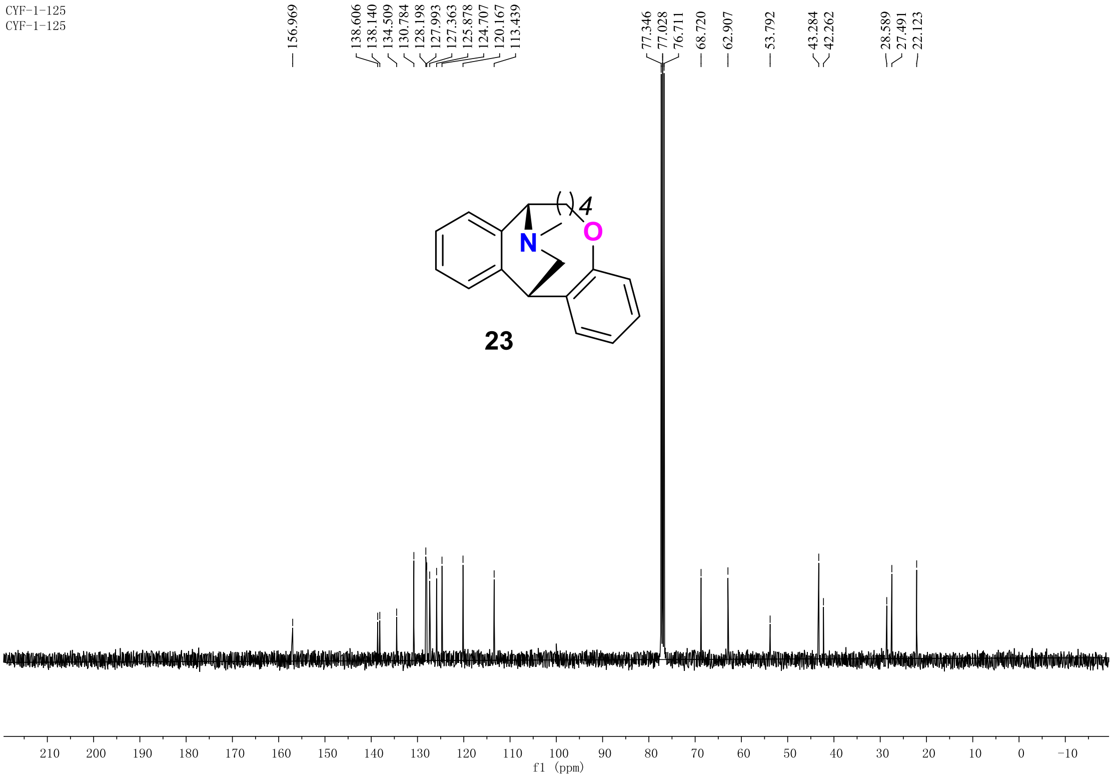

Supplementary Figure 193. ^{13}C NMR of **23**

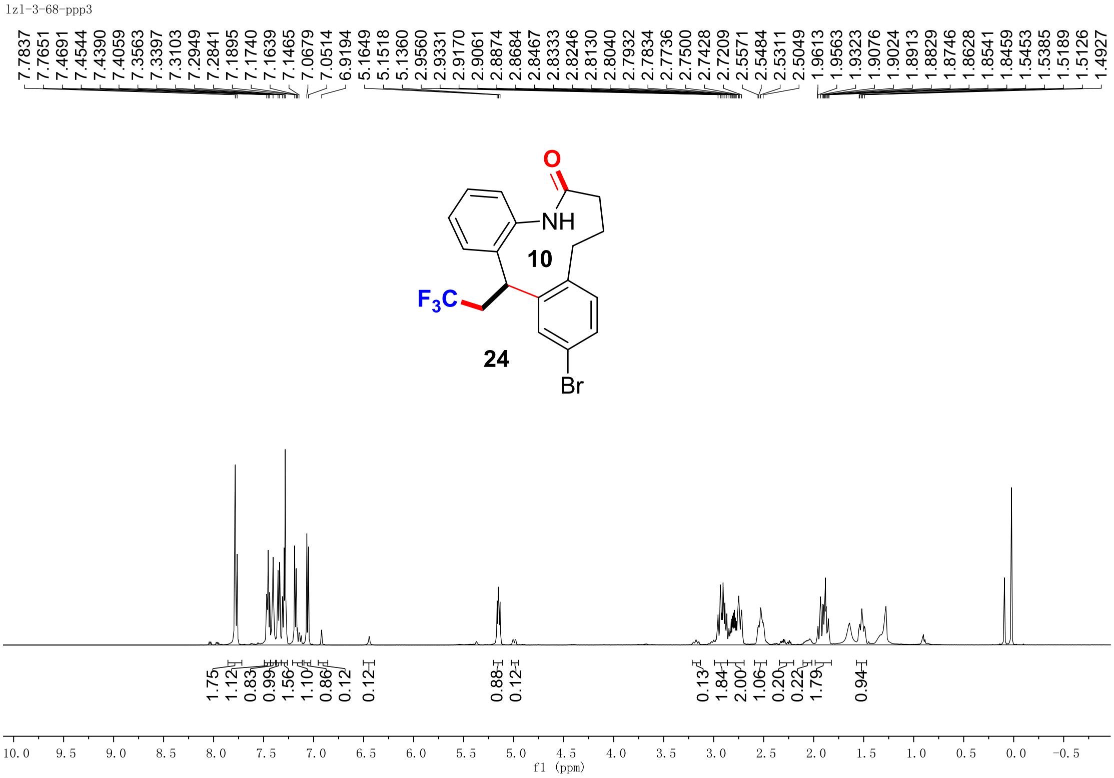

Supplementary Figure 194. ^{1}H NMR of **24**

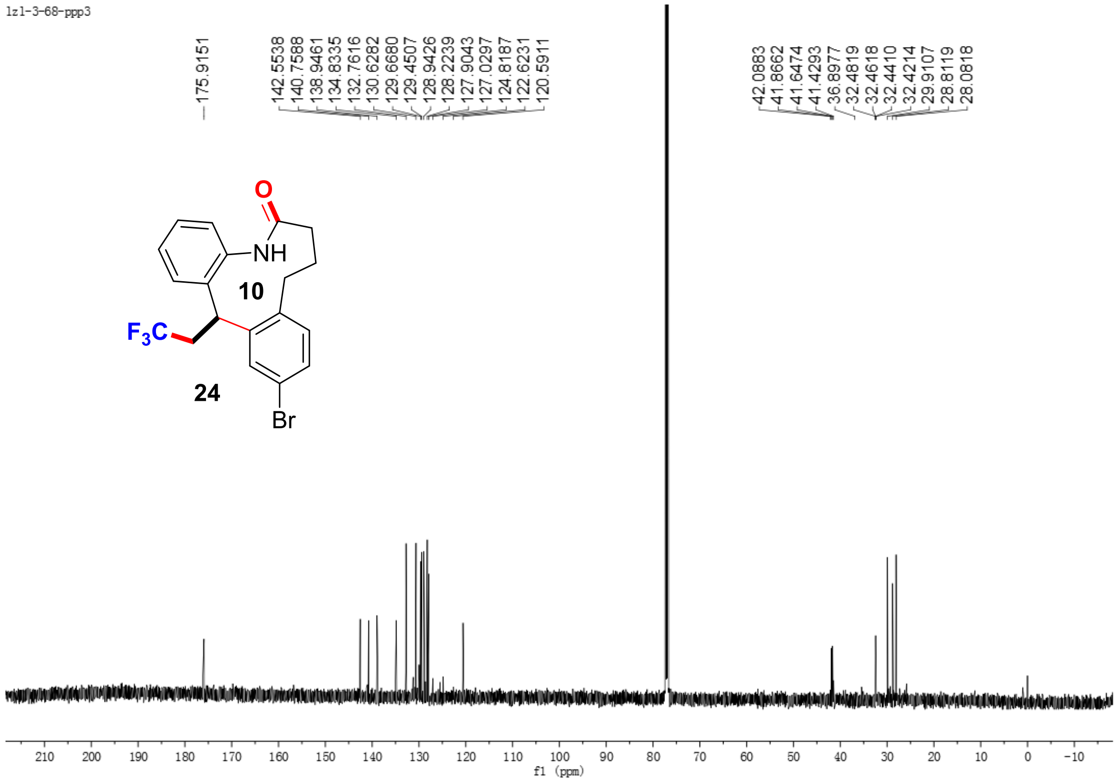

Supplementary Figure 195. ^{13}C NMR of **24**

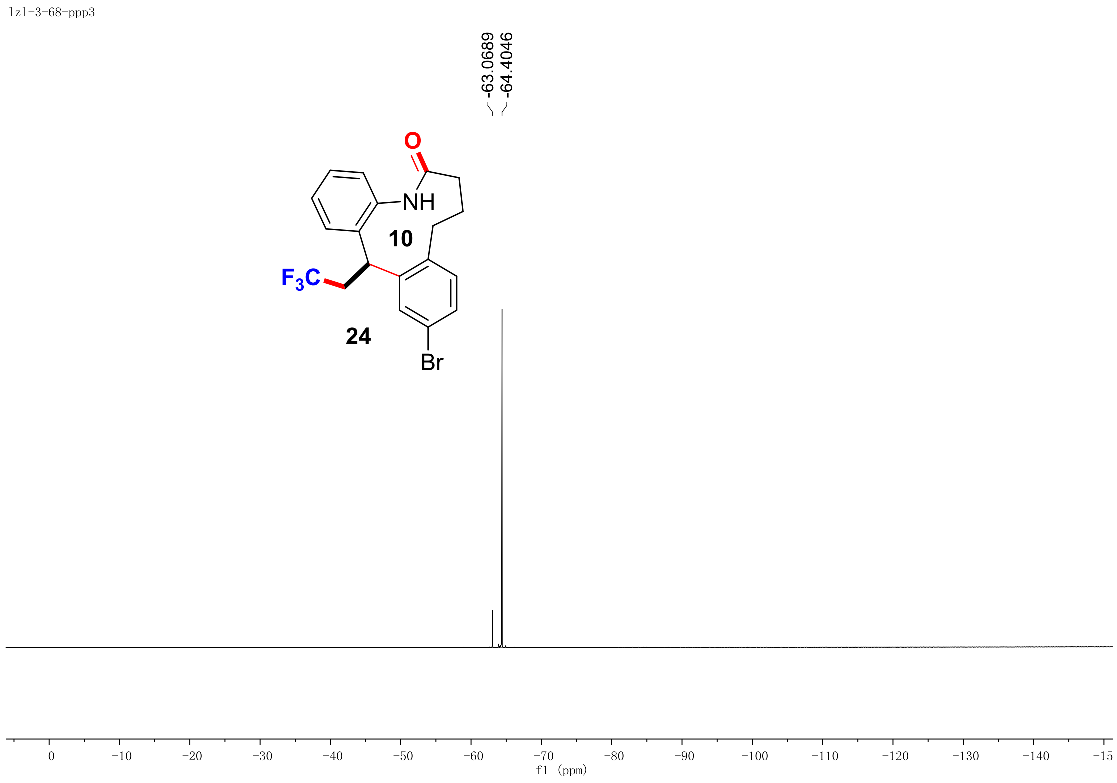

Supplementary Figure 196. ^{19}F NMR of **24**

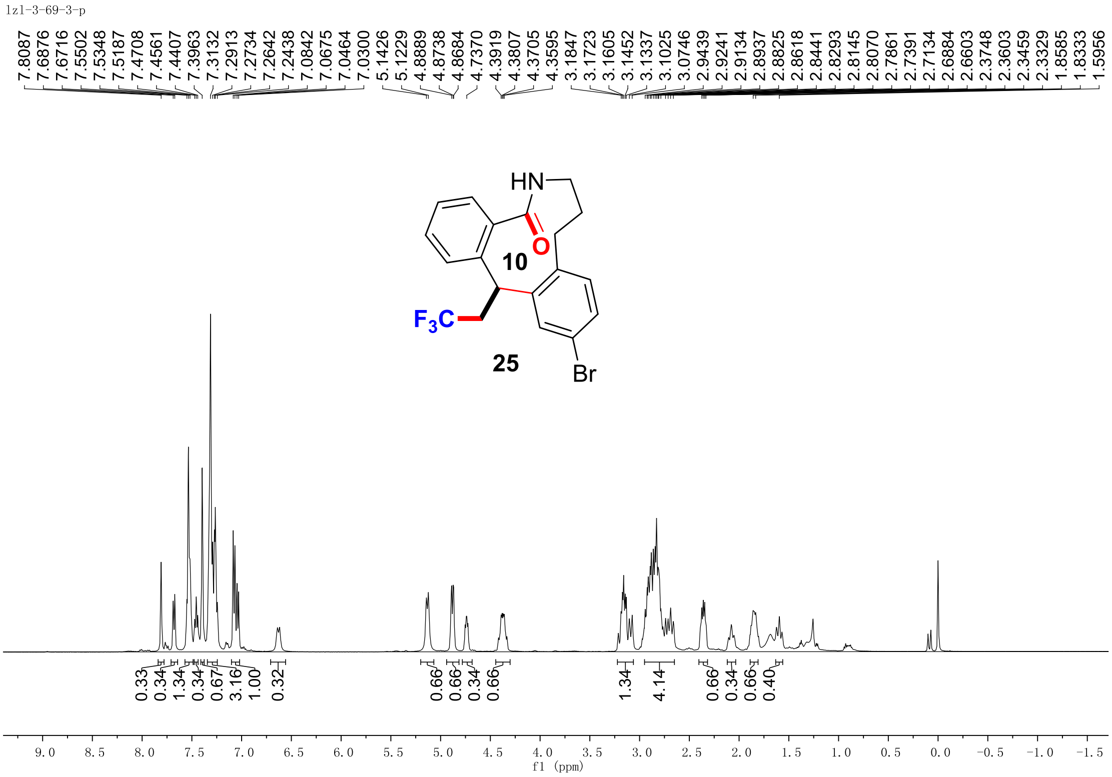

Supplementary Figure 197. ^{1}H NMR of **25**

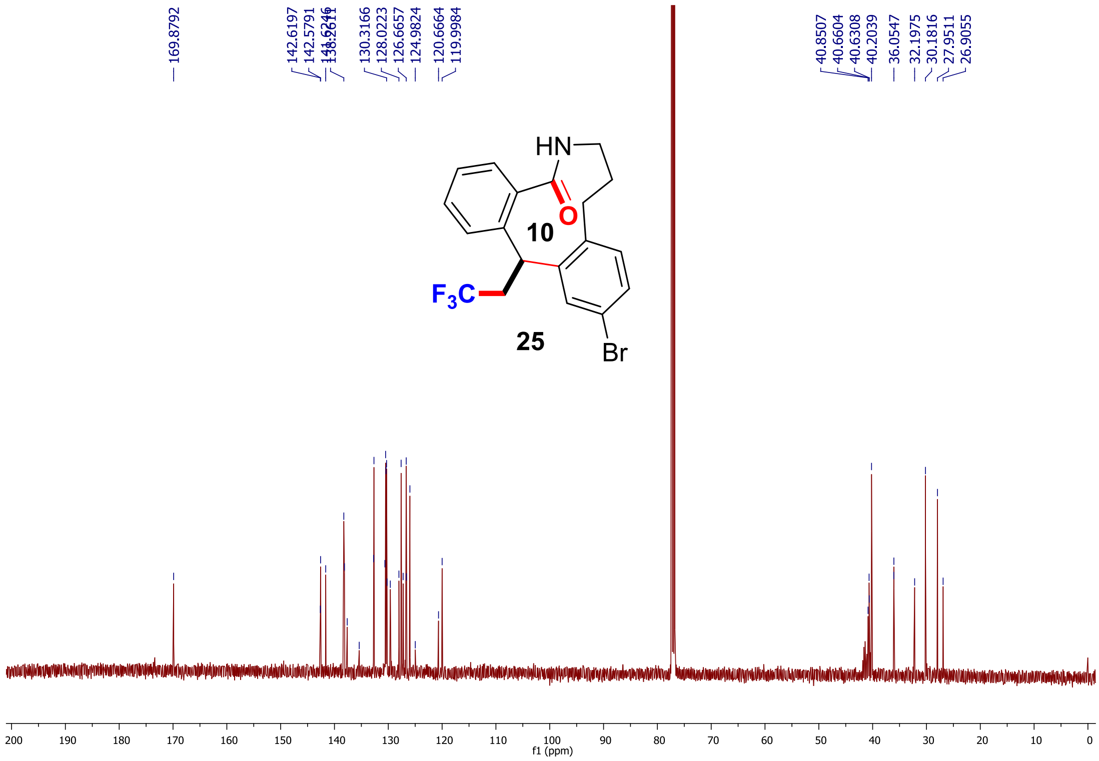

Supplementary Figure 198. ^{13}C NMR of **25**

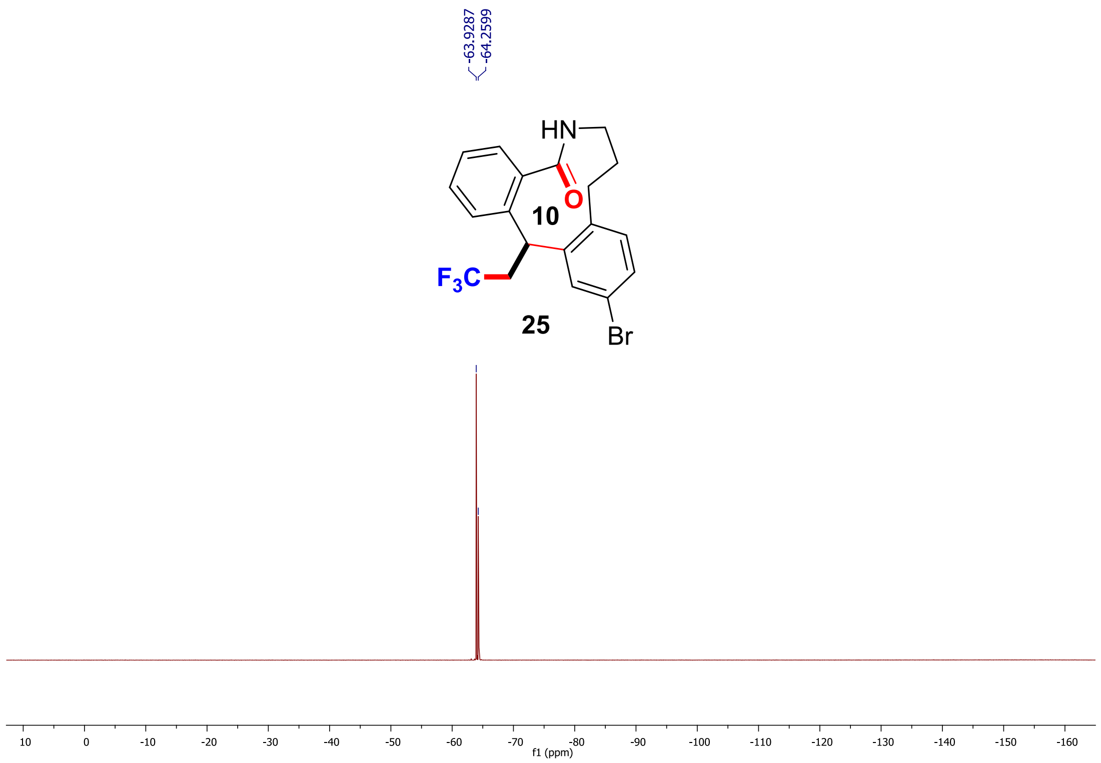

Supplementary Figure 199. ^{19}F NMR of **25**. For this compound, only the apparent aliphatic H and C of the major rotamer were characterized in 2D NMR.

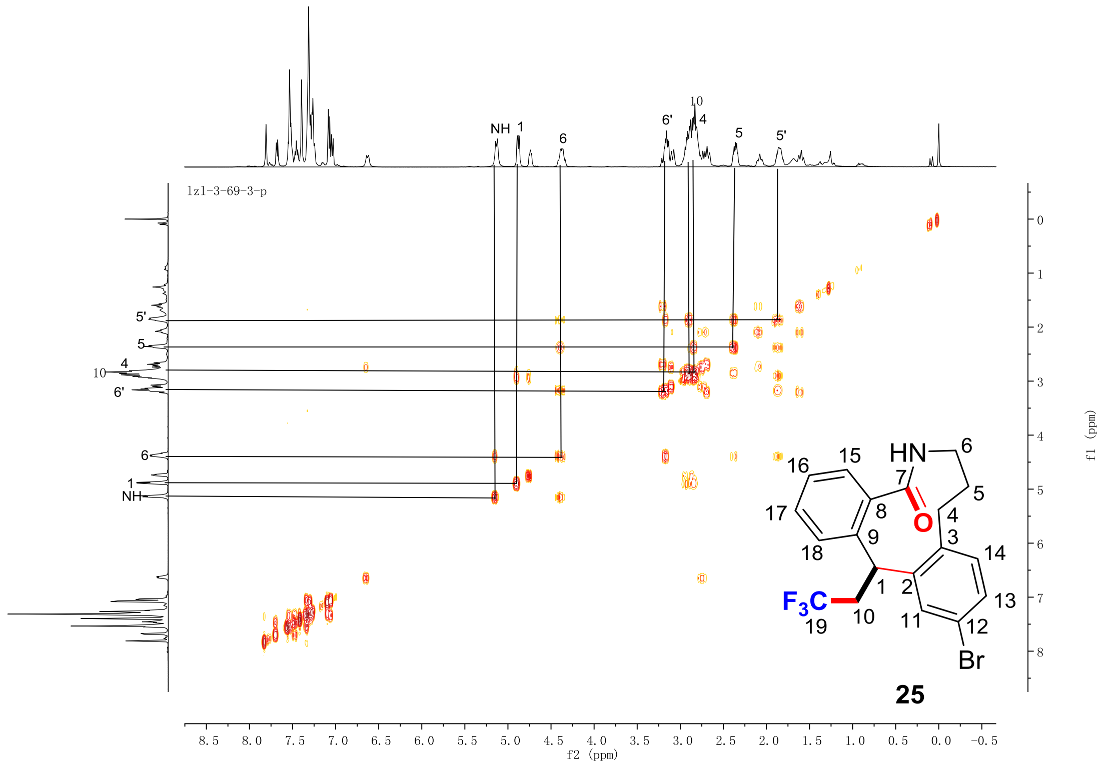

Supplementary Figure 200. COSY of **25**

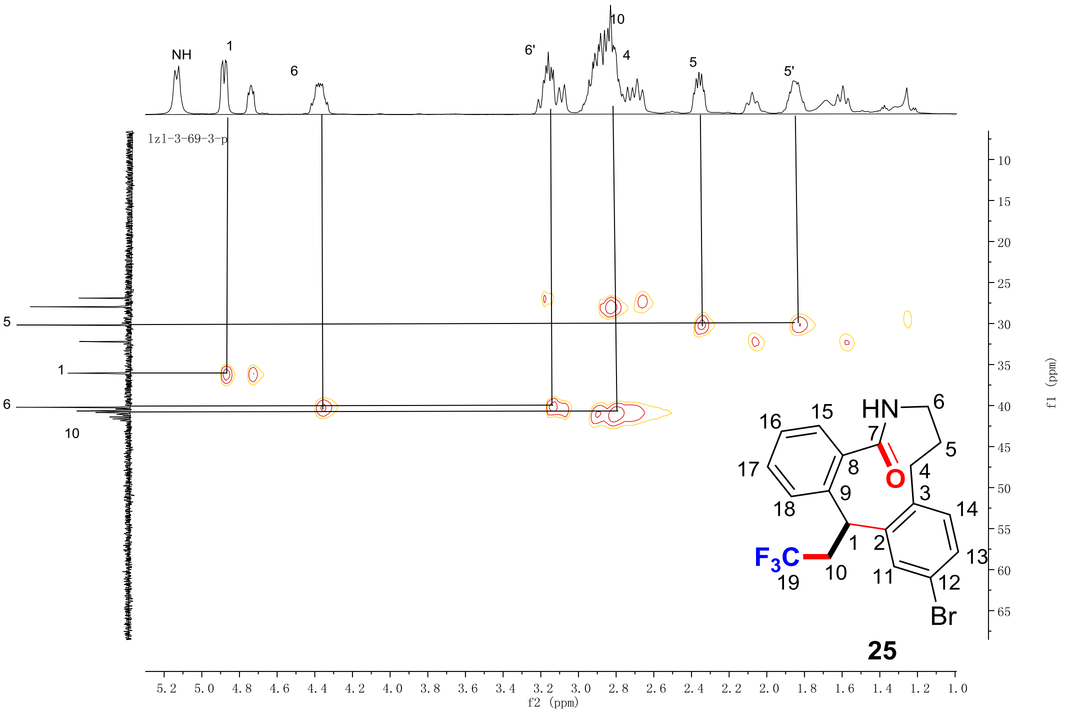

Supplementary Figure 201. HSQC of **25**

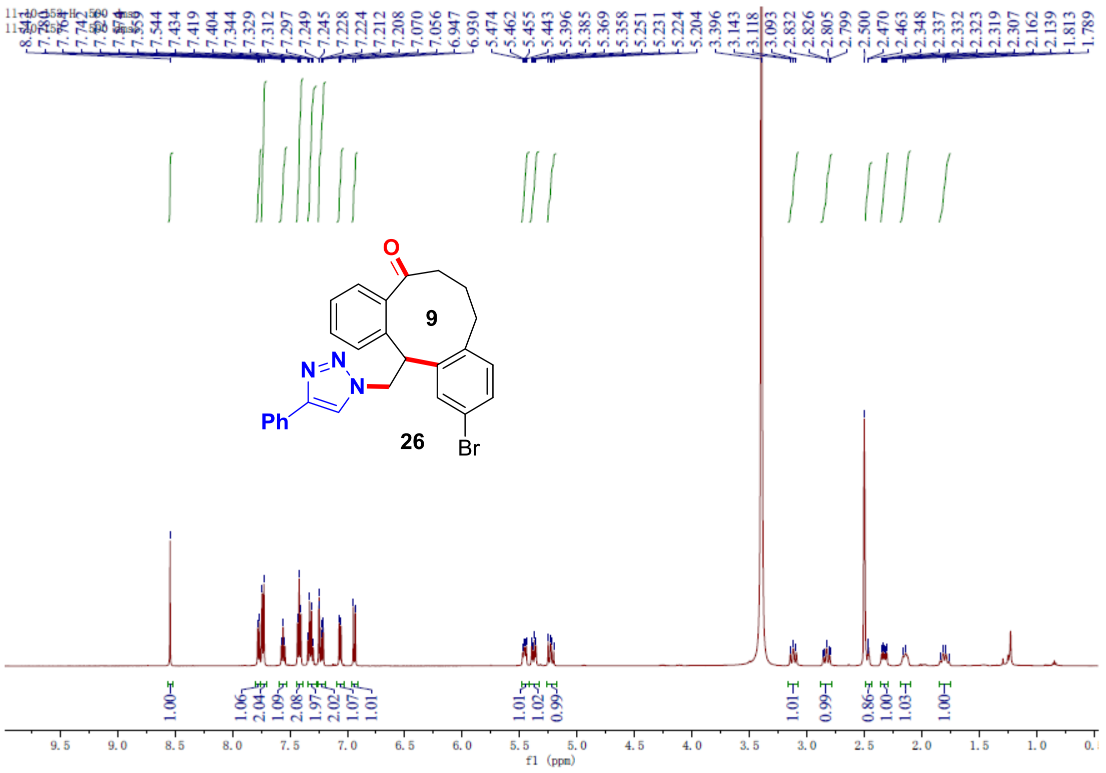

Supplementary Figure 202. ^{1}H NMR of **26**

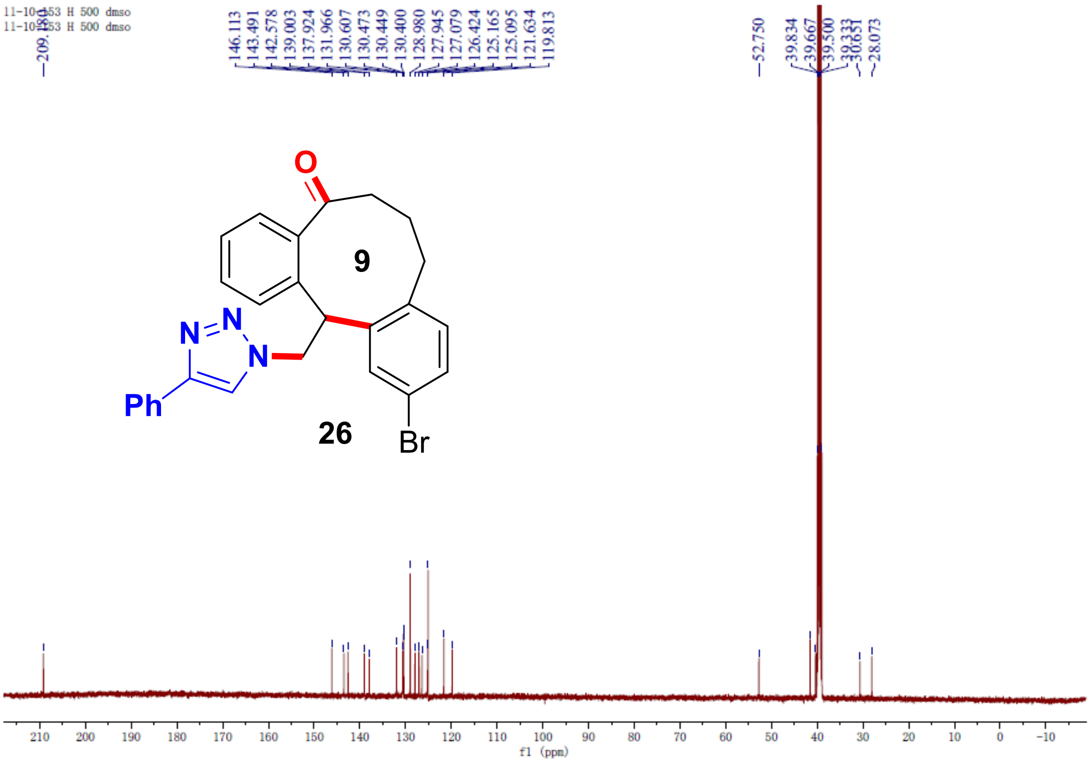

Supplementary Figure 203. ^{13}C NMR of **26**

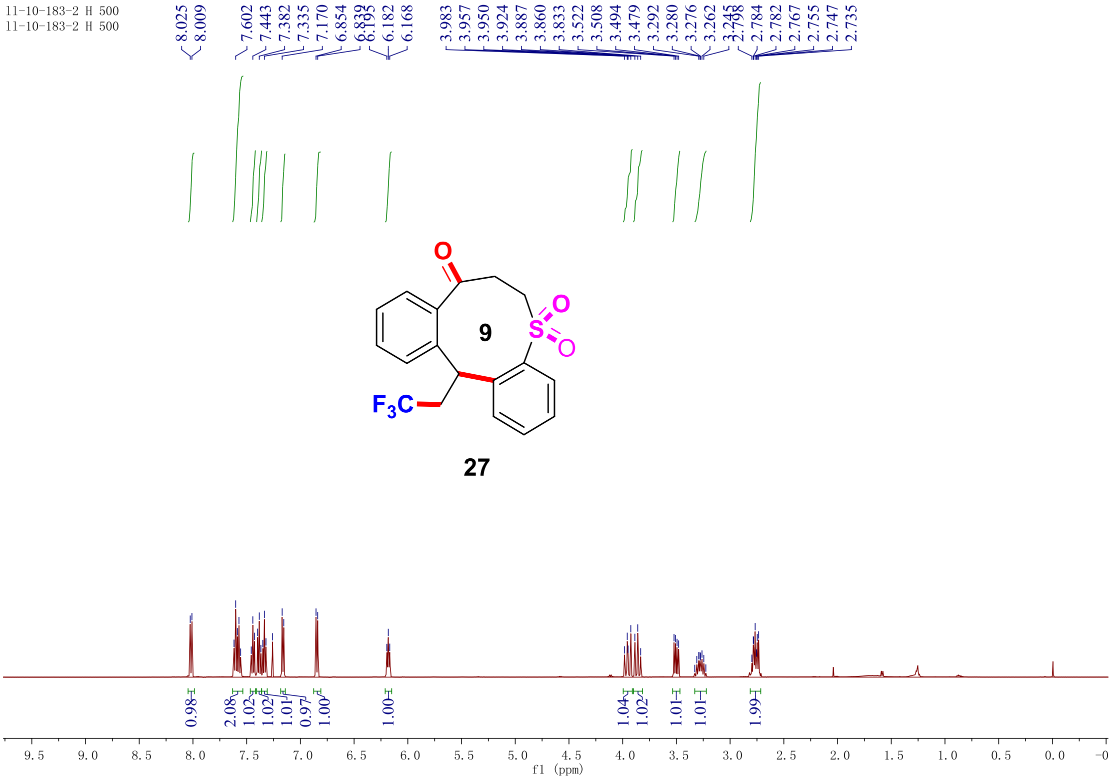

Supplementary Figure 204. ^{1}H NMR of **27**

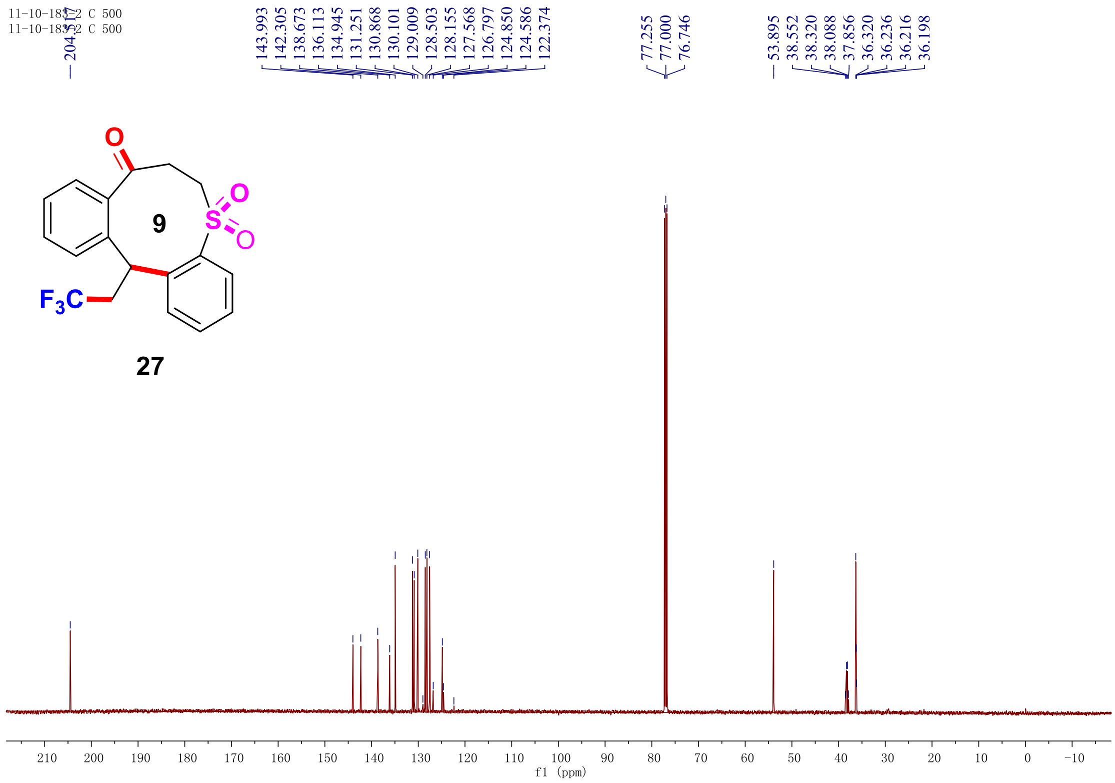

Supplementary Figure 205. ^{13}C NMR of **27**

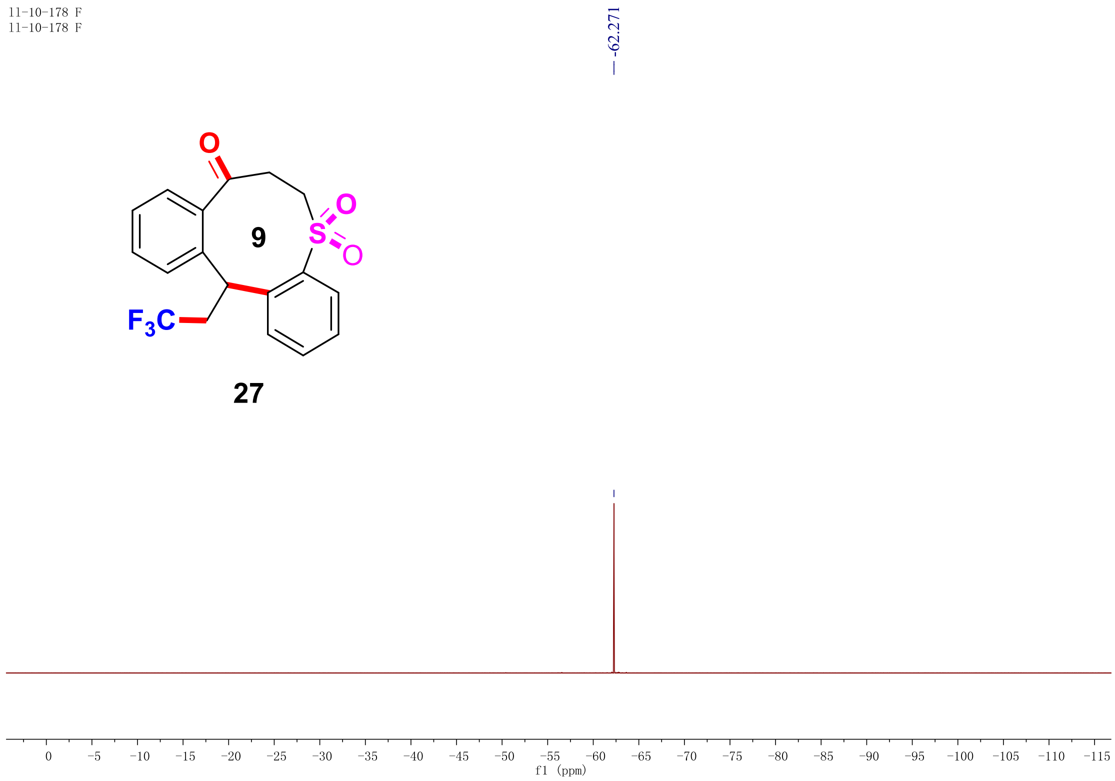

Supplementary Figure 206. ^{19}F NMR of **27**

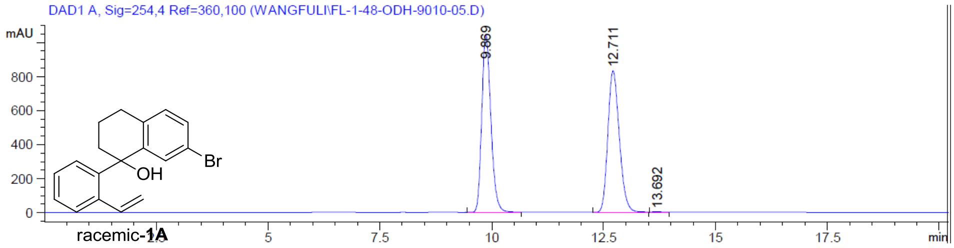

Signal 1: DAD1 A, Sig=254,4 Ref=360,100

Peak #	RetTime [min]	Type	Width [min]	Area [mAU*s]	Height [mAU]	Area %
1	9.869	BB	0.2196	1.48573e4	1046.79663	50.0150
2	12.711	BB	0.2760	1.48292e4	831.85687	49.9207

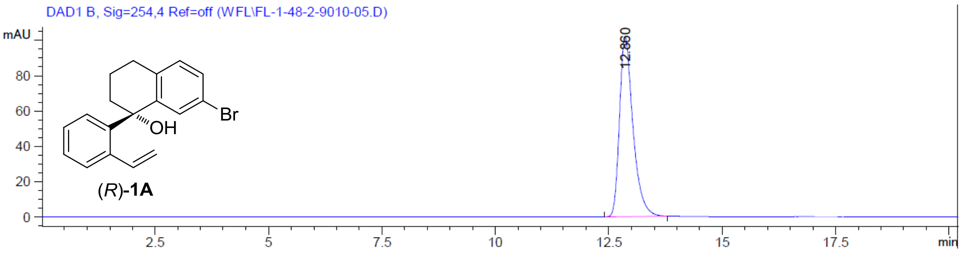

Signal 2: DAD1 B, Sig=254,4 Ref=off

Peak #	RetTime [min]	Type	Width [min]	Area [mAU*s]	Height [mAU]	Area %
1	12.860	BB	0.3102	2101.15942	102.04185	100.0000

Totals : 2101.15942 102.04185

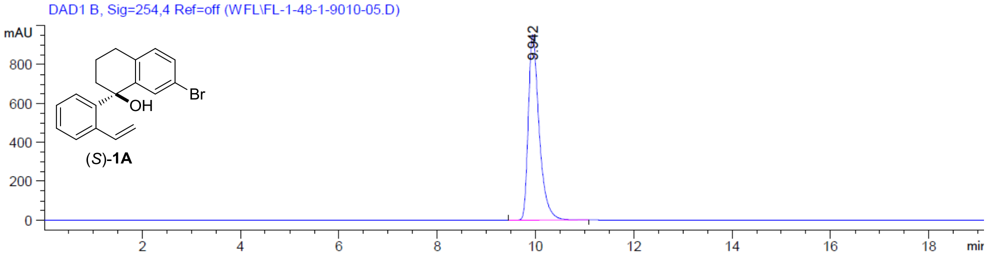

Signal 2: DAD1 B, Sig=254,4 Ref=off

Peak #	RetTime [min]	Type	Width [min]	Area [mAU*s]	Height [mAU]	Area %
1	9.942	BB	0.2308	1.46266e4	954.16766	100.0000

Totals : 1.46266e4 954.16766

Supplementary Figures 207. HPLC spectra for racemic and chiral **1A**

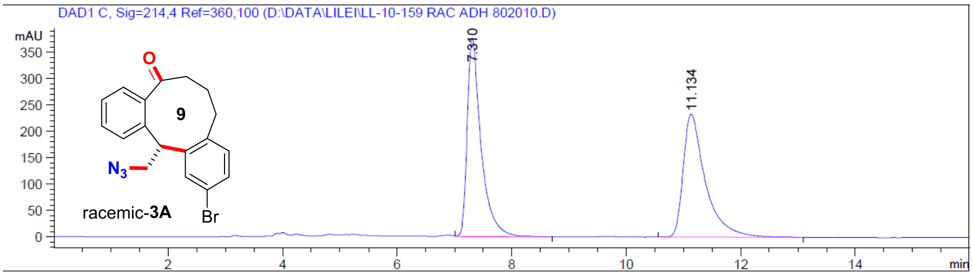

Signal 2: DAD1 C, Sig=214,4 Ref=360,100

Peak #	RetTime [min]	Type	Width [min]	Area [mAU*s]	Height [mAU]	Area %
1	7.310	VB	0.2459	6326.32178	376.74820	50.0803
2	11.134	BB	0.3968	6306.03027	233.73965	49.9197

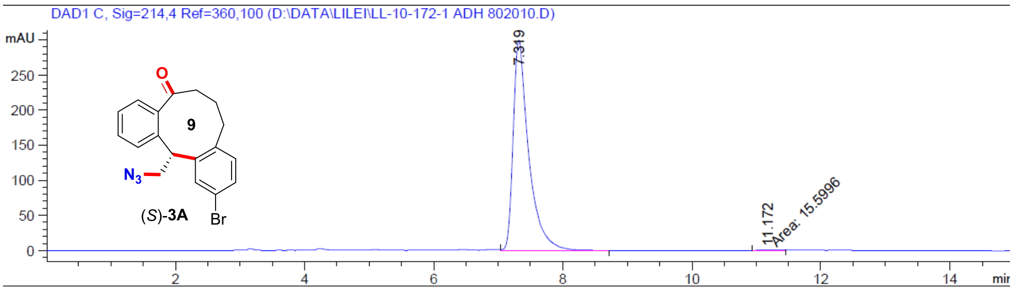

Signal 2: DAD1 C, Sig=214,4 Ref=360,100

Peak #	RetTime [min]	Type	Width [min]	Area [mAU*s]	Height [mAU]	Area %
1	7.319	VB	0.2457	5023.69629	299.50333	99.6904
2	11.172	MM	0.3350	15.59958	7.76151e-1	0.3096
Totals :				5039.29587	300.27948	

Supplementary Figures 208. HPLC spectra for racemic and chiral **3A**

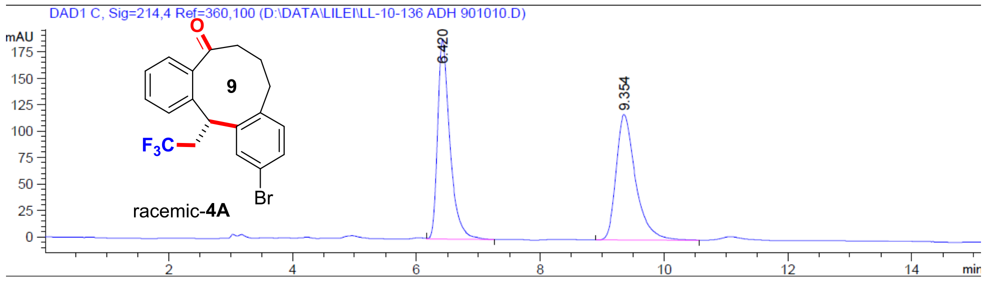

Signal 2: DAD1 C, Sig=214,4 Ref=360,100

Peak #	RetTime [min]	Type	Width [min]	Area [mAU*s]	Height [mAU]	Area %
1	6.420	VB	0.2054	2617.17480	189.19638	50.2215
2	9.354	BB	0.3271	2594.09180	118.63507	49.7785

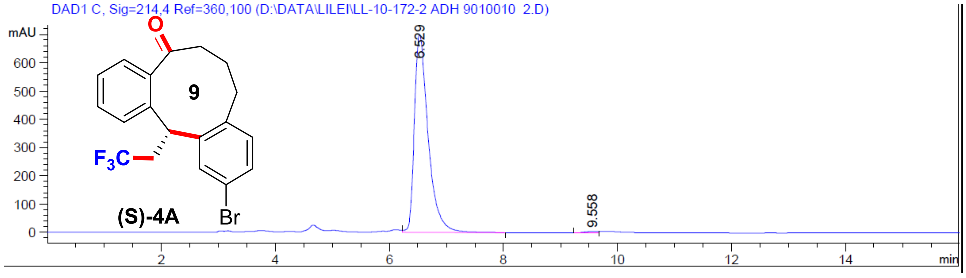

Signal 2: DAD1 C, Sig=214,4 Ref=360,100

Peak #	RetTime [min]	Type	Width [min]	Area [mAU*s]	Height [mAU]	Area %
1	6.529	VB	0.2523	1.17755e4	699.69702	99.4728
2	9.558	BV	0.2188	62.41514	4.26238	0.5272

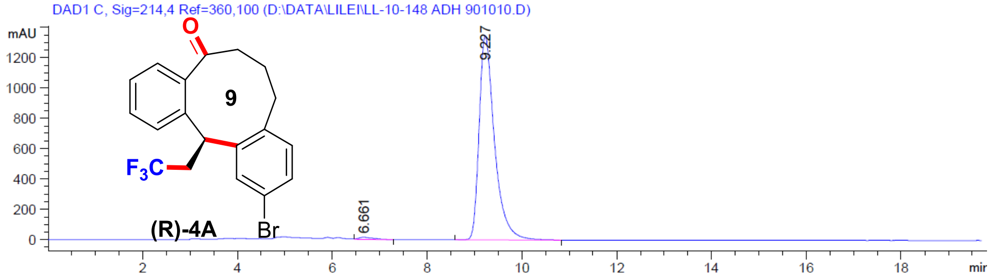

Signal 2: DAD1 C, Sig=214,4 Ref=360,100

Peak #	RetTime [min]	Type	Width [min]	Area [mAU*s]	Height [mAU]	Area %
1	6.661	VB	0.2936	283.00357	13.66404	0.9261
2	9.227	BB	0.3365	3.02768e4	1345.55933	99.0739

Supplementary Figures 209. HPLC spectra for racemic and chiral **4A**

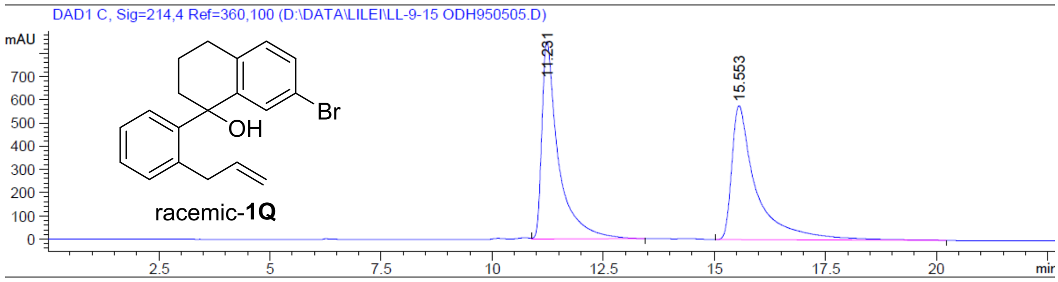

Signal 2: DAD1 C, Sig=214,4 Ref=360,100

Peak #	RetTime [min]	Type	Width [min]	Area [mAU*s]	Height [mAU]	Area %
1	11.231	VB	0.3679	2.20316e4	851.50696	49.6722
2	15.553	BB	0.5425	2.23223e4	578.04309	50.3278

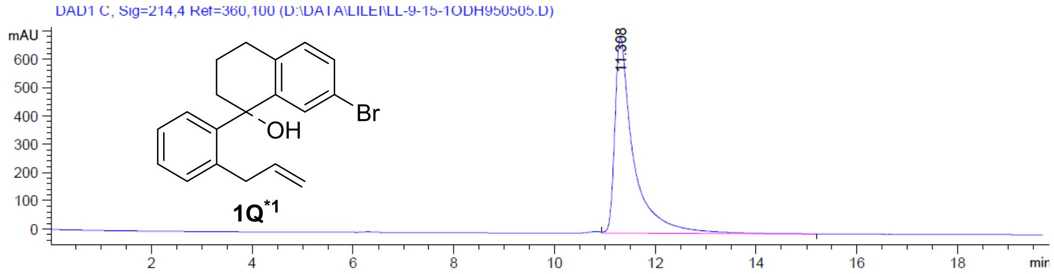

Signal 2: DAD1 C, Sig=214,4 Ref=360,100

Peak #	RetTime [min]	Type	Width [min]	Area [mAU*s]	Height [mAU]	Area %
1	11.308	VB	0.3645	1.79848e4	693.56543	100.0000

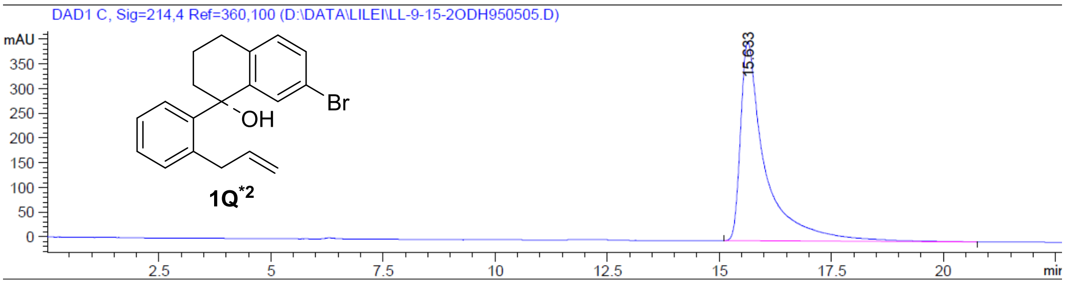

Signal 2: DAD1 C, Sig=214,4 Ref=360,100

Peak #	RetTime [min]	Type	Width [min]	Area [mAU*s]	Height [mAU]	Area %
1	15.633	BB	0.5356	1.54083e4	403.38303	100.0000

Supplementary Figures 210. HPLC spectra for racemic and chiral **1Q**

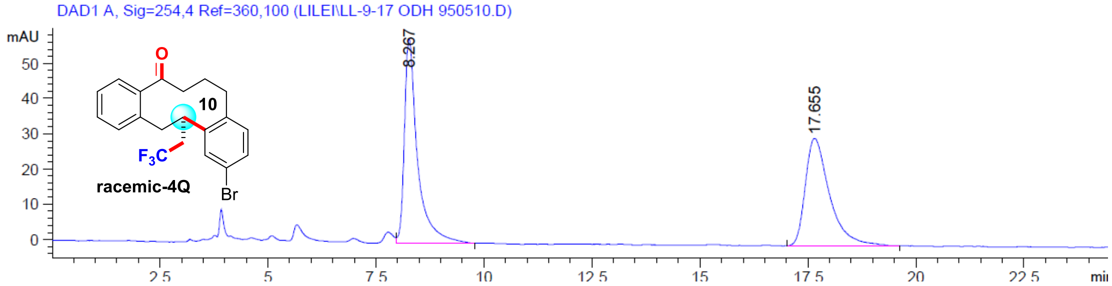

Signal 1: DAD1 A, Sig=254,4 Ref=360,100

Peak #	RetTime [min]	Type	Width [min]	Area [mAU*s]	Height [mAU]	Area %
1	8.267	VB	0.3044	1226.12097	58.06358	50.8140
2	17.655	BB	0.5804	1186.83948	30.47474	49.1860

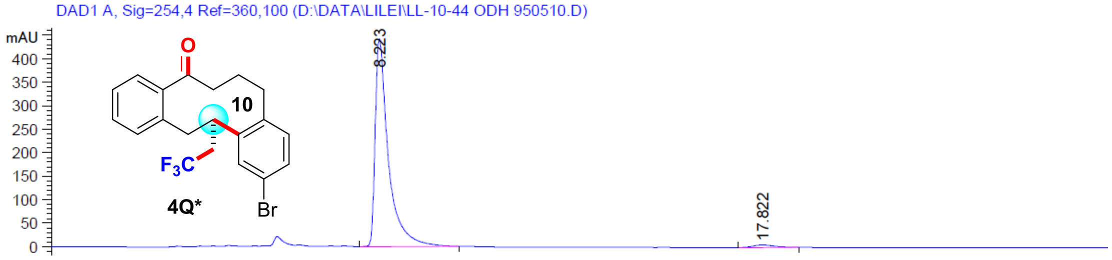

Signal 1: DAD1 A, Sig=254,4 Ref=360,100

Peak #	RetTime [min]	Type	Width [min]	Area [mAU*s]	Height [mAU]	Area %
1	8.223	BB	0.3330	1.01881e4	441.88507	97.9809
2	17.822	BB	0.5223	209.94885	5.69251	2.0191

Supplementary Figures 211. HPLC spectra for racemic and chiral **4Q**

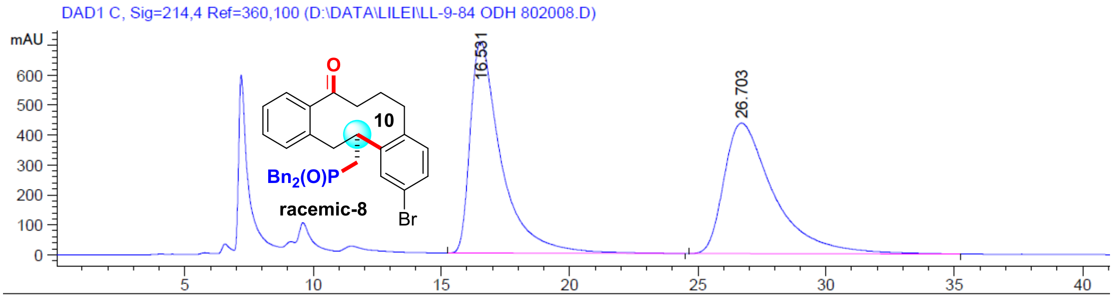

Signal 2: DAD1 C, Sig=214,4 Ref=360,100

Peak #	RetTime [min]	Type	Width [min]	Area [mAU*s]	Height [mAU]	Area %
1	16.531	BB	1.2178	5.94506e4	706.46680	50.2078
2	26.703	BB	1.9160	5.89585e4	436.44556	49.7922

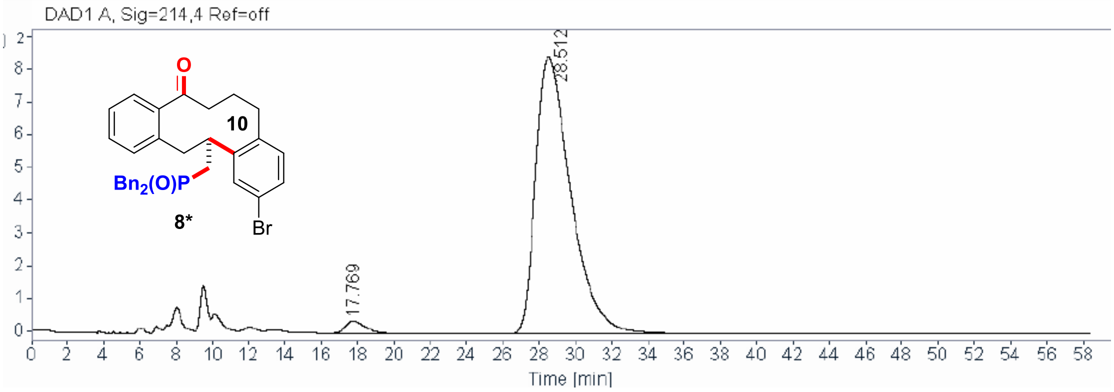

Signal:	DAD1 A, Sig=214,4 Ref=off			
RT [min]	Width [min]	Area	Height	Area%
17.769	1.1597	2943.6008	37.9161	2.5147
28.512	2.0183	114111.8125	848.8774	97.4853
	Sum	117055.4133		

Supplementary Figures 212. HPLC spectra for racemic and chiral **8**

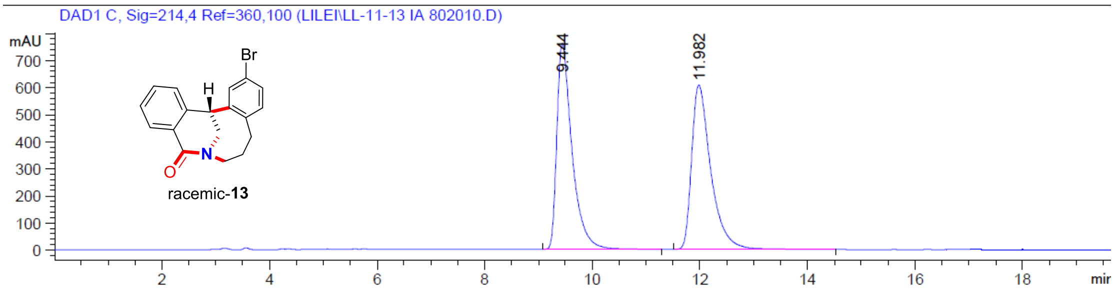

Signal 3: DAD1 C, Sig=214,4 Ref=360,100

Peak #	RetTime [min]	Type	Width [min]	Area [mAU*s]	Height [mAU]	Area %
1	9.444	BB	0.2906	1.49217e4	761.42749	49.7952
2	11.982	BB	0.3731	1.50444e4	607.11084	50.2048

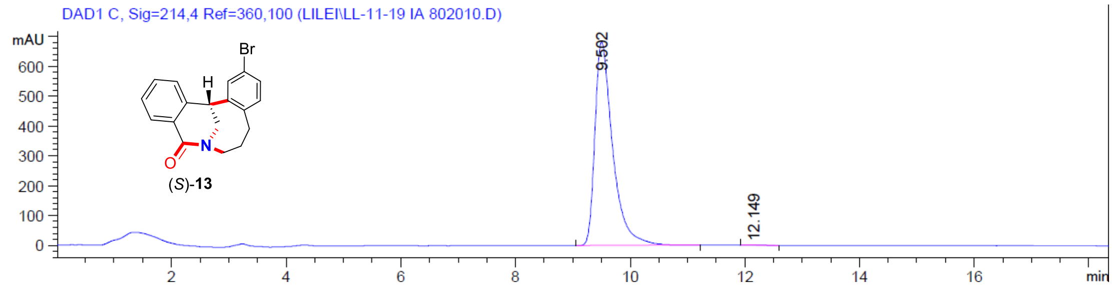

Signal 3: DAD1 C, Sig=214,4 Ref=360,100

Peak #	RetTime [min]	Type	Width [min]	Area [mAU*s]	Height [mAU]	Area %
1	9.502	BB	0.3214	1.47103e4	682.58014	99.8301
2	12.149	BB	0.2553	25.03953	1.23091	0.1699

Supplementary Figures 213. HPLC spectra for racemic and chiral **13**

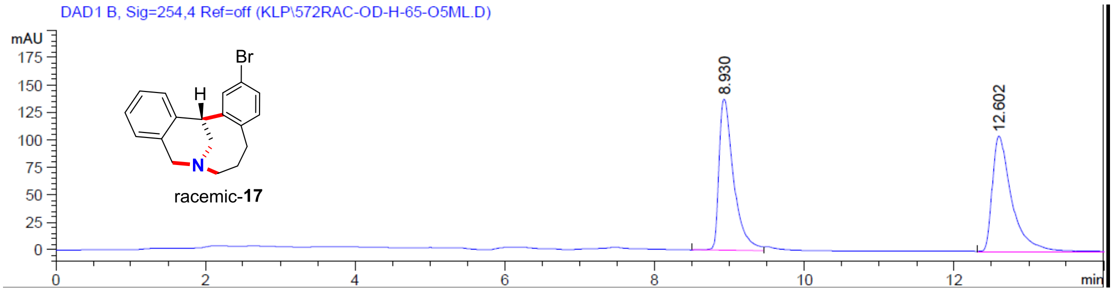

Signal 2: DAD1 B, Sig=254,4 Ref=off

Peak #	RetTime [min]	Type	Width [min]	Area [mAU*s]	Height [mAU]	Area %
1	8.930	BV	0.2024	1864.00842	137.25192	49.7245
2	12.602	BB	0.2660	1884.65991	104.69942	50.2755

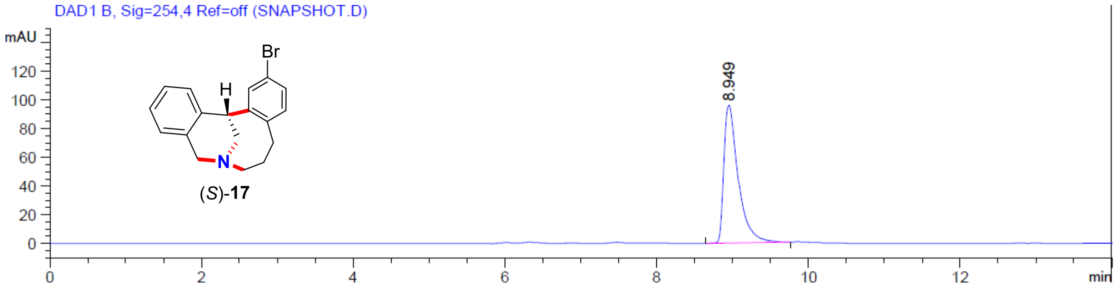

Signal 2: DAD1 B, Sig=254,4 Ref=off

Peak #	RetTime [min]	Type	Width [min]	Area [mAU*s]	Height [mAU]	Area %
1	8.949	BB	0.1947	1255.02271	95.86852	100.0000

Supplementary Figures 214. HPLC spectra for racemic and chiral **17**

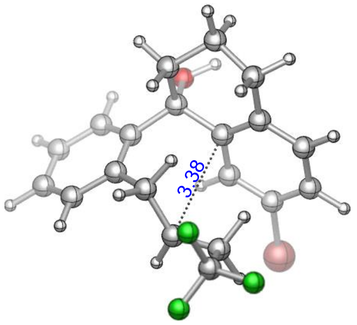

$1Q_1$

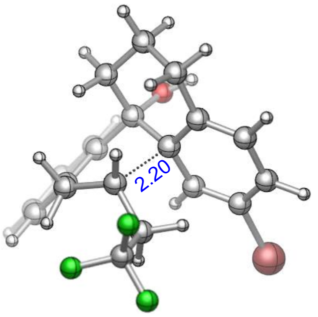

TS_{1Q1}

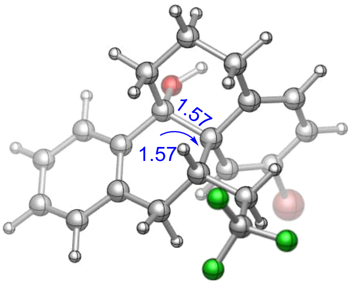

$2Q_1$

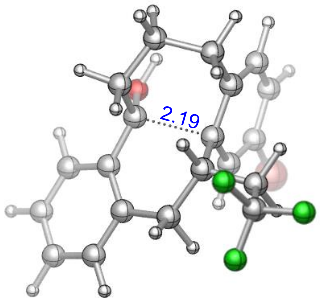

TS_{2Q1}

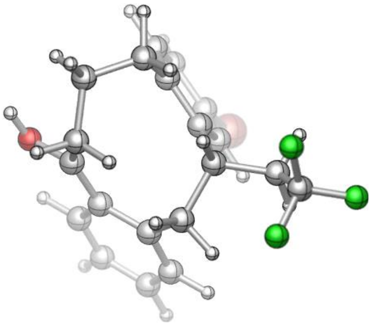

$3Q_1$

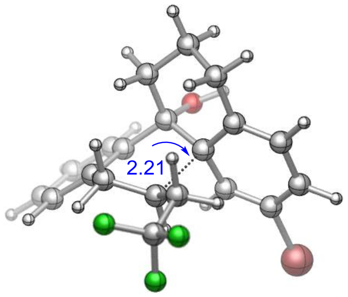

$TS_{1Q1'}$

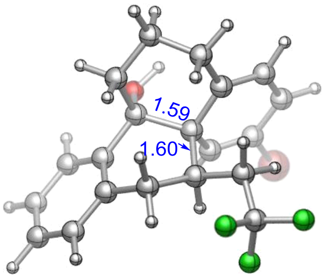

$2Q_1'$

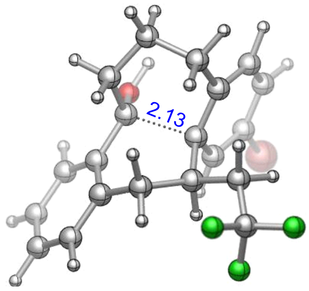

$TS_{2Q1'}$

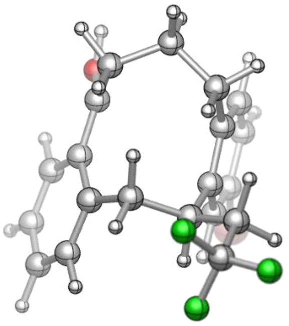

$3Q_1'$

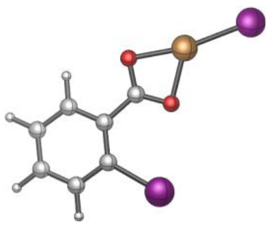

Cu^{II}-Species

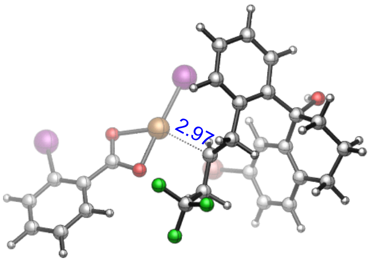

TS-SD

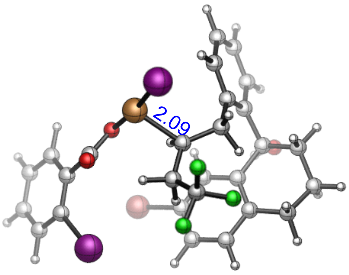

INT

Supplementary Figures 215. The key optimized structures for reactions (M11/6-31+G**/Aug-cc-PVTZ /Sdd in 1,4-dioxane)

Supplementary Table.

Supplementary Table 1. Screening of reaction conditions for azidation reaction of substrate **1A**.[a]

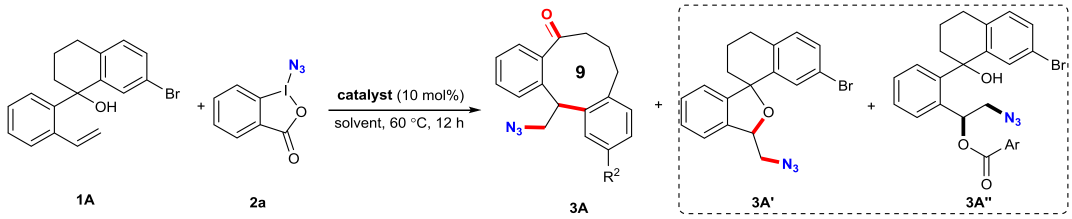

Entry	Catalyst	Solvent	Yield (%)[b]
1	CuI	EtOAc	47
2	$Cu(acac)_2$	EtOAc	37
3	CuCN	EtOAc	66
4	$Cu(CH_3CN)_4BF_4$	EtOAc	32
5	CuBr	EtOAc	38
6	CuCl	EtOAc	53
7	CuOAc	EtOAc	44
8	CuCN	DCE	65
9	CuCN	CH_3CN	38
10	CuCN	MeOH	27
11	CuCN	DMF	0
**12**[c]	**CuCN**	**EtOAc**	**72**
13[d]	CuCN	EtOAc	71
14[e]	CuCN	EtOAc	63

[a] Reaction conditions: **1A** (0.3 mmol), **2a** (0.5 mmol), catalyst (10 mol%), solvent (3.0 mL). [b] Determined by ^{1}H NMR spectroscopy with mesitylene as an internal standard. [c] **2a** (1.2 equiv). [d] **2a** (1.5 equiv). [e] **2a** (2.0 equiv).

Supplementary Table 2. Screening of reaction conditions for trifluoromethylation reaction of substrate **1A**.[a]

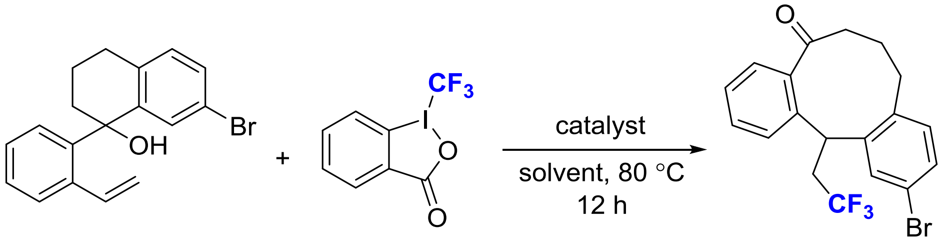

Entry	Catalyst	Solvent	Yield (%)[b]
1	CuI	EtOAc	47
2	CuCl	EtOAc	30
3	CuBr	EtOAc	45
4	TcCu	EtOAc	44
5	$Cu(CH_3CN)_4BF_4$	EtOAc	19
6	$Cu(CH_3CN)_4PF_6$	EtOAc	10
7	CuCN	EtOAc	50
8	DMAP	EtOAc	55
9	PPh_3	EtOAc	0
10[c]	CuCN	EtOAc	10
**11**	**CuCN**	**EtOAc**	**65 (64)**[d]
12	CuCN	DCE	55
13	CuCN	CH_3CN	---

[a] Reaction conditions: **1A** (0.2 mmol), **2b** (0.4 mmol), catalyst (10 mol%), solvent (2.0 mL). [b] Determined by ^{19}F NMR spectroscopy with $PhCF_3$ as an internal standard. [c] 60 °C. [d] Isolated yield in parenthesis.

Supplementary Table 3. Screening of reaction conditions for trifluoromethylation reaction of substrate **1N**.[a]

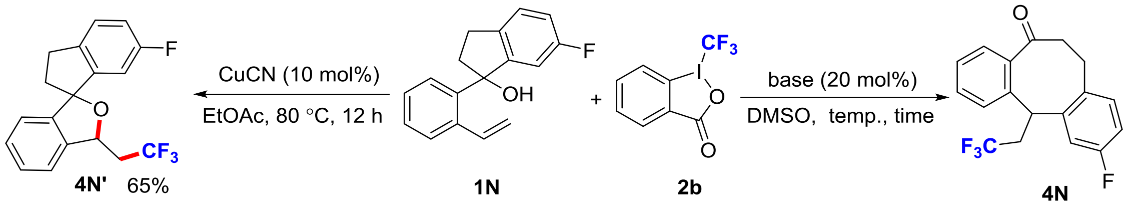

Entry	Base	Solvent	T (°C)	Time (h)	Yield (%)[b]
1	TBD	DMSO	50	24	0
2	TBD	DMSO	80	24	20
3	TBD	DMSO	100	10	22
4	DMAP	DMSO	100	10	14
5	DBU	DMSO	100	10	24
6	DABCO	DMSO	100	10	29
7	1,10-phen	DMSO	100	10	0
8	TBD	DMF	100	10	0
9	TBD	DCE	100	10	30
10	TBD	MeOH	100	10	20
11[c]	TBD	EtOAc/DMSO	100	10	30
**12**[c]	**TBD**	**DCE/DMSO**	**100**	**10**	**44**[d]
13[c]	TBD	MeOH/DMSO	100	10	11

[a] Reaction conditions: **1N** (0.2 mmol), Togni's reagent **2b** (0.4 mmol), base (20 mol%), solvent (2.0 mL). [b] Determined by ^{19}F NMR spectroscopy with $PhCF_3$ as an internal standard. [c] V(solvent: EtOAc, DCE, MeOH) : V(DMSO) = 10:1. [d] Isolated yield. DCE = 1,2-dichloroethane.

Supplementary Table 4. Screening of reaction conditions for trifluoromethylation reaction of substrate **1O**.[a]

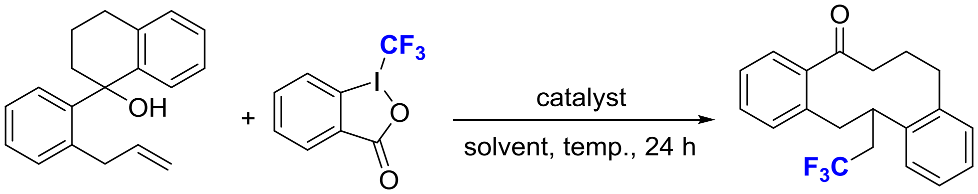

1O **2b** **4O**

Entry	Catalyst	Solvent (mL)	T (°C)	Yield (%)[b]
1	CuCN	EtOAc (2)	80	37
2	CuCN	Dioxane (2)	80	57
3	CuCN	CH_3CN (2)	80	30
4	CuCN	DGDE (2)	80	38
5	CuCN	DME (2)	80	41
6	CuCN	EtOAc (2)	60	40
7	CuCN	Dioxane (2)	60	62
**8**	**CuCN**	**Dioxane (4)**	**60**	**65**[c]
9[d]	CuCN	Dioxane (4)	60	55
10	CuI	Dioxane (4)	60	46
11	CuBr	Dioxane (4)	60	50
12	CuCl	Dioxane (4)	60	63
13	$Cu(CH_3CN)_4BF_4$	Dioxane (4)	60	22
14	$Cu(C_6H_6)_{0.5}OTf$	Dioxane (4)	60	17
15	Copper(I) hexafluoroacetylacetonate cyclooctadiene	Dioxane (4)	60	58

[a] Reaction conditions: **1O** (0.2 mmol), **2b** (0.4 mmol), catalyst (10 mol%). [b] Determined by ^{19}F NMR spectroscopy with $PhCF_3$ as an internal standard. [c] Isolated yield. [d] 12 h. DGDE = Diethylene glycol dimethyl ether, DME = 1,2-dimethoxyethane.

Supplementary Table 5. Screening of reaction conditions for phosphonylation reaction of **1Q**.[a]

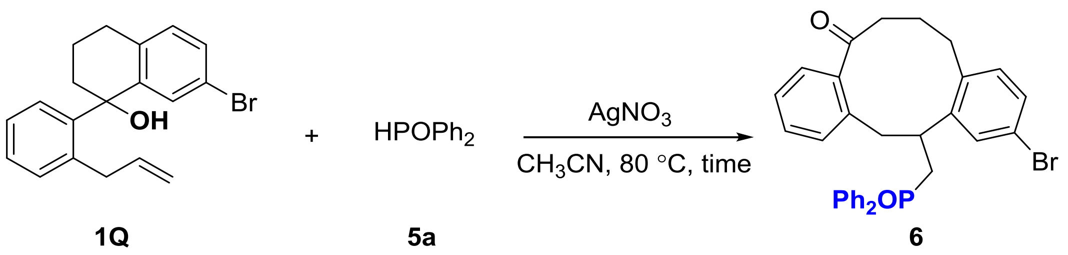

Entry	5a (equiv)	$AgNO_3$ (equiv)	CH_3CN (mL)	t (h)	Yield[b] (%)
1	2	0.3	2	24	40
**2**	**2**	**0.5**	**2**	**24**	**70**
3	2	1.0	2	24	69
4	3	0.5	2	24	56
5	3	0.5	2	12	61
6	2	0.5	4	24	55
7	2	0.6	2	24	63
8	2	0.8	2	24	65
9	3	1.0	2	24	58
10[c]	2	---	---	24	53
11[d]	2	0.5	2	24	0

[a] Reaction conditions: **1Q** (0.2 mmol), **5a**, $AgNO_3$, CH_3CN. [b] Determined by ^1H NMR spectroscopy with mesitylene as an internal standard. [c] AgOAc (3 equiv), DMF (2 mL), 100 °C. [d] $NaHCO_3$ (1.0 equiv) was added.

Supplementary Table 6. Screening of reaction conditions for Schmidt-Aubé reaction of **3A**.[a]

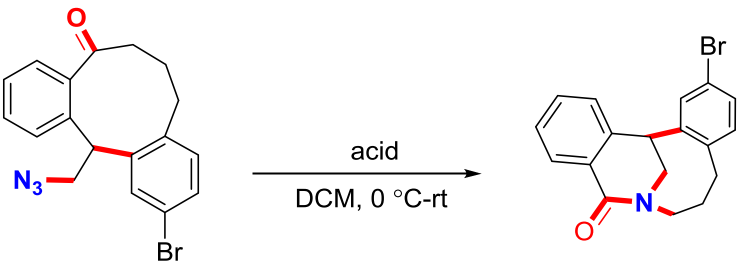

Entry	Acid	Yield (%)
1	$TiCl_4$ (1.2 equiv)	26
2	$BF_3{\cdot}OEt_2$ (1.2 equiv)	0
3	TfOH (1.2 equiv)	48
**4**	**TfOH (2.0 equiv)**	**85**

[a] Reaction conditions: **3A** (0.1 mmol), DCM (1 mL).

Supplementary Table 7. Screening of reaction conditions for one-pot reaction.[a]

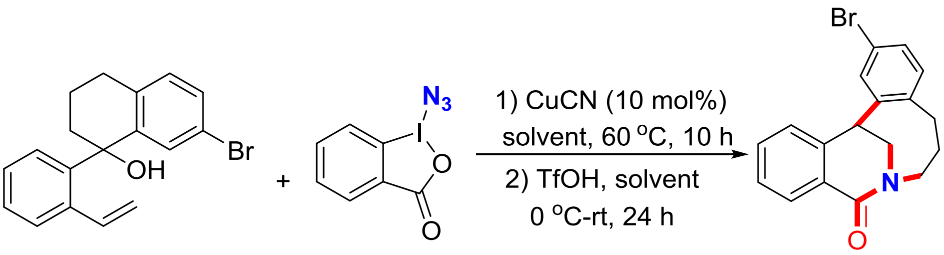

Entry	Solvent	Yield (%)
1[b]	1) EtOAc<br>2) EtOAc	18
2[c]	1) EtOAc<br>2) DCM	38
3[d]	1) EtOAc<br>2) DCM	62
4[e]	1) DCM<br>2) DCM	20

[a] Reaction conditions: **1A** (0.2 mmol), **2a** (0.24 mmol), TfOH (0.4 mmol), DCM (1 mL). [b] Solvent was not changed. [c] EtOAc was concentrated and DCM was added. [d] The reaction was washed with saturated $NaHCO_3$ and extracted with EtOAc, and the organic layer was concentrated and DCM was added. [e] Solvent was not changed.

Supplementary Table 8. Structural and physicochemical descriptors used in principal component analysis.

Parameter	Description	Method of Determination
MW	molecular weight	ChemDraw Analysis Window
N	number of nitrogens	ChemDraw Analysis Window
O	number of oxygenes	ChemDraw Analysis Window
HBD	number of hydrogen bond donors	http://www.molinspiration.com
HBA	number of hydrogen bond acceptors	http://www.molinspiration.com
RotB	number of rotatable bonds	http://www.molinspiration.com
tPSA	topological polar surface area	http://www.molinspiration.com
ALOGPs	calculated *n*-octanol/water partition coefficient	http://www.vcclab.org
ALOGpS	calculated aqueous solubility	http://www.vcclab.org
nStereo	number of stereocenters (R + S)	Microsoft Excel
R	number of *R* stereocenters	ChemDraw Show Stereochemistry
S	number of *S* stereocenters	ChemDraw Show Stereochemistry
nStMW	nStereo ÷ MW (stereochemical density)	Microsoft Excel
RSdelta	R – S	Microsoft Excel
Rings	number of rings	Manual inspection
RngAr	number of aromatic rings	Manual inspection
RngSys	number of ring systems	Manual inspection
RngLg	number of atoms in largest ring outline	Manual inspection
RRSys	Rings ÷ RngSys (ring complexity)	Microsoft Excel

Supplementary Table 9. Standard deviation and percent contribution for each principal component

	PC1	PC2	PC3	PC4	PC5	PC6	PC7	PC8	PC9	PC10
**Standard deviation**	2.878	1.710	1.570	1.199	1.058	0.941	0.768	0.633	0.590	0.378
**Proportion of Variance**	0.436	0.154	0.130	0.076	0.059	0.047	0.031	0.021	0.018	0.008
**Cumulative Proportion**	0.436	0.590	0.720	0.795	0.854	0.901	0.932	0.953	0.971	0.979

Supplementary Table 10. Cytotoxicity study of **3A**, **3I**, **13** and **15**.

Compound	IC_{50} (μM)	
	293T	H1299
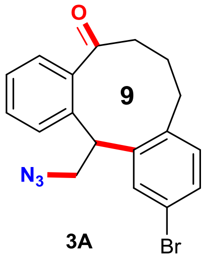 **3A**	60.9	23.95
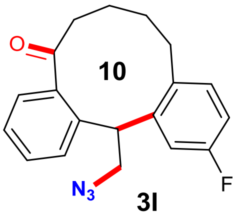 **3I**	76.6	10.8
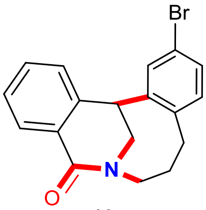 **13**	18.5	136.7
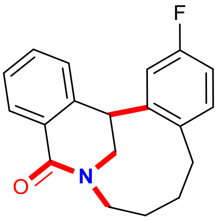 **15**	10.9	19.5

Supplementary Notes

General procedure

General procedure for synthesis of substrates 1A-1I, 1M-1U, 1W, 1X.

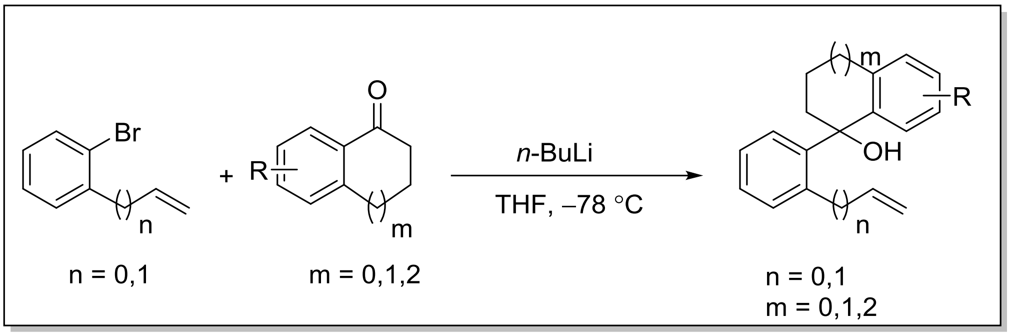

To a solution of 1-bromo-2-vinylbenzene (2.0 g, 11 mmol, 1.1 equiv) in anhydrous THF (10 mL) was added *n*-BuLi (4.6 mL, 2.4 M in *n*-hexane, 11 mmol, 1.1 equiv) over 10 min at – 78 °C. The reaction mixture was stirred for 1-2 h at that temperature and then to this solution was added a solution of 7-bromo-3, 4-dihydronaphthalen-1(2H)-one (2.2 g, 10 mmol, 1.0 equiv) in THF (10 mL) over 10 min. The reaction mixture was stirred for an additional 2 h, and quenched with saturated $NaHCO_3$ solution (10 mL). EtOAc was used to extract the product from the aqueous layer (3 × 30 mL). The combined organic layer was dried over anhydrous Na_2SO_4, filtered and concentrated to afford the crude product, which was purified by flash column chromatography to afford the desired product **1A** (2.1 g, 65%).

The synthesis of other substrates is similar to that of **1A**.

7-bromo-1-(2-vinylphenyl)-1,2,3,4-tetrahydronaphthalen-1-ol

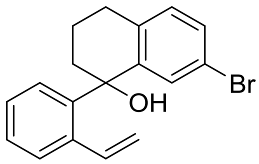

1A

^{1}H NMR (400 MHz, $CDCl_3$) δ 7.85 (dd, *J* = 8.0, 1.2 Hz, 1H), 7.41 (dd, *J* = 7.6, 1.6 Hz, 1H), 7.35 (td, *J* = 7.6, 1.6 Hz, 1H), 7.33 - 7.27 (m, 2H), 7.09 - 7.00 (m, 2H), 6.31 (dd, *J* = 17.2, 10.8 Hz, 1H), 5.40 (dd, *J* = 17.2, 1.2 Hz, 1H), 4.99 (dd, *J* = 10.8, 1.2 Hz, 1H), 2.92 - 2.75 (m, 2H), 2.35 - 2.25 (m, 1H), 2.11 - 2.01 (m, 2H), 2.01 - 1.93 (m, 1H), 1.89 - 1.81 (m, 1H).
^{13}C NMR (100 MHz, $CDCl_3$) δ 144.19, 144.0, 136.20, 135.75, 135.35, 130.71, 130.50, 130.33, 127.73, 127.27, 127.20, 126.06, 119.94, 115.17, 74.23, 37.99, 29.10, 19.15.
HRMS (ESI) m/z calcd. for $C_{18}H_{16}Br$ $[M-OH]^+$ 311.0429, found 311.0426.

7-fluoro-1-(2-vinylphenyl)-1,2,3,4-tetrahydronaphthalen-1-ol

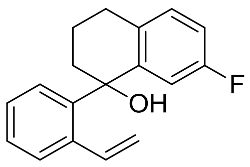

1B

^{1}H NMR (500 MHz, $CDCl_3$) δ 7.88 (d, *J* = 8.0 Hz, 1H), 7.41 (d, *J* = 7.5 Hz, 1H), 7.38 - 7.32 (m, 1H), 7.32 - 7.27 (m, 1H), 7.14 (dd, *J* = 8.5, 5.5 Hz, 1H), 6.90 (td, *J* = 8.5, 2.5 Hz, 1H), 6.63 (dd, *J* = 10.0, 2.5 Hz, 1H), 6.29 (dd, *J* = 17.0, 11.0 Hz, 1H), 5.40 (dd, *J* = 17.0, 1.5 Hz, 1H), 4.98 (dd, *J* = 11.0, 1.5 Hz, 1H), 2.95 - 2.87 (m, 1H), 2.87 - 2.79 (m, 1H), 2.30 (td, *J* = 13.5, 3.0 Hz, 1H), 2.13 - 2.03 (m, 2H), 2.03 - 1.96 (m, 1H), 1.91 - 1.82 (m, 1H).
^{13}C NMR (125 MHz, $CDCl_3$) δ 161.61 (d, *J* = 243.1 Hz), 144.51, 143.80 (d, *J* = 6.0 Hz), 136.27, 135.64, 132.61 (d, *J* = 3.0 Hz), 130.50 (d, *J* = 7.5 Hz), 127.90, 127.51, 127.41, 126.10, 115.44, 114.88 (d, *J* = 21.3 Hz), 114.31 (d, *J* = 21.1 Hz), 74.53, 38.06, 29.06, 19.62.
^{19}F NMR (376 MHz, $CDCl_3$) δ -115.46 (s).
HRMS (ESI) m/z calcd. for $C_{18}H_{16}F$ $[M\text{-}OH]^+$ 251.1231, found 251.1236.

1-(2-vinylphenyl)-1,2,3,4-tetrahydronaphthalen-1-ol

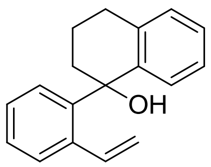

1C

^{1}H NMR (500 MHz, $CDCl_3$) δ 7.49 (d, *J* = 6.5 Hz, 1H), 7.34 - 7.29 (m, 3H), 7.28 - 7.08 (m, 5H), 5.51 (dd, *J* = 17.5, 3.5 Hz, 1H), 5.18 (dd, *J* = 11.0, 3.5 Hz, 1H), 3.02 - 2.91 (m, 1H), 2.89 - 2.72 (m, 2H), 2.35 (s, 1H), 2.16 - 2.06 (m, 1H), 2.05 - 1.96 (m, 1H), 1.72 - 1.62 (m, 1H).
^{13}C NMR (125 MHz, $CDCl_3$) δ 145.78, 143.90, 141.54, 138.25, 137.98, 130.88, 128.80, 127.80, 127.45, 127.40, 127.36, 126.95, 126.27, 115.17, 81.26, 40.12, 35.83, 27.12. 24.45
HRMS (ESI) m/z calcd. for $C_{18}H_{17}$ $[M\text{-}OH]^+$ 233.1325, found 233.1316.

6-methoxy-1-(2-vinylphenyl)-1,2,3,4-tetrahydronaphthalen-1-ol

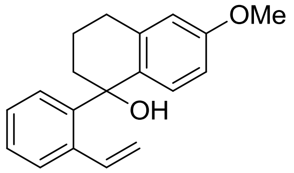

1D

^{1}H NMR (500 MHz, $CDCl_3$) δ 7.95 (d, *J* = 8.0 Hz, 1H), 7.41 (d, *J* = 7.5 Hz, 1H), 7.35 (t, *J* = 7.5 Hz, 1H), 7.28 (t, *J* = 7.5 Hz, 1H), 6.84 (d, *J* = 8.5 Hz, 1H), 6.70 (d, *J* = 2.0

Hz, 1H), 6.63 (dd, *J* = 8.5, 2.5 Hz, 1H), 6.31 (dd, *J* = 17.5, 11.0 Hz, 1H), 5.39 (d, *J* = 17.5 Hz, 1H), 4.97 (d, *J* = 11.0 Hz, 1H), 3.79 (s, 3H), 2.91 - 2.86 (m, 2H), 2.28 (td, *J* = 13.5, 2.5 Hz, 1H), 2.15 - 2.05 (m, 2H), 2.01 - 1.95 (m, 1H), 1.89 - 1.83 (m, 1H).
^{13}C NMR (125 MHz, $CDCl_3$) δ 158.63, 145.51, 138.57, 136.52, 135.72, 134.56, 129.27, 127.70, 127.24, 127.14, 126.18, 114.98, 113.24, 113.04, 74.22, 55.13, 38.51, 30.15, 19.63.
HRMS (ESI) m/z calcd. for $C_{19}H_{19}O$ $[M\text{-}OH]^+$ 263.1430, found 263.1419.

5,7-dimethyl-1-(2-vinylphenyl)-1,2,3,4-tetrahydronaphthalen-1-ol

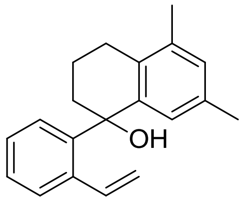

1E

^{1}H NMR (500 MHz, $CDCl_3$) δ 7.89 (d, *J* = 7.5 Hz, 1H), 7.42 (d, *J* = 7.5 Hz, 1H), 7.37 - 7.32 (m, 1H), 7.31 - 7.27 (m, 1H), 6.93 (s, 1H), 6.63 (s, 1H), 6.33 (t, *J* = 15.0 Hz, 1H), 5.40 (dd, *J* = 17.5, 1.5 Hz, 1H), 4.97 (dd, *J* = 11.5, 1.5 Hz, 1H), 2.91 - 2.84 (m, 1H), 2.65 - 2.55 (m, 1H), 2.33 - 2.25 (m, 4H), 2.15 - 2.05 (m, 5H), 2.00 - 1.82 (m, 2H).
^{13}C NMR (125 MHz, $CDCl_3$) δ 145.69, 141.76, 136.76, 136.03, 135.84, 135.71, 132.51, 130.12, 127.65, 127.17, 127.07, 126.36, 126.00, 114.87, 75.03, 37.98, 26.32, 20.90, 19.79, 19.35.
HRMS (ESI) m/z calcd. for $C_{20}H_{21}$ $[M\text{-}OH]^+$ 261.1643, found 261.1671

1-tosyl-4-(2-vinylphenyl)-1,2,3,4-tetrahydroquinolin-4-ol

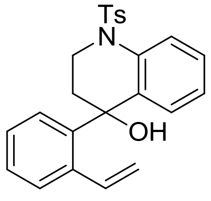

1F

^{1}H NMR (500 MHz, $CDCl_3$) δ 7.84 (d, *J* = 8.5 Hz, 1H), 7.74 (d, *J* = 8.0 Hz, 2H), 7.56 (d, *J* = 6.0 Hz, 1H), 7.35 (d, *J* = 7.5 Hz, 1H), 7.28 – 7.24 (m, 3H), 7.24 - 7.19 (m, 2H), 6.94 (t, *J* = 7.5 Hz, 1H), 6.88 (d, *J* = 7.5 Hz, 1H), 6.19 (dd, *J* = 16.5, 11.0 Hz, 1H), 5.29 (d, *J* = 17.0 Hz, 1H), 4.82 (d, *J* = 10.5 Hz, 1H), 4.36 - 4.20 (m, 1H), 3.85 (t, *J* = 13.0 Hz, 1H), 2.40 (s, 3H), 2.38 - 2.32 (m, 1H), 2.11 (s, 1H), 1.98 - 1.92 (m, 1H).
^{13}C NMR (125 MHz, $CDCl_3$) δ 143.86, 142.83, 137.34, 136.39, 135.89, 135.80, 134.09, 129.81, 128.54, 128.50, 127.86, 127.71, 127.27, 127.11, 126.60, 124.69, 121.90, 115.59, 73.09, 43.29, 36.36, 21.51.
HRMS (ESI) m/z calcd. for $C_{24}H_{24}NO_3S$ $[M+H]^+$ 406.1477, found 406.1471.

4-(2-vinylphenyl)thiochroman-4-ol

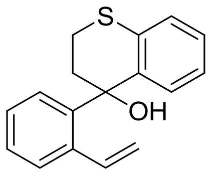

1G

^{1}H NMR (500 MHz, $CDCl_3$) δ 7.96 (d, *J* = 7.5 Hz, 1H), 7.44 (d, *J* = 7.5 Hz, 1H), 7.37 (t, *J* = 7.5 Hz, 1H), 7.31 (t, *J* = 7.5 Hz, 1H), 7.22 (d, *J* = 8.0 Hz, 1H), 7.18 - 7.10 (m, 1H), 6.92 (d, *J* = 4.0 Hz, 2H), 6.30 (dd, *J* = 17.0, 11.0 Hz, 1H), 5.41 (d, *J* = 17.0 Hz, 1H), 5.00 (d, *J* = 11.0 Hz, 1H), 3.48 (td, *J* = 13.0, 2.5 Hz, 1H), 2.78 - 2.73 (m, 1H), 2.64 - 2.49 (m, 1H), 2.29 - 2.11 (m, 2H).

^{13}C NMR (125 MHz, $CDCl_3$) δ 144.25, 138.51, 135.82, 135.42, 133.36, 129.16, 127.96, 127.63, 127.60, 127.47, 127.10, 125.98, 125.06, 115.57, 73.16, 37.03, 23.19.

HRMS (ESI) m/z calcd. for $C_{17}H_{15}S$ $[M-OH]^+$ 251.0889, found 251.0879.

5-(2-vinylphenyl)-6,7,8,9-tetrahydro-5H-benzo[7]annulen-5-ol

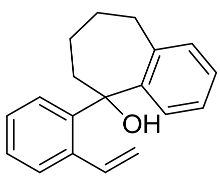

1H

^{1}H NMR (500 MHz, $CDCl_3$) δ 7.50 (d, *J* = 7.5 Hz, 1H), 7.35 - 7.30 (m, 3H), 7.27 - 7.22 (m, 2H), 7.22 - 7.10 (m, 3H), 5.54 (d, *J* = 17.0 Hz, 1H), 5.20 (d, *J* = 11.0 Hz, 1H), 3.01 - 2.94 (m, 1H), 2.88 - 2.75 (m, 2H), 2.41 (d, *J* = 5.5 Hz, 1H), 2.14 - 2.07 (m, 1H), 2.07 - 1.97 (m, 1H), 1.82 - 1.75 (m, 2H), 1.72 - 1.63 (m, 1H).

^{13}C NMR (125 MHz, $CDCl_3$) δ 145.77, 143.85, 141.50, 138.24, 137.97, 130.85, 128.77, 127.77, 127.46, 127.37, 127.34, 126.95, 126.24, 115.12, 81.24, 40.11, 35.83, 27.11, 24.46.

HRMS (ESI) m/z calcd. for $C_{19}H_{21}O$ $[M+H]^+$ 265.1592, found 265.1587.

3-fluoro-5-(2-vinylphenyl)-6,7,8,9-tetrahydro-5H-benzo[7]annulen-5-ol

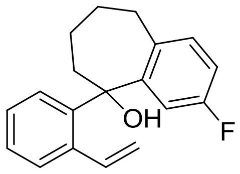

1I

^{1}H NMR (500 MHz, $CDCl_3$) δ 7.45 (d, *J* = 8.0 Hz, 1H), 7.28 (t, *J* = 7.5 Hz, 1H), 7.23 - 7.03 (m, 5H), 6.90 - 85 (m, 1H), 5.50 (dd, *J* = 17.0, 1.5 Hz, 1H), 5.19 (dd, *J* = 11.0, 1.5 Hz, 1H), 2.92 - 2.61 (m, 3H), 2.45 - 2.24 (m, 1H), 2.09 - 2.02 (m, 1H), 1.95 - 1.88 (m, 1H), 1.84 - 1.67 (m, 1H), 1.67 - 1.48 (m, 2H).

^{13}C NMR (125 MHz, $CDCl_3$) δ 161.53 (d, *J* = 241.9 Hz), 148.52 (d, *J* = 5.9 Hz), 142.30, 138.40, 138.19, 137.01 (d, *J* = 3.1 Hz), 132.16 (d, *J* = 7.5 Hz), 129.24, 127.90,

127.46, 127.33, 115.59, 114.60 (d, J = 23.1 Hz), 113.64 (d, J = 20.4 Hz), 81.11, 40.39, 35.27, 27.27, 24.83.
^{19}F NMR (376 MHz, $CDCl_3$) δ -116.66.
HRMS (ESI) m/z calcd. for $C_{19}H_{20}FO$ $[M+H]^+$ 283.1498, found 283.1493.

(*E*)-7-bromo-1-(2-(prop-1-en-1-yl)phenyl)-1,2,3,4-tetrahydronaphthalen-1-ol

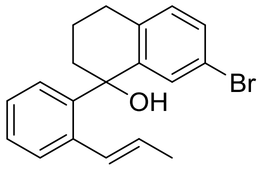

1M

^{1}H NMR (500 MHz, $CDCl_3$) δ 7.90 (d, J = 7.5 Hz, 1H), 7.38 - 7.29 (m, 4H), 7.16 - 7.13 (m, 1H), 7.10 (d, J = 8.0 Hz, 1H), 5.98 (d, J = 15.5 Hz, 1H), 5.89 - 5.81 (m, 1H), 2.97 - 2.90 (m, 1H), 2.87 - 2.78 (m, 1H), 2.43 - 2.32 (m, 2H), 2.09 - 1.96 (m, 2H), 1.92 - 1.86 (m, 1H), 1.68 (dd, J = 6.5, 2.0 Hz, 3H).
^{13}C NMR (125 MHz, $CDCl_3$) δ 144.23, 143.85, 135.92, 135.62, 130.80, 130.61, 130.42, 130.41, 128.09, 127.29, 127.02, 126.49, 125.84, 120.06, 74.32, 37.99, 29.38, 19.38, 18.59.
HRMS (ESI) m/z calcd. for $C_{19}H_{18}Br$ $[M-OH]^+$ 325.0592, found 325.0582.

6-fluoro-1-(2-vinylphenyl)-2,3-dihydro-1H-inden-1-ol

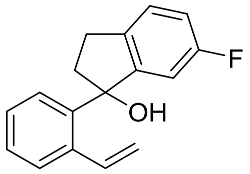

1N

^{1}H NMR (500 MHz, $CDCl_3$) δ 7.52 (d, J = 7.6 Hz, 1H), 7.28 - 7.21 (m, 2H), 7.20 - 7.15 (m, 2H), 6.99 (td, J = 9.0, 2.4 Hz, 1H), 6.96 - 6.88 (m, 1H), 6.78 (dd, J = 8.5, 2.5 Hz, 1H), 5.52 (dd, J = 17.5, 1.5 Hz, 1H), 5.13 (dd, J = 11.0, 1.5 Hz, 1H), 3.10 - 2.99 (m, 1H), 2.89 - 2.67 (m, 2H), 2.43 - 2.36 (m, 1H), 2.35 (s, 1H).
^{13}C NMR (125 MHz, $CDCl_3$) δ 16.36 (d, J = 243 Hz), 149.81 (d, J = 6.9 Hz), 141.48, 138.91 (d, J = 2.4 Hz), 136.64, 136.54, 127.82, 127.80, 127.16, 126.46, 126.10 (d, J = 8.4 Hz), 115.59 (d, J = 22.5 Hz), 115.24, 110.96 (d, J = 22.0 Hz), 85.89 (d, J = 1.9 Hz), 43.37, 29.08.
^{19}F NMR (376 MHz, $CDCl_3$) δ -115.57 (s).
HRMS (ESI) m/z calcd. for $C_{17}H_{14}F$ $[M-OH]^+$ 237.1080, found 237.1066.

1-(2-allylphenyl)-1,2,3,4-tetrahydronaphthalen-1-ol

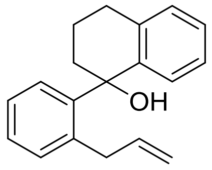

1O

^{1}H NMR (500 MHz, $CDCl_3$) δ 7.91 (d, *J* = 7.5 Hz, 1H), 7.28 - 7.24 (m, 2H), 7.19 - 7.14 (m, 3H), 7.09 - 7.04 (m, 1H), 6.89 (d, *J* = 7.5 Hz, 1H), 5.61 - 5.52 (m, 1H), 4.89 (d, *J* = 10.0 Hz, 1H), 4.79 (d, *J* = 17.0 Hz, 1H), 3.03 (dd, *J* = 15.5, 7.0 Hz, 1H), 2.95 - 2.82 (m, 3H), 2.20 - 2.05 (m, 3H), 2.02 (s, 1H), 1.91 - 1.85 (m, 1H).
^{13}C NMR (100 MHz, $CDCl_3$) δ 145.88, 142.66, 137.54, 136.69, 136.22, 131.03, 129.08, 127.86, 127.42, 126.94, 126.83, 126.52, 125.64, 115.57, 74.53, 38.62, 37.12, 29.80, 19.66.
HRMS (ESI) m/z calcd. for $C_{19}H_{21}O$ $[M+H]^+$ 265.1592, found 265.1587.

1-(2-allylphenyl)-7-fluoro-1,2,3,4-tetrahydronaphthalen-1-ol

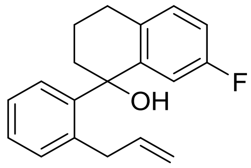

1P

^{1}H NMR (400 MHz, $CDCl_3$) δ 7.89 (dd, *J* = 5.6, 3.6 Hz, 1H), 7.33 - 7.27 (m, 2H), 7.24 - 7.14 (m, 2H), 6.92 (td, *J* = 8.4, 2.8 Hz, 1H), 6.63 (dd, *J* = 9.6, 2.8 Hz, 1H), 5.72 - 5.61 (m, 1H), 4.96 (dq, *J* = 10.0, 1.6 Hz, 1H), 4.83 (dq, *J* = 16.8, 1.6 Hz, 1H), 3.09 (dd, *J* = 16.0, 6.8 Hz, 1H), 2.99 - 2.84 (m, 3H), 2.23 - 2.04 (m, 4H), 1.96 - 1.88 (m, 1H).
^{13}C NMR (100 MHz, $CDCl_3$) δ 161.53 (d, *J* = 243.1 Hz), 145.13, 144.45 (d, *J* = 5.9 Hz), 137.39, 136.13, 132.28 (d, *J* = 3.0 Hz), 131.29, 130.63 (d, *J* = 7.5 Hz), 127.25, 126.48, 125.82, 115.72, 114.73 (d, *J* = 21.2 Hz), 114.17 (d, *J* = 21.1 Hz), 74.54, 38.34, 37.11, 29.08, 19.66.
^{19}F NMR (376 MHz, $CDCl_3$) δ -115.49.
HRMS (ESI) m/z calcd. for $C_{19}H_{20}FO$ $[M+H]^+$ 283.1498, found 283.1493.

1-(2-allylphenyl)-7-bromo-1,2,3,4-tetrahydronaphthalen-1-ol

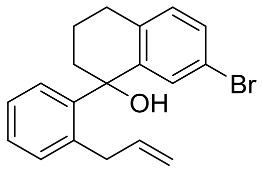

1Q

^{1}H NMR (400 MHz, $CDCl_3$) δ 7.85 (dd, *J* = 5.6, 3.6 Hz, 1H), 7.32 - 7.26 (m, 3H), 7.21 (dd, *J* = 5.6, 3.6 Hz, 1H), 7.08 - 7.02 (m, 2H), 5.69 – 5.57 (m, 1H), 4.95 (dq, *J* = 10.0, 1.6 Hz, 1H), 4.80 (dq, *J* = 16.8, 1.6 Hz, 1H), 3.08 (dd, *J* = 15.6, 6.4 Hz, 1H), 2.95 - 2.77 (m, 3H), 2.21 (s, 1H), 2.19 - 2.01 (m, 3H), 1.92 - 1.84 (m, 1H).

^{13}C NMR (100 MHz, $CDCl_3$) δ 144.93, 144.76, 137.22, 135.99, 135.59, 131.36, 130.84, 130.70, 130.51, 127.24, 126.51, 125.82, 120.15, 115.67, 74.43, 38.48, 37.10, 29.29, 19.39.
HRMS (ESI) m/z calcd. for $C_{19}H_{20}BrO$ $[M+H]^+$ 343.0698, found 343.0692.

4-(2-allylphenyl)-6-methylchroman-4-ol

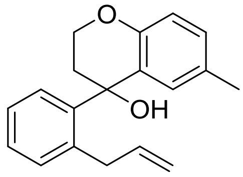

1R

^1H NMR (400 MHz, $CDCl_3$) δ 7.94 - 7.88 (m, 1H), 7.31 – 7.27 (m, 2H), 7.23 - 7.19 (m, 1H), 7.00 (dd, *J* = 8.4, 2.0 Hz, 1H), 6.83 (d, *J* = 8.4 Hz, 1H), 6.66 (d, *J* = 1.6 Hz, 1H), 5.68 - 5.57 (m, 1H), 4.93 (dd, *J* = 10.4, 1.6 Hz, 1H), 4.81 (dd, *J* = 17.2, 1.6 Hz, 1H), 4.25 (dt, *J* = 11.4, 2.0 Hz, 1H), 4.29 – 4.23 (m, 1H), 3.09 (dd, *J* = 15.6, 6.4 Hz, 1H), 2.94 (dd, *J* = 15.6, 6.4 Hz, 1H), 2.52 - 2.42 (m, 1H), 2.38 (s, 1H), 2.14 (s, 3H), 2.01 (dt, *J* = 14.4, 2.8 Hz, 1H).
^{13}C NMR (101 MHz, $CDCl_3$) δ 152.13, 143.97, 137.41, 136.45, 131.40, 130.43, 130.18, 128.71, 127.95, 127.33, 126.96, 125.74, 117.09, 115.64, 70.93, 63.27, 37.37, 36.99, 20.42.
HRMS (ESI) m/z calcd. for $C_{19}H_{19}O$ $[M-OH]^+$ 263.1430, found 263.1426.

4-(2-allylphenyl)-1-tosyl-1,2,3,4-tetrahydroquinolin-4-ol

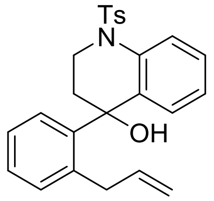

1S

^1H NMR (500 MHz, $CDCl_3$) δ 7.93 (d, *J* = 8.0 Hz, 1H), 7.71 (d, *J* = 8.0 Hz, 2H), 7.53 (d, *J* = 7.5 Hz, 1H), 7.25 - 7.19 (m, 4H), 7.16 - 7.09 (m, 2H), 6.98 (t, *J* = 8.0 Hz, 1H), 6.87 (d, *J* = 8.0 Hz, 1H), 5.50 - 5.40 (m, 1H), 4.87 (d, *J* = 10.0 Hz, 1H), 4.69 (d, *J* = 17.0 Hz, 1H), 4.45 - 4.38 (m, 1H), 3.93 - 3.95 (m, 1H), 2.81 - 2.72 (m, 1H), 2.64 (dd, *J* = 15.0, 5.0 Hz, 1H), 2.39 (s, 3H), 2.19 - 2.11 (m, 1H), 2.04 (s, 1H), 1.97 - 1.92 (m, 1H).
^{13}C NMR (125 MHz, $CDCl_3$) δ 143.79, 143.60, 137.44, 137.29, 136.40, 136.24, 134.92, 131.35, 129.92, 128.58, 128.28, 127.45, 127.09, 126.99, 125.79, 124.99, 122.70, 115.77, 73.22, 43.40, 37.09, 36.38, 21.54.
HRMS (ESI) m/z calcd. for $C_{25}H_{26}NO_3S$ $[M+H]^+$ 420.1633, found 420.1628.

1-(2-allylphenyl)-4-methyl-1,2,3,4-tetrahydronaphthalen-1-ol

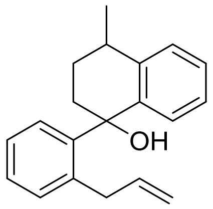

1T

^{1}H NMR (500 MHz, $CDCl_3$) δ 7.94 (d, *J* = 6.5 Hz, 1H), 7.32 - 7.26 (m, 4H), 7.24 - 7.19 (m, 1H), 7.11 (t, *J* = 7.5 Hz, 1H), 6.94 (d, *J* = 7.5 Hz, 1H), 5.65 - 5.55 (m, 1H), 4.93 (d, *J* = 10.0 Hz, 1H), 4.83 (d, *J* = 17.0 Hz, 1H), 3.10 - 2.95 (m, 2H), 2.87 (dd, *J* = 16.0, 6.5 Hz, 1H), 2.32 - 2.24 (m, 1H), 2.13 - 2.08 (m, 2H), 1.97 - 1.91 (m, 2H), 1.49 (d, *J* = 6.5 Hz, 3H).
^{13}C NMR (125 MHz, $CDCl_3$) δ 146.14, 142.55, 141.40, 137.59, 136.31, 131.08, 127.93, 127.70, 126.97, 126.74, 126.69, 126.48, 125.66, 115.60, 75.09, 38.02, 37.11, 32.98, 29.00, 20.94.
HRMS (ESI) m/z calcd. for $C_{20}H_{23}O$ $[M+H]^+$ 279.1479, found 279.1473.

5-(2-allylphenyl)-3-fluoro-6,7,8,9-tetrahydro-5H-benzo[7]annulen-5-ol

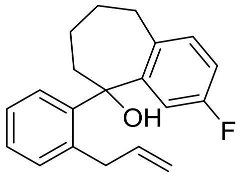

1U

^{1}H NMR (400 MHz, $CDCl_3$) δ 7.28 - 7.21 (m, 3H), 7.16 - 7.11 (m, 1H), 7.07 (dd, *J* = 8.4, 6.0 Hz, 1H), 6.91 (dd, *J* = 11.2, 2.4 Hz, 1H), 6.83 (td, *J* = 8.0, 2.8 Hz, 1H), 5.93 - 5.82 (m, 1H), 5.03 (dq, *J* = 10.0, 1.6 Hz, 1H), 4.95 (dq, *J* = 16.8, 1.6 Hz, 1H), 3.49 - 3.37 (m, 2H), 2.98 - 2.88 (m, 1H), 2.78 - 2.69 (m, 1H), 2.65 - 2.55 (m, 1H), 2.33 (s, 1H), 2.15 - 2.08 (m, 1H), 2.02 - 1.94 (m, 1H), 1.74 - 1.66 (m, 3H).
^{13}C NMR (100 MHz, $CDCl_3$) δ 161.36 (d, *J* = 241.7 Hz), 148.5 (d, *J* = 6.0 Hz), 144.20, 138.18, 137.06 (d, *J* = 3.1 Hz), 132.12 (d, *J* = 7.5 Hz), 132.04, 128.31, 127.44, 127.28, 126.00, 115.78, 115.10 (d, *J* = 22.9 Hz), 113.65 (d, *J* = 20.5 Hz), 81.01, 39.90, 38.28, 34.91, 27.16, 24.56.
HRMS (ESI) m/z calcd. for $C_{20}H_{22}FO$ $[M+H]^+$ 297.1655, found 297.1649.

1-(2-allylphenyl)-6-fluoro-2,3-dihydro-1H-inden-1-ol

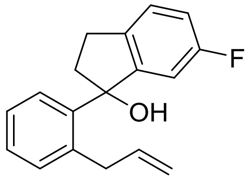

1W

^{1}H NMR (400 MHz, $CDCl_3$) δ 7.28 - 7.21 (m, 3H), 7.13 - 7.08 (m, 2H), 6.99 (td, *J* = 8.8, 2.4 Hz, 1H), 6.81 (dd, *J* = 8.8, 2.4 Hz, 1H), 6.00 - 5.87 (m, 1H), 5.03 (dd, *J* =

10.0, 1.6 Hz, 1H), 4.94 (dq, J = 17.2, 1.6 Hz, 1H), 3.49 (dd, J = 16.0, 6.4 Hz, 1H), 3.35 (dd, J = 16.0, 6.4 Hz, 1H), 3.12 - 3.00 (m, 1H), 2.85 - 2.76 (m, 1H), 2.70 - 2.61 (m, 1H), 2.51 - 2.43 (m, 1H), 2.28 (s, 1H).

^{13}C NMR (100 MHz, $CDCl_3$) δ 162.39 (d, J = 242.9 Hz), 150.38 (d, J = 6.9 Hz), 142.42, 138.78 (d, J = 2.4 Hz), 138.42, 137.68, 131.77, 127.60, 126.89, 126.15 (d, J = 8.4 Hz), 125.62, 115.67, 115.55 (d, J = 23.7 Hz), 110.89 (d, J = 22.1 Hz), 86.19, 43.68, 37.83, 29.06.

HRMS (ESI) m/z calcd. for $C_{18}H_{18}FO$ $[M+H]^+$ 269.1336, found 269.1339.

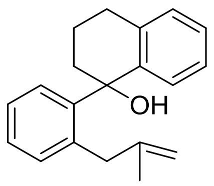

1X

^{1}H NMR (400 MHz, $CDCl_3$) δ 7.91 - 7.86 (m, 1H), 7.28 - 7.20 (m, 2H), 7.19 - 7.11 (m, 3H), 7.06 - 7.01 (m, 1H), 6.87 (d, J = 7.6 Hz, 1H), 4.77 (d, J = 0.8 Hz, 1H), 4.48 (d, J = 0.8 Hz, 1H), 3.02 (d, J = 16.0 Hz, 1H), 2.96 - 2.77 (m, 3H), 2.24 - 1.99 (m, 4H), 1.90 - 1.82 (m, 1H), 1.39 (s, 3H).

13C NMR (100 MHz, CDCl3) δ 146.35, 145.37, 142.59, 136.76, 135.57, 130.82, 129.02, 127.93, 127.39, 126.87, 126.68, 125.70, 112.76, 74.72, 41.02, 38.32, 29.90, 22.42, 19.67.

HRMS (ESI) m/z calcd. for $C_{20}H_{21}$ $[M-OH]^+$ 261.1643, found 261.1635.

Procedure for synthesis of substrate 1V and 1J to 1L:

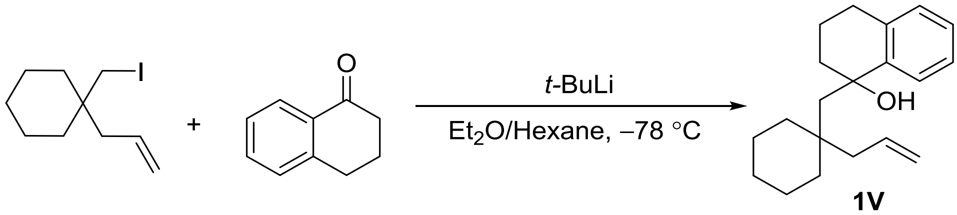

1V

To a solution of 1-allyl-1-(iodomethyl)cyclohexane (0.67 g, 2.3 mmol) in Et_2O/hexane (4 mL and 6 mL) was added *t*-BuLi (2.1 mL, 2.4 M in *n*-hexane, 5.1 mmol) at –78 °C. The reaction mixture was stirred for 1 h at –78°C. 3,4-Dihydronaphthalen-1(2H)-one (0.33 g, 2.3 mmol) in dry Et_2O (1 mL) was slowly added, and the reaction mixture was stirred for 2 h. Saturated NH_4Cl solution was added and the solution was extracted with EtOAc (3 × 20 mL). The organic layer was dried over anhydrous Na_2SO_4, filtered and concentrated to afford the crude product, which was purified by flash column chromatography to afford **1V** (106 mg, 16%).

The synthesis of substrates **1J** to **1L** is similar to that reported for **1V**.

1-((1-allylcyclohexyl)methyl)-1,2,3,4-tetrahydronaphthalen-1-ol

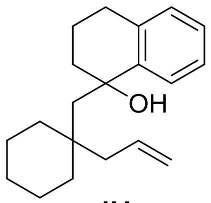

1V

^{1}H NMR (500 MHz, $CDCl_3$) δ 7.64 (d, *J* = 7.5 Hz, 1H), 7.23 (t, *J* = 7.5 Hz, 1H), 7.17 (t, *J* = 7.5 Hz, 1H), 7.07 (d, *J* = 7.5 Hz, 1H), 6.05 - 5.95 (m, 1H), 5.14 - 5.07 (m, 2H), 2.81 (t, *J* = 6.0 Hz, 2H), 2.44 (dd, *J* = 14.5, 7.5 Hz, 1H), 2.37 - 2.30 (m, 2H), 1.97 - 1.87 (m, 4H), 1.81 (s, 1H), 1.63 - 1.54 (m, 2H), 1.54 - 1.44 (m, 6H), 1.42 - 1.38 (m, 2H), 1.37 - 1.29 (m, 1H).
^{13}C NMR (125 MHz, $CDCl_3$) δ 145.26, 136.18, 135.85, 128.66, 126.71, 126.41, 126.09, 116.79, 74.56, 48.34, 41.35, 37.69, 37.44, 36.83, 36.79, 29.50, 26.14, 21.84, 21.64, 19.86.
HRMS (unstable to be detected).

6-(2-vinylphenyl)-3,4,5,6-tetrahydro-2H-benzo[b]oxocin-6-ol

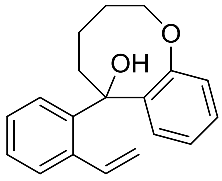

1J

^{1}H NMR (500 MHz, $CDCl_3$) δ 7.64 (brs, 1H), 7.54 (brs, 1H), 7.37 – 7.30 (m, 2H), 7.28 – 7.22 (m, 1H), 7.10 (dd, *J* = 8.0, 1.0 Hz, 1H), 6.99 (dd, *J* = 17.3, 10.9 Hz, 1H), 6.92 (t, *J* = 7.5 Hz, 1H), 5.36 (dd, *J* = 17.3, 1.6 Hz, 1H), 4.93 (d, *J* = 11.0 Hz, 1H), 4.28 (dd, *J* = 9.6, 5.4 Hz, 2H), 2.52 (brs, 2H), 2.14 – 2.05 (m, 1H), 2.05 – 1.92 (m, 1H), 1.74 (d, *J* = 10.8 Hz, 1H), 1.49 – 1.41 (m, 1H).
^{13}C NMR (126 MHz, $CDCl_3$) δ 154.73, 141.20, 138.18, 128.86, 128.74, 128.29, 127.53, 126.90, 125.91, 124.49, 122.52, 112.96, 78.19, 76.85, 42.91, 25.55, 22.09.
HRMS (ESI) m/z calcd. for $C_{19}H_{19}O$ $[M-OH]^+$ 263.1430, found 263.1426.

9-(2-vinylphenyl)-2,3,4,5,6,7,8,9-octahydrobenzo[b][1]oxacycloundecin-9-ol

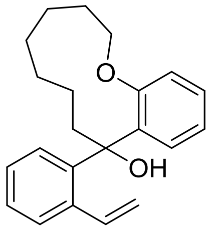

1K

This compound was not stable, ^{1}H NMR (400 MHz, $CDCl_3$) δ 7.71 - 7.66 (m, 1H), 7.61 (dd, *J* = 5.6, 3.6 Hz, 1H), 7.35 (dd, *J* = 5.6, 3.6 Hz, 2H), 7.27 - 7.21 (m, 1H), 7.12 (dd, *J* = 17.6, 10.8 Hz, 1H), 6.98 (d, *J* = 8.4 Hz, 1H), 6.84 - 6.74 (m, 2H), 5.44 (dd, *J* = 17.6, 1.6 Hz, 1H), 5.19 (s, 1H), 5.03 - 4.96 (m, 1H), 4.40 - 4.27 (m, 2H), 2.58

- 2.42 (m, 2H), 2.00 - 1.86 (m, 2H), 1.86 - 1.75 (m, 2H), 1.75 - 1.60 (m, 3H), 1.58 - 1.46 (m, 2H), 1.46 - 1.29 (m, 1H).
^{13}C NMR (100 MHz, $CDCl_3$) δ^{13}C NMR (101 MHz, $CDCl_3$) δ 156.06, 144.14, 138.16, 136.70, 135.36, 129.56, 127.96, 127.77, 127.27, 126.67, 126.15, 120.63, 112.65, 112.08, 80.64, 69.47, 39.92, 26.05, 25.84, 25.19, 23.58, 21.25.
HRMS (ESI) m/z calcd. for $C_{22}H_{25}O_1$ $[M\text{-}OH]^+$ 305.1905, found 305.1897.

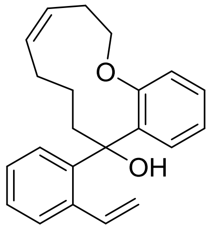

1L

This compound was not stable, ^{1}H NMR (500 MHz, $CDCl_3$) δ 7.66 - 7.61 (m, 1H), 7.60 - 7.57 (m, 1H), 7.36 - 7.31 (m, 2H), 7.23 (td, *J* = 8.0, 1.5 Hz, 1H), 7.03 - 6.97 (m, 1H), 6.96 - 6.90 (m, 1H), 6.79 - 6.67 (m, 2H), 5.75 - 5.67 (m, 1H), 5.55 - 5.48 (m, 1H), 5.45 (dd, *J* = 17.5, 1.5 Hz, 1H), 4.95 (dd, *J* = 11.0, 1.5 Hz, 1H), 4.53 (s, 1H), 4.45 - 4.40 (m, 1H), 4.25 - 4.11 (m, 1H), 2.71 - 2.54 (m, 2H), 2.52 - 2.41 (m, 2H), 2.34 - 2.18 (m, 1H), 1.93 - 1.74 (m, 2H), 1.61 - 1.52 (m, 1H).
^{13}C NMR (125 MHz, $CDCl_3$) δ 155.36, 141.03, 138.66, 138.54, 134.61, 131.97, 127.99, 127.90, 127.6, 127.08, 127.02, 126.88, 120.46, 120.26, 112.78, 112.30, 80.33, 67.55, 39.87, 27.57, 26.21, 23.25.
HRMS (ESI) m/z calcd. for $C_{22}H_{25}O_2$ $[M+H]^+$ 321.1855, found 321.1849.

General procedure for azidation reaction

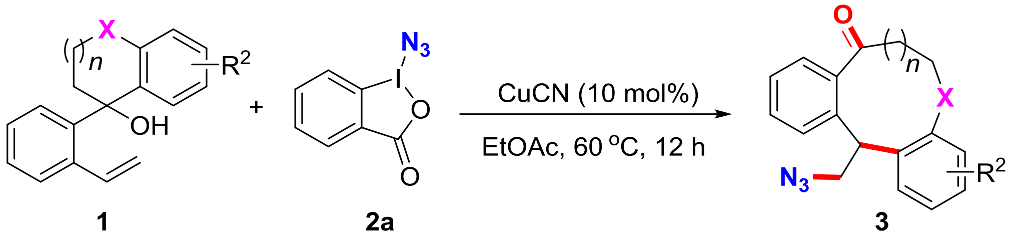

Under argon, a 25 mL Schlenk tube equipped with a magnetic stir bar was charged with **1** (0.3 mmol, 1.0 equiv), **2a** (104 mg, 0.36 mmol, 1.2 equiv), CuCN (2.7 mg, 0.03 mmol, 0.1 equiv) and EtOAc (3.0 mL). The sealed tube was then stirred at 60 °C for 12 h. After completion (monitored by TLC), EtOAc (30 mL) was added. The organic phase was washed with saturated $NaHCO_3$ solution (2 × 5 mL), dried over anhydrous Na_2SO_4, filtered and concentrated to afford the crude product, which was purified by flash column chromatography to afford the corresponding product **3**.

For preparation of **3M**, **2a** (2.0 equiv) was added. 1,10-Phenanthroline (1.5 equiv) was used instead of CuCN, and the reaction was permitted to stir 24 h at 60 °C.

13-(azidomethyl)-11-bromo-6,7,8,13-tetrahydro-5H-dibenzo[a,d][9]annulen-5-one

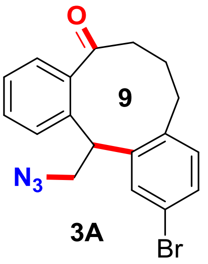

(72%), ^{1}H NMR (400 MHz, $CDCl_3$) δ 7.48 (td, *J* = 7.6, 1.2 Hz, 1H), 7.39 (d, *J* = 7.6 Hz, 1H), 7.31 - 7.23 (m, 2H), 7.12 (dd, *J* = 7.6, 0.8 Hz, 1H), 7.02 (d, *J* = 8.0 Hz, 1H), 6.94 (d, *J* = 2.0 Hz, 1H), 4.89 (t, *J* = 7.6 Hz, 1H), 4.09 (dd, *J* = 12.0, 6.8 Hz, 1H), 3.90 (dd, *J* = 12.0, 8.4 Hz, 1H), 3.14 (td, *J* = 13.6, 3.6 Hz, 1H), 2.99 - 2.90 (m, 1H), 2.81 (dt, *J* = 14.4, 4.0 Hz, 1H), 2.45 - 2.37 (m, 1H), 2.37 - 2.28 (m, 1H), 2.13 - 2.01 (m, 1H).

^{13}C NMR (100 MHz, $CDCl_3$) δ 209.63, 143.79, 142.46, 138.19, 137.67, 131.67, 131.07, 130.41, 130.28, 127.26, 126.65, 124.38, 120.96, 54.70, 41.31, 41.03, 31.63, 28.35.

HRMS (APCI) m/z calcd. for $C_{18}H_{17}BrN_3O$ $[M+H]^+$ 370.0555, found 370.0550.

13-(azidomethyl)-11-fluoro-6,7,8,13-tetrahydro-5H-dibenzo[a,d][9]annulen-5-one

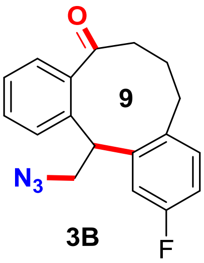

3B

(70%), ^{1}H NMR (400 MHz, $CDCl_3$) δ 7.47 (td, *J* = 7.6, 1.2 Hz, 1H), 7.38 (d, *J* = 7.6 Hz, 1H), 7.27 (t, *J* = 7.2 Hz, 1H), 7.14 - 7.08 (m, 2H), 6.84 (td, *J* = 8.4, 2.4 Hz, 1H), 6.49 (dd, *J* = 9.6, 2.4 Hz, 1H), 4.94 (t, *J* = 7.2 Hz, 1H), 4.08 (dd, *J* = 12.0, 6.4 Hz, 1H), 3.89 (dd, *J* = 12.0, 8.4 Hz, 1H), 3.15 (td, *J* = 13.6, 3.6 Hz, 1H), 3.03 - 2.93 (m, 1H), 2.84 (dt, *J* = 14.4, 3.6 Hz, 1H), 2.45 - 2.36 (m, 1H), 2.34 - 2.26 (m, 1H), 2.15 - 2.05 (m, 1H).

^{13}C NMR (100 MHz, $CDCl_3$) δ 209.53, 161.68 (d, *J* = 244.5 Hz), 143.76, 142.32 (d, *J* = 6.5 Hz), 138.03, 134.93 (d, *J* = 3.1 Hz), 131.58 (d, *J* = 7.9 Hz), 130.35, 127.18, 126.78, 124.24, 115.29 (d, *J* = 20.9 Hz), 113.86 (d, *J* = 21.2 Hz), 54.62, 41.53, 41.03, 31.47, 28.78.

^{19}F NMR (376 MHz, $CDCl_3$) δ -114.24.

HRMS (APCI) m/z calcd. for $C_{18}H_{17}FN_3O$ $[M+H]^+$ 310.1356, found 310.1350.

13-(azidomethyl)-6,7,8,13-tetrahydro-5H-dibenzo[a,d][9]annulen-5-one

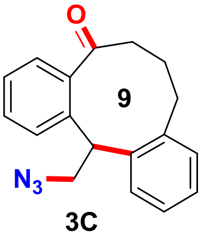

3C

(62%), ^{1}H NMR (400 MHz, $CDCl_3$) δ 7.49 - 7.42 (m, 2H), 7.28 - 7.24 (m, 1H), 7.17 - 7.13 (m, 2H), 7.13 - 7.06 (m, 2H), 6.81 (d, *J* = 7.6 Hz, 1H), 4.99 (t, *J* = 7.6 Hz, 1H), 4.09 (dd, *J* = 12.0, 7.2 Hz, 1H), 3.93 (dd, *J* = 12.0, 8.4 Hz, 1H), 3.22 (td, *J* = 13.6, 3.6 Hz, 1H), 3.04 - 2.96 (m, 1H), 2.86 (dt, *J* = 13.6, 3.6 Hz, 1H), 2.45 - 2.38 (m, 1H), 2.38 - 2.29 (m, 1H), 2.18 - 2.08 (m, 1H).

^{13}C NMR (100 MHz, $CDCl_3$) δ 209.70, 143.90, 140.33, 139.15, 138.85, 130.11, 129.92, 127.84, 127.47, 127.42, 126.87, 126.61, 124.34, 54.79, 41.30, 41.12, 32.09, 28.80

HRMS (APCI) m/z calcd. for $C_{18}H_{18}N_3O$ $[M+H]^+$ 292.1450, found 292.1444.

13-(azidomethyl)-10-methoxy-6,7,8,13-tetrahydro-5H-dibenzo[a,d][9]annulen-5-one

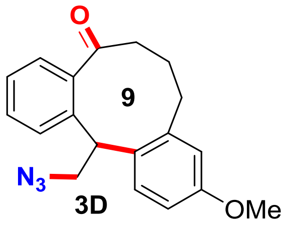

(41%), ^{1}H NMR (400 MHz, $CDCl_3$) δ 7.45 (td, *J* = 7.6, 1.2 Hz, 1H), 7.40 (d, *J* = 7.6 Hz, 1H), 7.27 - 7.21 (m, 1H), 7.10 (dd, *J* = 7.6, 1.2 Hz, 1H), 6.70 (d, *J* = 8.8 Hz, 1H), 6.65 - 6.61 (m, 2H), 4.91 (t, *J* = 7.6 Hz, 1H), 4.06 (dd, *J* = 12.0, 6.8 Hz, 1H), 3.87 (dd, *J* = 12.0, 8.0 Hz, 1H), 3.74 (s, 3H), 3.18 (td, *J* = 13.6, 3.6 Hz, 1H), 3.05 - 2.96 (m, 1H), 2.79 (dt, *J* = 14.0, 4.0 Hz, 1H), 2.44 - 2.36 (m, 1H), 2.34 - 2.26 (m, 1H), 2.20 - 2.10 (m, 1H).
^{13}C NMR (100 MHz, $CDCl_3$) δ 209.59, 158.69, 143.81, 140.57, 139.41, 132.75, 130.07, 128.74, 126.77, 126.61, 124.22, 114.35, 113.52, 55.00, 54.90, 41.12, 40.78, 32.45, 28.74.
HRMS (APCI) m/z calcd. for $C_{19}H_{20}N_3O_2$ $[M+H]^+$ 322.1556, found 322.1542.

13-(azidomethyl)-9,11-dimethyl-6,7,8,13-tetrahydro-5H-dibenzo[a,d][9]annulen-5-one

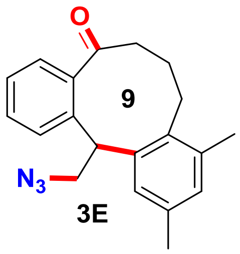

(49%), ^{1}H NMR (400 MHz, $CDCl_3$) δ 7.49 - 7.40 (m, 2H), 7.28 - 7.23 (m, 1H), 7.11 (dd, *J* = 7.6, 1.2 Hz, 1H), 6.82 (s, 1H), 6.44 (s, 1H), 5.00 (t, *J* = 7.6 Hz, 1H), 4.07 (dd, *J* = 12.0, 7.2 Hz, 1H), 3.90 (dd, *J* = 12.0, 8.0 Hz, 1H), 3.08 - 2.95 (m, 3H), 2.47 - 2.39 (m, 1H), 2.32 (s, 3H), 2.28 - 2.22 (m, 1H), 2.14 - 2.08 (m, 4H).
^{13}C NMR (100 MHz, $CDCl_3$) δ 210.04, 143.87, 140.39, 139.19, 136.18, 136.07, 134.00, 130.79, 130.16, 126.74, 126.61, 125.79, 124.38, 54.94, 41.50, 41.42, 27.20, 26.41, 21.00, 20.24.
HRMS (APCI) m/z calcd. for $C_{20}H_{22}N_3O$ $[M+H]^+$ 320.1763, found 320.1757.

13-(azidomethyl)-5-tosyl-6,7-dihydro-5H-dibenzo[b,e]azonin-8(13H)-one

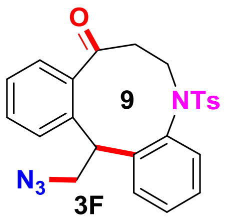

(76%), ^{1}H NMR (400 MHz, $CDCl_3$) δ 7.71 (d, *J* = 8.0 Hz, 2H), 7.61 (d, *J* = 7.6 Hz, 1H), 7.50 (t, *J* = 7.6 Hz, 1H), 7.35 (d, *J* = 8.0 Hz, 2H), 7.26 (t, *J* = 7.6 Hz, 1H), 7.19 (t,

J = 7.6 Hz, 1H), 7.11 (dd, *J* = 14.8, 7.6 Hz, 2H), 6.91 (d, *J* = 7.6 Hz, 1H), 6.71 (d, *J* = 8.0 Hz, 1H), 5.29 (dd, *J* = 9.6, 4.0 Hz, 1H), 4.59 - 4.51 (m, 1H), 4.05 - 3.91 (m, 2H), 3.58 - 3.50 (m, 1H), 3.41 - 3.31 (m, 1H), 2.53 - 2.44 (m, 4H).
^{13}C NMR (100 MHz, $CDCl_3$) δ 207.42, 143.84, 143.55, 143.44, 138.43, 136.91, 136.45, 130.54, 129.97, 129.78, 128.97, 128.83, 128.29, 127.39, 126.92, 126.86, 125.26, 52.26, 50.32, 42.83, 40.00, 21.55.
HRMS (APCI) m/z calcd. for $C_{24}H_{22}N_4O_3SNa$ $[M+Na]^+$ 469.1310, found 469.1288.

13-(azidomethyl)-6,7-dihydrodibenzo[b,e]thionin-8(13H)-one

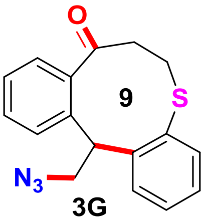

3G

(68%), ^{1}H NMR (400 MHz, $CDCl_3$) δ 7.67 (dd, *J* = 7.6, 1.6 Hz, 1H), 7.53 - 7.45 (m, 2H), 7.30 - 7.24 (m, 2H), 7.20 (td, *J* = 7.6, 1.6 Hz, 1H), 7.09 - 7.06 (m, 1H), 7.02 (dd, *J* = 7.6, 1.6 Hz, 1H), 5.83 (t, *J* = 7.6 Hz, 1H), 3.98 (dd, *J* = 12.4, 7.6 Hz, 1H), 3.86 (dd, *J* = 12.4, 7.6 Hz, 1H), 3.22 - 3.06 (m, 3H), 2.75 - 2.67 (m, 1H).
^{13}C NMR (100 MHz, $CDCl_3$) δ 208.11, 146.91, 143.38, 138.18, 137.32, 132.58, 130.46, 130.20, 128.43, 127.95, 127.00, 125.95, 125.25, 54.46, 43.46, 42.82, 33.19.
HRMS (APCI) m/z calcd. for $C_{17}H_{16}N_3OS$ $[M+H]^+$ 310.1014, found 310.1009.

14-(azidomethyl)-6,7,8,9-tetrahydrodibenzo[a,d][10]annulen-5(14H)-one

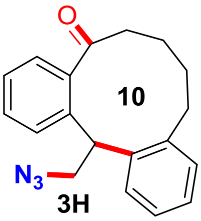

3H

(74%), ^{1}H NMR (400 MHz, $CDCl_3$) δ 7.64 (d, *J* = 8.0 Hz, 1H), 7.44 - 7.38 (m, 2H), 7.25 - 7.12 (m, 5H), 5.01 (t, *J* = 8.0 Hz, 1H), 4.07 - 3.92 (m, 2H), 3.22 - 3.13 (m, 1H), 3.06 - 2.95 (m, 1H), 2.69 (dt, *J* = 14.4, 4.0 Hz, 1H), 2.59 - 2.51 (m, 1H), 2.06 - 1.95 (m, 1H), 1.91 - 1.81 (m, 1H), 1.67 - 1.57 (m, 1H), 1.27 - 1.19 (m, 1H).
^{13}C NMR (100 MHz, $CDCl_3$) δ 211.70, 141.64, 140.09, 140.01, 139.19, 130.40, 130.23, 126.93, 126.52, 126.38, 126.31, 125.81, 125.69, 54.84, 45.31, 39.36, 32.75, 28.73, 21.27.
HRMS (APCI) m/z calcd. for $C_{19}H_{20}N_3O$ $[M+H]^+$ 306.1606, found 306.1601.

14-(azidomethyl)-12-fluoro-6,7,8,9-tetrahydrodibenzo[a,d][10]annulen-5(14H)-one

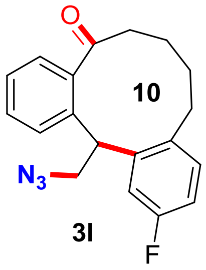

3l

(82%), ^{1}H NMR (400 MHz, $CDCl_3$) δ 7.59 (d, *J* = 8.0 Hz, 1H), 7.47 - 7.41 (m, 1H), 7.27 - 7.19 (m, 2H), 7.15 - 7.08 (m, 2H), 6.85 (td, *J* = 8.4, 2.8 Hz, 1H), 5.03 - 4.96 (m, 1H), 4.04 - 3.90 (m, 2H), 3.19 (ddd, *J* = 13.6, 9.2, 2.0 Hz, 1H), 3.01 - 2.91 (m, 1H), 2.67 (dt, *J* = 14.8, 4.0 Hz, 1H), 2.59 - 2.50 (m, 1H), 2.04 - 1.94 (m, 1H), 1.87 - 1.77 (m, 1H), 1.67 - 1.57 (m, 1H), 1.25 - 1.12 (m, 1H).

^{13}C NMR (100 MHz, $CDCl_3$) δ 211.56, 162.20 (d, *J* = 243.1 Hz), 142.17 (d, *J* = 6.1 Hz), 141.57, 138.51, 135.55 (d, *J* = 3.1 Hz), 131.71 (d, *J* = 7.9 Hz), 130.58, 126.64, 126.16, 125.78, 114.26 (d, *J* = 20.9 Hz), 112.32 (d, *J* = 21.2 Hz), 54.57, 45.27, 39.42, 32.75, 28.17, 21.11.

^{19}F NMR (376 MHz, $CDCl_3$) δ -115.55.

HRMS (APCI) m/z calcd. for $C_{19}H_{19}FN_3O$ $[M+H]^+$ 324.1512, found 324.1585.

15-(azidomethyl)-6,7,8,9-tetrahydrodibenzo[b,e][1]oxacycloundecin-10(15H)-one

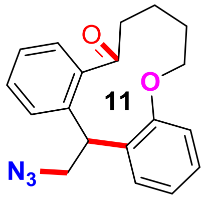

3J, 75%

(75%), ^{1}H NMR (400 MHz, $CDCl_3$) δ 7.39 -7.32 (m, 4H), 7.26 – 7.14 (m, 2H), 7.00 (t, *J* = 7.3 Hz, 1H), 6.70 (d, *J* = 8.0 Hz, 1H), 5.31 (t, *J* = 8.1 Hz, 1H), 4.12 – 3.99 (m, 3H), 3.86 – 3.72 (m, 1H), 3.23 (ddd, *J* = 16.1, 10.0, 2.7 Hz, 1H), 2.81 (ddd, *J* = 16.1, 8.5, 3.0 Hz, 1H), 2.34 – 2.16 (m, 2H), 2.08 – 1.92 (m, 1H), 1.75 – 1.62 (m, 1H).

^{13}C NMR (101 MHz, $CDCl_3$) δ 206.08, 156.23, 140.53, 139.51, 130.70, 130.29, 128.27, 127.85, 126.40, 126.06, 124.95, 120.65, 110.89, 68.26, 53.32, 42.47, 36.95, 26.80, 21.84.

18-(azidomethyl)-7,8,9,10,11,12-hexahydro-6H-dibenzo[b,e][1]oxacyclotetradecin-13(18H)-one

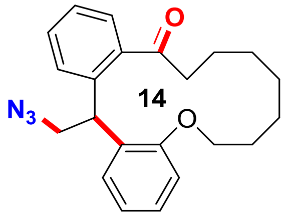

3K, 65%

(65% yield), ^{1}H NMR (500 MHz, $CDCl_3$) δ 7.64 (dd, *J* = 7.5, 1.5 Hz, 1H), 7.50 (dd, *J*

= 7.5, 1.5 Hz, 1H), 7.34 - 7.24 (m, 3H), 7.17 (dd, J = 7.5, 1.5 Hz, 1H), 7.05 (td, J = 7.5, 1.0 Hz, 1H), 6.83 (d, J = 8.0 Hz, 1H), 5.19 (dd, J = 10.0, 4.0 Hz, 1H), 4.11 (dd, J = 12.5, 4.0 Hz, 1H), 3.96 - 3.91 (m, 1H), 3.86 (td, J = 9.0, 2.5 Hz, 1H), 3.71 (dd, J = 12.5, 10.0 Hz, 1H), 3.27 - 3.20 (m, 1H), 2.87 - 2.80 (m, 1H), 2.09 - 2.00 (m, 1H), 1.80 - 1.68 (m, 2H), 1.65 - 1.54 (m, 2H), 1.50 - 1.30 (m, 5H).
^{13}C NMR (125 MHz, $CDCl_3$) δ 205.38, 157.12, 140.57, 138.28, 131.02, 129.46, 128.70, 128.48, 128.33, 128.07, 126.43, 119.88, 111.52, 68.67, 54.50, 41.25, 39.55, 28.27, 27.51, 26.66, 26.07, 23.19.
HRMS (ESI) m/z calcd. for $C_{22}H_{26}N_3O_2$ $[M+H]^+$ 364.2025, found 364.2016.

18-(azidomethyl)-7,10,11,12-tetrahydro-6H-dibenzo[b,e][1]oxacyclotetradecin-13(18H)-one

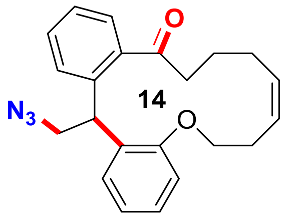

3L, 55%

(55% yield), ^{1}H NMR (500 MHz, $CDCl_3$) δ 7.54 - 7.50 (m, 2H), 7.30 - 7.27 (m, 1H), 7.25 -7.21 (m, 2H), 7.17 (dd, J = 8.0, 1.5 Hz, 1H), 7.06 (t, J = 7.5 Hz, 1H), 6.84 (d, J = 8.0 Hz, 1H), 5.46 - 5.34 (m, 2H), 5.16 (dd, J = 10.5, 4.0 Hz, 1H), 4.14 - 4.07 (m, 1H), 4.04 (dd, J = 12.5, 4.5 Hz, 1H), 3.86 (td, J = 8.5, 2.5 Hz, 1H), 3.66 (dd, J = 12.5, 10.5 Hz, 1H), 3.16 - 3.04 (m, 2H), 2.57 - 2.37 (m, 3H), 2.25 - 2.12 (m, 2H), 1.77 - 1.68 (m, 1H).
^{13}C NMR (100 MHz, $CDCl_3$) δ 205.01, 157.52, 139.91, 139.22, 131.02, 130.63, 129.22, 128.65, 128.51, 128.09, 128.07, 127.73, 126.49, 120.31, 112.48, 68.82, 54.71, 40.16, 38.64, 28.17, 25.79, 21.81.
HRMS (ESI) m/z calcd. for $C_{22}H_{24}N_3O_2$ $[M+H]^+$ 362.1869, found 362.1854.

13-(1-azidoethyl)-11-bromo-6,7,8,13-tetrahydro-5H-dibenzo[a,d][9]annulen-5-one

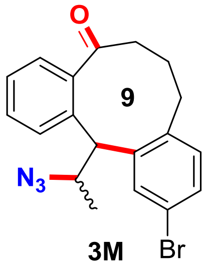

3M

(55% yield, dr = 1.5:1) ^{1}H NMR (400 MHz, $CDCl_3$) δ 7.64 (d, J = 7.9 Hz, 0.60H), 7.53 – 7.44 (m, 1.40H), 7.36 (d, J = 2.1 Hz, 0.40H), 7.29 – 7.24 (m, 2H), 7.12 – 7.02 (m, 2H), 6.98 (d, J = 2.1 Hz, 0.60H), 4.58 (d, J = 10.5 Hz, 0.60H), 4.37 – 4.19 (m, 1.40H), 3.17 – 3.03 (m, 1H), 2.90 – 2.74 (m, 1.60H), 2.61 – 2.54 (m, 0.40H), 2.39 – 2.36 (m, 2H), 2.04 – 1.96 (m, 0.60H), 1.85 – 1.78 (m, 0.40H), 1.37 (d, J = 5.8 Hz,

1.20H), 1.18 (d, J = 6.4 Hz, 1.80H).
^{13}C NMR (101 MHz, $CDCl_3$) δ 211.18, 210.19, 144.59, 144.41, 142.28, 141.84, 138.61, 137.94, 137.45, 136.81, 131.82, 131.47, 131.15, 130.82, 130.61, 130.39, 130.32, 130.21, 130.00, 127.06, 127.03, 126.04, 125.59, 124.67, 121.14, 120.73, 60.41, 58.92, 47.61, 47.04, 41.23, 40.82, 31.27, 30.43, 28.11, 27.50, 18.65, 17.54.
HRMS (ESI) m/z calcd. for $C_{19}H_{19}BrN_3O$ $[M+H]^+$ 384.0711, found 384.0713.

General procedure for trifluoromethylation reaction of 1 (method a)

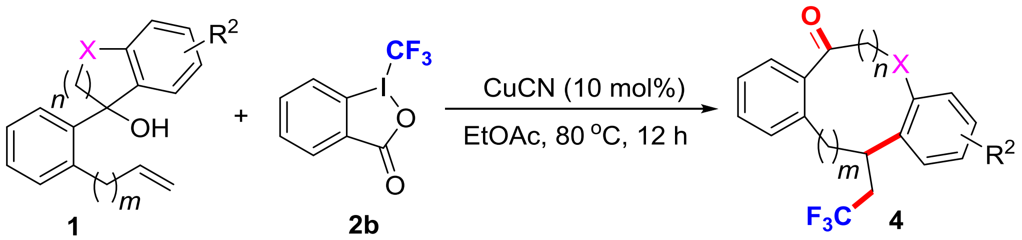

Under argon, a 25 mL Schlenk tube equipped with a magnetic stir bar was charged with **1** (0.2 mmol, 1.0 equiv), **2b** (126 mg, 0.4 mmol, 2.0 equiv), CuCN (1.8 mg, 0.02 mmol, 0.1 equiv) and EtOAc (2.0 mL). The sealed tube was then stirred at 80 °C for 12 h. After completion (monitored by TLC), EtOAc (30 mL) was added and the reaction mixture was washed with saturated $NaHCO_3$ (2 × 5 mL) solution. The organic layer was dried over anhydrous Na_2SO_4, filtered and concentrated to afford the crude product, which was purified by flash column chromatography to afford the product **4**.

11-bromo-13-(2,2,2-trifluoroethyl)-6,7,8,13-tetrahydro-5H-dibenzo[a,d][9]annulen-5-one

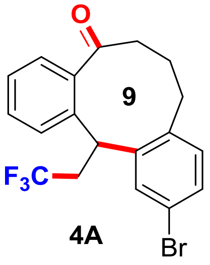

4A

(64% yield), ^{1}H NMR (400 MHz, $CDCl_3$) δ 7.47 (t, J = 7.6 Hz, 1H), 7.40 (d, J = 8.0 Hz, 1H), 7.28 (t, J = 8.0 Hz, 1H), 7.22 (dd, J = 8.4, 2.0 Hz, 1H), 7.11 (d, J = 7.6 Hz, 1H), 7.00 - 6.94 (m, 2H), 5.04 (dd, J = 10.4, 3.6 Hz, 1H), 3.13 - 2.99 (m, 2H), 2.95 - 2.75 (m, 3H), 2.44 - 2.30 (m, 2H), 2.12 - 2.03 (m, 1H).
^{13}C NMR (100 MHz, $CDCl_3$) δ 209.73, 143.66, 143.37, 138.62, 137.59, 131.66, 130.76, 130.43, 130.24, 127.27, 126.61, 126.31 (q, J = 275.9 Hz), 124.01, 120.83, 40.91, 38.51 (q, J = 27.5 Hz), 35.40 (q, J = 2.4 Hz), 31.15, 28.28.
^{19}F NMR (376 MHz, $CDCl_3$) δ -63.38.
HRMS (APCI) m/z calcd. for $C_{19}H_{17}BrF_3O$ $[M+H]^+$ 397.0415, found 397.0409

11-fluoro-13-(2,2,2-trifluoroethyl)-6,7,8,13-tetrahydro-5H-dibenzo[a,d][9]annule

n-5-one

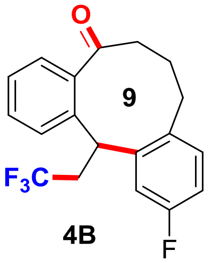

4B

(61% yield), ^{1}H NMR (400 MHz, $CDCl_3$) δ 7.48 (t, J = 7.2 Hz, 1H), 7.40 (d, J = 7.6 Hz, 1H), 7.28 (t, J = 7.6 Hz, 1H), 7.14 - 7.05 (m, 2H), 6.82 (td, J = 8.4, 2.8 Hz, 1H), 6.49 (dd, J = 10.0, 2.4 Hz, 1H), 5.09 (d, J = 7.6 Hz, 1H), 3.15 - 3.02 (m, 2H), 3.00 - 2.92 (m, 1H), 2.88 - 2.77 (m, 2H), 2.46 - 2.38 (m, 1H), 2.36 - 2.28 (m, 1H), 2.16 - 2.04 (m, 1H).

^{13}C NMR (100 MHz, $CDCl_3$) δ 209.72, 161.77 (d, J = 243.9 Hz), 143.77, 143.31 (d, J = 6.3 Hz), 139.04, 134.41, 131.66 (d, J = 7.9 Hz), 130.47, 127.31, 126.90, 126.43 (q, J = 275.9 Hz), 123.94, 115.13 (d, J = 21.0 Hz), 113.83 (d, J = 21.3 Hz), 41.04, 38.47 (q, J = 27.6 Hz), 35.76 (q, J = 2.0 Hz), 31.11, 28.81.

^{19}F NMR (376 MHz, $CDCl_3$) δ -63.36 (s, 3F), -114.50 (s, 1F).

HRMS (APCI) m/z calcd. for $C_{19}H_{17}F_4O$ $[M+H]^+$ 337.1216, found 337.1210.

13-(2,2,2-trifluoroethyl)-6,7,8,13-tetrahydro-5H-dibenzo[a,d][9]annulen-5-one

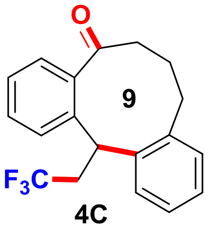

4C

(54% yield), ^{1}H NMR (400 MHz, $CDCl_3$) δ 7.49 - 7.41 (m, 2H), 7.26 (td, J = 7.6, 1.2 Hz, 1H), 7.14 - 7.05 (m, 4H), 6.82 (d, J = 7.6 Hz, 1H), 5.11 (dd, J = 10.0, 4.0 Hz, 1H), 3.20 - 3.05 (m, 2H), 3.01 - 2.80 (m, 3H), 2.45 - 2.30 (m, 2H), 2.18 - 2.07 (m, 1H).

^{13}C NMR (100 MHz, $CDCl_3$) δ 209.85, 143.84, 141.36, 139.82, 138.60, 130.14, 129.90, 127.51, 127.42, 127.40, 126.93, 126.63, 126.53 (q, J = 276.0 Hz), 123.98, 41.06, 38.55 (q, J = 27.4 Hz), 35.46 (q, J = 2.5 Hz), 31.65, 28.77.

^{19}F NMR (376 MHz, $CDCl_3$) δ -63.34.

HRMS (APCI) m/z calcd. for $C_{19}H_{18}F_3O$ $[M+H]^+$ 319.1310, found 319.1304.

10-methoxy-13-(2,2,2-trifluoroethyl)-6,7,8,13-tetrahydro-5H-dibenzo[a,d][9]annulen-5-one

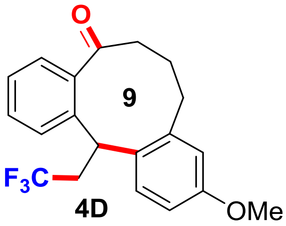

4D

(49% yield), ^{1}H NMR (400 MHz, $CDCl_3$) δ 7.47 - 7.38 (m, 2H), 7.27 - 7.22 (m, 1H), 7.09 (dd, J = 7.6, 1.2 Hz, 1H), 6.71 (d, J = 8.4 Hz, 1H), 6.65 - 6.59 (m, 2H), 5.04 (dd, J = 10.4, 4.0 Hz, 1H), 3.73 (s, 3H), 3.17 - 2.92 (m, 3H), 2.86 - 2.73 (m, 2H), 2.45 - 2.27 (m, 2H), 2.18 - 2.07 (m, 1H).
^{13}C NMR (100 MHz, $CDCl_3$) δ 209.74, 158.44, 143.74, 140.38, 139.98, 133.78, 130.09, 128.65, 126.83, 126.62, 126.56 (q, J = 275.9 Hz), 123.83, 114.17, 113.65, 55.00, 41.06, 38.56 (q, J = 27.1 Hz), 34.97 (q, J = 2.5 Hz), 32.01, 28.70.
^{19}F NMR (376 MHz, $CDCl_3$) δ -63.28 (s).
HRMS (APCI) m/z calcd. for $C_{20}H_{20}F_3O_2$ $[M+H]^+$ 349.1415, found 349.1410.

5-tosyl-13-(2,2,2-trifluoroethyl)-6,7-dihydro-5H-dibenzo[b,e]azonin-8(13H)-one

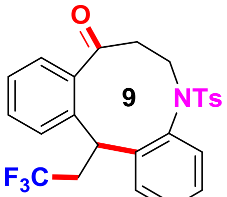

4F

(35% yield), ^{1}H NMR (500 MHz, $CDCl_3$) δ 7.68 (d, J = 8.5 Hz, 2H), 7.55 (d, J = 8.0 Hz, 1H), 7.44 (td, J = 7.5, 1.0 Hz, 1H), 7.33 (d, J = 8.0 Hz, 2H), 7.27 - 7.21 (m, 2H), 7.12 (td, J = 8.0, 1.5 Hz, 1H), 7.08 - 7.04 (m, 2H), 6.81 (d, J = 8.0 Hz, 1H), 5.27 (dd, J = 11.5, 2.5 Hz, 1H), 4.59 - 4.53 (m, 1H), 3.43 - 3.36 (m, 1H), 3.26 - 3.16 (m, 2H), 2.67 - 2.55 (m, 2H), 2.45 (s, 3H).
^{13}C NMR (125 MHz, $CDCl_3$) δ 207.60, 144.81, 143.97, 143.07, 137.75, 136.25, 136.06, 130.08, 129.72, 128.42, 128.31, 127.92, 127.74, 126.89, 125.93 (q, J = 276.4 Hz), 125.71, 125.23, 125.21, 49.83, 42.59, 36.49 (q, J = 28.5 Hz), 34.43 (q, J = 2.6 Hz), 21.55.
^{19}F NMR (376 MHz, $CDCl_3$) δ -62.85.
HRMS (APCI) m/z calcd. for $C_{25}H_{23}F_3NO_3S$ $[M+H]^+$ 474.1351, found 474.1345.

13-(2,2,2-trifluoroethyl)-6,7-dihydrodibenzo[b,e]thionin-8(13H)-one

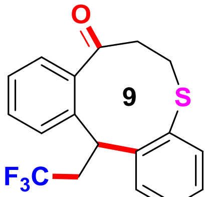

4G

(54% yield), ^{1}H NMR (500 MHz, $CDCl_3$) δ 7.65 (d, J = 7.5 Hz, 1H), 7.52 - 7.45 (m, 2H), 7.32 - 7.25 (m, 2H), 7.18 (t, J = 7.5 Hz, 1H), 7.12 (d, J = 8.0 Hz, 1H), 7.05 (d, J = 7.5 Hz, 1H), 5.89 (dd, J = 9.0, 5.0 Hz, 1H), 3.20 - 3.08 (m, 2H), 3.05 - 2.98 (m, 2H), 2.87 - 2.78 (m, 1H), 2.74 - 2.67 (m, 1H).
^{13}C NMR (125 MHz, $CDCl_3$) δ 208.43, 148.12, 143.14, 139.06, 137.49, 132.08, 130.46, 130.13, 128.08, 127.65, 126.99, 126.13 (q, J = 276.3 Hz), 125.48, 125.10, 43.14, 39.02 (q, J = 27.8 Hz), 37.02 (q, J = 2.4 Hz), 32.84.
^{19}F NMR (376 MHz, $CDCl_3$) δ -63.25.

HRMS (APCI) m/z calcd. for $C_{18}H_{16}F_3OS$ $[M+H]^+$ 337.0874, found 337.0868.

14-(2,2,2-trifluoroethyl)-6,7,8,9-tetrahydrodibenzo[a,d][10]annulen-5(14H)-one

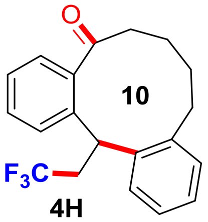

4H

(67% yield), ^{1}H NMR (400 MHz, $CDCl_3$) δ 7.66 (d, *J* = 8.0 Hz, 1H), 7.45 - 7.40 (m, 2H), 7.23 - 7.09 (m, 5H), 5.20 (t, *J* = 7.2 Hz, 1H), 3.25 - 3.17 (m, 1H), 3.06 - 2.94 (m, 3H), 2.68 (dt, *J* = 14.4, 4.0 Hz, 1H), 2.60 - 2.52 (m, 1H), 2.06 - 1.89 (m, 2H), 1.67 - 1.57 (m, 1H), 1.25 - 1.13 (m, 1H).

^{13}C NMR (100 MHz, $CDCl_3$) δ 211.68, 141.55, 140.92, 140.90, 139.13, 130.50, 129.96, 126.63, 126.52, 126.49, 126.27 (q, *J* = 276.3 Hz), 126.19, 126.13, 125.59, 45.55, 39.57 (q, *J* = 27.4 Hz), 33.30 (q, *J* = 2.9 Hz), 32.88, 28.37, 21.14.

^{19}F NMR (376 MHz, $CDCl_3$) δ -64.23.

HRMS (APCI) m/z calcd. for $C_{20}H_{20}$F3O $[M+H]^+$ 333.1466, found 333.1460.

12-fluoro-14-(2,2,2-trifluoroethyl)-6,7,8,9-tetrahydrodibenzo[a,d][10]annulen-5(14H)-one

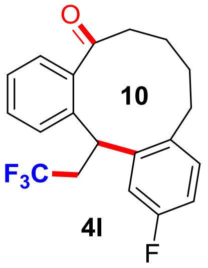

4I

(75% yield), ^{1}H NMR (400 MHz, $CDCl_3$) δ 7.61 (d, *J* = 8.0 Hz, 1H), 7.47 - 7.41 (m, 1H), 7.25 - 7.18 (m, 2H), 7.13 – 7.07 (m, 2H), 6.83 (td, *J* = 8.4, 2.8 Hz, 1H), 5.19 (t, *J* = 7.2 Hz, 1H), 3.26 - 3.18 (m, 1H), 3.01 - 2.90 (m, 3H), 2.65 (dt, *J* = 14.8, 4.0 Hz, 1H), 2.59 - 2.51 (m, 1H), 2.05 - 1.95 (m, 1H), 1.93 - 1.82 (m, 1H), 1.68 - 1.56 (m, 1H), 1.22 - 1.10 (m, 1H).

^{13}C NMR (100 MHz, $CDCl_3$) δ 211.55, 161.19 (d, *J* = 242.9 Hz), 143.52 (d, *J* = 6.1 Hz), 140.88, 140.23, 134.67 (d, *J* = 3.1 Hz), 131.48 (d, *J* = 8.0 Hz), 130.71, 126.47, 126.32, 126.10 (q, *J* = 276.2 Hz), 125.68, 114.07 (d, *J* = 20.9 Hz), 112.55 (d, *J* = 21.3 Hz), 45.50, 39.38 (q, *J* = 27.7 Hz), 33.44, 32.87, 27.80, 20.96.

^{19}F NMR (376 MHz, $CDCl_3$) δ -64.25 (s, 3F), -115.68 (s, 1F).

HRMS (APCI) m/z calcd. for $C_{20}H_{19}F_4O$ $[M+H]^+$ 351.1372, found 351.1353.

5-(2,2,2-trifluoroethyl)-8,10,11,12-tetrahydro-5H-spiro[benzo[10]annulene-7,1'-cyclohexan]-9(6H)-one

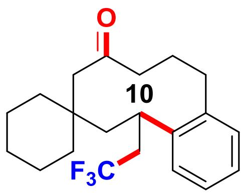

4V

(65% yield), ^{1}H NMR (400 MHz, $CDCl_3$) δ 7.22 (td, J = 7.6, 1.2 Hz, 1H), 7.14 (td, J = 7.2, 1.2 Hz, 1H), 7.11 (d, J = 7.6 Hz, 1H), 7.07 (dd, J = 7.6, 1.2 Hz, 1H), 3.06 - 2.99 (m, 1H), 2.91 (td, J = 14.0, 3.6 Hz, 1H), 2.63 (d, J = 11.2 Hz, 1H), 2.60 - 2.46 (m, 2H), 2.42 - 2.26 (m, 3H), 2.17 - 2.02 (m, 2H), 1.98 (d, J = 11.2 Hz, 1H), 1.84 (d, J = 4.0 Hz, 2H), 1.72 - 1.59 (m, 4H), 1.53 - 1.46 (m, 1H), 1.38 - 1.24 (m, 5H).

^{13}C NMR (100 MHz, $CDCl_3$) δ 212.82, 145.77, 138.49, 129.48, 126.70, 126.49, 126.27, 126.01 (q, J = 276.4 Hz), 50.78, 50.01, 44.80 (q, J = 26.3 Hz), 42.59, 41.53, 40.04, 31.27, 28.87 (q, J = 2.1 Hz), 28.10, 26.15, 24.36, 21.80, 21.71.

^{19}F NMR (376 MHz, $CDCl_3$) δ -64.23.

HRMS (APCI) m/z calcd. for $C_{21}H_{28}F_3O$ $[M+H]^+$ 353.2092, found 353.2073.

Procedure for synthesis of 4N and 4W

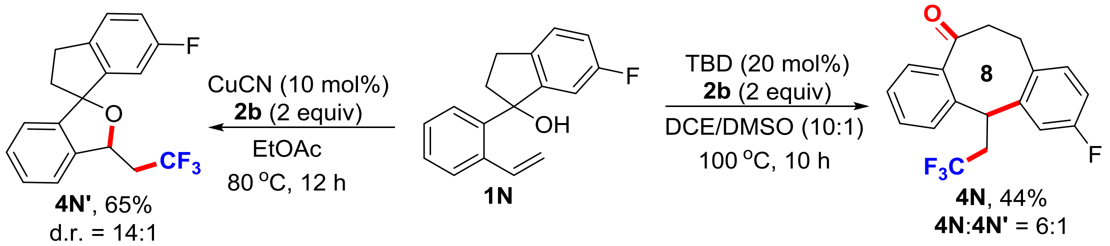

Under argon, a 25 mL Schlenk tube equipped with a magnetic stir bar was charged with **1N** (51 mg, 0.2 mmol, 1.0 equiv), **2b** (126 mg, 0.4 mmol, 2.0 equiv), TBD (5 mg, 0.04 mmol, 0.2 equiv) DCE (1.8 mL) and DMSO (0.18 mL). The sealed tube was then stirred at 100 °C for 10 h. After completion (monitored by TLC), EtOAc (40 mL) was added and the reaction mixture was washed with saturated $NaHCO_3$ solution (2 × 5 mL). The organic layer was dried over anhydrous Na_2SO_4, filtered and concentrated to afford the crude product, which was purified by flash column chromatography to afford the product **4N** (29 mg, 44%).

To synthesize **4N'**, CuCN was used as the catalyst and reaction proceeded in EtOAc at 80 °C for 12 h. To synthesized **4W**, DMSO was utilized as the reaction solvent, and the reaction was heated up to 100 °C for 6 h.

6-fluoro-3'-(2,2,2-trifluoroethyl)-2,3-dihydro-3'H-spiro[indene-1,1'-isobenzofura n

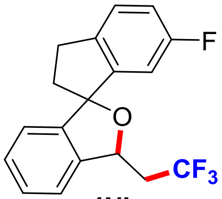

4N'

^{1}H NMR (400 MHz, $CDCl_3$) δ 7.39 - 7.23 (m, 4H), 7.01 - 6.93 (m, 2H), 6.58 (dd, *J* = 8.4, 2.4 Hz, 1H), 5.58 - 5.53 (m, 1H), 3.21 - 3.11 (m, 1H), 3.02 - 2.93 (m, 1H), 2.76 - 2.67 (m, 1H), 2.65 - 2.53 (m, 2H), 2.42 - 2.33 (m, 1H).
^{13}C NMR (100 MHz, $CDCl_3$) δ 162.36 (d, *J* = 242.6 Hz), 147.61 (d, *J* = 7.0 Hz), 144.24, 140.07, 139.27 (d, *J* = 2.4 Hz), 128.81, 128.27, 125.89 (d, *J* = 8.4 Hz), 125.84 (q, *J* = 275.7 Hz), 121.71, 116.05 (d, *J* = 22.6 Hz), 111.06 (d, *J* = 21.9 Hz), 96.96, 76.11 (q, *J* = 3.2 Hz), 41.85 (q, *J* = 27.2 Hz), 41.24, 29.30.
^{19}F NMR (376 MHz, $CDCl_3$) δ -63.23 (s, 3 F), -116.06 (s, 1 F).
HRMS (APCI) m/z calcd. for $C_{18}H_{15}F_4O$ $[M+H]^+$ 323.1059, found 323.1054.

10-fluoro-12-(2,2,2-trifluoroethyl)-6,7-dihydrodibenzo[a,d][8]annulen-5(12H)-one

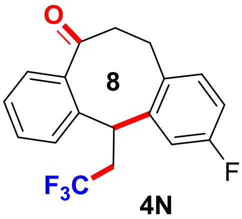

4N

(44% yield), ^{1}H NMR (500 MHz, $CDCl_3$) δ 7.40 (t, *J* = 7.5 Hz, 1H), 7.32 (d, *J* = 7.5 Hz, 1H), 7.24 (t, *J* = 7.5 Hz, 1H), 7.17 - 7.11 (m, 2H), 6.95 (dd, *J* = 10.0, 2.5 Hz, 1H), 6.82 (td, *J* = 8.0, 2.5 Hz, 1H), 4.63 (dd, *J* = 9.0, 6.0 Hz, 1H), 3.43 - 3.36 (m, 1H), 3.25 - 3.19 (m, 1H), 3.16 - 2.96 (m, 3H), 2.85 - 2.78 (m, 1H).
^{13}C NMR (125 MHz, $CDCl_3$) δ 209.15, 162.11 (d, *J* = 244.4 Hz), 143.59 (d, *J* = 6.6 Hz), 142.98, 136.75, 133.40 (d, *J* = 3.1 Hz), 132.10 (d, *J* = 8.1 Hz), 130.67, 127.50, 126.25, 126.08 (q, *J* = 276.0 Hz),124.08, 114.07 (d, *J* = 20.8 Hz), 111.82 (d, *J* = 22.1 Hz), 45.64, 38.08, 36.38 (q, *J* = 28.0 Hz), 31.05.
^{19}F NMR (376 MHz, $CDCl_3$) δ -63.96 (s, 3F), -114.12 (s, 1F).
HRMS (APCI) m/z calcd. for $C_{18}H_{15}F_4O$ $[M+H]^+$ 323.1059, found 323.1054.

3-fluoro-5-(2,2,2-trifluoroethyl)-5,6,12,13-tetrahydro-11H-dibenzo[a,e][9]annulen-11-one

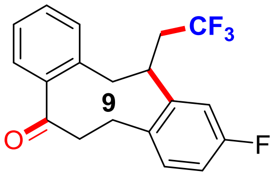

4W, 40%

(40% yield) ^{1}H NMR (400 MHz, $CDCl_3$) δ 7.18 – 6.97 (m, 4H), 6.92 (d, *J* = 7.4 Hz,

1H), 6.74 – 6.57 (m, 2H), 3.96 – 3.81 (m, 1H), 3.73 (dd, *J* = 14.5, 6.9 Hz, 1H), 3.40 – 3.28 (m, 1H), 3.24 – 3.14 (m, 1H), 2.98 – 2.87 (m, 2H), 2.67 – 2.45 (m, 3H).
^{13}C NMR (101 MHz, $CDCl_3$) δ 212.28, 161.74 (d, *J* = 244.0 Hz), 142.47 (d, *J* = 6.2 Hz), 142.24, 134.33 (d, *J* = 78.7 Hz), 133.90, 131.66, 131.36 (d, *J* = 8.2 Hz), 129.69, 126.17 (q, *J* = 276.0 Hz), 126.05, 125.49, 113.90 (d, *J* = 20.9 Hz), 112.11 (d, *J* = 21.2 Hz), 46.06, 41.50, 40.71 (q, *J* = 27.6 Hz), 31.57, 31.44. ^{19}F NMR (376 MHz, $CDCl_3$) δ -63.96, -115.13.
HRMS (ESI) m/z calcd. for $C_{19}H_{17}F_4O$ $[M+H]^+$ 337.1210, found 337.1210.

General procedure for trifluoromethylation reaction (method b)

Under argon, a 25 mL Schlenk tube equipped with a magnetic stir bar was charged with **1** (0.2 mmol, 1.0 equiv), **2b** (126 mg, 0.4 mmol, 2.0 equiv), CuCN (1.8 mg, 0.02 mmol, 0.1 equiv) and dioxane (4.0 mL). The sealed tube was then stirred at 60 °C for 24 h. After completion (monitored by TLC), EtOAc (30 mL) was added and the reaction mixture was washed with saturated $NaHCO_3$ solution (2 × 5 mL). The organic layer was dried over anhydrous Na_2SO_4, filtered and concentrated to afford the crude product, which was purified by flash column chromatography to afford the product **4**.

13-(2,2,2-trifluoroethyl)-7,8,13,14-tetrahydrodibenzo[a,e][10]annulen-5(6H)-one

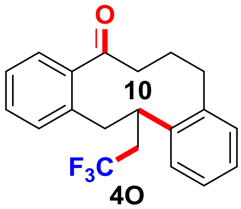

4O

(60% yield), ^{1}H NMR (400 MHz, $CDCl_3$) δ 7.45 (d, *J* = 7.2 Hz, 1H), 7.31 (dd, *J* = 7.2, 1.2 Hz, 1H), 7.28 (d, *J* = 7.2 Hz, 1H), 7.23 - 7.19 (m, 1H), 7.17 - 7.10 (m, 4H), 3.37 - 3.27 (m, 2H), 3.24 - 3.10 (m, 2H), 2.94 - 2.84 (m, 1H), 2.75 - 2.64 (m, 2H), 2.55 - 2.30 (m, 3H), 1.73 - 1.62 (m, 1H).
^{13}C NMR (100 MHz, $CDCl_3$) δ 206.90, 141.87, 140.32, 139.41, 137.61, 131.16, 130.86, 130.69, 129.21, 127.22, 126.91, 125.99 (q, *J* = 275.9 Hz), 125.40, 44.22, 39.75 (q, *J* = 27.6 Hz), 37.99, 36.24, 29.13, 26.72.
^{19}F NMR (376 MHz, $CDCl_3$) δ -64.12.
HRMS (APCI) m/z calcd. for $C_{20}H_{20}F_3O$ $[M+H]^+$ 333.1466, found 333.1461.

11-fluoro-13-(2,2,2-trifluoroethyl)-7,8,13,14-tetrahydrodibenzo[a,e][10]annulen-5(6H)-one

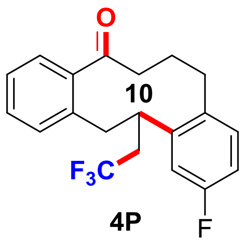

4P

(69% yield), ^{1}H NMR (400 MHz, $CDCl_3$) δ 7.43 (dd, *J* = 7.6, 1.6 Hz, 1H), 7.33 (td, *J* = 7.6, 1.6 Hz, 1H), 7.30 - 7.24 (m, 1H), 7.14 - 7.09 (m, 2H), 6.88 - 6.80 (m, 2H), 3.37 - 3.30 (m, 1H), 3.27 - 3.21 (m, 1H), 3.21 - 3.12 (m, 2H), 2.86 - 2.79 (m, 1H), 2.73 - 2.62 (m, 2H), 2.47 - 2.34 (m, 3H), 1.67 - 1.59 (m, 1H).
^{13}C NMR (100 MHz, $CDCl_3$) δ 206.88, 161.73 (d, *J* = 243.2 Hz), 143.68 (d, *J* = 6.5 Hz), 140.17, 137.10, 135.25 (d, *J* = 3.0 Hz), 132.20 (d, *J* = 7.9 Hz), 131.27, 130.95, 129.05, 127.29, 125.85 (q, *J* = 275.9 Hz), 114.14 (d, *J* = 20.8 Hz), 111.94 (d, *J* = 20.8 Hz), 43.81, 39.76 (q, *J* = 27.5 Hz), 38.29, 36.18, 28.46, 26.73.
^{19}F NMR (376 MHz, $CDCl_3$) δ -64.17 (s, 3F), -115.70 (s, 1F).
HRMS (APCI) m/z calcd. for $C_{20}H_{19}F_4O$ $[M+H]^+$ 351.1372, found 351.1367.

11-bromo-13-(2,2,2-trifluoroethyl)-7,8,13,14-tetrahydrodibenzo[a,e][10]annulen-5(6H)-one

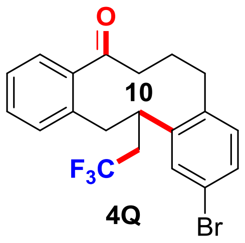

4Q

(70% yield), ^{1}H NMR (400 MHz, $CDCl_3$) δ 7.44 (dd, *J* = 7.6, 1.2 Hz, 1H), 7.34 (td, *J* = 7.6, 1.6 Hz, 1H), 7.30 - 7.25 (m, 3H), 7.12 (d, *J* = 7.2 Hz, 1H), 7.04 - 7.01 (m, 1H), 3.33 - 3.25 (m, 2H), 3.22 - 3.11 (m, 2H), 2.86 - 2.77 (m, 1H), 2.72 - 2.63 (m, 2H), 2.48 - 2.34 (m, 3H), 1.68 - 1.60 (m, 1H).
^{13}C NMR (125 MHz, $CDCl_3$) δ 206.69, 144.15, 140.13, 138.60, 137.13, 132.43, 131.21, 131.04, 130.12, 129.24, 128.55, 127.40, 125.82 (q, *J* = 277.3 Hz), 120.53, 43.97, 39.70 (q, *J* = 27.8 Hz), 38.10, 36.21, 28.76, 26.58.
^{19}F NMR (376 MHz, $CDCl_3$) δ -64.19.
HRMS (APCI) m/z calcd. for $C_{20}H_{19}BrF_3O$ $[M+H]^+$ 411.0571, found 411.0565.

2-methyl-14-(2,2,2-trifluoroethyl)-13,14-dihydro-6H-dibenzo[b,f]oxecin-8(7H)-one

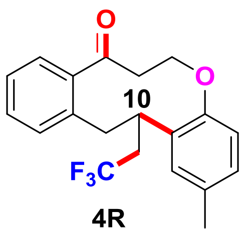

4R

(51% yield), ^{1}H NMR (400 MHz, $CDCl_3$) δ 7.15 - 7.03 (m, 3H), 7.00 (d, *J* = 7.2 Hz,

1H), 6.78 (dd, J = 8.0, 1.6 Hz, 1H), 6.71 (s, 1H), 6.60 (d, J = 8.4 Hz, 1H), 4.54 - 4.48 (m, 1H), 4.30 - 4.20 (m, 1H), 3.75 - 3.63 (m, 1H), 3.50 - 3.41 (m, 1H), 3.28 - 3.18 (m, 1H), 3.08 - 2.92 (m, 2H), 2.59 - 2.45 (m, 2H), 2.15 (s, 3H).

^{13}C NMR (100 MHz, $CDCl_3$) δ 207.45, 154.63, 140.32, 136.55, 131.42, 131.34, 130.82, 129.76, 128.24, 128.17, 126.55 (q, J = 275.9 Hz), 125.67, 124.88, 115.49, 68.88, 42.99, 39.31 (q, J = 27.1 Hz), 38.37, 38.21, 20.51.

^{19}F NMR (376 MHz, $CDCl_3$) δ -63.72.

HRMS (APCI) m/z calcd. for $C_{20}H_{20}F_3O_2$ $[M+H]^+$ 349.1415, found 349.1410.

5-tosyl-14-(2,2,2-trifluoroethyl)-6,7,13,14-tetrahydrodibenzo[b,f]azecin-8(5H)-one

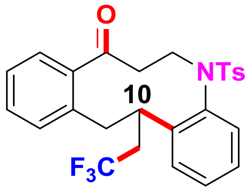

4S

(55% yield), ^{1}H NMR (500 MHz, $CDCl_3$) δ 7.49 (d, J = 8.0 Hz, 2H), 7.25 (d, J = 8.5 Hz, 2H), 7.16 - 7.12 (m, 3H), 7.04 - 6.96 (m, 2H), 6.85 - 6.79 (m, 2H), 6.18 (d, J = 8.0 Hz, 1H), 4.38 - 4.30 (m, 1H), 4.18 - 4.08 (m, 2H), 3.54 - 3.46 (m, 1H), 3.24 - 3.15 (m, 2H), 2.65 - 2.48 (m, 2H), 2.48 - 2.37 (m, 4H).

^{13}C NMR (125 MHz, $CDCl_3$) δ 205.47, 144.03, 143.64, 140.44, 138.71, 137.57, 134.32, 132.99, 130.49, 129.46, 128.81, 128.30, 127.68, 127.07, 127.05, 126.69 (q, J = 276.3 Hz), 126.10, 125.82, 51.43, 44.96, 39.91, 39.58 (q, J = 28.1 Hz), 31.37, 21.53.

^{19}F NMR (376 MHz, $CDCl_3$) δ -62.56.

HRMS (APCI) m/z calcd. for $C_{26}H_{25}F_3NO_3S$ $[M+H]^+$ 488.1507, found 488.1501.

8-methyl-13-(2,2,2-trifluoroethyl)-7,8,13,14-tetrahydrodibenzo[a,e][10]annulen-5(6H)-one

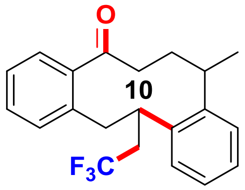

4T

(54% yield), ^{1}H NMR (500 MHz, $CDCl_3$) δ 7.30 - 7.25 (m, 2H), 7.23 - 7.16 (m, 2H), 7.15 - 7.06 (m, 3H), 7.00 (d, J = 8.0 Hz, 1H), 3.75 - 3.68 (m, 1H), 3.45 (dd, J = 15.5, 8.0 Hz, 1H), 3.35 - 3.28 (m, 1H), 3.13 (dd, J = 15.4, 6.0 Hz, 1H), 2.89 - 2.81 (m, 1H), 2.57 - 2.41 (m, 4H), 1.61 - 1.54 (m, 1H), 1.33 (d, J = 7.0 Hz, 3H).

^{13}C NMR (125 MHz, $CDCl_3$) δ 207.57, 143.73, 140.89, 140.54, 137.00, 131.66, 130.53, 128.00, 126.88, 126.59, 126.42, 126.15, 126.14 (q, J = 276.1 Hz), 125.99, 43.27, 41.08 (q, J = 27.0 Hz), 38.85, 34.84, 32.83 (q, J = 2.3 Hz), 30.26, 22.20.

^{19}F NMR (376 MHz, $CDCl_3$) δ -63.99.

HRMS (APCI) m/z calcd. for $C_{21}H_{22}F_3O$ $[M+H]^+$ 347.1623, found 347.1617.

12-fluoro-14-(2,2,2-trifluoroethyl)-6,7,8,9,14,15-hexahydro-5H-dibenzo[a,e][11]annulen-5-one

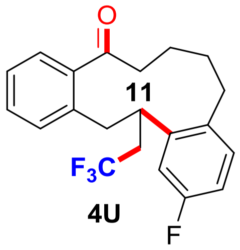

4U

(30% yield), ^{1}H NMR (500 MHz, $CDCl_3$) δ 7.66 (d, *J* = 7.5 Hz, 1H), 7.47 - 7.30 (m, 1H), 7.23 (td, *J* = 7.5, 1.0 Hz, 1H), 7.19 (dd, *J* = 8.0, 1.5 Hz, 1H), 7.14 (dd, *J* = 10.5, 3.0 Hz, 1H), 7.09 (dd, *J* = 9.0, 6.5 Hz, 1H), 6.82 (td, *J* = 8.0, 2.5 Hz, 1H), 4.70 (t, *J* = 7.0 Hz, 1H), 3.25 - 3.19 (m, 1H), 2.93 - 2.86 (m, 1H), 2.61 (dt, *J* = 15.0, 4.5 Hz, 1H), 2.56 - 2.50 (m, 1H), 2.50 - 2.41 (m, 1H), 2.30 - 2.21 (m, 1H), 2.13 - 1.94 (m, 3H), 1.86 - 1.77 (m, 1H), 1.66 - 1.59 (m, 1H), 1.23 - 1.14 (m, 1H).
^{13}C NMR (125 MHz, $CDCl_3$) δ 212.05, 161.45 (d, *J* = 243.1 Hz), 144.54 (d, *J* = 6.3 Hz), 141.59, 140.44, 134.95 (d, *J* = 3.1 Hz), 131.45 (d, *J* = 7.9 Hz), 130.80, 126.98 (q, *J* = 274.5 Hz), 126.31, 126.19, 125.46, 113.87 (d, *J* = 21.0 Hz), 112.32 (d, *J* = 21.1 Hz), 45.52, 38.38, 32.93, 32.26 (q, *J* = 28.6 Hz), 28.08, 27.64 (q, *J* = 2.8 Hz), 21.25.
^{19}F NMR (376 MHz, $CDCl_3$) δ -66.40 (s, 3F), -115.59 (s, 1F).
HRMS (APCI) m/z calcd. for $C_{21}H_{21}F_4O$ $[M+H]^+$ 365.1529, found 365.1510.

Procedure for trifluoromethylation of 1X

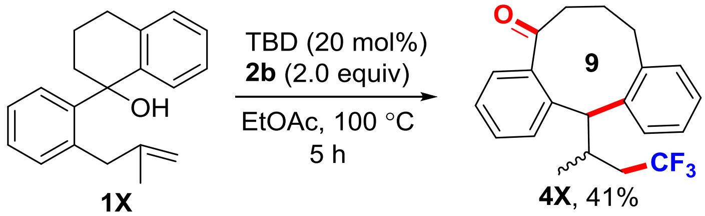

Under argon, a 25 mL Schlenk tube equipped with a magnetic stir bar was charged with **1X** (51 mg, 0.2 mmol, 1.0 equiv), **2b** (126 mg, 0.4 mmol, 2.0 equiv), TBD (5 mg, 0.04 mmol, 0.2 equiv) and EtOAc (2.0 mL). The sealed tube was then stirred at 100 °C for 5 h. After completion (monitored by TLC), EtOAc (40 mL) was added and the reaction mixture was washed with saturated $NaHCO_3$ solution (2 × 5 mL). The organic layer was dried over anhydrous Na_2SO_4, filtered and concentrated to afford the crude product, which was purified by flash column chromatography to afford the product **4X** as diastereomers (28 mg, dr = 2:1, 41%).

The structure of **4X** was confirmed by 2D NMR analysis.

13-(4,4,4-trifluorobutan-2-yl)-6,7,8,13-tetrahydro-5H-dibenzo[a,d][9]annulen-5-one

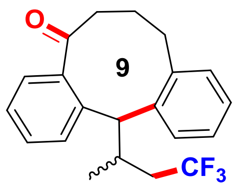

4X, 41%

(41% yield, diasteromers), ^{1}H NMR (400 MHz, $CDCl_3$) δ 7.57 (d, *J* = 7.9 Hz, 1H), 7.44 – 7.39 (m, 1H), 7.24 – 7.08 (m, 5H), 7.00 (d, *J* = 7.5 Hz, 1H), 4.11 – 4.08 (m, 1H), 3.12 – 2.95 (m, 1H), 2.88 – 2.71 (m, 2H), 2.54 – 2.31 (m, 3H), 1.97 – 1.83 (m, 1H), 1.83 – 1.61 (m, 2H), 1.14 (d, *J* = 6.3 Hz, 2H), 0.85 (d, *J* = 6.4 Hz, 1H).

^{13}C NMR (101 MHz, $CDCl_3$) δ 212.44, 212.34, 145.29, 145.14, 140.66, 139.23, 138.13, 138.09, 129.88, 129.73, 129.58, 127.64, 127.33, 127.28 (q, *J* = 278.0 Hz), 127.21 (q, J = 276.0 Hz), 127.08, 126.81, 126.77, 126.75, 126.54, 126.35, 125.81, 125.51, 123.86, 123.78, 47.65, 47.32, 41.25, 41.05, 38.77 (q, *J* = 27.1 Hz), 37.82 (q, *J* = 27.1 Hz), 1, 31.74, 31.72, 31.44, 31.42, 31.40, 30.26, 30.16, 27.57, 18.86, 17.94.

^{19}F NMR (376 MHz, DMSO) δ -61.53, -61.72.

General procedure for phosphonylation reaction of 1

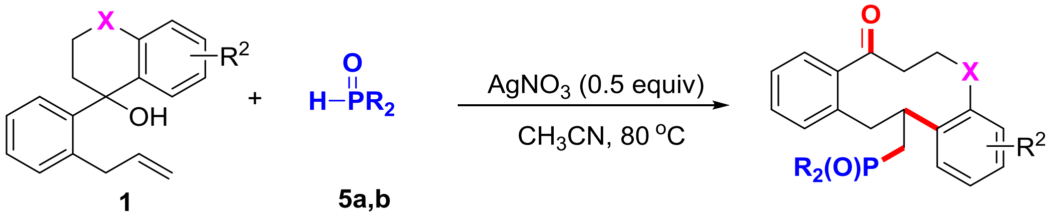

To a flame-dried Schlenk tube equipped with a magnetic stir bar were added **1** (0.2 mmol, 1.0 equiv), **5a**/**5b** (0.4 mmol, 2.0 equiv) and $AgNO_3$ (18 mg, 0.10 mmol, 0.5 equiv). The tube was evacuated and backfilled with argon for three times, and then CH_3CN (2.0 mL) were added. The tube was stirred at 80 °C for 24 h and then H_2O (5 mL) was added. The product was extracted with EtOAc (3 × 10 mL). The combined organic layers were dried over anhydrous Na_2SO_4, filtered and concentrated to afford the crude product, which was purified by flash column chromatography to afford the product **6-8**.

11-bromo-13-((diphenylphosphoryl)methyl)-7,8,13,14-tetrahydrodibenzo[a,e][10] annulen-5(6H)-one

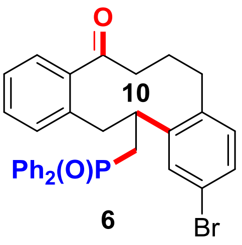

6

(70% yield), ^{1}H NMR (400 MHz, $CDCl_3$) δ 7.61 - 7.54 (m, 2H), 7.46 (td, *J* = 7.6, 1.6 Hz, 1H), 7.41 - 7.25 (m, 7H), 7.25 - 7.18 (m, 3H), 7.15 - 7.10 (m, 2H), 7.00 (dd, *J* = 8.4, 2.4 Hz, 1H), 6.75 (d, *J* = 8.4 Hz, 1H), 3.52 - 3.42 (m, 1H), 3.34 - 3.26 (m, 2H),

3.11 - 3.01 (m, 1H), 2.82 - 2.73 (m, 1H), 2.73 - 2.63 (m, 2H), 2.54 - 2.38 (m, 2H), 2.33 - 2.25 (m, 1H), 1.59 - 1.42 (m, 1H).

^{13}C NMR (100 MHz, $CDCl_3$) δ 206.17, 144.46, 140.01, 138.96, 137.59, 133.87, 133.72 (d, *J* = 66.8 Hz), 132.89, 132.48, 131.60 (d, *J* = 2.6 Hz), 131.50, 131.14 (d, *J* = 2.8 Hz), 131.50 (d, *J* = 33.1 Hz), 131.14 (d, *J* = 2.7 Hz), 130.27, 130.21 (d, *J* = 5.9 Hz), 129.41 (d, *J* = 50.2 Hz), 128.89, 128.59 (d, *J* = 11.6 Hz), 128.02 (d, *J* = 11.7 Hz), 126.88, 120.05, 45.42 (d, *J* = 13.0 Hz), 38.49, 37.00 (d, *J* = 70.5 Hz), 36.29 (d, *J* = 2.8 Hz), 29.16, 26.51.

^{31}P NMR (202 MHz, $CDCl_3$) δ 28.22.

HRMS (APCI) m/z calcd. for $C_{31}H_{28}BrO_2PNa$ $[M+Na]^+$ 565.0908, found 565.0903.

((11-bromo-5-hydroxy-5,6,7,8,13,14-hexahydrodibenzo[a,e][10]annulen-13-yl)methyl)diphenylphosphine oxide (Since **6** is difficult to purify through flash column chromatography, reduction of **6** with $NaBH_4$ in methanol gave pure product **6'**.)

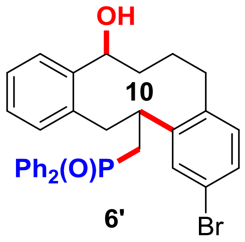

6'

^{1}H NMR (500 MHz, $CDCl_3$) δ 7.74 (dd, *J* = 11.0, 8.0 Hz, 2H), 7.58 (dd, *J* = 11.5, 7.5 Hz, 2H), 7.51 (t, *J* = 7.0 Hz, 1H), 7.46 (t, *J* = 7.5 Hz, 2H), 7.41 (t, *J* = 7.5 Hz, 1H), 7.34 (t, *J* = 7.5 Hz, 2H), 7.23 (d, *J* = 8.0 Hz, 1H), 7.18 (s, 1H), 7.03 (t, *J* = 7.5 Hz, 1H), 6.91 - 6.83 (m, 2H), 6.67 (d, *J* = 7.5 Hz, 1H), 6.47 (d, *J* = 8.5 Hz, 1H), 5.32 (s, 1H), 4.19 - 4.08 (m, 1H), 3.81 (dd, *J* = 14.0, 6.0 Hz, 1H), 3.54 (s, 1H), 2.78 - 2.61 (m, 3H), 2.33 - 2.20 (m, 2H), 2.07 - 1.98 (m, 1H), 1.92 - 1.82 (m, 1H), 1.80 - 1.72 (m, 1H), 0.28 (t, *J* = 13.5 Hz, 1H).

^{13}C NMR (125 MHz, $CDCl_3$) δ 142.20, 141.78, 141.72, 135.86, 133.81 (d, *J* = 98.3 Hz), 131.89, 131.85 (d, *J* = 2.5 Hz), 131.63 (d, *J* = 98 Hz), 131.54 (d, *J* = 2.5 Hz), 131.34, 130.73 (d, *J* = 9.1 Hz), 130.36, 130.29, 129.27 (d, *J* = 12.4 Hz), 125.35 (d, *J* = 6.8 Hz), 128.80, 128.70, 128.39 (d, *J* = 11.8 Hz), 126.92 (d, *J* = 11.8 Hz), 126.25, 119.33, 67.13, 43.78 (d, *J* = 7.8 Hz), 38.31, 38.22 (d, *J* = 69.3 Hz), 34.64, 28.92, 26.59.

^{31}P NMR (202 MHz, CDCl3) δ 30.53.

HRMS (APCI) m/z calcd. for $C_{31}H_{31}BrO_2P$ $[M+H]^+$ 545.1245, found 545.1221.

14-((diphenylphosphoryl)methyl)-5-tosyl-6,7,13,14-tetrahydrodibenzo[b,f]azecin-8(5H)-one

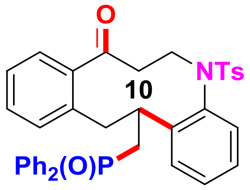

7

(65% yield), ^{1}H NMR (500 MHz, $CDCl_3$) δ 8.20 - 8.14 (m, 2H), 7.86 – 7.81 (m, 2H), 7.74 - 7.69 (m, 3H), 7.50 (d, *J* = 8.0 Hz, 2H), 7.44 - 7.38 (m, 3H), 7.33 (d, *J* = 8.0 Hz, 1H), 7.27 (d, *J* = 8.0 Hz, 2H), 7.13 (t, *J* = 7.5 Hz, 1H), 6.96 (t, *J* = 7.5 Hz, 1H), 6.91 (t, *J* = 6.5 Hz, 2H), 6.86 (t, *J* = 7.0 Hz, 1H), 6.76 (t, *J* = 7.5 Hz, 1H), 5.95 (d, *J* = 8.0 Hz, 1H), 4.28 - 4.18 (m, 1H), 4.10 (dd, *J* = 14.0, 6.0 Hz, 1H), 4.05 - 3.99 (m, 1H), 3.21 - 3.09 (m, 3H), 3.02 - 2.93 (m, 2H), 2.73 - 2.62 (m, 1H), 2.44 (s, 3H).
^{13}C NMR (125 MHz, $CDCl_3$) δ 204.42, 143.96, 139.87, 139.24, 137.91, 135.17 (d, *J* = 97.8 Hz), 134.36, 133.12, 131.92 (d, *J* = 2.5 Hz), 131.29 (d, *J* = 9.6 Hz), 131.22 (d, *J* = 2.5 Hz), 130.09, 130.43 (d, *J* = 9.1 Hz), 129.46, 129.01,128.52 (d, *J* = 11.4 Hz), 128.93, 128.15, 127.87,126.80 (d, *J* = 31.4 Hz), 51.06, 44.51, 39.79, 37.09 (d, *J* = 67.1 Hz), 33.37, 21.55.
^{31}P NMR (162 MHz, $CDCl_3$) δ 31.67.
HRMS (APCI) m/z calcd. for $C_{37}H_{35}NO_4PS$ $[M+H]^+$ 620.2024, found 620.2018.

11-bromo-13-((dibenzylphosphoryl)methyl)-7,8,13,14-tetrahydrodibenzo[a,e][10] annulen-5(6H)-one

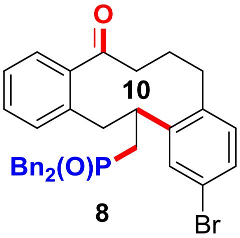

8

(66% yield), ^{1}H NMR (500 MHz, $CDCl_3$) δ 7.45 (d, *J* = 7.0 Hz, 1H), 7.30 (t, *J* = 6.0 Hz, 2H), 7.27 - 7.24 (m, 2H), 7.22 - 7.13 (m, 6H), 7.07 - 7.03 (m, 2H), 6.98 - 6.90 (m, 4H), 3.41 - 3.33 (m, 1H), 3.29 (d, *J* = 15.0 Hz, 1H), 3.26 - 3.14 (m, 2H), 3.10 (t, *J* = 13.0 Hz, 1H), 2.82 - 2.76 (m, 1H), 2.75 - 2.66 (m, 3H), 2.62 (d, *J* = 13.5 Hz, 2H), 2.49 - 2.41 (m, 1H), 2.06 - 1.97 (m, 2H), 1.73 - 1.64 (s, 1H).
^{13}C NMR (125 MHz, $CDCl_3$) δ 207.24, 145.39, 140.30, 138.79, 137.55, 132.72, 131.54, 131.28 (d, *J* = 7.4 Hz), 131.05, 130.36, 129.75 (d, *J* = 5.0 Hz), 129.21, 129.16, 128.72 (d, *J* = 2.0 Hz), 128.62 (d, *J* = 2.0 Hz), 127.30, 126.89 (d, *J* = 2.6 Hz), 126.77 (d, *J* = 2.6 Hz), 120.90, 45.86 (d, *J* = 13.3 Hz), 38.51, 36.48, 36.37 (d, *J* = 59.9 Hz), 35.17 (d, *J* = 60.0 Hz), 33.76 (d, *J* = 63.3 Hz), 29.51, 26.83.
^{31}P NMR (202 MHz, $CDCl_3$) δ 41.66.
HRMS (APCI) m/z calcd. for $C_{33}H_{33}BrO_2P$ $[M+H]^+$ 571.1402, found 571.1375.

Procedure for sulfonylation reaction of 1l

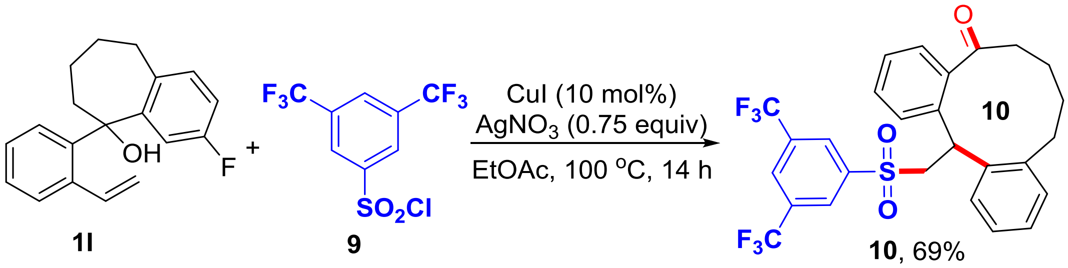

Under argon, a 25 mL Schlenk tube equipped with a magnetic stir bar was charged

with **1l** (57 mg, 0.2 mmol, 1.0 equiv), **9** (93 mg, 0.30 mmol, 1.5 equiv), CuI (1.8 mg, 0.02 mmol, 0.1 equiv), Ag_2CO_3 (42 mg, 0.15 mmol, 0.75 equiv) and EtOAc (2.0 mL). The sealed tube was then stirred at 100 °C for 14 h. After completion (monitored by TLC), EtOAc (30 mL) was added and the reaction mixture was washed with brine (2 × 5 mL). The organic layer was dried over anhydrous Na_2SO_4, filtered and concentrated to afford the crude product, which was purified by flash column chromatography to afford the product **10** (76 mg, 69%).

14-(((3,5-bis(trifluoromethyl)phenyl)sulfonyl)methyl)-7,8,9,14-tetrahydrodibenzo [a,d][10]annulen-5(6H)-one

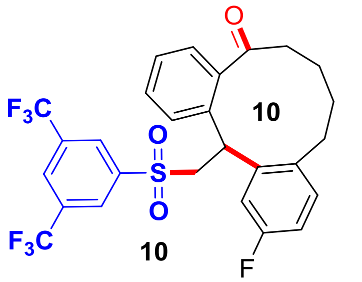

10

(69% yield), ^1H NMR (400 MHz, $CDCl_3$) δ 8.22 (s, 2H), 7.89 (s, 1H), 7.34 (d, *J* = 7.9 Hz, 1H), 7.30 – 7.22 (m, 1H), 7.14 (d, *J* = 4.1 Hz, 2H), 7.05 (dd, *J* = 8.6, 6.0 Hz, 1H), 6.81 (dd, *J* = 10.2, 2.6 Hz, 1H), 6.77 – 6.69 (m, 1H), 5.42 (t, *J* = 6.5 Hz, 1H), 4.15 (dd, *J* = 15.0, 7.1 Hz, 1H), 4.05 (dd, *J* = 15.0, 7.1 Hz, 1H), 3.20 (ddd, *J* = 13.1, 8.9, 1.6 Hz, 1H), 3.04 – 2.93 (m, 1H), 2.66 (dt, *J* = 14.8, 4.0 Hz, 1H), 2.60 – 2.51 (m, 1H), 2.12 – 1.95 (m, 1H), 1.93 – 1.79 (m, 1H), 1.67 – 1.53 (m, 1H), 1.10 – 1.00 (m, 1H).
^{13}C NMR (126 MHz, $CDCl_3$) δ 210.89, 161.09 (d, *J* = 244.5 Hz), 141.88 (d, *J* = 5.3 Hz), 140.60, 138.24, 134.84 (d, *J* = 3.1 Hz), 132.46 (q, *J* = 34.4 Hz), 131.97 (d, *J* = 7.9 Hz), 130.73, 128.74 (q, *J* = 3.3 Hz), 127.07 (sept, *J* = 3.5 Hz), 126.93, 126.06, 125.96, 122.25 (q, *J* = 271.8 Hz), 114.71 (d, *J* = 20.8 Hz), 112.46 (d, *J* = 21.4 Hz), 60.79, 45.41, 34.94, 32.97, 28.05, 20.87.
^{19}F NMR (376 MHz, $CDCl_3$) δ -62.90, -115.01.
HRMS (ESI) m/z calcd. for $C_{27}H_{23}F_6O_2S$ $[M+H]^+$ 541.1266, found 541.1252.

Procedure for perfluoroalkylation reaction of 1l

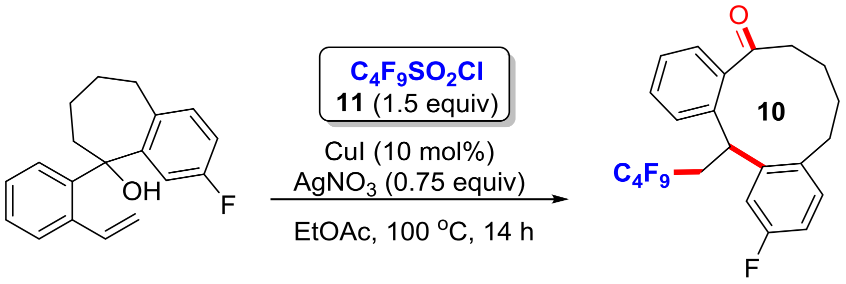

1l **12**, 59%

Under argon, a 25 mL Schlenk tube equipped with a magnetic stir bar was charged with **1l** (57 mg, 0.2 mmol, 1.0 equiv), **11** (96 mg, 0.30 mmol, 1.5 equiv), CuI (1.8 mg, 0.02 mmol, 0.1 equiv), Ag_2CO_3 (42 mg, 0.15 mmol, 0.75 equiv) and EtOAc (2.0 mL). The sealed tube was then stirred at 100 °C for 14 h. After completion (monitored by

TLC), EtOAc (30 mL) was added and the reaction mixture was washed with brine (2 × 5 mL). The organic layer was dried over anhydrous Na_2SO_4, filtered and concentrated to afford the crude product, which was purified by flash column chromatography to afford the product **12** (70 mg, 59%).

12-fluoro-14-(2,2,3,3,4,4,5,5,5-nonafluoropentyl)-7,8,9,14-tetrahydrodibenzo[a,d][10]annulen-5(6H)-one

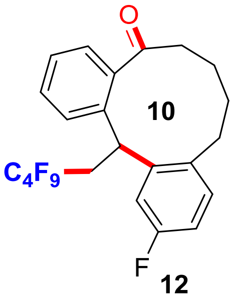

12

(59% yield), ^{1}H NMR (400 MHz, $CDCl_3$) δ 7.65 (d, *J* = 7.9 Hz, 1H), 7.53 – 7.42 (m, 1H), 7.25 (ddd, *J* = 9.5, 8.3, 1.9 Hz, 2H), 7.19 – 7.06 (m, 2H), 6.85 (td, *J* = 8.3, 2.6 Hz, 1H), 5.35 (t, *J* = 6.5 Hz, 1H), 3.25 (ddd, *J* = 13.2, 8.9, 1.7 Hz, 1H), 3.13 – 2.90 (m, 3H), 2.78 – 2.50 (m, 2H), 2.12 – 1.86 (m, 2H), 1.70 – 1.61 (m, 1H), 1.26 – 1.14 (m, 1H).

^{13}C NMR (101 MHz, $CDCl_3$) δ 211.42, 161.29 (d, *J* = 244.5 Hz), 143.96 (d, *J* = 6.1 Hz), 140.87, 140.63, 134.62 (d, *J* = 3.1 Hz), 131.56 (d, *J* = 7.9 Hz), 130.79, 126.54, 126.42, 125.78, 126.23 – 108.55, 114.11 (d, *J* = 20.8 Hz), 112.63 (d, *J* = 21.3 Hz), 45.52, 36.11 (t, *J* = 20.8 Hz), 32.90, 32.15, 27.89, 21.02.

^{19}F NMR (376 MHz, $CDCl_3$) δ -80.86 – -81.52 (m), -112.33 – -114.60 (m), -115.65 (s), -124.32 (dd, *J* = 11.9, 6.8 Hz), -125.88 (t, *J* = 11.0 Hz).

HRMS (ESI) m/z calcd. for $C_{23}H_{20}F_9OS$ $[M+H]^+$ 483.1365, found 483.1356.

Procedure for synthesis of enantioenriched medium-sized ketones through chirality transfer strategy

The chiral tertiary alcohols (**1A** and **1Q**) were prepared by pre-HPLC. Otherwise, asymmetric synthesis of the chiral tertiary alcohols has been reported by literature.[1-3]

Compound 1A: Pre-HPLC conditions: [Daicel Chiralpak OD-H, 20mmΦ×250mml, Particle 5μm, isopropanol/hexane = 10/90, 10 mL/min, λ= 254 nm, t_R = 9.6 min, t_R = 12.6 min].

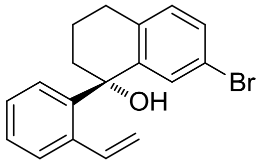

(*R*)-**1A**

(*R*)-**1A**: 100% ee, HPLC analysis [Daicel Chiralpak OD-H, isopropanol/hexane = 10/90, 0.5 mL/min, λ= 254 nm, t_R (major) = 12.86 min].

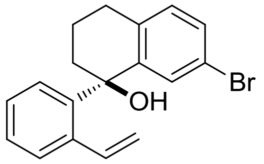

(*S*)-**1A**

(*S*)-**1A**: 100% ee, HPLC analysis [Daicel Chiralpak OD-H, isopropanol/hexane = 10/90, 0.5 mL/min, λ= 254 nm, t_R (major) = 9.4 min].

Compound 1Q: Pre-HPLC conditions: [Daicel Chiralpak OD-H, 20mmΦ×250mml, Particle 5μm, isopropanol/hexane = 5/95, 10 mL/min, λ= 214 nm, t_R = 10.2 min, t_R = 14.2 min].

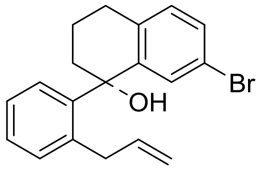

1Q[*1]

>99% ee, HPLC analysis [Daicel Chiralpak OD-H, isopropanol/hexane = 10/90, 0.5 mL/min, λ= 214 nm, t_R (major) = 11.3 min].

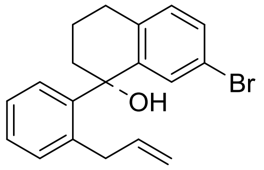

1Q[*2]

>99% ee, HPLC analysis [Daicel Chiralpak OD-H, isopropanol/hexane = 10/90, 0.5 mL/min, λ= 214 nm, t_R (major) = 15.6 min].

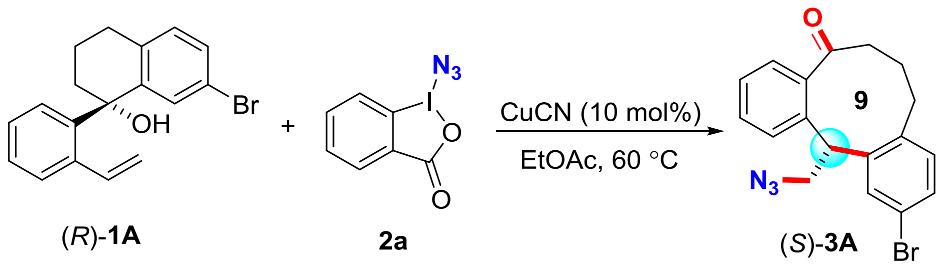

The procedure for synthesis of (*S*)-**3A** is similar to that of racemic **3A**.

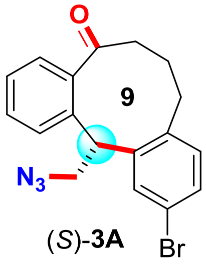

(*S*)-**3A**

(*S*)-**3A**: 99.4% ee, HPLC analysis [Daicel Chiralpak AD-H, isopropanol/hexane = 20/80, 1.0 mL/min, λ= 214 nm, t_R (major) = 11.2 min, t_R (minor) = 7.3 min].

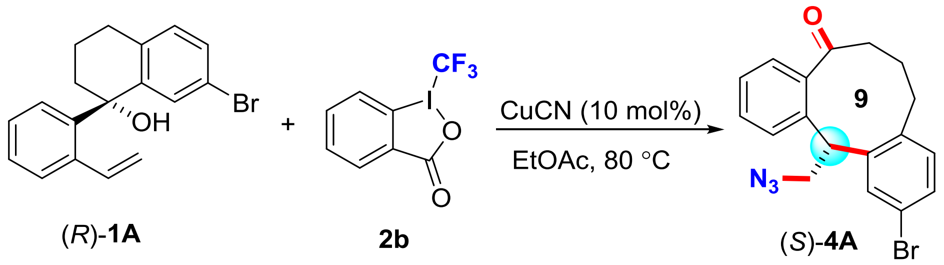

The procedure for synthesis of (*S*)-**4A** and (*R*)-**4A** is similar to that of racemic **4A**.

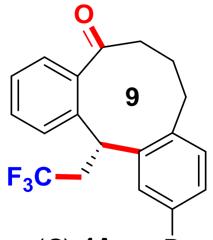

(*S*)-**4A**

(*S*)-**4A**: 99% ee, HPLC analysis [Daicel Chiralpak AD-H, isopropanol/hexane = 10/90, 1.0 mL/min, λ= 214 nm, t_R (major) = 6.5 min, t_R (minor) = 9.5 min].

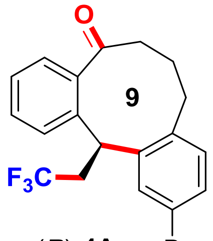

(*R*)-**4A**

(*R*)-**4A**: 98% ee, HPLC analysis [Daicel Chiralpak AD-H, isopropanol/hexane = 10/90, 1.0 mL/min, λ= 214 nm, t_R (minior) = 6.6 min, t_R (major) = 9.2 min].

The procedure for synthesis of **4Q*** and **8*** is similar to that of racemic **4Q** and **8**.

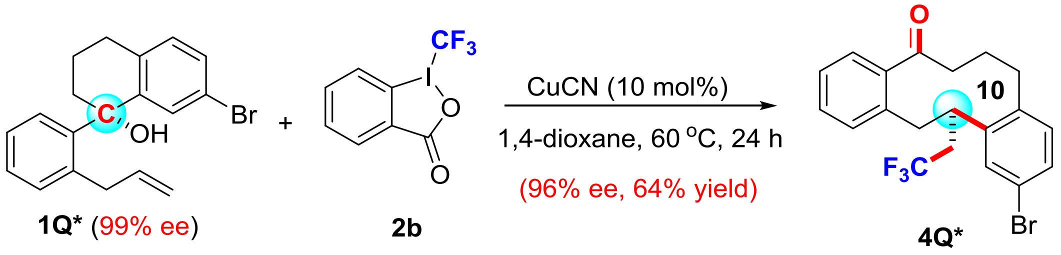

4Q*: 96% ee, HPLC analysis [Daicel Chiralpak OD-H, isopropanol/hexane = 5/95, 1.0 mL/min, λ= 254 nm, t_R (major) = 8.2 min, t_R (major) = 17.8 min].

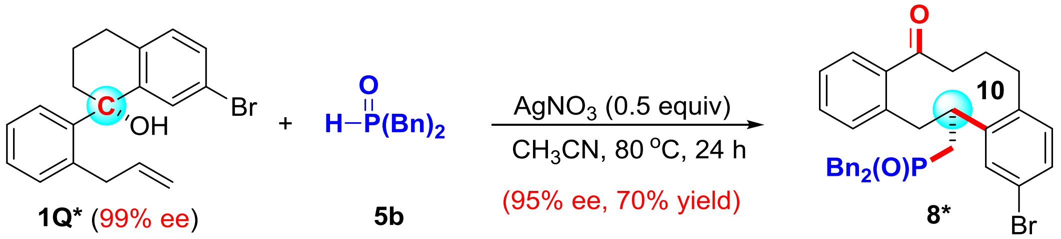

Compound 8*: 95% ee, HPLC analysis [Daicel Chiralpak OD-H, isopropanol/hexane = 20/80, 0.8 mL/min,λ= 214 nm, t_R (major) = 17.7 min, t_R (major) = 28.5 min].

Mechanistic Study

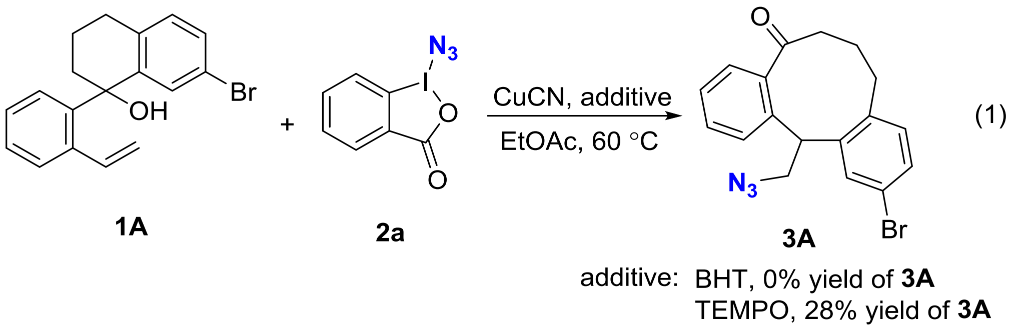

Under argon, a 25 mL Schlenk tube equipped with a magnetic stir bar was charged with **1A** (66 mg, 0.2 mmol, 1.0 equiv), **2a** (70 mg, 0.24 mmol, 1.2 equiv), CuCN (1.8 mg, 0.02 mmol, 0.1 equiv), 2,6-di-tert-butyl-4-methylphenol (BHT, 88 mg, 0.4 mmol, 2.0 equiv) or 2,2,6,6-Tetramethylpiperidinooxy (TEMPO, 63 mg, 0.4 mmol, 2.0 equiv) and EtOAc (2.0 mL). The sealed tube was then stirred at 60 °C for 12 h. After completion, the reaction solution was added with EtOAc (30 mL), followed by washing with saturated $NaHCO_3$ (2 × 5 mL) solution. The organic layer was dried over anhydrous Na_2SO_4, filtered and concentrated to afford the crude product. Mesitylene (internal standard, 24.0 mg, 0.2 mmol) was added. ^{1}H NMR analysis of this reaction mixture showed that **3A** was formed in 0 and 28% yield, respectively.

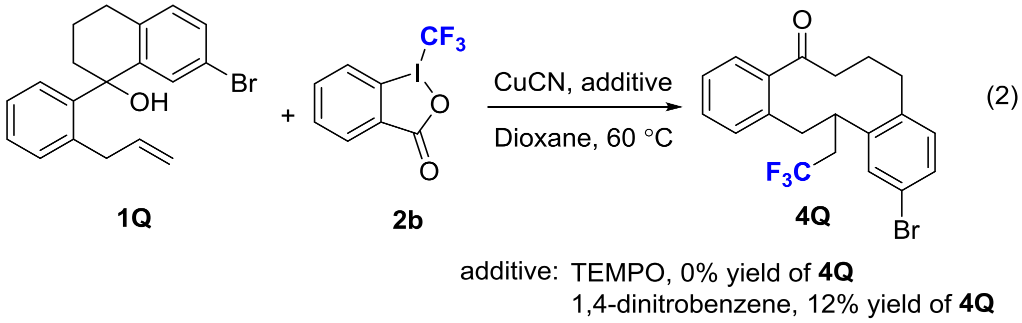

Procedure: Under argon, a 25 mL Schlenk tube equipped with a magnetic stir bar was charged with **1Q** (69 mg, 0.2 mmol, 1.0 equiv), **2b** (126 mg, 0.4 mmol, 2.0 equiv), CuCN (1.8 mg, 0.02 mmol, 0.1 equiv), 2,2,6,6-Tetramethylpiperidinooxy (63 mg, 0.4 mmol, 2.0 equiv) or 1,4-dinitrobenzene (67 mg, 0.4 mmol, 2.0 equiv) and Dioxane (4.0 mL). The sealed tube was then stirred at 60 °C for 24 h. After completion, $PhCF_3$ (internal standard, 29.2 mg, 0.2 mmol) was added. ^{19}F NMR analysis of this reaction mixture showed that **4Q** was formed in 0 and 12% yield, respectively.

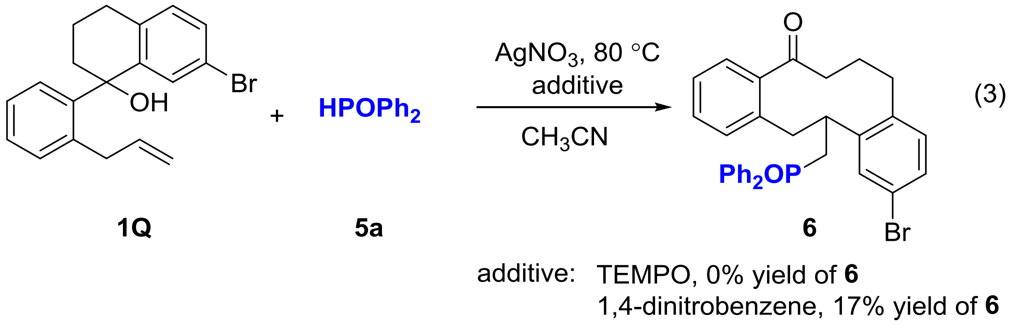

Procedure: Under argon, a 25 mL Schlenk tube equipped with a magnetic stir bar was charged with **1Q** (69 mg, 0.2 mmol, 1.0 equiv), **5a** (0.4 mmol, 2.0 equiv), $AgNO_3$ (18 mg, 0.1 mmol, 0.5 equiv), 2,2,6,6-tetramethylpiperidinooxy (63 mg, 0.4 mmol, 2.0 equiv) or 1,4-dinitrobenzene (67 mg, 0.4 mmol, 2.0 equiv) and CH_3CN (2.0 mL). The sealed tube was stirred at 80 °C for 24 h and then H_2O (5 mL) was added. EtOAc was used to extract the product from the aqueous layer (3 × 20 mL). The combined organic layer was dried over anhydrous Na_2SO_4, filtered and concentrated to afford the crude product. Mesitylene (internal standard, 24.0 mg, 0.2 mmol) was added. ^1H NMR analysis of this reaction mixture showed that **6** was formed in 0 and 17% yield, respectively.

Procedure for synthetic application:

General procedure for synthesis of medium-bridged lactams and amines 13-16

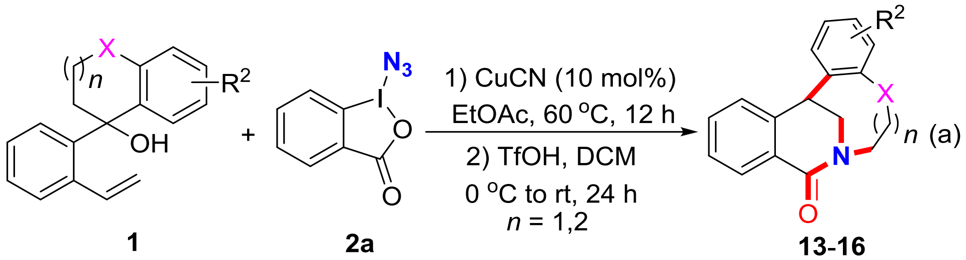

Under argon, a 25 mL Schlenk tube equipped with a magnetic stir bar was charged with **1** (0.3 mmol, 1.0 equiv), **2a** (104 mg, 0.36 mmol, 1.2 equiv), CuCN (2.7 mg, 0.03 mmol, 0.1 equiv) and EtOAc (3.0 mL). The sealed tube was then stirred at 60 °C for 10 h. After completion (monitored by TLC), the reaction solution was added with EtOAc (30 mL), which was washed with saturated $NaHCO_3$ (2 × 5 mL), and brine (5 mL) sequentially. The organic phase was dried over anhydrous Na_2SO_4, filtered and concentrated to afford the crude product, which was transferred to a 25 mL round-bottom flask in argon. Dry DCM (3.0 mL) and TfOH (0.6 mmol, 2 equiv) were added at 0 °C and the solution was stirred for 24 h at room temperature. After completion (monitored by TLC), the reaction solution was added with DCM (30 mL), which was washed with saturated $NaHCO_3$ (2 × 5 mL) and brine (5 mL). The organic layer was dried over anhydrous Na_2SO_4, filtered and concentrated to afford the crude product, which was purified by flash column chromatography to afford the product **13-16**.

12-bromo-7,8,9,14-tetrahydro-5H-6,14-methanodibenzo[c,f]azecin-5-one

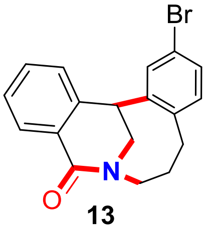

13

(62% yield), ^{1}H NMR (400 MHz, $CDCl_3$) δ 8.12 - 8.01 (m, 1H), 7.52 - 7.41 (m, 2H), 7.41 - 7.31 (m, 2H), 7.23 (dd, *J* = 8.0, 2.0 Hz, 1H), 6.94 (d, *J* = 8.0 Hz, 1H), 4.65 - 4.55 (m, 1H), 4.14 (dd, *J* = 14.0, 4.4 Hz, 1H), 4.07 (d, *J* = 4.0 Hz, 1H), 3.85 (d, *J* = 14.4 Hz, 1H), 2.98 - 2.90 (m, 1H), 2.83 (dd, *J* = 14.8, 11.2 Hz, 1H), 2.35 (dd, *J* = 15.2, 7.6 Hz, 1H), 2.27 - 2.16 (m, 1H), 1.89 - 1.80 (m, 1H).

^{13}C NMR (100 MHz, $CDCl_3$) δ 165.68, 141.69, 141.37, 140.07, 134.97, 134.95, 131.78, 130.25, 129.35, 128.70, 127.2, 127.52, 119.98, 48.59, 44.67, 31.46, 28.25, 21.77.

HRMS (APCI) m/z calcd. for $C_{18}H_{17}BrNO$ $[M+H]^+$ 342.0494, found 342.0488.

99.6% ee, HPLC analysis [Daicel Chiralpak IA, isopropanol/hexane = 20/80, 1.0 mL/min, λ= 214 nm, t_R (major) = 9.5 min, t_R (minor) = 12.1 min].

12-fluoro-7,8,9,14-tetrahydro-5H-6,14-methanodibenzo[c,f]azecin-5-one

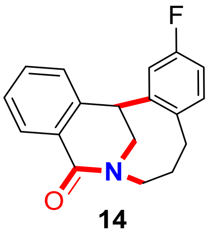

14

(56% yield), ^{1}H NMR (400 MHz, $CDCl_3$) δ 8.05 (d, J = 8.0 Hz, 1H), 7.48 - 7.42 (m, 1H), 7.39 - 7.34 (m, 2H), 7.05 - 6.99 (m, 2H), 6.79 (td, J = 8.0, 2.8 Hz, 1H), 4.65 - 4.56 (m, 1H), 4.14 (dd, J = 14.0, 4.0 Hz, 1H), 4.07 (d, J = 4.0 Hz, 1H), 3.96 (d, J = 14.0 Hz, 1H), 2.98 - 2.87 (m, 2H), 2.45 (dd, J = 15.2, 7.6 Hz, 1H), 2.24 - 2.13 (m, 1H), 1.92 - 1.84 (m, 1H).

^{13}C NMR (100 MHz, $CDCl_3$) δ 165.40, 160.95 (d, J = 243.3 Hz), 142.14, 140.69, 136.57 (d, J = 3.3 Hz), 134.88 (d, J = 7.6 Hz), 131.64, 129.43, 128.65, 127.66, 127.41, 118.84 (d, J = 21.4 Hz), 113.87 (d, J = 20.0 Hz), 48.64, 44.80, 44.60, 31.51, 28.53.

^{19}F NMR (376 MHz, $CDCl_3$) δ -117.25.

HRMS (APCI) m/z calcd. for $C_{18}H_{17}FNO$ $[M+H]^+$ 282.1294, found 282.1289.

13-fluoro-7,8,9,10-tetrahydro-6,15-methanodibenzo[c,f][1]azacycloundecin-5(15 H)-one

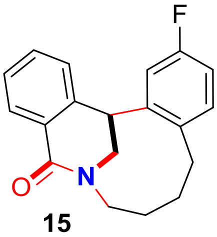

15

(40% yield), ^{1}H NMR (500 MHz, $CDCl_3$) δ 8.18 (dd, J = 7.5, 1.5 Hz, 1H), 7.44 - 7.36 (m, 2H), 7.22 - 7.19 (m, 1H), 7.10 - 7.04 (m, 2H), 6.98 (td, J = 8.5, 3.0 Hz, 1H), 4.77 - 4.69 (m, 1H), 4.16 (dd, J = 13.5, 6.0 Hz, 1H), 4.11 (d, J = 6.0 Hz, 1H), 3.57 (d, J = 13.5 Hz, 1H), 2.82 (dd, J = 13.5, 1.0 Hz, 1H), 2.46 (td, J = 14.0, 4.5 Hz, 1H), 2.39 - 2.32 (m, 1H), 1.99 - 1.90 (m, 1H), 1.76 - 1.67 (m, 1H), 1.46 - 1.34 (m, 2H).

^{13}C NMR (125 MHz, $CDCl_3$) δ 165.82, 160.86 (d, J = 243.4 Hz), 143.19 (d, J = 6.1 Hz), 139.78, 135.91 (d, J = 3.3 Hz), 133.72 (d, J = 7.8 Hz), 131.90, 130.47, 128.65, 128.55, 127.58, 118.22 (d, J = 20.8 Hz), 114.66 (d, J = 20.1 Hz), 48.66, 46.36, 45.28, 30.63, 29.71, 21.55.

^{19}F NMR (376 MHz, $CDCl_3$) δ -117.65.

HRMS (APCI) m/z calcd. for $C_{19}H_{19}FNO$ $[M+H]^+$ 296.1451, found 296.1445.

6,7,8,9-tetrahydro-10,16-methanodibenzo[h,k][1]oxa[6]azacyclododecin-11(16H)-one

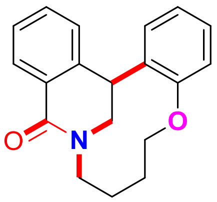

16

(40% yield), ^{1}H NMR (400 MHz, $CDCl_3$) δ 8.18 (dd, *J* = 7.7, 1.0 Hz, 1H), 7.42 – 7.17 (m, 5H), 6.94 (t, *J* = 7.2 Hz, 1H), 6.76 (d, *J* = 8.1 Hz, 1H), 4.86 (td, *J* = 13.4, 3.8 Hz, 1H), 4.30 – 4.19 (m, 2H), 4.11 (dd, *J* = 13.4, 8.1 Hz, 1H), 3.71 (d, *J* = 13.4 Hz, 1H), 3.60 (ddd, *J* = 11.9, 8.9, 2.6 Hz, 1H), 2.70 (ddd, *J* = 13.8, 3.7, 2.7 Hz, 1H), 2.29 – 2.16 (m, 1H), 2.10 – 1.99 (m, 1H), 1.94 – 1.81 (m, 1H), 1.80 – 1.66 (m, 1H); ^{13}C NMR (101 MHz, $CDCl_3$) δ 163.51, 158.43, 137.96, 133.32, 131.30, 130.20, 128.62, 128.49, 128.09, 127.98, 126.92, 120.51, 113.50, 70.95, 47.24, 44.95, 41.78, 26.14, 25.60. HRMS (ESI) m/z calcd. for $C_{19}H_{20}NO_2$ $[M+H]^+$ 294.1488, found 294.1485.

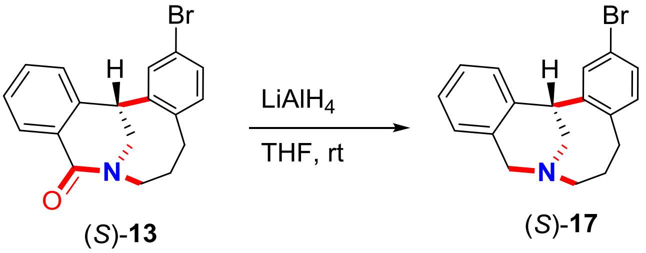

To a cooled solution of (*S*)-**13** (9 mg, 0.03 mmol) in dry THF (2 mL) was added $LiAlH_4$ (12 mg, 0.3 mmol, 10 equiv) at 0 °C and the solution was stirred for 12 h at room temperature. After completion (monitored by TLC), the reaction solution was quenched with water (0.1 mL). EtOAc (10 mL) was then added and the organic layer was dried over anhydrous Na_2SO_4, filtered and concentrated to afford the crude product, which was purified by flash column chromatography to afford the title product (*S*)-**17** (6.9 mg, 79% yield).

12-bromo-7,8,9,14-tetrahydro-5H-6,14-methanodibenzo[c,f]azecine

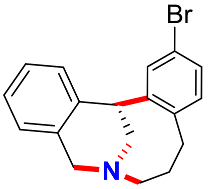

(*S*)-**17**

(79%), ^{1}H NMR (500 MHz, $CDCl_3$) δ 7.29 (d, *J* = 7.0 Hz, 1H), 7.24 - 7.14 (m, 3H), 7.13 - 7.04 (m, 3H), 6.97 (d, *J* = 7.5 Hz, 1H), 4.35 (d, *J* = 17.5 Hz, 1H), 4.06 - 4.00 (m, 2H), 3.71 (dd, *J* = 14.0, 5.0 Hz, 1H), 3.41 - 3.35 (m, 1H), 3.17 (d, *J* = 14.0 Hz, 1H), 2.63 - 2.55 (m, 1H), 2.21 - 2.15 (m, 1H), 2.12 - 2.03 (m, 2H), 1.70 - 1.58 (m, 1H). ^{13}C NMR (125 MHz, $CDCl_3$) δ 144.37, 139.47, 137.19, 136.80, 131.84, 130.77, 130.25, 126.80, 126.56, 126.49, 126.39, 125.38, 58.79, 52.05, 48.77, 45.16, 31.80, 29.89.

HRMS (APCI) m/z calcd. for $C_{18}H_{18}BrNNa$ $[M+Na]^+$ 350.0520, found 350.1857
>99.9% ee, HPLC analysis [Daicel Chiralpak OD-H, isopropanol/hexane = 35/65, 1.0 mL/min, λ= 254 nm, t_R (major) = 8.9 min, t_R (minor) = 12.6 min].

Transformation of azido ketone 3 to analogues of isopavine 18-23

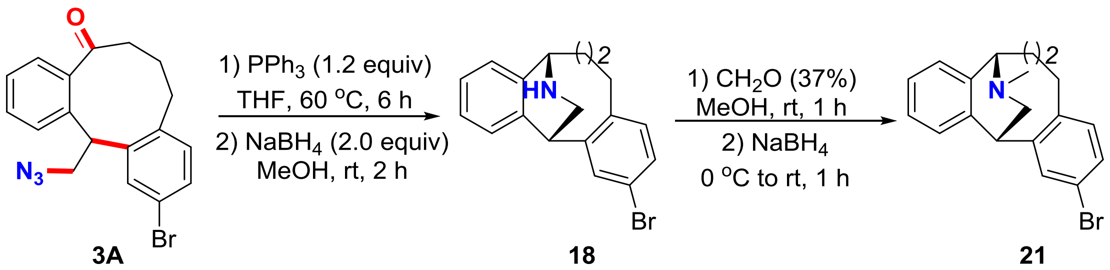

To a solution of **3Aa** (150 mg, 0.4 mmol, 1.0 equiv) in THF (6.0 mL) was added PPh_3 (128 mg, 0.48 mmol, 1.2 equiv). The resulting mixture was then stirred at 60 °C for 6 h. After completion, the solvent was removed under reduced pressure. Then the crude product was dissolved in MeOH (6.0 mL) and $NaBH_4$ (30 mg, 0.8 mmol, 2.0 equiv) was added in two portions. After the mixture was stirred at room temperature for 2 h, it was extracted with ethyl acetate (2 × 30 mL) and the organic phase was dried over $MgSO_4$, filtered and concentrated to afford the crude product, which was purified by flash column chromatography to give **18** (95 mg, 77%) as a colorless oil.
The synthesis of **19** and **20** is similar to that of **18**.

11-bromo-6,7,8,13-tetrahydro-5H-5,13-(epiminomethano)dibenzo[a,d][9]annulene

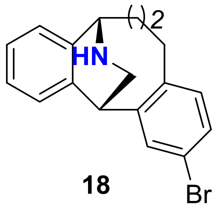

18

(77% yield), ^{1}H NMR (400 MHz, $CDCl_3$) δ 7.44 (d, *J* = 1.9 Hz, 1H), 7.31 – 7.24 (m, 2H), 7.20 – 7.14 (m, 2H), 7.04 (d, *J* = 7.4 Hz, 1H), 6.83 (d, *J* = 8.2 Hz, 1H), 4.53 (d, *J* = 5.7 Hz, 1H), 4.15 (s, 1H), 3.28 (dd, *J* = 12.9, 3.4 Hz, 1H), 3.15 (dd, *J* = 12.9, 1.9 Hz, 1H), 2.17 – 1.86 (m, 6H), 1.68 – 1.60 (m, 1H).
^{13}C NMR (100 MHz, $CDCl_3$) δ 143.58, 143.21, 141.94, 136.57, 134.83, 134.14, 129.81, 128.70, 127.57, 126.32, 126.22, 119.84, 53.79, 50.43, 47.80, 34.05, 27.45, 25.75.
HRMS (ESI) m/z calcd. for $C_{18}H_{18}BrN$ $[M+H]^+$ 328.0695, found: 328.0693.

5,6,7,8,9,14-hexahydro-5,14-(epiminomethano)dibenzo[a,d][10]annulene

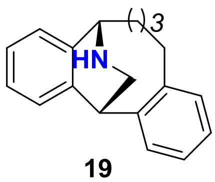

19

(80% yield), ^{1}H NMR (400 MHz, $CDCl_3$) δ 7.35 – 7.32 (m, 2H), 7.27 – 7.17 (m, 5H), 7.03 (dd, J = 7.6, 1.2 Hz, 1H), 4.57 (t, J = 4.0 Hz, 1H), 4.25 (s, 1H), 3.33 (s, 2H), 2.30 – 2.09 (m, 3H), 1.92 – 1.73 (m, 2H), 1.64 – 1.49 (m, 3H), 1.38 – 1.31 (m, 1H).
^{13}C NMR (100 MHz, $CDCl_3$) δ 142.50, 141.83, 140.83, 138.96, 132.03, 131.08, 129.33, 126.93, 126.41, 126.19, 125.99, 125.76, 54.66, 52.60, 47.23, 36.93, 29.91, 29.57, 21.47.
HRMS (ESI) m/z calcd. for $C_{19}H_{21}N$ $[M+H]^+$ 264.1747, found: 264.1739.

6,7,8,9,10,15-hexahydro-10,15-(epiminomethano)dibenzo[b,e][1]oxacycloundecine

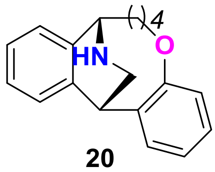

20

(92% yield), ^{1}H NMR (400 MHz, $CDCl_3$) δ 7.33 – 7.28 (m, 2H), 7.23 – 7.15 (m, 2H), 7.04 (t, J = 7.4 Hz, 1H), 6.98 – 6.94 (m, 2H), 6.67 (d, J = 8.0 Hz, 1H), 4.43 (s, 1H), 3.88 – 3.80 (m, 2H), 3.52 – 3.38 (m, 3H), 2.91 (brs, 1H), 2.62 – 2.56 (m, 1H), 2.28 – 2.22 (m, 1H), 1.78 – 1.56 (m, 4H).
^{13}C NMR (100 MHz, $CDCl_3$) δ 156.17, 139.35, 137.88, 133.58, 131.05, 129.21, 128.15, 125.76, 125.07, 125.05, 120.50, 111.07, 67.79, 54.93, 49.26, 44.17, 33.29, 27.19, 21.08.
HRMS (ESI) m/z calcd. for $C_{19}H_{21}NO$ $[M+H]^+$ 280.1699, found: 280.1687.

To a solution of compound **18** (94 mg, 0.3 mmol) in MeOH (6.0 mL) was added a 37% (w/v) aqueous solution of CH_2O (0.40 mL). After the mixture was stirred for 1 h at room temperature, it was cooled to 0 ^oC. $NaBH_4$ (350 mg, 9 mmol) was added in four portions. The reaction mixture was warmed to room temperature and stirred for additional 1 h. After that, 1N NaOH solution (20 mL) was added and the mixture was extracted with ethyl acetate (2 × 30 mL). The combined organic phase was dried over $MgSO_4$, filtered and concentrated to afford the crude product, which was purified by flash column chromatography to give **21** (91 mg, 93%) as a white solid.
The synthesis of **22** and **23** is similar to that of **21**.

11-bromo-15-methyl-6,7,8,13-tetrahydro-5H-5,13-(epiminomethano)dibenzo[a,d][9]annulene

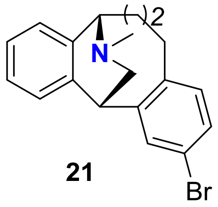

21

(93% yield), ^{1}H NMR (400 MHz, $CDCl_3$) δ 7.43 (d, *J* = 2.1 Hz, 1H), 7.27 – 7.11 (m, 4H), 7.02 (d, *J* = 7.2 Hz, 1H), 6.79 (d, *J* = 8.2 Hz, 1H), 4.11 (t, *J* = 2.5 Hz, 1H), 3.80 (d, *J* = 6.4 Hz, 1H), 3.03 (dd, *J* = 10.4, 3.2 Hz, 1H), 2.92 (dd, *J* = 10.4, 2.6 Hz, 1H), 2.34 (s, 3H), 2.25 – 2.19 (m, 1H), 2.12 – 2.04 (m, 1H), 1.84 – 1.70 (m, 4H).
^{13}C NMR (100 MHz, $CDCl_3$) δ 144.85, 144.08, 142.44, 135.84, 134.36, 133.59, 129.19, 128.05, 127.14, 126.70, 126.06, 119.01, 61.85, 58.55, 48.81, 43.29, 33.73, 28.35, 25.64.
HRMS (ESI) m/z calcd. for $C_{19}H_{20}BrN$ $[M+H]^+$ 342.0852, found: 342.0842.

12-bromo-16-methyl-5,6,7,8,9,14-hexahydro-5,14-(epiminomethano)dibenzo[a,d][10]annulene

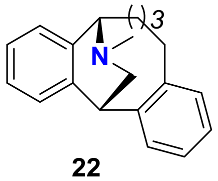

22

(82% yield), ^{1}H NMR (400 MHz, $CDCl_3$) δ 7.36– 7.15 (m, 7H), 7.02 (d, *J* = 7.4 Hz, 1H), 4.25 (s, 1H), 3.73 (d, *J* = 5.0 Hz, 1H), 3.14 – 3.06 (m, 2H), 2.53 – 2.46 (m, 1H), 2.33 (s, 3H), 2.13 – 2.07 (m, 1H), 1.91 – 1.75 (m, 3H), 1.61 – 1.55 (m, 1H), 1.44 – 1.32 (m, 1H), 1.22 – 1.14 (m, 1H).
^{13}C NMR (100 MHz, $CDCl_3$) δ 143.15, 142.62, 141.57, 138.30, 131.82, 130.79, 128.78, 127.10, 126.75, 125.97, 125.89, 125.37, 63.14, 61.37, 47.94, 43.85, 35.27, 30.81, 30.34, 21.01.
HRMS (ESI) m/z calcd. for $C_{20}H_{23}N$ $[M+H]^+$ 278.1903, found: 278.1895.

17-methyl-6,7,8,9,10,15-hexahydro-10,15-(epiminomethano)dibenzo[b,e][1]oxacycloundecine

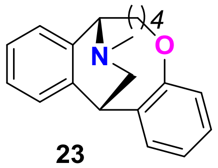

23

(88% yield), ^{1}H NMR (400 MHz, $CDCl_3$) δ 7.32 (dd, *J* = 7.4, 1.6 Hz, 1H), 7.21 (td, *J* = 7.9, 1.7 Hz, 1H), 7.15 (d, *J* = 7.4 Hz, 1H), 7.10 (td, *J* = 7.3, 1.3 Hz, 1H), 7.05 (td, *J* = 7.3, 1.5 Hz, 1H), 6.95 – 6.91 (m, 2H), 6.71 (d, *J* = 7.5 Hz, 1H), 4.06 (t, *J* = 7.0 Hz, 1H), 3.87 (td, *J* = 9.7, 2.5 Hz, 1H), 3.80 (t, *J* = 7.1 Hz, 1H), 3.52 – 3.48 (m, 1H), 3.40 (dd, *J* = 12.6, 6.5 Hz, 1H), 3.04 (dd, *J* = 12.7, 7.5 Hz, 1H), 2.45 (s, 3H), 2.40 – 2.34 (m, 1H), 2.21 – 2.13 (m, 1H), 1.77 – 1.71 (m, 1H), 1.67 – 1.58 (m, 1H), 1.53 – 1.46

(m, 1H), 1.11 – 1.02 (m, 1H).
^{13}C NMR (100 MHz, $CDCl_3$) δ 156.93, 138.57, 138.09, 134.47, 130.75, 128.16, 127.96, 127.33, 125.85, 124.68, 120.14, 113.41, 68.69, 62.88, 53.76, 43.25, 42.23, 28.56, 27.47, 22.10.
HRMS (ESI) m/z calcd. for $C_{20}H_{23}NO$ $[M+H]^+$ 294.1852, found: 294.1843.

Transformation of 4A to lactams 24 and 25

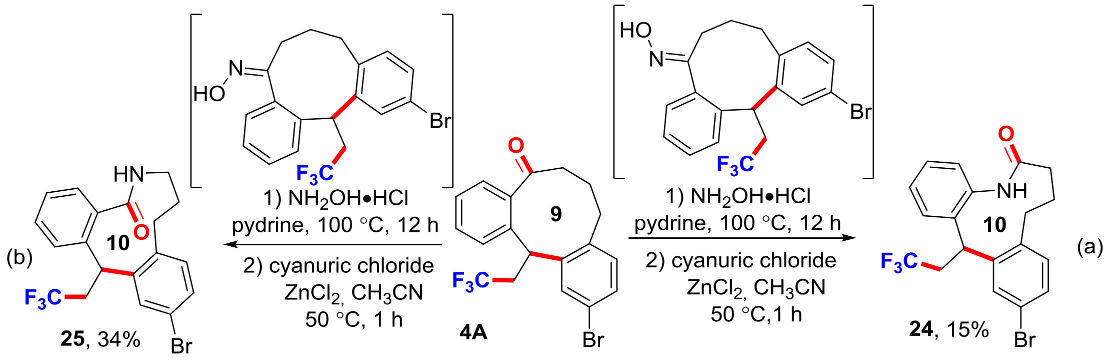

The solution of **4A** (118 mg, 0.30 mmol) and hydroxylamine hydrochloride (100 mg, 1.5 mmol) in pyridine (2.0 mL) was heated up to 100 °C for 12 h. Solvent was removed to afford the crude product, which was purified by flash column chromatography to afford **Oxime-I** (25 mg, 20%) and **Oxime-II** (60 mg, 50%).

To a solution of **Oxime-I** (24 mg, 0.06 mmol) in CH_3CN (1.0 mL) were added $ZnCl_2$ (7.2 mg, 0.05 mmol) and cyanuric chloride (9.6 mg, 0.05 mmol). The reaction solution was heated up to 50 °C for 1 h. Saturated $NaHCO_3$ (5 mL) was added to quench the reaction. EtOAc was used to extract the product from the aqueous layer (3 × 15 mL). The combined organic layer was washed with brine (5 mL), dried over anhydrous Na_2SO_4, filtered and concentrated to afford the crude product, which was purified by flash column chromatography to afford the desired product **24** (18 mg, 75%) as a white solid.

The synthesis of **25** (68% yield) is the similar to that for **24**.

12-bromo-14-(2,2,2-trifluoroethyl)-7,8,9,14-tetrahydrodibenzo[b,e]azecin-6(5H)-one

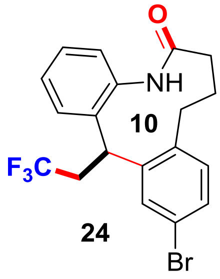

(0.88:0.12 rotamer) major: ^{1}H NMR (500 MHz, $CDCl_3$) δ 7.78 (s, 1H), 7.77 (d, J = 9.3 Hz, 1H), 7.45 (t, J = 7.5 Hz, 1H), 7.41 (brs, 1H), 7.35 (d, J = 8.3 Hz, 1H), 7.32 –

7.26 (m, 1H), 7.18 (d, J = 7.5 Hz, 1H), 7.06 (d, J = 8.3 Hz, 1H), 5.20 – 5.11 (m, 1H), 2.99 – 2.86 (m, 2H), 2.86 – 2.70 (m, 2H), 2.55 – 2.50 (m, 1H), 1.99 – 1.82 (m, 2H), 1.57 – 1.47 (m, 1H).

^{13}C NMR (125 MHz, $CDCl_3$) δ 175.92, 142.55, 140.76, 138.95, 134.83, 132.76, 130.63, 129.67, 129.45, 128.94, 128.22, 127.90, 125.93 (q, J = 278 Hz), 120.59, 41.76 (q, J = 28.5 Hz), 32.45 (q, J = 2.9 Hz), 29.91, 28.81, 28.08.

^{19}F NMR (376 MHz, $CDCl_3$) δ -64.40.

HRMS (APCI) m/z calcd. for $C_{19}H_{18}BrF_3NO$ $[M+H]^+$ 412.0524, found 412.0511.

12-bromo-14-(2,2,2-trifluoroethyl)-6,7,8,9-tetrahydrodibenzo[c,f]azecin-5(14H)-one

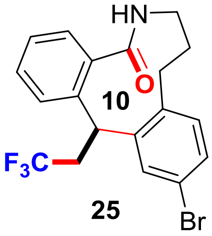

^{1}H NMR (500 MHz, $CDCl_3$, major rotamer) δ 7.53 (t, J = 7.9 Hz, 1H), 7.39 (s, 1H), 7.35 – 7.24 (m, 4H), 7.07 (d, J = 8.3 Hz, 1H), 5.13 (d, J = 9.8 Hz, 1H), 4.88 – 4.83 (m, 1H), 4.45 – 4.26 (m, 1H), 3.21 – 3.10 (m, 1H), 2.95 – 2.65 (m, 4H), 2.40 – 2.32 (m, 1H), 1.88 – 1.81 (m, 1H).

^{13}C NMR (125 MHz, $CDCl_3$, mixture) δ 169.88, 142.62, 142.58, 141.62, 138.26, 138.15, 137.63, 135.40, 132.73, 132.66, 130.63, 130.50, 130.42, 130.32, 130.23, 129.62, 128.02, 127.62, 127.24, 126.67, 126.63, 126.09 (q, J = 276 Hz), 125.98, 120.67, 120.00, 40.85, 40.66, 40.63, 40.20, 36.07, 36.05, 32.20, 30.18, 27.95, 26.91.

^{19}F NMR (376 MHz, $CDCl_3$, mixture) δ -63.93, -64.25.

HRMS (APCI) m/z calcd. for $C_{19}H_{18}BrF_3NO$ $[M+H]^+$ 412.0524, found 412.0506.

Experimental procedure for transformation of 3Aa to 26

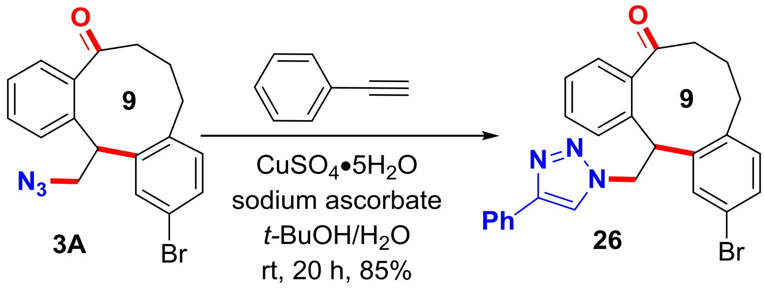

To a solution of **3A** (37 mg, 0.1 mmol, 1.0 equiv), phenylacetylene (18 mg, 0.15 mmol, 1.5 equiv) in H_2O/t-BuOH (1 mL/1 mL) were added $CuSO_4·5H_2O$ (12 mg, 0.05 mmol, 0.5 equiv) and sodium ascorbate (21 mg, 0.11 mmol, 1.1 equiv). The resulting mixture was stirred at room temperature for 20 h. Then the mixture was diluted with ethyl acetate (30 mL), saturated aqueous EDTA solution (0.2 mL) and water (5 mL). The aqueous layer was extracted with ethyl acetate (3 × 5 mL). The combined organic layer was dried over Na_2SO_4, filtered through a short silica gel plug,

and concentrated to afford the crude product, which was purified by flash column chromatography to afford the product **26** (40 mg, 85%).

11-bromo-13-((4-phenyl-1H-1,2,3-triazol-1-yl)methyl)-6,7,8,13-tetrahydro-5H-dibenzo[a,d][9]annulen-5-one

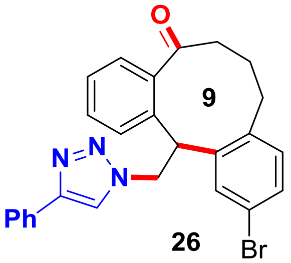

26

^{1}H NMR (500 MHz, DMSO) δ 8.54 (s, 1H), 7.77 (d, J = 8.0 Hz, 1H), 7.73 (d, J = 7.5 Hz, 2H), 7.56 (t, J = 7.5 Hz, 1H), 7.42 (t, J = 7.5 Hz, 2H), 7.32 (dd, J = 16.0, 7.5 Hz, 2H), 7.23 (td, J = 10.5, 2.0 Hz, 2H), 7.06 (d, J = 7.0 Hz, 1H), 6.94 (d, J = 8.5 Hz, 1H), 5.46 (dd, J = 9.5, 6.0 Hz, 1H), 5.38 (dd, J = 13.5, 5.5 Hz, 1H), 5.23 (dd, J = 13.5, 10.0 Hz, 1H), 3.12 (t, J = 12.5 Hz, 1H), 2.83 (td, J = 13.0, 3.0 Hz, 1H), 2.50 - 2.44 (m, 1H), 2.36 - 2.29 (m, 1H), 2.19 - 2.10 (m, 1H), 1.85 - 1.75 (m, 1H).
^{13}C NMR (125 MHz, DMSO) δ 209.18, 146.11, 143.49, 142.58, 139.00, 137.92, 131.97, 130.61, 130.47, 130.45, 130.40, 128.98, 127.94, 127.08, 126.42, 125.17, 125.10, 121.63, 119.8, 52.75, 41.55, 40.36, 30.65, 28.07.
HRMS (APCI) m/z calcd. for $C_{26}H_{23}BrN_3O$ [M+H]$^+$ 472.1024, found 472.1019.

Experimental procedure for transformation of 4G to 27

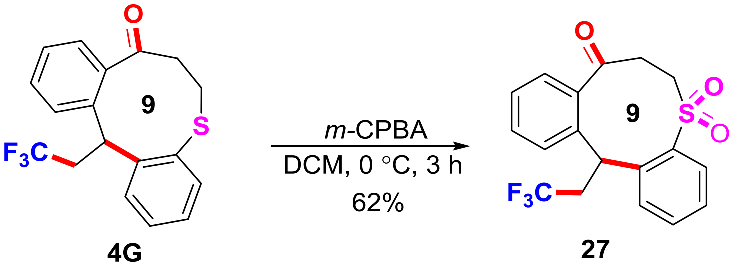

To a solution of **4G** (67 mg, 0.2 mmol, 1.0 equiv) in DCM (2.0 mL) was added *m*-CPBA (110 mg, 0.5 mmol, 2.5 equiv) at 0 °C. The reaction solution was stirred at 0 °C for 3 h. After completion (monitored by TLC), saturated $NaHCO_3$ (10 mL) was added to quench the reaction. EtOAc was used to extract the product from the aqueous layer (3 × 15 mL). The combined organic layer was washed with brine (5 mL), dried over anhydrous Na_2SO_4, filtered and concentrated to afford the crude product, which was purified by flash column chromatography to afford the product **27** (46 mg, 62%).

13-(2,2,2-trifluoroethyl)-6,7-dihydrodibenzo[b,e]thionin-8(13H)-one 5,5-dioxide

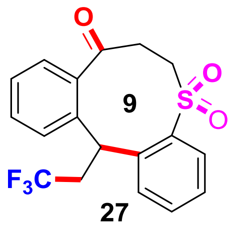

27

^{1}H NMR (500 MHz, $CDCl_3$) δ 8.02 (d, *J* = 8.0 Hz, 1H), 7.63 - 7.55 (m, 2H), 7.44 (t, *J* = 7.0 Hz, 1H), 7.38 (t, *J* = 8.0 Hz, 1H), 7.34 (t, *J* = 7.5 Hz, 1H), 7.16 (d, *J* = 7.5 Hz, 1H), 6.85 (d, *J* = 7.5 Hz, 1H), 6.18 (t, *J* = 6.5 Hz, 1H), 3.95 (dd, *J* = 16.5, 13.0 Hz, 1H), 3.86 (t, *J* = 13.5 Hz, 1H), 3.50 (dd, *J* = 14.0, 7.0 Hz, 1H), 3.33 - 3.22 (m, 1H), 2.81 - 2.71 (m, 2H).

^{13}C NMR (125 MHz, $CDCl_3$) δ 204.52, 143.99, 142.31, 138.67, 136.11, 134.95, 131.25, 130.87, 130.10, 128.50, 128.16, 127.57, 125.69 (q, *J* = 276.4 Hz), 124.85, 53.90, 38.20 (q, *J* = 29.0 Hz), 36.32, 36.23 (q, *J* = 2.5 Hz).

^{19}F NMR (376 MHz, $CDCl_3$) δ -62.27.

HRMS (APCI) m/z calcd. for $C_{18}H_{16}F_3O_3S$ $[M+H]^+$ 369.0722, found 369.0767.

Principal component analysis

Principal component analysis (PCA) of 52 our prepared compounds (Supplementary Figure 4), 27 benzannulated medium-ring natural products (Supplementary Figure 5), 47 brand-name small molecule drugs of 2006 (Supplementary Figure 6), 60 diverse natural products (Supplementary Figure 7), and 20 commercial available drug-like library compounds in the Molecular Libraries Small Molecule Repository (Supplementary Figure 8) based on 19 structural and physicochemical parameters (Supplementary Table 8) has been done by following literature protocols.[4,5] The original 19-dimensional data set is projected onto three unitless, orthogonal axes that represent linear combinations of the original 19 parameters. Because several parameters are highly correlated, 71% of the total variation is represented in the first three principal components (Supplementary Table 9 and Figure 9). Overall, parameters associated with increasing molecular size shift molecules to the right along the x axis (PC1). Increased aqueous solubility shifts molecules up along the y axis (PC2) while increased aromatic ring, ring system size and complexity shift molecules downward. Stereochemical complexity shifts molecules forward along the z axis (PC3) while increased nitrogen content shifts molecules rearward. PCA results are shown in Figure S10. Drug-like compouds (20-C) and drugs (47-D) cluster in a distinct region of the plot, while diverse natural products (60-N) occupy a distinct, larger, and more dispersed area. Benzanuulated medium-ring natural products (27-B) overlap with our prepared compouds (52-M), drug-like compouds (20-C) and drugs (47-D).

MTT cell proliferation assay

The anti-proliferative activities of compounds (**3A**, **3I**, **13** and **15**) on cell lines of 293T (derivative of human embryonic kidney) and H1299 (human non-small cell lung carcinoma) were measured by using the MTT (3-(4,5-dimethylthiazol-2-yl)-2,5-diphenyltetrazolium bromide) cell proliferation assay. Cells were seeded in 96-well microtiter plates at a density of 8,000 or 10,000 cells per well and were incubated overnight for attachment before being exposed to each of the compounds (final concentrations ranging from 5μM ~ 200μM) for additional 48 hours. The viability of treated cell lines was tested by adding 100μL MTT solution (0.5 mg/mL in PBS; Sigma-Aldrich). Plates were then incubated for 4 hours at 37°C until purple formazan precipitate is visible. 100μL of DMSO was added into each well and plates were left at room temperature in the dark for 2 hours to dissolve the precipitate. Absorbance at 570nm of each well was recorded on an automated micro-plate spectrophotometer (EnSpire® Multilabel reader 2300, PerkinElmer). Assays were carried out in triplicates and were repeated twice. The half maximal inhibitory concentration (IC_{50}) was calculated using the software of GraphPad Prism 6 (version 6.01).

Supplementary Methods

All reactions were carried out under argon using Schlenk techniques. Reagents were purchased at the commercial quality and used without further purification. Analytical thin layer chromatography (TLC) was performed on precoated silica gel 60 GF254 plates. Flash column chromatography was performed using Tsingdao silica gel (60, particle size 0.040-0.063 mm). Visualization on TLC was achieved by use of UV light (254 nm) or iodine. NMR spectra were recorded on a Bruker DPX 400 spectrometer at 400 MHz for ^{1}H NMR, 100 MHz for ^{13}C NMR and 376 MHz for ^{19}F NMR in $CDCl_3$ with tetramethylsilane (TMS) as internal standard. Microwave irradiation experiments were carried out in a dedicated Biotage Initator Robot 8 auto microwave apparatus. The chemical shifts are expressed in ppm and coupling constants are given in Hz. Data for ^{1}H NMR are recorded as follows: chemical shift (ppm), multiplicity (s, singlet; d, doublet; t, triplet; q, quartet; m, multiplet), coupling constant (Hz), integration. Data for ^{13}C NMR are reported in terms of chemical shift (δ, ppm). ^{19}F NMR spectra were recorded on a Bruker DPX 400 MHz spectrometer ($CFCl_3$ as an external reference (0 ppm)). Mass spectrometric data were obtained using Bruker Apex IV RTMS.

To disclose the reaction mechanisms, density functional theory (DFT) studies have been performed with the Gaussian 09 Program using the M11 method. For C, H O, F atoms, the 6-31+G** basis set were used and for Br atom, the Aug-cc-PVTZ basis set was used and for Cu atom, the Sdd basis set were used. Structures were optimized with the SMD method in 1,4-dioxane. Harmonic vibration frequency calculations (298 K, 1 atm) confirmed the optimized stationary points are either minima (having no imaginary vibration) or transition states (having one imaginary vibration).

Supplementary References:

1. Shibasaki, M. & Kanai, M. Asymmetric Synthesis of Tertiary Alcohols and α-Tertiary Amines via Cu-Catalyzed C−C Bond Formation to Ketones and Ketimines. *Chem. Rev.* **108**, 2853–2873 (2008).

2. Hatano, M., Miyamoto, T. & Ishihara, K. Highly Active Chiral Phosphoramide-Zn(II) Complexes as Conjugate Acid-Base Catalysts for Enantioselective Organozinc Addition to Ketones. *Org. Lett.* **9**, 4535–4538 (2007).

3. Osakama, K. & Nakajima, M. Asymmetric Direct 1,2-Addition of Aryl Grignard Reagents to Aryl Alkyl Ketones. *Org. Lett.* **18**, 236–239 (2016).

4. Bauer, R. A., Wurst, J. M. & Tan, D. S. Expanding the range of ‘druggable’ targets with natural product-based libraries: an academic perspective. *Curr. Opin. Chem. Biol.* **14**, 308–314 (2010).

5. Ibbeson, B. M., *et al.* Diversity-oriented synthesis as a tool for identifying new modulators of mitosis. *Nat. Commun.* **5**, 3155 (2014).
