# Supplementary material for: Radical aryl migration enables diversity-oriented synthesis of structurally diverse medium/macro- or bridged-rings
Source: Nat Commun. 2016 Dec 22;7:13852. doi: 10.1038/ncomms13852 (PMC5192181; doi:10.1038/ncomms13852)
Supplement: Supplementary Dataset 1 — Calculated total energies and geometrical coordinates for structures. [file ncomms13852-s2.pdf]

Calculated total energies and geometrical coordinates for structures in the main text (M11/6-31+G/Aug-cc-PVTZ in 1,4-dioxane).**

1Q_1

E(UM11) = -3721.67916485 (Hartree/Particle)
Zero-point correction= 0.344336
Thermal correction to Gibbs Free Energy= 0.289592
Sum of electronic and thermal Enthalpies= -3721.310969
Sum of electronic and thermal Free Energies= -3721.389573

C,0,1.680171756,-1.3045430787,1.3081693763
C,0,0.224471555,-0.8225892733,1.2949531938
C,0,0.5021787789,0.2793855064,3.5745920724
C,0,1.7677207085,-0.562700597,3.7301938118
C,0,2.5172031281,-0.6112912653,2.4007520364
H,0,0.7915001257,1.3343525586,3.4215615166
H,0,-0.109339279,0.2548883298,4.4876538009
H,0,2.4101374942,-0.139206668,4.5142198316
H,0,1.5074298519,-1.5833257001,4.0559191982
H,0,2.7782200739,0.4110000788,2.0860573148
H,0,3.456141674,-1.1761144327,2.4839862441
C,0,2.3852629674,-1.1572716278,-0.045731424
C,0,3.0699393931,-2.2617417505,-0.5645414141
C,0,3.1476676722,0.1260524719,-1.9464121777
C,0,3.7815532406,-2.1800626018,-1.7585731165
H,0,3.0342792356,-3.2021031943,-0.0175283514
C,0,3.823209105,-0.9773170501,-2.457146996
H,0,4.3037199693,-3.0595956237,-2.1387518919
H,0,4.3786985746,-0.8950371785,-3.3928574695
C,0,-0.3401546284,-0.1596656449,2.3945040619
C,0,-1.7125582998,0.1251024706,2.3755657072
H,0,-2.1609571498,0.6314656686,3.2337398481
C,0,-2.5145454362,-0.2133286839,1.2921512721
H,0,-3.5791284074,0.0217738669,1.2873351664
C,0,-1.9228311821,-0.8492420711,0.2039427941
C,0,-0.5739927836,-1.1671448817,0.1986221215
H,0,-0.1266262171,-1.6784437658,-0.6564197767
H,0,3.1737219247,1.0764149158,-2.4869859432
C,0,2.4255028556,0.0604416666,-0.7497110922
C,0,1.6806887279,1.3202668427,-0.3352068866
H,0,2.3604448476,2.181617947,-0.4818533052
C,0,0.4320137823,1.5147528822,-1.1400114714
O,0,1.637231214,-2.7129008981,1.602762477

H,0,0.9426101028,-2.8734847863,2.2530347312
Br,0,-2.9796802862,-1.2635647237,-1.3215445516
H,0,1.4243313416,1.3290147038,0.7338174969
C,0,-0.7382999338,2.2494713242,-0.5766894537
H,0,-1.657236151,2.0634728192,-1.1493077517
H,0,-0.9180532791,1.9589712239,0.4716697195
C,0,-0.5301927981,3.749979021,-0.55953524
F,0,0.5600630101,4.1025151249,0.1737913947
F,0,-1.5893855593,4.4056742825,-0.018115211
F,0,-0.3448232271,4.2655405124,-1.8001912641
H,0,0.4055004082,1.1794035945,-2.1768061646

TS_{1Q1}

E(UM11) = -3721.65897816(Hartree/Particle)
Zero-point correction= 0.345222
Thermal correction to Gibbs Free Energy= 0.293032
Sum of electronic and thermal Enthalpies= 3721.291227
Sum of electronic and thermal Free Energies= -3721.365946
Imaginary Frequency= -471.3882 cm^{-1}

C,0,1.7425727423,-0.7974552005,1.0812505262
C,0,0.2614987727,-0.4892656844,0.7792888578
C,0,0.0344417179,0.6960437854,3.0312165411
C,0,1.4515775907,0.2238630921,3.3742738473
C,0,2.3297026336,0.1720119693,2.1221767961
H,0,0.0702647455,1.7715545193,2.7813918534
H,0,-0.6292895552,0.6089392924,3.9027543075
H,0,1.8972665092,0.8981870595,4.1184828284
H,0,1.4162793466,-0.7742825373,3.837365192
H,0,2.4337962804,1.1798213579,1.6959339746
H,0,3.3392132402,-0.1869574998,2.3668935176
C,0,2.5787971684,-0.8523296139,-0.196688889
C,0,3.3011912993,-2.0095917192,-0.5004347198
C,0,3.3760931413,0.1249500885,-2.2569657058
C,0,4.0604313915,-2.0998215799,-1.6654344111
H,0,3.2535940769,-2.8522874398,0.1869171107
C,0,4.0958514228,-1.0291951882,-2.5532747289
H,0,4.6150459387,-3.0146653146,-1.880432797
H,0,4.6760989149,-1.0904820817,-3.4754299991
C,0,-0.571353672,-0.0548435487,1.8653683239
C,0,-1.9439891295,-0.2625089002,1.792973427
H,0,-2.5781881984,0.1100130898,2.6012029832
C,0,-2.5366100903,-0.9592327085,0.7338171233

H,0,-3.6130686985,-1.1215690939,0.6994547201
C,0,-1.7034292227,-1.4720975595,-0.2648058579
C,0,-0.3372791598,-1.2895628422,-0.2401519853
H,0,0.2972910594,-1.7430726021,-1.0036934257
H,0,3.3956324291,0.9701559157,-2.9496177505
C,0,2.6222391943,0.2337952532,-1.0849255493
C,0,1.9253378067,1.5528946366,-0.7975545078
H,0,1.8610191353,2.1063204614,-1.7469979101
C,0,0.5468878016,1.4744036803,-0.1814166755
O,0,1.7709080213,-2.1159654767,1.6528884196
H,0,0.949622788,-2.2674909186,2.137271673
Br,0,-2.4797205645,-2.474425167,-1.6840365089
H,0,2.5788759794,2.1531903751,-0.1463201867
H,0,0.4152913703,2.0558753471,0.7334460207
C,0,-0.6425786705,1.5399446259,-1.1033168055
H,0,-0.4705746333,0.9847021468,-2.0370869029
H,0,-1.5430536277,1.134183032,-0.6197197507
C,0,-0.996384838,2.9617497364,-1.477219579
F,0,-1.290834281,3.7151867403,-0.3844971942
F,0,0.0075616333,3.6082640629,-2.1272290951
F,0,-2.08097595,3.0157101084,-2.2900629372

$2Q_1$

E(UM11) = -3721.69823342 (Hartree/Particle)
Zero-point correction= 0.347092
Thermal correction to Gibbs Free Energy= 0.295610
Sum of electronic and thermal Enthalpies=-3721.328741
Sum of electronic and thermal Free Energies= -3721.402623

C,0,1.517522053,-0.6547694917,1.2967011671
C,0,0.1489248972,-0.0265300123,0.8467249599
C,0,-0.1186149668,0.9299350825,3.2176759264
C,0,1.2068806347,0.2849607625,3.6556594587
C,0,2.153345024,0.1316010414,2.4648978438
H,0,0.0772186896,1.971940552,2.9060508872
H,0,-0.8296792913,0.9746790651,4.0539103615
H,0,1.6835847447,0.8905809135,4.4390168695
H,0,1.0023846973,-0.7027457603,4.0994853778
H,0,2.4891010627,1.1178294216,2.1095949444
H,0,3.0584639492,-0.4206984322,2.7574797756
C,0,2.50702972,-0.7069210975,0.1432948573
C,0,3.5393955961,-1.6510230625,0.1905119053
C,0,3.425673503,0.1438473558,-1.9161266997

C,0,4.5080232245,-1.6970800865,-0.8042415434
H,0,3.5618771291,-2.3612090132,1.0187245386
C,0,4.452649853,-0.790056249,-1.864435864
H,0,5.305084257,-2.4406717233,-0.7558223877
H,0,5.2078983644,-0.8149756045,-2.6517545731
C,0,-0.7205962273,0.1525955089,2.0779716628
C,0,-1.9797023401,-0.3799221816,2.1519222858
H,0,-2.572598916,-0.2085349951,3.0540675856
C,0,-2.5488800884,-1.1404226533,1.1025856779
H,0,-3.5587969215,-1.537741839,1.1771273219
C,0,-1.7472604656,-1.4110713801,-0.0341267303
C,0,-0.4847942215,-0.9387199303,-0.1771399695
H,0,0.1154349516,-1.2263998856,-1.0414784421
H,0,3.3721403557,0.8506021206,-2.748352065
C,0,2.4427395232,0.1998946556,-0.9191855359
C,0,1.3172537036,1.2044279317,-1.0649002686
H,0,0.6818249815,0.9016335474,-1.9153107825
C,0,0.4479924322,1.3661655581,0.1878813726
O,0,1.2857719188,-1.999974173,1.7181656047
H,0,0.4727748136,-2.0334169571,2.2423931877
Br,0,-2.4880438885,-2.5273234986,-1.391657233
H,0,1.7438163801,2.1794958577,-1.3416718824
H,0,1.0028479029,1.9667767859,0.9219540087
C,0,-0.8733020355,2.0907055069,-0.1045696219
H,0,-1.5013516702,1.5113408242,-0.7981439242
H,0,-1.4445634332,2.2361475009,0.8227299722
C,0,-0.6942589233,3.4621498876,-0.7050849951
F,0,0.249128575,4.1921299813,-0.0498656168
F,0,-0.3188599875,3.4331731998,-2.0101258727
F,0,-1.8449910096,4.1812125296,-0.6563453083

TS_{2Q1}

E(UM11) = -3721.66912490 (Hartree/Particle)
Zero-point correction= 0.346428
Thermal correction to Gibbs Free Energy= 0.294946
Sum of electronic and thermal Enthalpies= -3721.300499
Sum of electronic and thermal Free Energies= -3721.374179
Imaginary Frequency= -385.2507cm^{-1}

C,0,1.0956829157,1.2830458668,-1.2646035986
C,0,-0.2665686279,-0.2991839297,-0.6060442886
C,0,0.1544879944,-1.1628225659,-2.9593656201
C,0,1.1711042619,-0.1005672029,-3.4341862992

C,0,1.955652038,0.5853120762,-2.3037507219
H,0,0.6838202431,-2.0446913933,-2.5681500256
H,0,-0.4327030468,-1.5066319813,-3.8224229446
H,0,1.8851557114,-0.5572230708,-4.1339015066
H,0,0.6274260779,0.6642257253,-4.0153822887
H,0,2.6392982527,-0.1176286853,-1.8090451454
H,0,2.6010735199,1.3660131216,-2.7469766807
C,0,1.7452063617,1.7471371162,-0.0127138679
C,0,2.0873619599,3.1067169559,0.0861002823
C,0,2.6807445324,1.4073177223,2.1814323519
C,0,2.7355426249,3.6059339132,1.2069725114
H,0,1.8316147181,3.7679706471,-0.7429674072
C,0,3.0447329667,2.7449482568,2.2612059435
H,0,2.9982974811,4.6636046517,1.2607866669
H,0,3.5543761994,3.1199251091,3.1503299493
C,0,-0.7655721695,-0.5565762368,-1.9263841952
C,0,-1.9912542764,-0.0298084513,-2.3088911912
H,0,-2.3375828948,-0.1887549108,-3.334057172
C,0,-2.8049985615,0.6900332641,-1.420194212
H,0,-3.7700774175,1.08653958,-1.7316019207
C,0,-2.3440823623,0.8644485307,-0.1173885678
C,0,-1.1330278144,0.3563706155,0.3128414395
H,0,-0.8214116229,0.4999719984,1.3483737498
H,0,2.8930100435,0.7380120392,3.019629397
C,0,2.0233168664,0.8849933156,1.0586888179
C,0,1.6035160437,-0.5730530026,1.1483344942
H,0,1.0059181027,-0.6830574273,2.0694738515
C,0,0.8311041453,-1.1927886255,-0.0258502989
O,0,0.2488868805,2.2372242607,-1.7704974208
H,0,-0.2957817817,1.8585341939,-2.4767797148
Br,0,-3.4407624237,1.800594691,1.1269845914
H,0,2.5064127181,-1.1787421763,1.3199377797
H,0,1.5462137359,-1.4332659629,-0.8180963913
C,0,0.1611113279,-2.520954773,0.3834026928
H,0,-0.5790699103,-2.3610891795,1.1810121583
H,0,-0.3654563826,-2.9535338782,-0.4793272912
C,0,1.1260852101,-3.5736370644,0.8702560189
F,0,2.2196524745,-3.6815336037,0.0672096049
F,0,1.5922180627,-3.3330135256,2.1237076431
F,0,0.5496665615,-4.8019005037,0.9089653662

3Q$_1$

E(UM11) = -3721.68859730 (Hartree/Particle)

Zero-point correction= 0.348053
Thermal correction to Gibbs Free Energy= 0.295819
Sum of electronic and thermal Enthalpies=-3721.317743
Sum of electronic and thermal Free Energies= -3721.392779

C,0,1.6100528674,1.5645707261,-1.7885504498
C,0,-0.3951793651,-0.7155016911,-0.5722212288
C,0,0.3594596662,-1.1400905467,-3.0023756349
C,0,1.2198902971,-0.0896317382,-3.732899763
C,0,2.2194895423,0.6997036519,-2.8558614979
H,0,1.0020233189,-1.9185391445,-2.572471858
H,0,-0.2685705903,-1.6423882716,-3.7541978932
H,0,1.7859264444,-0.5846185059,-4.5355840086
H,0,0.5429101699,0.6240615076,-4.2319370118
H,0,2.9888163487,0.0415066948,-2.4418056583
H,0,2.7729324534,1.381569771,-3.5293676183
C,0,1.7625535771,1.5335246915,-0.3663918112
C,0,1.5175405462,2.7432056258,0.3424206771
C,0,2.313137496,0.5197079635,1.7781613607
C,0,1.6893188213,2.8381426017,1.7129981651
H,0,1.2041257186,3.6185275073,-0.2239261793
C,0,2.1198806531,1.7295425437,2.4456877918
H,0,1.4998960238,3.7894574251,2.2135198705
H,0,2.2848357434,1.7984265108,3.5215964193
C,0,-0.5390457776,-0.5073266929,-1.9556286395
C,0,-1.5151830428,0.3870595511,-2.4083418641
H,0,-1.6487767498,0.5229166009,-3.4850588588
C,0,-2.2983089597,1.1325224465,-1.5363197729
H,0,-3.0384003685,1.8412138744,-1.9073746233
C,0,-2.0922106732,0.9622369413,-0.1747073219
C,0,-1.1728139039,0.0426683521,0.3098139031
H,0,-1.0452182038,-0.0486346867,1.3883171195
H,0,2.6121762414,-0.3657626945,2.34677019
C,0,2.1085711118,0.3778140071,0.4049979258
C,0,2.0807942341,-1.0360452864,-0.1341385484
H,0,2.7978530584,-1.6551550731,0.4209917143
C,0,0.659825926,-1.6725117319,-0.0263383154
O,0,0.953707163,2.6651360354,-2.2712922776
H,0,1.0210822453,2.7048649162,-3.233534214
Br,0,-3.085835659,2.0117769355,1.0616217637
H,0,2.3813929127,-1.0930978516,-1.1801098291
H,0,0.6582738266,-2.5736379565,-0.6568174157
C,0,0.3045092317,-2.1352463922,1.3961859877
H,0,0.4425452089,-1.3402443305,2.1426145456

H,0,-0.7464919104,-2.4539218113,1.4317188045
C,0,1.1119842138,-3.3154607937,1.8812343447
F,0,1.2316120848,-4.2947626981,0.9471236151
F,0,2.378902214,-2.9853623917,2.2528989108
F,0,0.5385288505,-3.8865263547,2.9714738851

$TS_{1Q1'}$

E(UM11) = -3721.65672066 (Hartree/Particle)
Zero-point correction=0.344891
Thermal correction to Gibbs Free Energy= 0.293050
Sum of electronic and thermal Enthalpies= -3721.289327
Sum of electronic and thermal Free Energies= -3721.363671
Imaginary Frequency= -473.3275 cm-1

C,0,1.4392325822,-1.1780084743,1.0302058285
C,0,0.0092397458,-0.5849175006,0.914573588
C,0,0.2648513504,0.4831935395,3.2163799468
C,0,1.4549344701,-0.4419296964,3.4754377248
C,0,2.2640792398,-0.5765945168,2.1891567007
H,0,0.6598815888,1.4800532959,2.9437479076
H,0,-0.3472825003,0.6205822453,4.1183615979
H,0,2.0883900954,-0.0378291345,4.2772744718
H,0,1.1008407714,-1.4283586098,3.816689355
H,0,2.659311144,0.4088711241,1.9088538937
H,0,3.127235613,-1.2436534883,2.3217498022
C,0,2.2667621816,-1.091790075,-0.2507603198
C,0,2.8656937506,-2.2435173548,-0.7654162451
C,0,3.3576801116,0.1967213489,-1.9791266022
C,0,3.7062257091,-2.1789736556,-1.876044124
H,0,2.6670398887,-3.1995469295,-0.2836609544
C,0,3.9597414536,-0.9538433562,-2.4837599174
H,0,4.1615397042,-3.0918809636,-2.2634987527
H,0,4.6157130476,-0.8910684795,-3.3535516496
C,0,-0.5984816235,-0.0475917641,2.0968404952
C,0,-1.9866742891,0.0123594101,2.1825005979
H,0,-2.442450652,0.4621620839,3.0680554946
C,0,-2.8139606804,-0.5025115298,1.1827911327
H,0,-3.8987458503,-0.448303898,1.2649255229
C,0,-2.2113749932,-1.1462202035,0.0929624393
C,0,-0.8455435943,-1.2527488155,-0.022239869
H,0,-0.3909215549,-1.8050446919,-0.8475291877
H,0,3.5355096609,1.1606255636,-2.4620152106
C,0,2.5147550016,0.1474540634,-0.8672422454
C,0,1.8233120729,1.4123958022,-0.4014313823

H,0,2.0511123747,2.2147166791,-1.125294589
C,0,0.337157146,1.198808426,-0.3485718804
O,0,1.2557523059,-2.5746737111,1.3144254828
H,0,0.5127710457,-2.6739075756,1.9235934351
Br,0,-3.3246930363,-1.9461584736,-1.2284551865
H,0,2.1994572535,1.7746518896,0.568551781
C,0,-0.5615589791,2.2588591819,0.2289640183
H,0,-1.603105351,1.9100711564,0.2473988653
H,0,-0.274849134,2.5435559934,1.2514482503
C,0,-0.5498474548,3.5363142532,-0.5805978422
F,0,0.6443556187,4.1841044939,-0.5075205074
F,0,-1.4890718879,4.4152989196,-0.1462474928
F,0,-0.7922762681,3.3208444463,-1.8976431096
H,0,-0.0604303277,0.7505772581,-1.2631365009

2Q$_1$´

E(UM11) = -3721.68718574 (Hartree/Particle)
Zero-point correction= 0.368674
Thermal correction to Gibbs Free Energy= 0.296173
Sum of electronic and thermal Enthalpies=-3721.317568
Sum of electronic and thermal Free Energies= -3721.391013

C,0,0.9833575613,-1.3771558231,1.0421517477
C,0,0.0186186624,-0.1488232606,0.7487765727
C,0,0.4166539957,0.5969560226,3.1881968371
C,0,1.1118565949,-0.7273624909,3.5299649436
C,0,1.873230237,-1.2084420034,2.2995793049
H,0,1.1945443747,1.3240015932,2.8909854866
H,0,-0.1115948583,1.0153667879,4.055944044
H,0,1.80592879,-0.595328913,4.3718682591
H,0,0.3625419789,-1.4712898528,3.846021007
H,0,2.6980276787,-0.5128817136,2.0942160541
H,0,2.3270527493,-2.1954254043,2.4692071176
C,0,1.9061053905,-1.6208766161,-0.1343561803
C,0,2.1144297688,-2.8956420709,-0.6569791338
C,0,3.5485845675,-0.7278170336,-1.658786347
C,0,3.0463695525,-3.0876231836,-1.6769998785
H,0,1.5403897873,-3.7324254881,-0.2582116883
C,0,3.7691572548,-2.0050321176,-2.17367347
H,0,3.2059086366,-4.0870286396,-2.0852594033
H,0,4.49739335,-2.1505498113,-2.9732793214
C,0,-0.5497151773,0.3631325178,2.0609406905
C,0,-1.8944721528,0.5680171867,2.2287262264

H,0,-2.248968308,0.9765718855,3.1785090295
C,0,-2.8450863396,0.2643779784,1.2277901984
H,0,-3.9048428787,0.4615132531,1.3748494715
C,0,-2.3837632841,-0.3736087981,0.0475950227
C,0,-1.074759228,-0.6401489988,-0.1745916185
H,0,-0.7518928474,-1.1776739995,-1.0687219808
H,0,4.094487609,0.1280642362,-2.0632859089
C,0,2.6229518012,-0.5243354274,-0.6355583935
C,0,2.2882431651,0.8584891346,-0.1220588355
H,0,2.7051673816,1.6018746265,-0.8147104646
C,0,0.7569442286,0.99203562,-0.0889588402
O,0,0.1775612584,-2.5385752621,1.2310110536
H,0,-0.5657179785,-2.3211904508,1.811170809
Br,0,-3.6841312096,-0.9140786098,-1.2383665568
H,0,2.7317699852,1.0673398536,0.8641638058
C,0,0.2200367964,2.3782938189,0.2923544529
H,0,-0.8742528888,2.389752844,0.1820407134
H,0,0.4574218157,2.6608321856,1.3268145424
C,0,0.7557780906,3.4887734447,-0.578018188
F,0,2.0311000413,3.8378817446,-0.2532101288
F,0,0.0133438561,4.6187652426,-0.4582208559
F,0,0.7697849672,3.1675024238,-1.8968656542
H,0,0.4429648282,0.8251489196,-1.1303450874

$TS_{2Q1'}$

E(UM11) = -3721.65672066 (Hartree/Particle)
Zero-point correction=0.346614
Thermal correction to Gibbs Free Energy= 0.295956
Sum of electronic and thermal Enthalpies= -3721.294585
Sum of electronic and thermal Free Energies= -3721.367285
Imaginary Frequency= -447.8922 cm-1

C,0,0.9452918237,-1.5426754765,1.0760499601
C,0,-0.2574199932,0.1312987851,0.5248470934
C,0,0.255459445,0.772824436,2.9518280624
C,0,0.8987779054,-0.5248379804,3.4718524244
C,0,1.6974174061,-1.2192772068,2.3649178791
H,0,1.0506485455,1.4346200913,2.5801350642
H,0,-0.2612859726,1.2976656449,3.7678343999
H,0,1.5726687453,-0.314218717,4.3145778339
H,0,0.1069044949,-1.1875368998,3.8604502936
H,0,2.5893851821,-0.6271004074,2.1326352316
H,0,2.0690074265,-2.192622831,2.7304408901

C,0,1.7893979133,-1.7227119852,-0.1210852975
C,0,1.8812105385,-2.9630203696,-0.7625393269
C,0,3.3448102534,-0.7982376815,-1.7168933123
C,0,2.7230055229,-3.1248546827,-1.8597999516
H,0,1.2921090685,-3.7983837569,-0.3827469431
C,0,3.4675123887,-2.0445834438,-2.3304878072
H,0,2.7975595975,-4.0977024526,-2.3485067695
H,0,4.1289652554,-2.1649061501,-3.1899520113
C,0,-0.7222612266,0.3806582128,1.870843404
C,0,-1.9831998514,-0.0479647236,2.260524113
H,0,-2.2844160684,0.1014096837,3.3016051972
C,0,-2.8813203034,-0.6667965064,1.3785256215
H,0,-3.874307086,-0.9754224193,1.7009458197
C,0,-2.4557766624,-0.8595705281,0.0620150941
C,0,-1.2175584477,-0.4494778611,-0.3756287922
H,0,-0.9357959014,-0.5962171715,-1.419652275
H,0,3.8931496647,0.0600969555,-2.1127266778
C,0,2.5088801308,-0.6172439072,-0.6142297507
C,0,2.2442433654,0.7684853579,-0.0685229975
H,0,2.8056155399,1.4857781546,-0.6811907687
C,0,0.7363177783,1.078555231,-0.1973090133
O,0,0.0133098476,-2.5406849339,1.2222856179
H,0,-0.5839997473,-2.3343291846,1.9566622272
Br,0,-3.6521746495,-1.6732572727,-1.1755057548
H,0,2.5898606731,0.90942766,0.9648695037
C,0,0.3456000848,2.5352595976,0.1241259694
H,0,-0.7091726693,2.6881449209,-0.1429399206
H,0,0.4570660753,2.7876703566,1.1860305769
C,0,1.1412853631,3.5668325948,-0.6368874688
F,0,2.3852360077,3.7590611698,-0.1172896048
F,0,0.5332185975,4.7803975543,-0.615905563
F,0,1.3167002578,3.2396417946,-1.9432388373
H,0,0.5219507905,0.9479735281,-1.2682832037

$3Q_1$´

E(UM11) = -3721.68722951 (Hartree/Particle)
Zero-point correction=0.347732
Thermal correction to Gibbs Free Energy= 0.294783
Sum of electronic and thermal Enthalpies=-3721.316505
Sum of electronic and thermal Free Energies= -3721.392446

C,0,1.5719942231,-1.8415663308,1.6875961191
C,0,-0.338661672,0.5109862293,0.2342562809

C,0,0.2013365541,0.8469041919,2.7530075393
C,0,0.9188336639,-0.2364182452,3.5779153882
C,0,2.0396587868,-0.9822819422,2.8238335332
H,0,0.9451348953,1.5765771144,2.4167326569
H,0,-0.4831116911,1.3911956894,3.4224590069
H,0,1.3545691958,0.2308984009,4.4731517422
H,0,0.1751879374,-0.9652565283,3.9410959448
H,0,2.8234192576,-0.2906545485,2.4999285365
H,0,2.5391484849,-1.6557580506,3.5465071463
C,0,1.8894264149,-1.7870395135,0.2944200761
C,0,1.7658286413,-2.9969596772,-0.4461499506
C,0,2.6259674872,-0.7341918063,-1.7704446438
C,0,2.0879381826,-3.0731882542,-1.7895888631
H,0,1.4236102702,-3.8874825011,0.078228482
C,0,2.5446623628,-1.9392194988,-2.4657842755
H,0,1.9898978312,-4.0258516646,-2.3132553448
H,0,2.8144584919,-1.9864234487,-3.5214911642
C,0,-0.6099820029,0.2823034138,1.5958818776
C,0,-1.6856427329,-0.5496522389,1.9310597487
H,0,-1.9161065929,-0.7135111094,2.9871916753
C,0,-2.4646911832,-1.1853641933,0.9724484997
H,0,-3.2903165034,-1.8364628291,1.2592943025
C,0,-2.1542756717,-0.9748101635,-0.3640865832
C,0,-1.1146793088,-0.1352190717,-0.7348855081
H,0,-0.8829186908,0.0072988963,-1.791972977
H,0,2.9348000993,0.1709644596,-2.302694915
C,0,2.2919507132,-0.6157129142,-0.4200647824
C,0,2.1941716376,0.8088337951,0.0987355258
H,0,2.9656748129,1.4069190462,-0.4053458698
C,0,0.8015260217,1.3874858796,-0.2721342583
O,0,0.8744663735,-2.9534406634,2.0780481814
H,0,0.8556796927,-3.0206879869,3.0410530052
Br,0,-3.1755556471,-1.8435874706,-1.7121854643
H,0,2.3810020428,0.9096093456,1.168837896
C,0,0.5582723433,2.8658543394,0.1007315218
H,0,-0.3379856957,3.2218166941,-0.4251801842
H,0,0.3883333365,3.0255805623,1.1724720769
C,0,1.6859913189,3.7840247377,-0.2944259772
F,0,2.767697432,3.665184105,0.5231466188
F,0,1.3143796888,5.0887479225,-0.2434335241
F,0,2.1293922687,3.5532385792,-1.5581339497
H,0,0.7561519594,1.3441770489,-1.3709767064

Cu^{II}-Species

E(UM11) = -639.528769845 (Hartree/Particle)
Zero-point correction=0.095672
Thermal correction to Gibbs Free Energy= 0.051826
Sum of electronic and thermal Enthalpies=-639.420069
Sum of electronic and thermal Free Energies= -639.476944

O,0,1.4809031037,1.8411040729,0.2814415818
C,0,0.6145761263,0.9213383058,0.0749991009
O,0,1.0667830892,-0.2484845365,-0.1280860178
C,0,-0.8336009819,1.2587521345,0.0462376491
C,0,-1.1326822448,2.6283834292,0.0114325744
C,0,-1.8800746906,0.3242913758,0.0240437085
C,0,-2.4439718594,3.0747222973,-0.060215222
H,0,-0.3037870083,3.3355076688,0.0327158638
C,0,-3.1980342715,0.7809303901,-0.0394561684
C,0,-3.479049582,2.1434238759,-0.0874132263
H,0,-2.656615063,4.1434178799,-0.0953296001
H,0,-4.0152004918,0.0593600971,-0.051476012
H,0,-4.5174875899,2.4736881593,-0.1437271629
Cu,0,2.9102644692,0.4887172134,0.0296074723
I,0,-1.6489670878,-1.782600261,0.1292858504
I,0,4.9859991227,-0.8350759224,-0.2645727518

TS-SD

E(UM11) = -4361.19964294 (Hartree/Particle)
Zero-point correction=0.441581
Thermal correction to Gibbs Free Energy= 0.366397
Sum of electronic and thermal Enthalpies= -4360.721345
Sum of electronic and thermal Free Energies= -4360.833246
Imaginary Frequency= --56.4912 cm-1
<Sx>= 0.0000 <Sy>= 0.0000 <Sz>= 0.0000 <S**2>= 0.9894 S= 0.6133

C,0,5.1822358829,0.2195517101,0.3779825626
C,0,4.4394774274,-0.6834235077,-0.6112934926
C,0,6.0871436252,-2.5866933777,-0.2232318773
C,0,7.0066653654,-1.52619807,0.3822126009
C,0,6.1817732481,-0.5685353777,1.2380633185
H,0,5.720757843,-3.2503054119,0.5803686617
H,0,6.6289194012,-3.2282915485,-0.9325473789
H,0,7.7880302029,-2.0052612835,0.9875605189

H,0,7.5042464961,-0.9552817455,-0.4152664495
H,0,5.6373332417,-1.1241478924,2.0178606929
H,0,6.8138579034,0.1730249654,1.7467121969
C,0,4.2455349455,1.0150452854,1.3111640824
C,0,4.5299992805,2.3687338218,1.519235108
C,0,2.4858775638,1.2115022548,2.9606067472
C,0,3.8050406611,3.1393550354,2.4262323518
H,0,5.3528629551,2.8175332638,0.9640105109
C,0,2.7767631047,2.5570793458,3.1580695872
H,0,4.052464848,4.1934792198,2.5596101065
H,0,2.1963955996,3.1449295772,3.8704277687
C,0,4.8876340279,-1.9767813002,-0.9188122041
C,0,4.1807469287,-2.7269245186,-1.8665834607
H,0,4.5329237103,-3.7304648503,-2.1166021864
C,0,3.0261988375,-2.2424462656,-2.4749223041
H,0,2.4581094228,-2.8535113643,-3.1763969226
C,0,2.5979931121,-0.9635000419,-2.1401472001
C,0,3.3108703395,-0.1676936891,-1.2542878847
H,0,2.9448273952,0.8331576569,-1.0011911358
H,0,1.6750713255,0.7439976738,3.5264539078
C,0,3.1991068441,0.4255626145,2.0499354635
C,0,2.7391640811,-1.0158734495,1.9092804531
H,0,2.4307594797,-1.3881218463,2.9012995862
C,0,1.6037590584,-1.1603509859,0.9378734928
O,0,5.995823699,1.1105108957,-0.3972790987
H,0,5.4726073551,1.4814542775,-1.1202871581
Br,0,0.9131065299,-0.3440725781,-2.7765668896
H,0,3.5544963905,-1.6915306811,1.5932925175
C,0,1.1361339184,-2.5089157352,0.4919067695
H,0,0.3341011402,-2.431997989,-0.2521259475
H,0,1.9722154364,-3.0741236116,0.0400525883
C,0,0.623042671,-3.3602919411,1.6345244115
F,0,1.6371636266,-3.7956797725,2.4326491585
F,0,-0.0168854503,-4.4685018493,1.1819332057
F,0,-0.2431432261,-2.7048155608,2.4404321337
H,0,1.4506292455,-0.3275568276,0.2479606899
Cu,0,-0.630043764,0.6406611853,0.155783244
I,0,0.5578761148,2.8118137335,-0.0690879064
O,0,-1.5099593759,-1.1108089709,0.3091732345
C,0,-2.6477913433,-0.5289644152,0.2040314945
O,0,-2.6278584179,0.728052585,0.037254723
C,0,-3.8950735888,-1.3331869233,0.3087424712
C,0,-5.1869707039,-0.8558799835,0.0424477514
C,0,-3.7225614186,-2.6619871863,0.7239058195

C,0,-6.2761740243,-1.7166418987,0.1940314284
C,0,-4.8095957965,-3.5100591167,0.8798868216
H,0,-2.7086516532,-3.006153711,0.9309739338
C,0,-6.0905332872,-3.0319007568,0.6119961496
H,0,-7.2823017194,-1.3537298733,-0.0171574042
H,0,-4.6581585466,-4.5376567759,1.2101742775
H,0,-6.9578821676,-3.6834039747,0.7276096173
I,0,-5.6586618449,1.1063927811,-0.615791978

INT

E(UM11) = -4361.30521999 (Hartree/Particle)
Zero-point correction=0.479373
Thermal correction to Gibbs Free Energy= 0.371676
Sum of electronic and thermal Enthalpies= -4360.824903
Sum of electronic and thermal Free Energies= -4360.933544

C,0,-2.876471772,2.9443417052,-0.5723495017
C,0,-1.5023115087,2.8467075268,0.1057454447
C,0,-2.4946867507,3.5286139647,2.3473446031
C,0,-3.6627495403,4.0927001934,1.5400364234
C,0,-4.0298731637,3.1091000399,0.4339088524
H,0,-2.8278278188,2.6258597806,2.8879186511
H,0,-2.1571117994,4.2412328957,3.1128789314
H,0,-4.5267828922,4.2670448302,2.1957823287
H,0,-3.3847188239,5.0579266636,1.0913978261
H,0,-4.2968999111,2.1336884195,0.869550528
H,0,-4.899809929,3.4498572537,-0.1451627438
C,0,-3.1905160898,1.7557367868,-1.5021269162
C,0,-3.6285185068,2.032212702,-2.8000847418
C,0,-3.5940353504,-0.5918067289,-1.9393982522
C,0,-4.0303998594,1.0175495329,-3.666505534
H,0,-3.6712819525,3.0710482603,-3.124747753
C,0,-4.0215059894,-0.302944093,-3.2328315402
H,0,-4.3650224734,1.2673867007,-4.6746114554
H,0,-4.3527663285,-1.1084248097,-3.8899254571
C,0,-1.3226504915,3.1691374471,1.458911131
C,0,-0.0221396756,3.1692092052,1.9799823821
H,0,0.1237861231,3.4102198048,3.0358013417
C,0,1.0888189158,2.9080610324,1.1844305129
H,0,2.0969741343,2.9416535205,1.5982848961
C,0,0.8885501903,2.6182280675,-0.1634881863
C,0,-0.3920926913,2.560847127,-0.699750736
H,0,-0.5296322025,2.3069477272,-1.755658377

H,0,-3.6069527118,-1.6245837257,-1.5862455369
C,0,-3.1637346323,0.41349898,-1.0685881463
C,0,-2.6550813685,-0.0030752517,0.3017219021
H,0,-3.3201141867,-0.7279475504,0.7897059646
C,0,-1.2242854835,-0.479741526,0.2618208625
O,0,-2.8768423709,4.1719142144,-1.315282344
H,0,-2.0098071092,4.3053694359,-1.7218228591
Br,0,2.3864034536,2.3602171137,-1.2965097206
H,0,-2.6134264341,0.8629960765,0.9817446455
C,0,-0.3669091738,-0.5431097635,1.498900834
H,0,0.4070776194,-1.3256029342,1.4226956092
H,0,0.189120841,0.4143802626,1.5244295218
C,0,-1.0505969243,-0.6685067779,2.8418616263
F,0,-1.8415093471,0.4062516642,3.1184661655
F,0,-0.1202011122,-0.7195324105,3.8245229759
F,0,-1.8307010384,-1.7590369948,2.966550748
H,0,-0.6630669212,-0.0345192592,-0.5711624572
Cu,0,-0.6386639246,-2.1823294919,-0.8035709735
I,0,-2.3559726592,-3.5816207167,0.2031492086
O,0,0.8388119691,-1.2283478469,-1.719136783
O,0,1.7154988612,-2.3699374904,-0.0329109523
C,0,1.8407521462,-1.5943529374,-0.9961044051
C,0,3.1928982524,-1.06749356,-1.3920408056
C,0,4.1365280296,-0.6075012536,-0.4711739024
C,0,3.5021123618,-1.0406610371,-2.7548248036
C,0,5.3805141584,-0.1543798786,-0.9075086984
C,0,4.7459931229,-0.6019774594,-3.1948492088
H,0,2.7400008091,-1.3678705838,-3.4636109528
C,0,5.6881066286,-0.1636051947,-2.2666766481
H,0,6.1076781671,0.2198133398,-0.1858994235
H,0,4.9771719575,-0.5958112134,-4.2608497014
H,0,6.6667842248,0.1874429484,-2.5978338502
I,0,3.6979839522,-0.3764037339,1.5948844511
